# Tripodal Silanolate Ligands Expand [MoX_3_] Chemistry Beyond Its Traditional Borders

**DOI:** 10.1021/jacs.5c02178

**Published:** 2025-04-11

**Authors:** Daniel Rütter, Nils Nöthling, Markus Leutzsch, Alexander A. Auer, Alois Fürstner

**Affiliations:** Max-Planck-Institut für Kohlenforschung, 45470 Mülheim/Ruhr, Germany

## Abstract

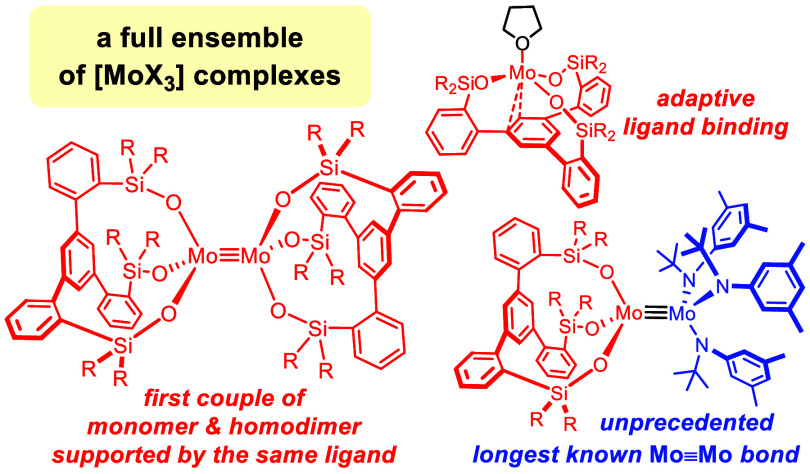

Homodimeric complexes [X_3_Mo≡MoX_3_] are commonplace, but in no case is the corresponding monomeric [MoX_3_] species known; conversely, none of the very rare monomeric complexes [MoX_3_] has the respective homodimeric analogue. This mutual exclusivity ends with the present study; on top, an entirely unprecedented class of heterodimers of type [X_3_Mo≡MoY_3_] is reported. Key to success was the use of tripodal silanolates as ancillary ligands; the fence formed by properly chosen peripheral substituents shields the sensitive Mo(+3) center; homodimerization of the resulting [MoX_3_] complexes is then kinetically strongly disfavored, though possible. The monomers are able to cleave N_2_O and convert *gem*-dihalides into metal alkylidynes; they exist in different binding modes, in which the basal phenyl ring of the ligand backbone is either completely unengaged with the central metal or tightly bound to it, depending on whether the ligand sphere is complemented by solvent molecules or not. If the latter are sufficiently labile, a surprisingly facile heterodimerization of the d^3^ electron fragments will ensue; the resulting products [X_3_Mo≡MoY_3_] incorporate the intact Cummins complex [(*t*Bu)(Ar)N]_3_Mo (Ar = 3,5-dimethylphenyl) as one of their constituents, which is famous for not engaging in metal–metal triple bonding otherwise. Heterodimerization was also observed with simple *tert*-butoxide ligands. The new type of heterodimers features unusually long yet robust Mo≡Mo bonds, which are notably polarized according to DFT. However, there is no direct correlation between the extreme Mo≡Mo bond lengths and the strikingly deshielded ^95^Mo NMR signals, since ligand-based orbitals can also markedly affect the shielding tensor.

## Introduction

The chemistry of Mo(+3) is dominated by the massive bias of [MoX_3_] units to dimerize. It is the stability of the resulting σ^2^π^4^ metal–metal triple bond between the two d^3^ electron fragments that provides a formidable thermodynamic driving force for the formation of homobimetallic complexes of the general type [X_3_Mo≡MoX_3_] (X = alkyl, (pseudo)halide, alkoxy, silyloxy, dialkylamido, etc.). Ever since the first such complex had been described by Wilkinson and co-workers in 1971,^1^ the field flourished, leading to a plethora of homoleptic representatives; in no case has the corresponding monomer [MoX_3_] ever been detected.^2,3^ Some of the dimers were transformed into heteroleptic variants by partial ligand exchange via protonolysis or salt metathesis, whereby “symmetrical” ligand distributions ([X_2_YMo≡MoYX_2_], “1,2-pattern”)^4,5^ were far more commonly attained than “unsymmetrical” ones ([XY_2_Mo≡MoX_3_], “1,1-pattern”).^6−9^ Such isomers tend not to interconvert, suggesting that the barriers for group transfer between the two neighboring molybdenum centers under thermal conditions are high in most cases.^7,10^ Heteroleptic examples of the type [X_2_YMo≡MoX_3_] or [XY_2_Mo≡MoX_3_] are rare,^11−16^ and heterodimers of the general constitution [X_3_Mo≡MoY_3_] seem to be unknown.^17^

**Figure 1:**
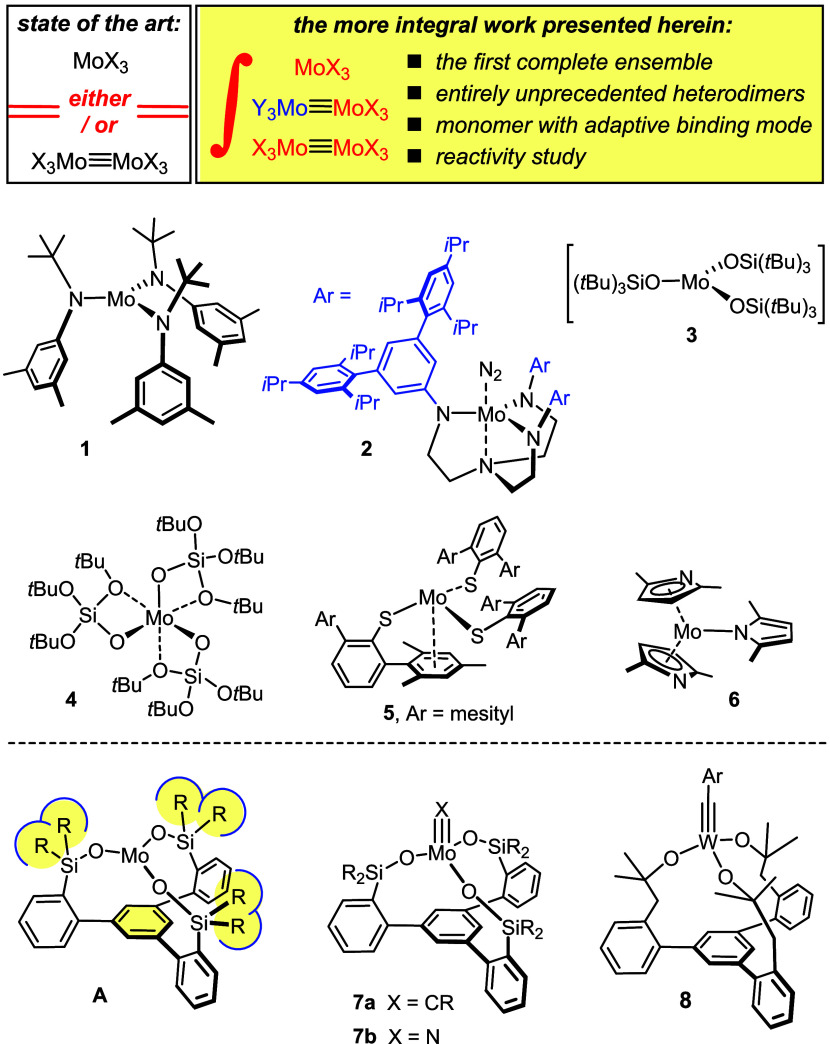
Top: the literature knows of either monomeric or homodimeric Mo(+3) complexes but in no case are both types supported by the same ligand set; the present study closes the gap and reports the first full ensemble, including an entirely new type of heterodimeric complexes; middle: known monomeric Mo(+3) complexes; bottom: envisaged use of tripodal ligands for the stabilization of monomeric Mo(+3); these ligands were previously used to craft “canopy catalysts” for alkyne metathesis and analogous Mo(+6) nitrido complexes; R = aryl, alkyl.

The challenge of obtaining a kinetically stable monomeric Mo(+3) complex was first met in 1995 by Cummins and co-workers, who disclosed the preparation, structure and stunning reactivity of the trigonal-planar complex [(*t*Bu)(Ar)N]_3_Mo (Ar = 3,5-dimethylphenyl) (**1**) (Figure 1).^18−22^ This particular trisamidomolybdenum species and its siblings are capable of activating numerous small molecules under notably mild conditions, including substrates as unreactive as N_2_ or N_2_O. The serendipitous discovery in our laboratory that **1** also reacts with CH_2_Cl_2_ to generate alkyne metathesis catalysts in situ that proved highly chemoselective and therefore appropriate for applications to polyfunctionalized substrates opened an interface to advanced organic synthesis as well.^23−27^ In any case, the strongly π-donating and very bulky amido ligands safeguard the Mo(+3) center so effectively that dimerization of **1** has never been observed, not even as a side reaction in any of the applications that this complex has found so far in the literature.

Very few other monomeric Mo(+3) complexes were described ever since. Arguably most prominent among them are complexes such as **2** comprising a trisamidoamine ligand framework,^28−30^ which served as entry point for the reductive cleavage of N_2_ with formation of NH_3_ in a catalytic mode.^31^ Though much less π-donating than amides, bulky silanolates were also shown to be adequate ligands, as witnessed by complex [(*t*Bu_3_SiO)_3_Mo] (**3**), even though the structure of **3** proper is unknown and its monomeric nature was solely inferred from a crystalline phosphine adduct.^32,33^ Complex [[*t*BuO)_3_SiO]_3_Mo] (**4**) constitutes an interesting variation on that theme: by virtue of the additional lateral oxygen atoms, each siloxide engages in weak κ^2^ binding; **4** is hence a coordinatively saturated octahedral entity, yet retains the prototypical reactivity of a monomeric Mo(+3) species toward N_2_ and other small molecules.^34^ Complex **5** bearing overcrowded, strongly π-donating thiolate ligands gains additional stability – at the expense of reactivity – by coordination of one of the lateral arene rings onto the Mo(+3) center.^35^ An as yet higher level of coordinative and electronic saturation also helps stabilize complex **6**, in which two of the three 2,5-dimethylpyrrolide ligands are η^5^-bound, thus rendering the bonding situation similar to that of a bent metallocene.^36^

Intrigued by these reports, we wondered whether tripodal silanolates or alkoxides of the types recently used to support high-valent molybdenum or tungsten alkylidynes could serve similar purposes. For the excellent synergy between these ligands and the respective high-valent metal centers, catalysts such as **7a** and **8** excel in alkyne metathesis for reasons that are fairly clear by now.^37−43^ The isolobal terminal molybdenum(+6) nitrido complexes **7b** are equally well behaved monomeric entities.^44^ In view of this encouraging precedent, we reasoned that such tripodal ligands might also be able to support Mo(+3): the phenyl ring forming the basal plane of the “canopy” ligand framework would effectively block one side of the targeted complex **A** (Figure 1), whereas the fence of upward-pointing substituents R on the tethers encircling the Mo(+3) atom potentially shields the opposite face; if properly chosen, dimerization might thus be prevented.

While this reasoning ultimately turned out to be correct, the new ligand platform actually proved far more auspicious, as it enabled the first “collective” synthesis of an entire panel of coordination compounds of this series. Specifically, it opened access to the corresponding monomeric Mo(+3) complex, the homoleptic dimer thereof with a tripodal silanolate cap on both ends, and even to heterodimeric complexes of type [X_3_Mo≡MoY_3_] that are without precedent in the literature. What makes this latter finding particularly stunning is the fact that these heterodimers comprise the intact Cummins complex **1** as one of the subunits, which is famous for not engaging in metal–metal triple bonding otherwise; the surprising ease of their formation is hence deemed remarkable, as are their structural and spectroscopic properties.

## Results and Discussion

### Monomeric Mo(+3) Complexes and a Homodimer with an Ultralong [Mo≡Mo] Bond

**Scheme 1:**
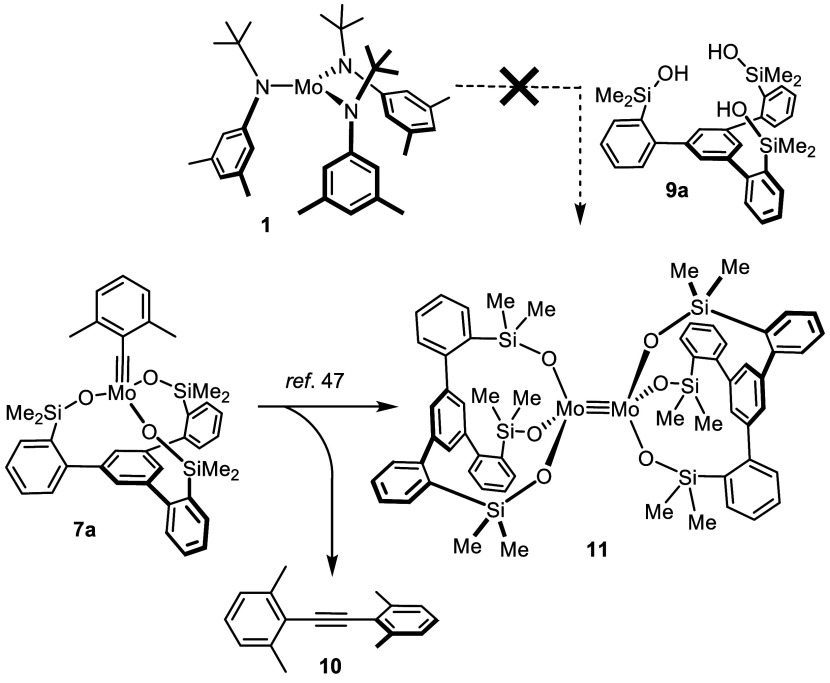
A Dumbbell Dimer with Two Tripodal Silanolate End-Caps

During our attempts at developing ever more efficient catalysts for alkyne metathesis,^45,46^ we noticed that the alkylidyne complex **7a** (R = Me) is highly active but rather short-lived. The isolation of the corresponding tolane derivative **10** suggested that bimolecular coupling of **7a** constitutes a (major) decomposition pathway; in this case, the corresponding homobimetallic dimer **11** should also be formed (Scheme 1).^47^ Although no X-ray crystal structure of this compound was obtained, the very diagnostic ^95^Mo NMR shift (δ_Mo_ = 2631.5 ppm)^48^ in combination with matching HRMS and combustion analysis data allowed the assignment to be made with confidence. This result shows that methyl substituents on the silicon linkers of the tripodal ligand framework do not suffice to prevent metal–metal bonding from occurring. Interestingly though, reaction of **1** with the free ligand **9a** failed to afford complex **11** but gave an ill-defined mixture of presumably oligomeric species.

**Figure 2:**
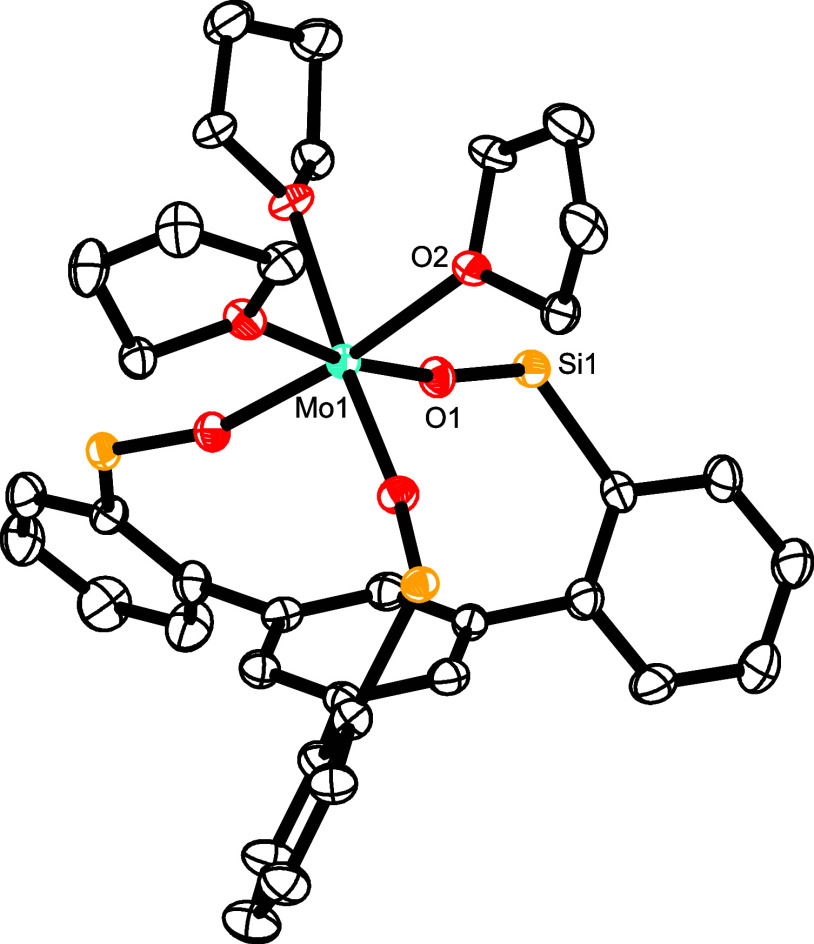
Truncated structure of complex [**12**·3thf] in the solid state; the 3,5-dimethylphenyl substituents on the silicon linkers and H-atoms are removed for clarity; the full structure is contained in the Supporting Information. Selected bond lengths (Å) and angles (°): Mo1–O1 2.0146(19), Mo1–O2 2.2407(19), Si1–O1–Mo1 163.16(17).

**Scheme 2:**
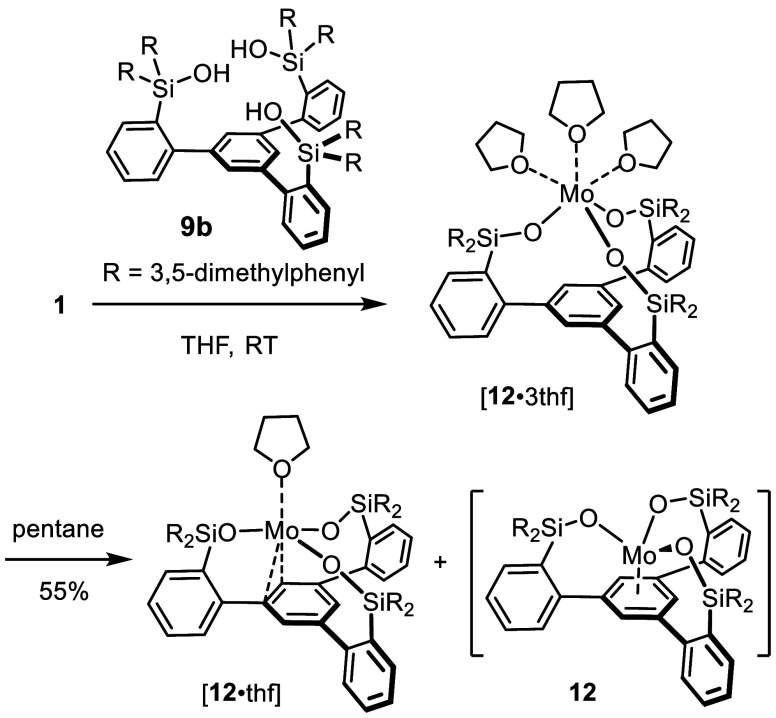
A New Monomeric Mo(+3) Complex Supported by a Tripodal Silanolate Ligand Scaffold Exists in Three Distinctly Different Bonding Modes

Therefore, we turned our attention to ligand **9b** carrying sterically much more demanding 3,5-dimethylphenyl substituents on the lateral Si-atoms (Scheme 2). Addition of **9b** to a solution of **1** in THF furnished a dark red solution; upon concentration, the newly formed pale-orange product [**12**·3thf] crystallized out. This allowed the constitution to be unambiguously determined as the targeted Mo(+3) monomer supported by the tripodal silanolate scaffold and three ancillary THF ligands (Figure 2). With a distance of 4.08 Å between the Mo center and the centroid of the basal phenyl ring, the metal does evidently not interact with the π-system underneath.

This information was all the more relevant as further analytical data of [**12**·3thf] could not be obtained. All attempts at drying the crystals in vacuo were accompanied by a drastic color change from orange to dark red; the same is true when [**12**·3thf] was dissolved in pentane: a deep red solution was instantly formed, from which red single crystals could be grown, leaving a colorless supernatant behind.

**Figure 3:**
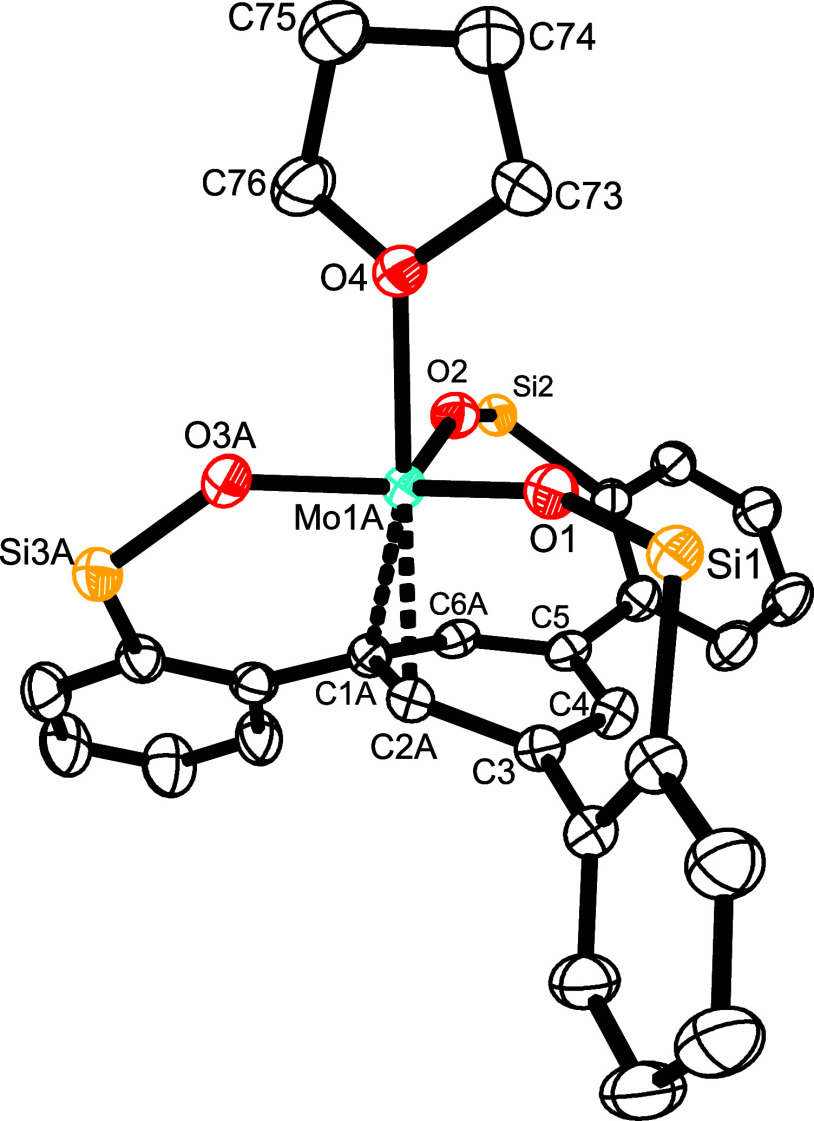
Truncated structure of complex [**12**·thf] in the solid state; the 3,5-dimethylphenyl substituents on the silicon linkers and H-atoms are removed for clarity; the full structure is contained in the Supporting Information. Selected bond lengths (Å) and angles (°): Mo1A-O1 1.9162(14), Mo1A-O2 1.9134(14), Mo1A-O3A 1.8979(18), Mo1A-O4 2.2524(15), Mo1A-C1A 2.276(2), Mo1A-C2A 2.224(2), C1A-C2A 1.466(3), C2A-3 1.453(3), C3–C4 1.351(3), C4–C5 1.438(3), C5–C6A 1.375(4), C6A-C1A 1.429(4), C1A-C2A–C3–C4 19.7.

While the elemental analysis of the crystals agreed with the mono-THF adduct [**12**·thf], single crystal X-ray diffraction revealed a more intricate composition, rendering the analysis particularly challenging. Optically, the crystals looked good, but the diffraction pattern was of medium quality. Refinement revealed several disorders that could explain the lower intensities at high diffraction angles. We interpreted the structure model as a multicomponent crystal with three different chemical species. The by far major species with a refined occupancy of ≈90% was the mono-THF adduct [**12**·thf] (Figure 3). The entirely THF-free complex **12** accounting for an occupancy of ≈3% could also be identified from the residual electron density. The third component (ca. 7%) could not be adequately modeled, although the bis-THF adduct [**12**·2thf] seems possible and arguably likely (for details, see the Supporting Information). It is important to mention that this analysis was repeated with 9 different crystals from various batches at temperatures between 100 and 253 K; each of them was shown to comprise the constituents outlined above with similar ratios (for details, see the Supporting Information).

The fact that THF is bound to the Mo(+3) center is notable since the parent Cummins complex **1** had been explicitly classified as being non-Lewis acidic, incapable of ligating THF, pyridine or PEt_3_.^21^ The notion that silanolates gently upregulate the acidity of the central metal they are attached to had already surfaced in our previous studies.^40,42^ However, the THF ligands in [**12**·3thf] can only be weakly bound. Loss of two of them entails ligation of the basal phenyl moiety to the Mo-center, which is displaced from the central axis of the aryl ring underneath. Along comes a notable distortion of the aromatic π-system (Figure 3); this fact is manifested in the notable kink of the formerly planar ring toward the metal atom, resulting in fairly short Mo1A–C1A (2.276(2) Å) and Mo1A–C2A (2.224(2) Å) distances. These structural attributes indicate significant electron donation from the metal center into the antibonding π* orbital, corresponding to an η^2^-bonding mode of the arene.^49^ The ensuing partial loss of aromaticity of the basal plane illustrates the reactivity of the central metal atom.

**Scheme 3:**
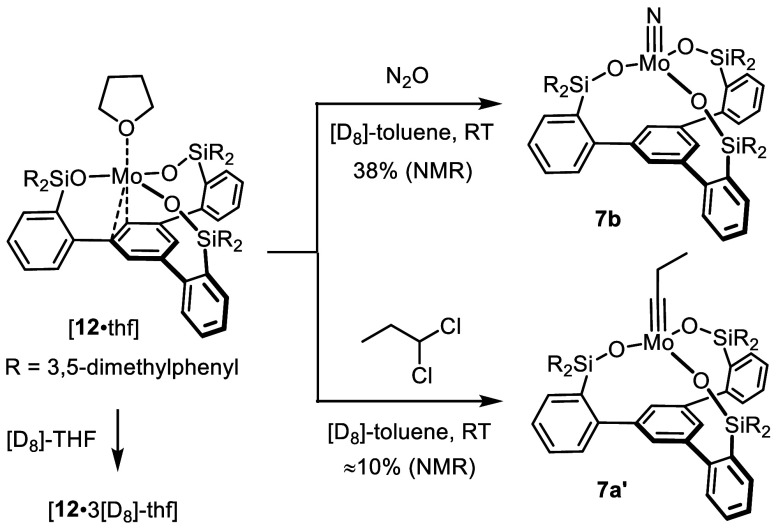
Reactions of [**12**·thf]

As one might expect, the loss of THF is reversible (Scheme 3). Thus, addition of excess [D_8_]-THF to a solution of [**12**·thf] in [D_8_]-toluene allowed the reformation of labeled [**12**·3thf] to be monitored by ^2^H NMR spectroscopy despite the paramagnetic nature of the complexes (for details, see the SI); moreover, crystal structure analysis confirmed the constitution of the resulting adduct. In line with the literature precedent on monomeric Mo(+3) species,^19^ [**12**·thf] reacted with N_2_O to give the nitrido complex **7b**, which was identified by comparison with an authentic sample;^44^ the expected nitrosyl complex, however, which should be concomitantly formed, has not been identified in the mixture. Likewise, treatment with 1,1-dichloropropane furnished the corresponding alkylidyne **7a′**;^23,24,27^ although the (unoptimized) yield was low, the very characteristic spectroscopic fingerprints, most notably the deshielded alkylidyne C atom (δ_C_ = 322.8 ppm), cannot be missed; the structure was fully assigned from the mixture (for details, see the Supporting Information).

**Figure 4:**
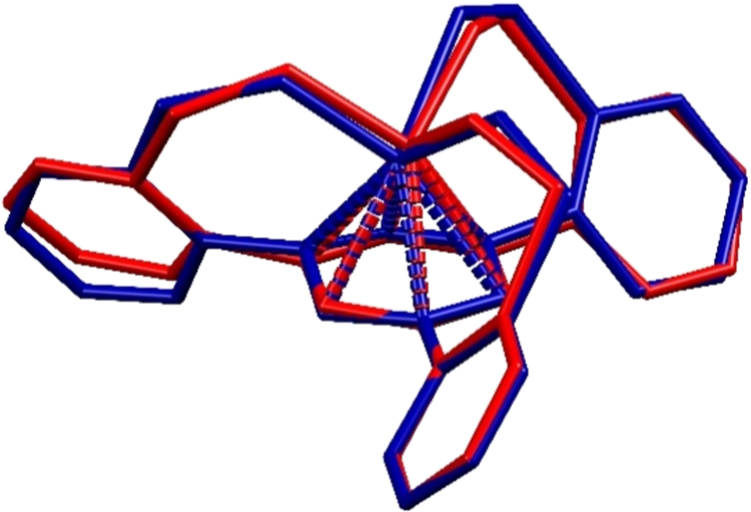
Superposition of the truncated experimental structure (blue) of the THF-free complex **12** in the solid state with the truncated calculated structure (red); the 3,5-dimethylphenyl substituents R on the Si-linkers were removed for clarity; for the full structure and further details, see the Supporting Information.

As mentioned above, [**12**·thf] is accompanied by the entirely THF-free species **12**. Although the latter is only a very minor constituent with ≈3% occupancy in the unit cell, the diffraction data allowed its constitution to be deduced, although the atomic positions could not be determined very accurately and a detailed discussion is hence not possible. A comparison with the computed structure shows good agreement, thus confirming the tentative assignment (Figure 4). In any case, the Mo-atom is located on a 3-fold axis; it resides only 1.726 Å above the centroid of the basal phenyl group, which is almost planar. Therefore, an η^6^-interaction between the Mo(+3) center and the arene ring seems to be the most appropriate description of the bonding situation.^49^ The situation is reminiscent of what has been observed with the tris-thiolate complex **5**.^35^

Comparison of the structures displayed in Figures 2–4 showcases yet another remarkable attribute of the tripodal ligand architecture. In [**12**·3thf], the Mo-atom resides 1.05 Å *above* the plane defined by the three O atoms, in [**12**·thf] it lies roughly *in* this plane, whereas in **12** it comes to rest recognizably *below* the plane. The amazing floppiness of the silanol linkages is also manifested in the evidently adaptable bond lengths; thus, the average Mo–O distances vary considerably (2.034 Å in [**12**·3thf]; 1.909(6) Å in [**12**·thf]; 2.156 Å (computed) in **12**). For these attributes, this type of ligand is broadly applicable: it can adapt to the needs of a single metal in different oxidation states, coordination environments and/or binding modes as shown herein, but can also bind to many different central metals of largely variable radii. A more comprehensive study sampling the periodic table is underway and will be published in due course.^50^

### Homodimerization and Discovery of an Entirely New Type of Heterodimers

**Figure 5:**
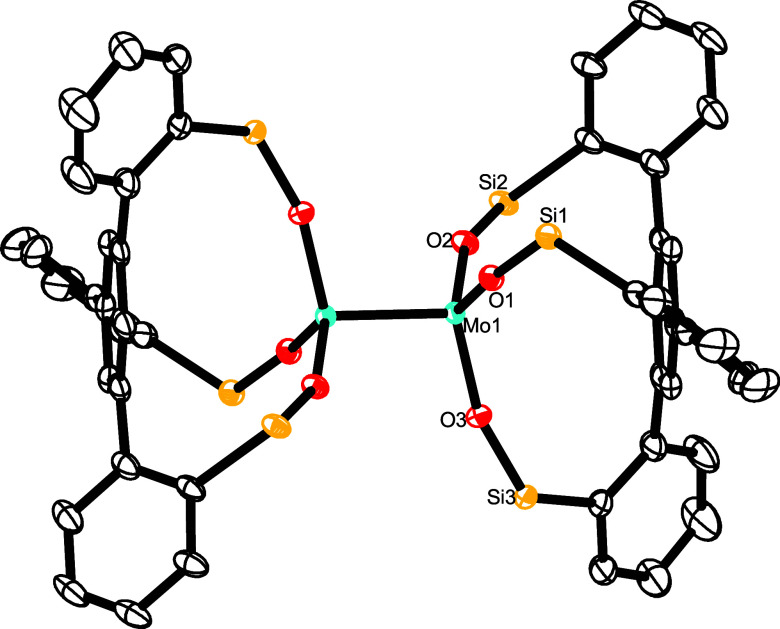
Truncated structure of the homodimeric complex **13** in the solid state; the 3,5-dimethylphenyl substituents on the silicon linkers and H-atoms are removed for clarity; the full structure is contained in the Supporting Information. Selected bond lengths (Å) and angles (°): Mo1–Mo1′ 2.2873(3), Mo1–O1 1.9107(12), Mo1–O2 1.9043(17), Mo1–O3 1.9148(12), Si1–O1–Mo1 158.67(8), O1–Mo1–Mo1′ 103.23(4).

**Scheme 4:**
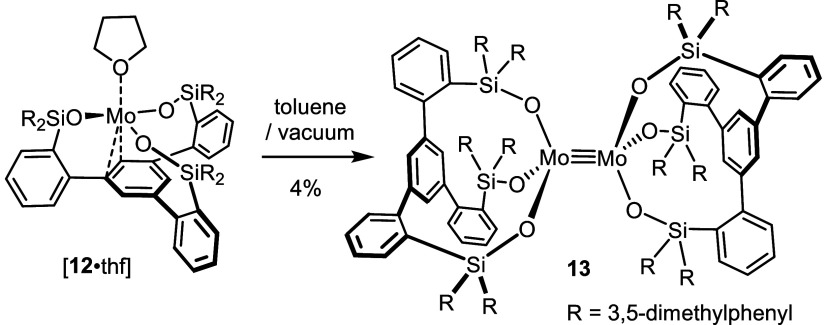
Formation of an Overcrowded Homodimer

Attempts were made at stripping the remaining THF off in order to obtain ligand-free **12** in pure form. To this end, [**12**·thf] was dissolved in toluene and all volatile materials were evaporated in high vacuum; this procedure was repeated three times. When a solution of the residue in toluene was then kept at ambient temperature, the slow formation of large orange single crystals was observed, which proved highly insoluble in all common inert solvents. Therefore, analysis was essentially limited to HRMS, combustion data, and single crystal X-ray diffraction. Much to our surprise, the complex turned out not to be the expected THF-free monomer **12** but rather the corresponding homodimer **13** (Scheme 4), akin to homodimer **11** derived from alkylidyne **7a** (R = Me) that had previously only been inferred from the analytical and spectral data (Scheme 1).^47^ The 3,5-dimethylphenyl substituents on the Si-atoms of **13** are innately interlocked, thus rendering the core region exceptionally crowded; the exceptionally long Mo≡Mo bond (2.2873(3) Å) is likely a derivative thereof (Figure 5). This truly encumbered situation may also explain why **13** was obtained in only very low yield.^51^

**Scheme 5:**
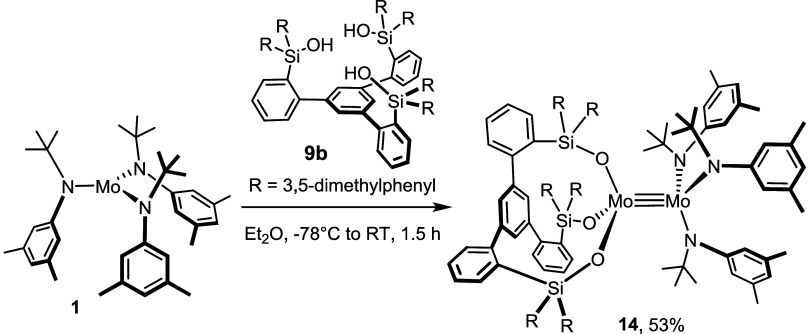
A Heterodimeric Complex Comprising an Intact “Cummins Complex” Entity as One of the Constituents

We conjectured that the use of a less good donor solvent could pave an alternative way to THF-free **12**. To this end, the trisilanol ligand **9b** was added to a suspension of **1** in Et_2_O at −78 °C and the resulting mixture was slowly warmed to ambient temperature. Surprisingly, NMR inspection of the crude material suggested that ca. 50% of the ligand remained unchanged even though **1** was fully consumed. To confirm this paradoxical result, the experiment was repeated with a 1:2 ratio of the reaction partners, which allowed the new complex **14** to be isolated in analytically pure form in 53% yield (Scheme 5). Its diamagnetic nature suggested that a Mo≡Mo bond might have been formed. ROESY cross peaks showed that two chemically different ligand spheres are present as part of one and the same molecule. The recorded ^15^N NMR shift (δ_N_ = −129 ppm) differs significantly from that of the free (*t*Bu)(Ar)NH (δ_N_ = −289 ppm), thus indicating a metal-bound amide ligand. Overall, the complex has *C*_3_ symmetry about the Mo≡Mo axis on the NMR time scale, since only one set of signals was observed for each of the ligands. In contrast, high barriers to rotation about the N–Ar as well as the Si–R bonds render the H-atoms on the aryl rings diastereotopic.

**Figure 6:**
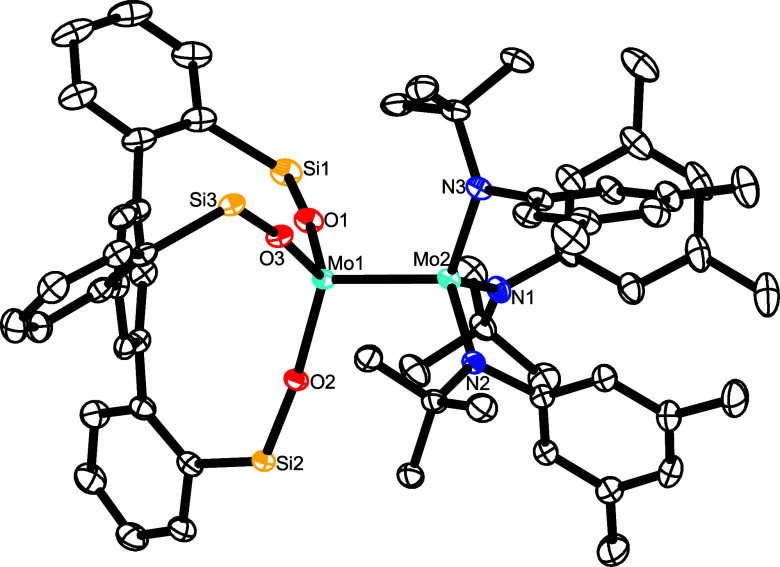
Truncated molecular structure of complex **14** in the solid state; the 3,5-dimethylphenyl substituents on the silicon linkers, disordered parts, and H-atoms are not shown for clarity; the full structure is contained in the Supporting Information. Selected bond lengths (Å) and angles (°): Mo1–Mo2 2.3440(3); Mo1–O1 1.9305(15), Mo1–O2 1.9455(14), Mo1–O3 1.9409(14), Mo2–N1 1.9885(18), Mo2–N2 1.9810(18), Mo2–N3 1.9954(18), O1–Mo1–Mo2 105.10(5), N3–Mo2–Mo1 110.15(5), Si1–O1–Mo1 173.36(11).

The structure of **14** in the solid state confirmed the conclusions drawn from the NMR data. To the best of our knowledge, this complex is the first example of a heterodimeric species of the type [X_3_Mo≡MoY_3_] (Figure 6). Truly perplexing is the fact that **14** incorporates an intact “Cummins fragment” as one of its constituents, even though complex **1** is famous for *not* forming metal–metal triple bonding otherwise. With 2.3440(3) Å, the Mo≡Mo bond of **14** is by far the longest triple bond between two unbridged Mo-centers with CN = 4 each (CN = coordination number) documented in the literature (see below);^52^ it is even longer than that of the overcrowded homodimer **13**. Despite this striking attribute, which one could (mis)take for a sign of weak bonding, the monomeric complex **12** (with or without additional Et_2_O ligands) has neither been observed nor isolated; heterodimerization must hence be fast and facile. Along the same lines, we note that **14**, once formed, is thermally and chemically robust; no indications for dissociation into the constituent monomeric units or for “scrambling” into the corresponding homodimer **13** and free **1** were noticed on prolonged warming of a solution in toluene or on treatment with two-electron donor ligands.

**Figure 7:**
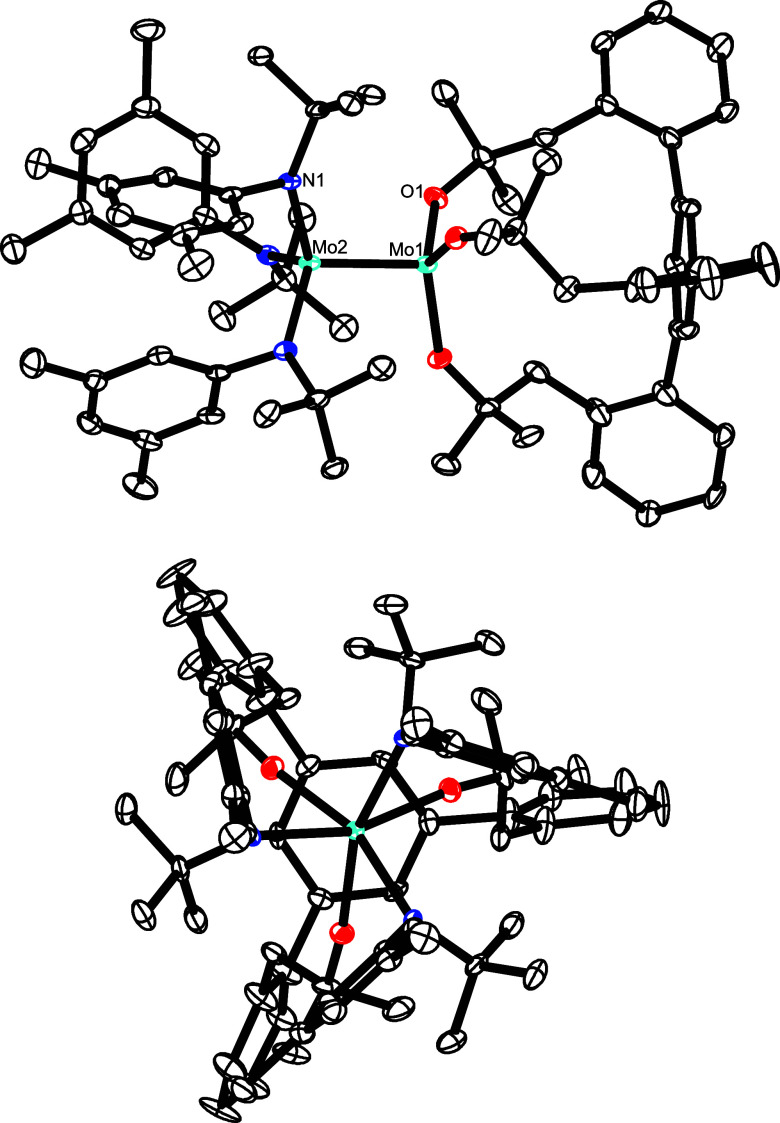
Molecular structure of the heterodimeric complex **16** in the solid state; top: side view; bottom: projection along the 3-fold crystallographic Mo–Mo axis; H-atoms, disordered parts, and solute solvent molecules in the unit cell not shown for clarity; the full structure is contained in the Supporting Information. Selected bond lengths (Å) and angles (°): Mo1–Mo2 2.2955(7), Mo1–O1 1.915(7), Mo2–N1 1.998(2), C1–O1–Mo1 144.7(7), O1–Mo1–Mo2 100.44(7), N1–Mo2–Mo1 106.29(8).

**Scheme 6:**
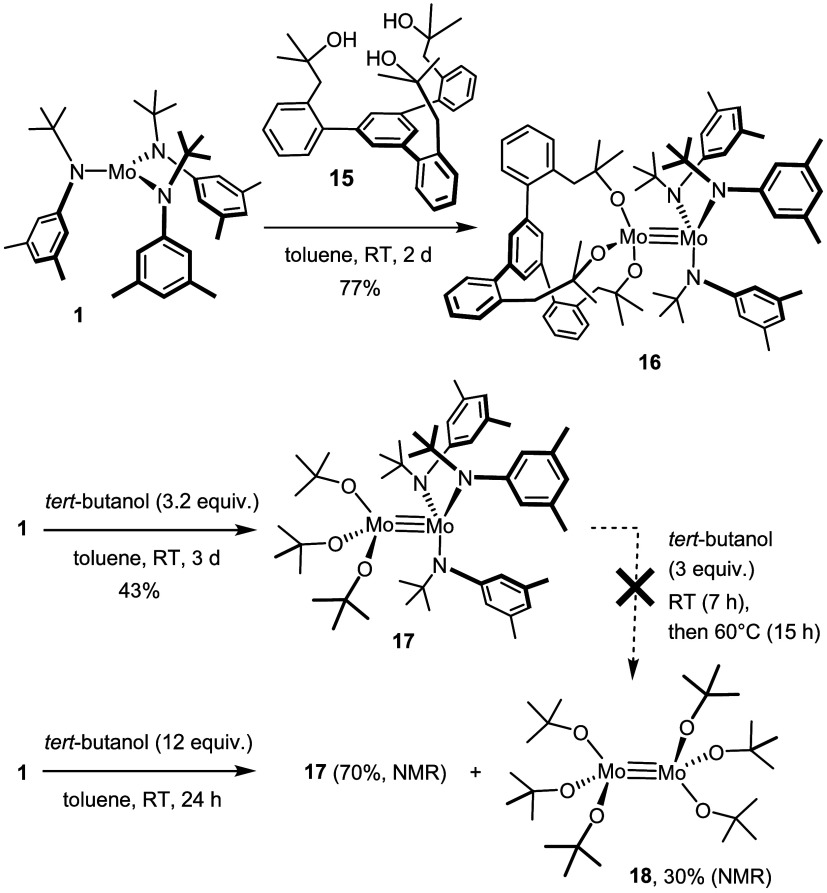
Heterodimers by Alcoholysis of **1**

Next, it was probed whether the formation of **14** is due to the peculiar trisilanolate ligand framework. To this end, we first resorted to the tripodal alcohol derivative **15**, which had originally been designed to bolster the activity of tungsten alkylidyne catalysts of type **8**.^42^ This particular ligand is similar to **9** in geometric terms, but its −OH groups are less acidic than the corresponding silanols in **9**; once bound to the metal center, alkoxides are notably better π-donor ligands than siloxides. These nuances apart, addition of **1** to a solution of **15** cleanly furnished the corresponding heterodimeric complex **16**; in this case, the best results were obtained in toluene as the solvent, which allowed **16** to be isolated in analytically pure form in 77% yield (Scheme 6). The recorded NMR data were analogous to those of **14**; the diagnostic ^15^N NMR shift (δ_N_ = −147 ppm) was taken as a clear sign for the incorporation of an intact “Cummins fragment” into the newly formed Mo≡Mo bond. Variable temperature NMR spectra showed high rotational barriers about the N–Ar bonds of the amido ligand; one of the aromatic protons evidently resides in the anisotropy cone of a neighboring aryl ring and is therefore notably shielded (δ_H_ = 4.19 ppm).^53^ The constitution of **16** as a heterodimeric entity was ascertained by crystal structure analysis (Figure 7). With 2.2955(7) Å, the Mo≡Mo distance is slightly shorter than that of **14** (2.3440(3) Å). Another interesting structural feature is visible in the Newman-type projection, which shows that the ligands are not perfectly staggered about the Mo–Mo axis, despite their bulk; the smallest torsion (O1–Mo1–Mo2–N1) is only 39.6° (rather than 60°).

**Figure 8:**
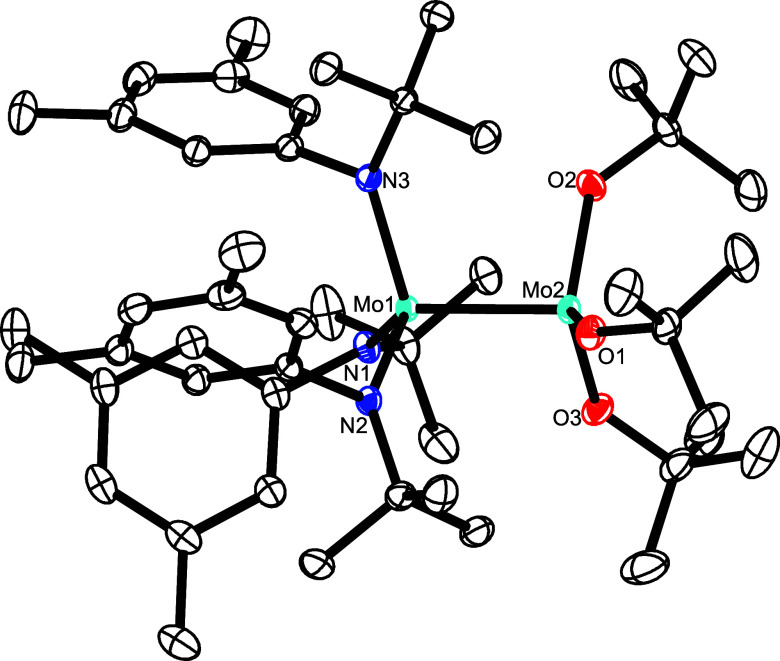
Molecular structure of complex **17** in the solid state; H-atoms and disordered solvent in the unit cell removed for clarity. Selected bond lengths (Å) and angles (°): Mo1–Mo2 2.2944(2), Mo1–N1 1.9893(14), Mo1–N2 1.9910(14), Mo1–N3 2.0010(14), Mo2–O1 1.9147(12), Mo2–O2 1.9122(12), Mo2–O3 1.9175(13), Mo1–Mo2–O1 99.49(4), Mo2–Mo1–N1 106.72(4).

The astounding bias for heterodimerization manifested in these examples is not caused by the peculiar tripodal ligand framework of **9** and **15**, in which the three arms carrying the protic substituents are tied back onto a rigidifying arene linker. This became clear when **1** was reacted with ordinary *tert*-butanol (Scheme 6). Once again, the corresponding heterodimer **17** was formed in respectable yield. It shows the same distinctive structural attributes, namely a very long Mo≡Mo bond (2.2944(2) Å) as well as small torsional angles (O–Mo–Mo–N: 32.57, 33.35, 34.72°) even further away from the ideal 60° of a staggered “ethane-like” conformer that one might intuitively expect to be optimal for such a crowded ligand environment (Figure 8). It is likely that these peculiar structural features as well as the striking stability of such overcrowded dimers are due, at least in part, to attractive forces such as “interligand” London dispersion interactions.^54−56^

Other structural parameters of the heterodimers are also best discussed with complex **17** as it lacks any distortion by a caged ligand framework. With an average Mo–Mo–O bond angle of 100.3°, the coordination geometry about Mo2 is halfway between planar and tetrahedral, which may indicate that the bending back of the ligands is more likely caused by ligand–ligand repulsion rather than rehybridization. The fact that the Mo–Mo–N angles are slightly larger (average: 106.1°) does not discount the argument, as the sterically more demanding −NAr(*t*Bu) groups must tilt further away. The Mo2–O*t*Bu distances in **17** (average: 1.9148 Å) are comparable to those in [(RO)_3_Mo≡Mo(OR)_3_] (R = CMe_2_Ph; average: 1.892 Å)^57^ and in the alkylidyne ArC≡Mo(O*t*Bu)_3_ (average: 1.884 Å; Ar = 2,6-dimethylphenyl);^58^ as expected, they are shorter than the Mo–OSi bonds in complex **14** (average: 1.939 Å), which confirms that alkoxides are better π-donors than the corresponding silanolates.^40,59,60^ The Mo–N bonds in **17** (average: 1.994 Å) are longer than those in the parent Cummins complex **1** (1.967 Å);^19^ the barrier to rotation about the N–Ar bond was determined by NMR to be on the order of 12.5 kcal·mol^–1^ (for details, see the Supporting Information).

Perplexed by the ease with which these unorthodox heterodimers are formed, the alcoholysis of **1** was repeated with *tert*-butanol in excess. Although a mixture was obtained in this case, the heterodimer **17** was still the major species in the crude material, whereas the homoleptic dimer [Mo_2_(O*t*Bu)_6_] (**18**) was minor (Scheme 6). Similarly instructive was another control experiment, in which purified **17** was treated with *tert*-butanol; the recorded spectra showed no sign of formation of **18** even when the temperature was raised and the solution stirred at 60 °C for 15 h.^61^ This result is striking if one considers that the alcoholysis of [Mo_2_(NMe_2_)_6_] (**19**) with *tert*-butanol in a hydrocarbon solvent is the standard method for the preparation of **18**.^62^ Moreover, **18** is the final product of and thermodynamic sink in many different reactions starting from various molybdenum sources.^5,12,63−67^ The remarkable preference for the formation of the heterodimer **17** is hence most likely kinetic in origin: putative [(*t*BuO)_3_Mo] (or a mixed species [(*t*BuO)_*n*_[(*t*Bu)(Ar)N]_*m*_Mo], *n* + *m* = 3) *in statu nascendi* is evidently so “hot” that it instantly traps any remaining **1** with formation of **17**.^68^ Once formed, the crowded ligand sphere precludes protonolysis of the remaining Mo–N bonds, at least by an encumbered reaction partner such as *tert*-butanol.

**Scheme 7:**
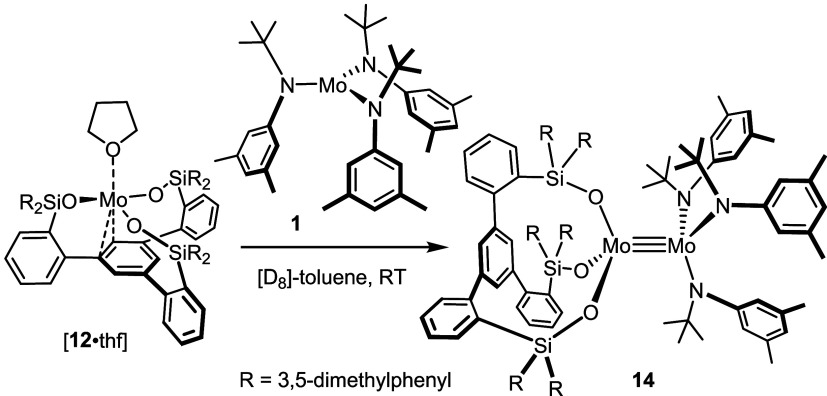
Cross-Dimerization of Two Molecularly Defined Mo(+3) Complexes

The notion that the Cummins complex **1** can be intercepted by a (transient) monomeric [MoX_3_] entity was experimentally confirmed. Thus, a 1:1 mixture of [**12**·thf] and **1** in toluene cleanly afforded the corresponding heterodimer **14** as proven by NMR spectroscopy (Scheme 7).

### ^95^Mo NMR Data and Computational Analysis

A brief comment on the ^95^Mo NMR shifts of the heterodimeric complexes is warranted. Because this molybdenum isotope is a spin 5/2 nucleus with low natural abundance (≈15.9%), an unfavorable gyromagnetic ratio and a low quadrupole moment, ^95^Mo NMR is not without challenges.^69−71^ Early work, however, had shown that the nuclei in dimeric Mo≡Mo complexes are strongly deshielded and can therefore be easily distinguished from other molybdenum species.^72^ Although the problems caused by the quadrupolar nature of ^95^Mo are massively enhanced by lowering symmetry and increasing the molecular weight of the compounds, we managed to record *two* distinct ^95^Mo resonances for the new heterodimeric complex **17**: the sharper signal at 3260 ppm is tentatively attributed to the [≡Mo(O*t*Bu)_3_] unit, whereas a much broader signal at 3127 ppm likely stems from the [≡Mo[N(Ar)(*t*Bu)]_3_] entity.^73^ For complex **14**, however, only one very broad peak at 3314 ppm was detected even after 5 d of acquisition time.

**Figure 9:**
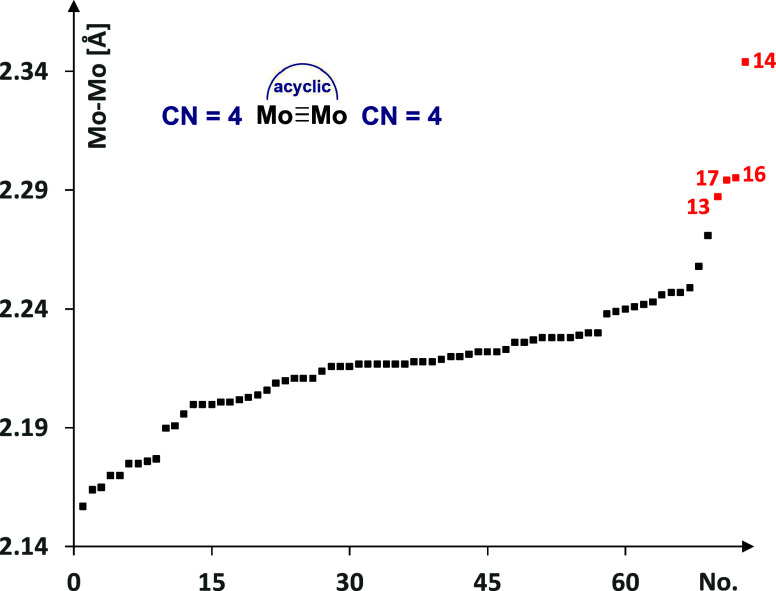
Plot of the Mo–Mo bond distances of complexes comprising an unbridged Mo≡Mo core with a CN = 4 on both metal atoms found in the Cambridge Crystallographic Data Centre (black dots); comparison with the new heterodimeric complexes reported herein (for details including structures and accession codes of the literature-known complexes, see the Supporting Information).

Although the data set is currently small, some aspects are noteworthy (Table 1). The heterodimers **14** and **17** are massively deshielded (≈600–800 ppm) compared to homodimeric siblings carrying the same or similar alkoxide, siloxide or amido ligands (**11**, **18**, **19**); at the same time, they show much longer Mo–Mo distances compared to these (and all other) homodimers bearing heteroatom ligands, for which metric data are available (Figure 9). Therefore, one might speculate that both properties are manifestations of the peculiar electronic character of their polarized Mo≡Mo core. A look at the homoleptic complex [R_3_Mo≡MoR_3_] (**20**, R = CH_2_Me_3_) devoid of any π-donating ligands, however, teaches that a simple correlation between shift and bond length does not exist: **20** resonates at δ_Mo_ = 3695 ppm,^72^ yet shows an extremely short Mo≡Mo bond (2.1654(7) Å).^76^

**Table 1:**
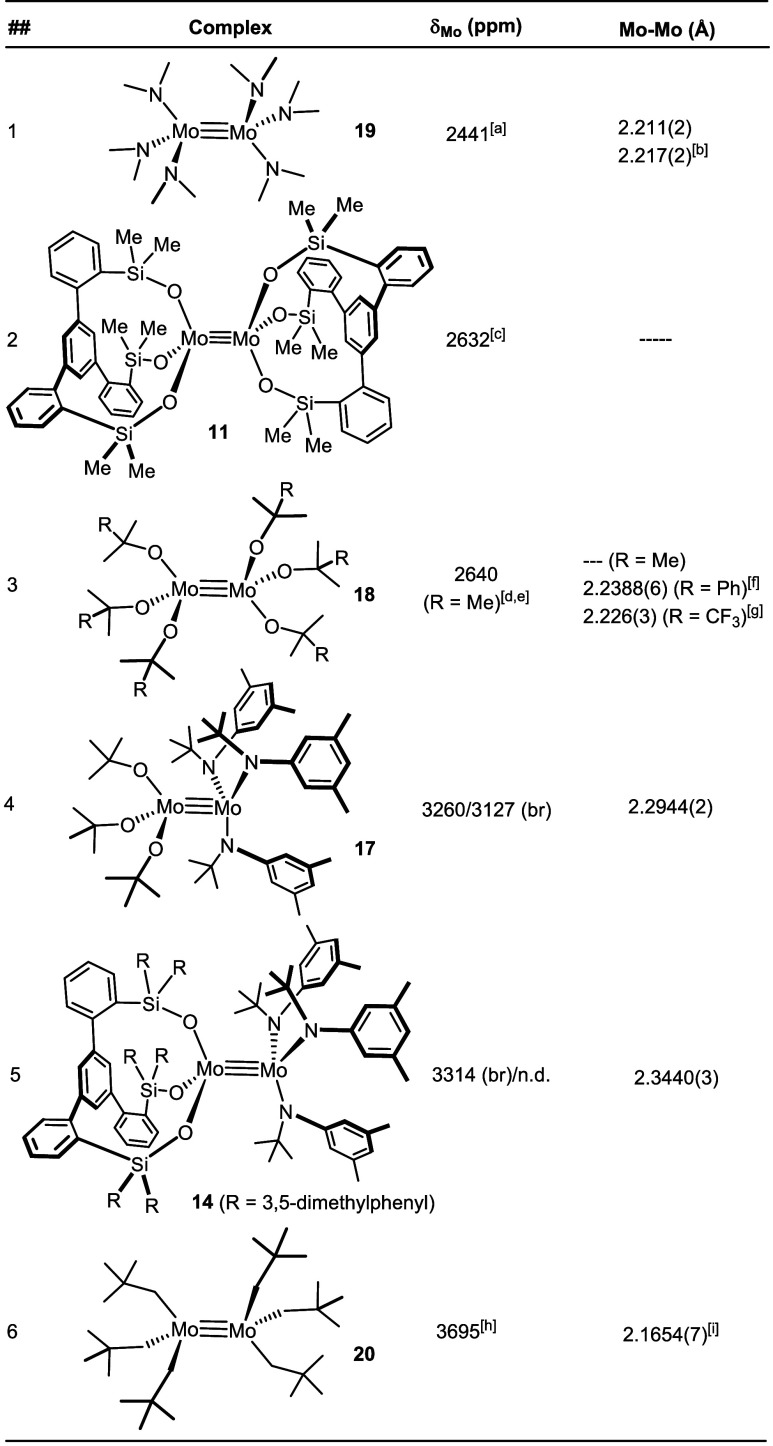
^95^Mo NMR Shifts ([D_8_]-Toluene, 333 K (unless stated otherwise)) and Mo–Mo Distances of Selected Dimeric Complexes

a Ref (72): 2430 ppm (C_6_D_6_, RT).

b Two independent complexes in the asymmetric unit cell, ref (74).

c Ref (47).

d Ref (67).

e Ref (72): 2645 ppm (C_6_D_6_, RT).

f Ref (57).

g Ref (75).

h Ref (72).

i Ref (76).

**Figure 10:**
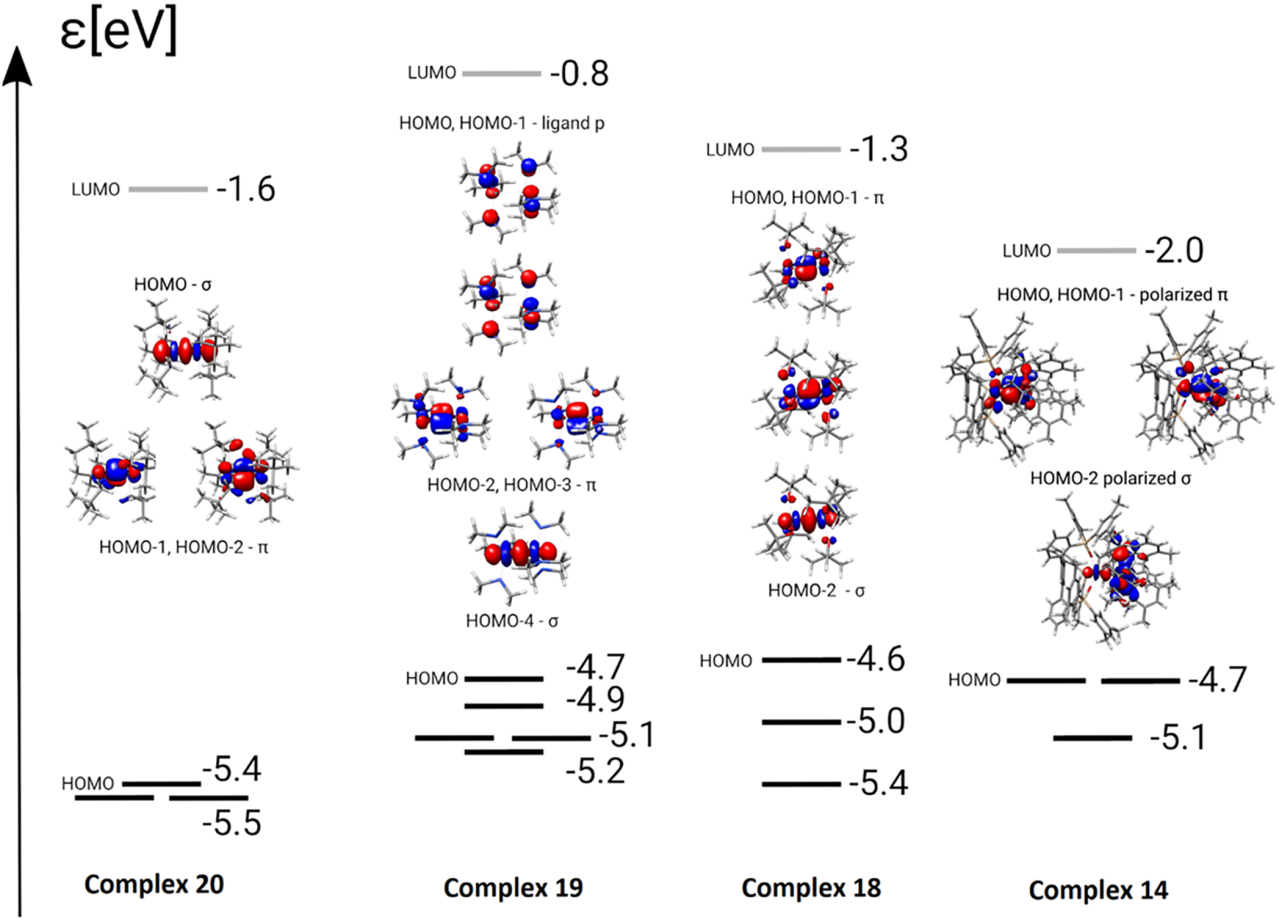
Comparison of the MO schemes of complexes **20**, **19**, **18** and the new heterodimeric complex **14**; for full MO plots and further details, see the SI.

As these experimental data are ostensibly difficult to reconcile, we resorted to a computational analysis. The computed ^95^Mo NMR shieldings/shifts (Figure 10) match the recorded values well, thus corroborating the validity of the chosen level of theory (TPSSH/def2-TZVPP (SARC-ZORA-TZVPP for Mo) and CPCM(toluene) with structures optimized at the B3LYP/def2-TZVP D3BJ CPCM(toluene) level of theory). In line with what the data of complex **20** had suggested, a meaningful correlation between any of the paramagnetic components of the shielding tensors and the [Mo≡Mo] bond distances has not been found.

A more detailed orbital analysis provided some insights into why this is the case. Qualitatively, the bonding in [X_3_Mo≡MoX_3_] complexes is adequately described by assuming that the d_*z*^2^_ and the d_*xz*_ and d_*yz*_ orbitals of the individual metal atoms form one σ- and two π-bonds, which constitute the HOMO–2, HOMO–1 and the HOMO, respectively.^2^ Interestingly, however, it turns out that this rather intuitive interpretation is valid only for X = OR, that is for complexes of type **18** (Figure 10). In case of complex **19** endowed with amido ligands, the p-orbitals of the N-lone pairs lie in the HOMO–LUMO region; although they do not contribute to Mo–Mo binding, they affect the ^95^Mo NMR shifts. Actually, the orbitals forming the Mo≡Mo triple bond of **19** are HOMO–2, HOMO–3 and HOMO–4. Although formed by entirely different orbitals, the energies of the HOMOs of **18** and **19** are similar.

It is striking that the ordering of the σ- and the π-orbitals changes completely in the neopentyl complex **20** devoid of heteroatoms in the ligand sphere (entry 6):^77^ it is the σ-orbital derived from the d_*z*^2^_ orbitals of the individual Mo atoms which constitutes the HOMO rather than the HOMO–2, as one might expect and as has indeed been found in case of **18**; in energetic terms, it is significantly lower-lying than the HOMOs of **18** and **19**. These three examples suffice to demonstrate that one has to beware of generalizations even when analyzing the bonding situation in simple homodimeric and homoleptic [X_3_Mo≡MoX_3_] complexes: the HOMO of **18** has π-symmetry, that of **20** has σ-character, whereas the HOMO of **19** is largely centered on the heteroatoms of the ancillary amido ligands.

Therefore, it is unsurprising that heterodimer **14** is yet another special case. All frontier orbitals are strongly polarized toward the side of the Mo atom carrying the −NR_2_ ligands; the *average* energy of the three frontier orbitals is about −4.8 eV, which is higher than that of the homodimers (ca. −5.5 eV (**20**), −5.1 eV (**19**), −5.0 eV (**18**)) (obtained at the TPSS level of theory, see the Supporting Information for details). While this trend suggests that stretching of the [Mo≡Mo] bond comes along with the expected increase in the energies of the orbitals forming the Mo–Mo σ- and π-bonds, no simple correlation between this trend and the ^95^Mo NMR chemical shifts does emerge; the relationship is obviously more complex. An in depth analysis seems warranted once more experimental shift data is available.

## Conclusions

Although tripodal silanols of type **9** have originally been designed to foster the catalytic activity and performance of high-valent Mo(+6)-alkylidyne complexes, they proved equally versatile in the realm of Mo(+3) chemistry; they are the first and, so far, only ligands capable of supporting highly reactive monomeric [MoX_3_] complexes, the corresponding homodimers, as well as a previously unknown type of heterodimers. Specifically, the resulting mononuclear trigonal-planar Mo(+3) complexes are rendered kinetically stable if the substituents on the peripheral Si-linkers of the tripodal ligand framework are sufficiently encumbered, even though such d^3^ electron species are exceptionally reactive and hence exceedingly rare otherwise. For the peculiar ligand architecture, the Mo(+3) complex resulting from ligand **9b** was shown to exist in three distinctly different binding modes: it can form an adduct with three, one or no THF ligand. Along comes a massive change in the interaction with the basal plane of the ligand framework itself, which is either not ligated at all, η^2^-bound, or forced to bind in an η^6^-coordination mode, respectively. The adaptive character manifested in this data is expected to be enabling in other context too.

Homodimerization of the monomeric complexes is possible but kinetically handicapped. In striking contrast, they can intercept unreacted Cummins complex **1** with exceptional ease even though the latter is famous for not engaging in metal–metal bonding otherwise. The resulting unsymmetrical heterodimers are the first examples with a ligand pattern of the general type [X_3_Mo≡MoY_3_]. Their metal–metal bond is polarized and exceptionally long, though chemically and thermally rather stable. Moreover, this new type of heterodimerization is not limited to tripodal silanolates as the ligands but is clearly a more general transformation. The novel class of heterodimeric [X_3_Mo≡MoY_3_] complexes described herein shows strongly deshielded ^95^Mo NMR signals; however, a simple correlation between the chemical shift and the bond length does not exist because ligand-based orbitals also affect the shielding tensor to a significant extent.

## Supporting Information Available

Experimental Section including characterization data and NMR spectra of new compounds (PDF)
Crystallographic Data and Discussion Computational Details (PDF)
Computational Study (PDF)
