# Supplementary material for: Tripodal Silanolate Ligands Expand [MoX3] Chemistry Beyond Its Traditional Borders
Source: J Am Chem Soc. 2025 Apr 11;147(16):13871–84. doi: 10.1021/jacs.5c02178 (PMC12022994; doi:10.1021/jacs.5c02178)
Supplement: Supplementary file 1 — ja5c02178_si_001.pdf [file ja5c02178_si_001.pdf]

SUPPORTING INFORMATION

Tripodal Silanolate Ligands Expand [MoX_3] Chemistry Beyond Its Traditional Borders

Daniel Rütter, Nils Nöthling, Markus Leutzsch, Alexander A. Auer, Alois Fürstner*

Max-Planck-Institut für Kohlenforschung, 45470 Mülheim/Ruhr, Germany

Email: fuerstner@kofo.mpg.de

Table of Contents

General S2
Experimental Data S4
Bis(3,5-dimethylphenyl)dimethoxysilane (**S1**) S4
Ligand **9b** S5
Complex [**12**·3thf] S6
Complex [**12**·thf] S6
Reversible Loss and Binding of THF S7
Reaction of Complex [**12**·thf] with N_2O S9
Reaction of Complex [**12**·thf] with 1,1-Dichloropropane S10
Heterodimer Formation by Reaction of Complex [**12**·thf] with Complex **1** S14
Complex **13** S15
Complex **14** S16
Complex **16** S17
Complex **17** S19
NMR Spectra S20
References S46

GENERAL

Manipulations of air and hydrolysis sensitive substances were carried out using standard Schlenk techniques. Unless stated otherwise, all reactions were carried out in glassware which had been heated in high vacuum with a Bunsen burner, cooled to ambient temperature and filled with argon prior to use. Likewise, all cannulas and syringes were carefully flushed with argon before use.

The solvents used were distilled after drying over the indicated reagents and stored under an argon atmosphere over molecular sieves (3 Å): tetrahydrofuran (magnesium/anthracene), benzene (CaH_2), dichloromethane (CaH_2), diethyl ether (Na/K), toluene (sodium tetraethylaluminium), *n*-pentane (Na/K). Hexamethyldisiloxane (HMDSO), [D_6]-benzene and [D_8]-toluene were degassed by three freeze-pump-thaw cycles and then stored over molecular sieves (3 Å). Molecular sieves were activated at 180 °C for 3 d under high vacuum (10^{-3} mbar) and stored under argon.

Commercially available chemicals were used without further purification unless otherwise noted. The following compounds were prepared according to the literature: Mo[N(*t*Bu)(Ar)]$_3$ (Ar = 3,5-dimethylphenyl) (**1**),[1-4] ligands **9a**,**b**, **15**.[5-7]

All ligands used were dried by the following method: The ligand was dissolved in toluene and then powdered molecular sieves (4 Å) were added. The resulting suspension was stirred overnight, filtered *via* cannula and all volatile components were removed *in vacuo* (10^{-3} mbar).

Elemental analysis were performed by Mikroanalytisches Laboratorium H. Kolbe, Oberhausen, Germany.

Nuclear magnetic resonance spectroscopy (NMR)

Spectra were recorded on Bruker Avance III HD 400 MHz or Avance Neo 600 MHz NMR spectrometers in the indicated solvents; chemical shifts (δ) are given in ppm relative to tetramethylsilane (TMS), coupling constants (*J*) in Hz. The multiplicities of the signals are described by the following abbreviations: s: singlet, d: doublet, t: triplet, q: quartet, pent: quintet, sept: septet, m: multiplet, bs: broad signal. Solvent signals were used as reference and chemical shifts were converted to the TMS scale ([D_6]-benzene: δ_C = 128.06 ppm, residual ^{1}H: δ_H = 7.16 ppm; CD_2Cl_2: δ_C = 53.84 ppm, residual ^{1}H: δ_H = 5.32 ppm; [D_8]-toluene: δ_C = 20.43 ppm, residual ^{1}H: δ_H = 2.09 ppm). 1D ^{29}Si NMR spectra were acquired with a polarisation transfer pulse sequence (refocussed INEPT) and broadband proton decoupling. ^{95}Mo NMR spectra were acquired using the Bruker aring pulse sequence to minimize acoustic ringing from the NMR probe at 333 K unless noted otherwise. The π/2 ^{95}Mo pulse was calibrated with a Na_2MoO_4 (2 M in D_2O) sample and had a typical length of 22.5 μs. ^{15}N NMR shifts were extracted from cross peaks in a ^{1}H-^{15}N-HMBC experiments. ^{29}Si and ^{95}Mo chemical shifts were referenced indirectly to the ^{1}H chemical shift of the solvent according to IUPAC recommendations using

the *xiref* macro in Bruker Topspin.[8] ^{15}N chemical shifts are reported relative to CH_3NO_2 (δ = 0 ppm; Ξ = 10.136767%), ^{29}Si chemical shifts are reported relative to Me_4Si (δ = 0 ppm; Ξ = 19.867187%) and ^{95}Mo chemical shifts relative to Na_2MO_4 (δ = 0 ppm; Ξ = 6.516926%).

Infrared spectroscopy (IR)

IR spectra were measured on a Spectrum One (Perkin-Elmer) spectrometer at room temperature. Absorption bands are reported in wavenumbers (cm^{-1}); in case of the metal complexes, inert conditions were secured (glove box).

Mass spectrometry (MS)

High-resolution mass spectra (HRMS) were measured with a Finnigan MAT 95 spectrometer (EI) or a Bruker APEX III FT-ICR-MS (ESI). All values are given in mass units per elementary charge (*m/z*).

EXPERIMENTAL DATA

Bis(3,5-dimethylphenyl)dimethoxysilane (**S1**)

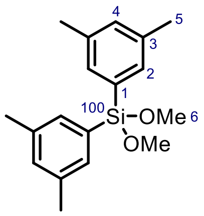

A solution of 5-bromo-*m*-xylene (15 mL, 0.11 mol) in THF (75 mL) was added dropwise over a period of 1 h to a suspension of Mg turnings (2.9 g, 0.12 mol) and LiCl (4.7 g, 0.11 mol) in THF (40 mL). After 5 min, heat evolution was observed. Stirring was continued for 30 min before the mixture was transferred *via* cannula into the dropping funnel used in the reaction described below.

This solution of (3,5-dimethylphenyl)magnesium bromide in THF was added dropwise over a period of 10 min to a solution of $Si(OMe)_4$ (7.5 mL, 51 mmol) in THF (135 mL) at 0 °C. Once the addition was complete, stirring was continued for 2 h at ambient temperature before water (150 mL) was carefully added to quench the reaction. The mixture was transferred into a separatory funnel and the aqueous phase extracted with EtOAc (3 x 100 mL). The combined organic layers were dried over $MgSO_4$, and all volatile components were removed *in vacuo* to give a colorless oil. The crude product was purified by distillation in high vacuum (10^{-3} mbar, bath temperature: 185 °C; b. p. 145-147 °C) to give the title compound as a colorless solid (7.6 g, 50%).

^{1}H NMR (600 MHz, CD_2Cl_2, 298 K): δ = 7.24−7.23 (m, 4H; H-2), 7.08−7.07 (m, 2H; H-4), 3.59 (s, 6H; H-6), 2.30 ppm (q, *J* = 0.6 Hz, 12H; H-5).

^{13}C NMR (151 MHz, CD_2Cl_2, 298 K): δ = 137.7 (C-3), 132.69 (C-1), 132.68 (C-2), 132.4 (C-4), 51.0 (C-6), 21.5 ppm (C-5).

^{29}Si NMR (119 MHz, CD_2Cl_2, 298 K): δ = −28.9 ppm.

IR (ATR): ṽ = 2934, 2857, 2834, 1594, 1455, 1404, 1379, 1273, 1182, 1139, 1072, 991, 938, 870, 851, 799, 720, 696, 569, 547, 532, 485, 447, 431, 417 cm^{-1}.

HRMS (EI^+): *m/z* calculated for $C_{18}H_{24}O_2Si$ $[M]^+$: 300.15401, found: 300.15412.

Ligand **9b**

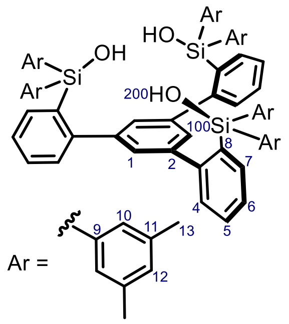

A solution of *tert*-butyllithium (1.7 M in *n*-pentane, 28 mL, 48 mmol) was added dropwise over a period of 5 min to a suspension of 1,3,5-tris-2'-bromophenylbenzene (4.3 g, 8.0 mmol)[6] in Et_2O (100 mL) at −110 °C. Stirring was continued for 1 h at ambient temperature, after which the reaction mixture was cooled again to −110 °C and a solution of compound **S1** (7.3 g, 24 mmol) in Et_2O (20 mL) was slowly added. Stirring was continued for 17 h at ambient temperature before the reaction was carefully quenched with water (50 mL). The aqueous phase was extracted with CH_2Cl_2 (3 x 100 mL) and the combined organic layers were dried over $MgSO_4$. All volatile components were removed *in vacuo* to give a light yellow foam (9.2 g), which was used in the next step without further purification.

Concentrated aq. HCl (60 mL) was added to a solution of this crude material (9.2 g) in THF (200 mL) at 0 °C in air. Stirring was continued for 2 h at ambient temperature before aq. NaOH (3 M, 250 mL) was carefully added. The aqueous phase was extracted with CH_2Cl_2 (3 x 100 mL) and the combined organic layers were dried over $MgSO_4$. All volatile components were removed *in vacuo* to give a light yellow crystalline solid, which was washed with CH_2Cl_2 (3 x 5 mL) and dried under high vacuum (10^{-3} mbar) to give the title compound as a white powder (7.5 g, 88% over two steps).

^{1}H NMR (600 MHz, CD_2Cl_2, 298 K): δ = 7.41−7.39 (m, 3H; H-7), 7.25−7.20 (m, 6H; H-5 and H-6), 7.13−7.12 (m, 12H; H-10), 7.07 (s, 3H; H-1), 6.91−6.90 (m, 6H; H-12), 6.84−6.83 (m, 3H; H-4), 3.96 (s, 3H; H-200), 2.12 ppm (s, 36H; H-13).

^{13}C NMR (151 MHz, CD_2Cl_2, 298 K): δ = 148.9 (C-3), 144.1 (C-2), 137.8 (C-7), 137.5 (C-11), 134.5 (C-8), 132.2 (C-10), 131.6 (C-12), 130.0 (C-5), 129.2 (C-4), 129.0 (C-1), 126.3 (C-6), 21.4 ppm (C-13).

^{29}Si NMR (119 MHz, CD_2Cl_2, 298 K): δ = –12.4 ppm.

IR (ATR): ṽ = 3415, 3013, 2916, 2857, 1584, 1557, 1469, 1437, 1402, 1376, 1269, 1168, 1138, 1088, 1064, 1037, 989, 939, 896, 860, 845, 800, 761, 739, 714, 696, 666, 652, 637, 622, 553, 537, 520, 491, 475, 411x cm^{-1}.

HRMS (ESI^{+}): *m/z* calculated for $C_{72}H_{72}NaO_3Si_3$ $[M+Na]^{+}$: 1091.46815, found: 1091.46797.

Complex [**12**·3thf]

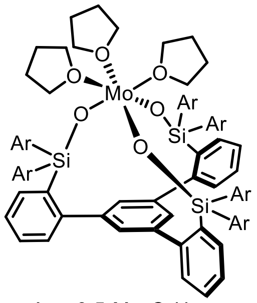

Ar = 3,5-$Me_2C_6H_3$

A dark red solution of complex **1** (312 mg, 0.499 mmol) in THF (27 mL) was added to a colorless solution of ligand **9b** (484 mg, 0.453 mmol) in THF (27 mL). After the addition was complete, the dark red solution was concentrated under high vacuum (10^{-3} mbar) to ca. 1/4 of its volume to give a dark red-brown suspension, which was filtered under Ar to give a first crop of complex [**12**·3thf] as the pale orange filter cake. The dark red-brown filtrate was stored for 1 d at −20°C, causing the precipitation of additional [**12**·3thf] as pale orange crystals. Complex [**12**·3thf] decomposes upon application of high vacuum; therefore no elemental analysis was obtained.

Complex [**12**·3thf] can be stored as a suspension in THF to avoid decomposition. Although it has a limited solubility in THF, diluted solutions can be prepared, from which single crystals suitable for X-ray diffraction were grown upon storage at −20°C.

Complex [**12**·thf]

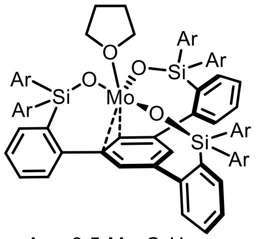

Ar = 3,5-$Me_2C_6H_3$

A dark red solution of complex **1** (312 mg, 0.499 mmol) in THF (27 mL) was added to a colorless solution of ligand **9b** (484 mg, 0.453 mmol) in THF (27 mL). The resulting dark red solution was concentrated under high vacuum (10^{-3} mbar) to ca. 1/4 of its volume to give a dark red-brown suspension, which was filtered to give complex [**12**·3thf] as the pale orange filter cake.

The pale orange filter cake was dissolved in *n*-pentane (9 mL) to form a deep red solution, which was vigorously stirred for a few minutes until a large amount of a red microcrystalline solid started to precipitate. Once the precipitation was complete as indicated by the loss of the red color of the solution, the solvent was filtered off and the resulting red filter cake was dried under high vacuum to give complex [**12**·thf] as a red microcrystalline solid material (259 mg). The pale orange filtrate was stored for at least 1 d at −20°C causing the formation of a second crop of pale orange crystals, which were collected and dissolved in *n*-pentane (9 mL) to form again a deep red solution. After vigorous stirring for a few minutes, a second crop of red microcrystalline solid material started to precipitate, which was filtered off and dried under high vacuum (46.7 mg). Combined yield of the dark red complex [**12**·thf]: 306 mg, 55%.

Complex [**12**·thf] has a broad and featureless ^{1}H NMR spectrum. Elemental analysis (%) calculated for $C_{76}H_{77}MoO_4Si_3$: C 73.93, H 6.29, Mo 7.77, Si 6.82; found: C 73.72 H 6.34, Mo 7.75, Si 6.81.

The crystallization of this complex proved to be extremely challenging, as SC-XRD revealed that all the

crystals examined were multicomponent, containing complex [**12**·thf] as the major species, along with two minor constituents. These minor species were identified as a η^6 species **12** devoid of any THF ligands and a third species, which could not be unambiguously assigned (see Crystallographic Section of the Supporting Information).

Single crystals were obtained using different methods in numerous attempts at isolating the η^2 species in pure form. A typical crystallization experiment is described below:

To a colorless solution of ligand **9b** (158 mg, 0.147 mmol) in THF (9 mL) was added a dark red solution of complex **1** (98.3 mg, 0.157 mmol) in THF (9 mL). The resulting dark red solution was concentrated to ca. 1/4 of its volume to give a dark red-brown suspension, which was filtered under Ar. The pale orange filter cake was dissolved in *n*-pentane (3 mL) to form a deep red solution, from which red crystals precipitated after some minutes. The solvent was filtered off, and the resulting red filter cake was dissolved in toluene (1.5 mL; added in 0.5 mL increments). The solution was carefully layered with *n*-pentane (4.5 mL). Short vigorous stirring was necessary to initiate the precipitation of a red solid. The mixture was then left standing for approximately 1 h before the supernatant solution was filtered off and discarded. The red filter cake was dissolved again in toluene (1 mL; added in 0.5 mL increments) and the resulting solution was again carefully layered with *n*-pentane (3 mL). Over time, red single crystals began to form, which were used for numerous XRD analyses.

Notably, after precipitation from *n*-pentane, the crystals no longer dissolve in *n*-pentane.

Reversible Loss and Binding of THF

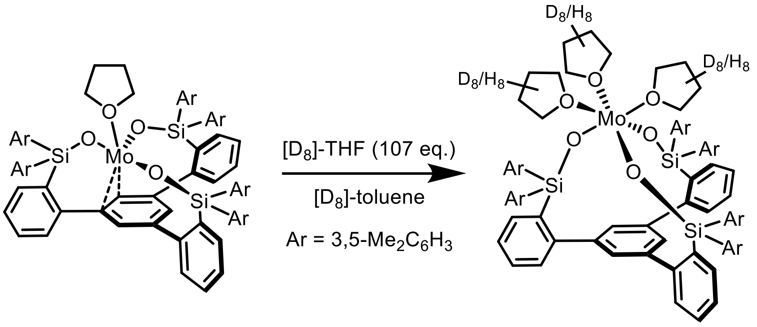

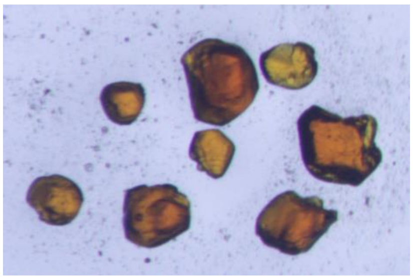

[D_8]-THF (0.4 mL) was added to a dark red solution of complex [**12**·thf] (56.6 mg, 0.0458 mmol) in [D_8]-toluene (0.4 mL). The resulting solution was transferred into a J-Young NMR tube and spectra were recorded at different temperatures. Over time, crystals started to precipitate from this solution. Some of these crystals were transferred to a microscope slide, where the crystalline batch appeared homogeneous (see inserted photograph). Determination of the unit cell of these crystals proved that the tris-THF adduct [**12**·3thf] had formed.

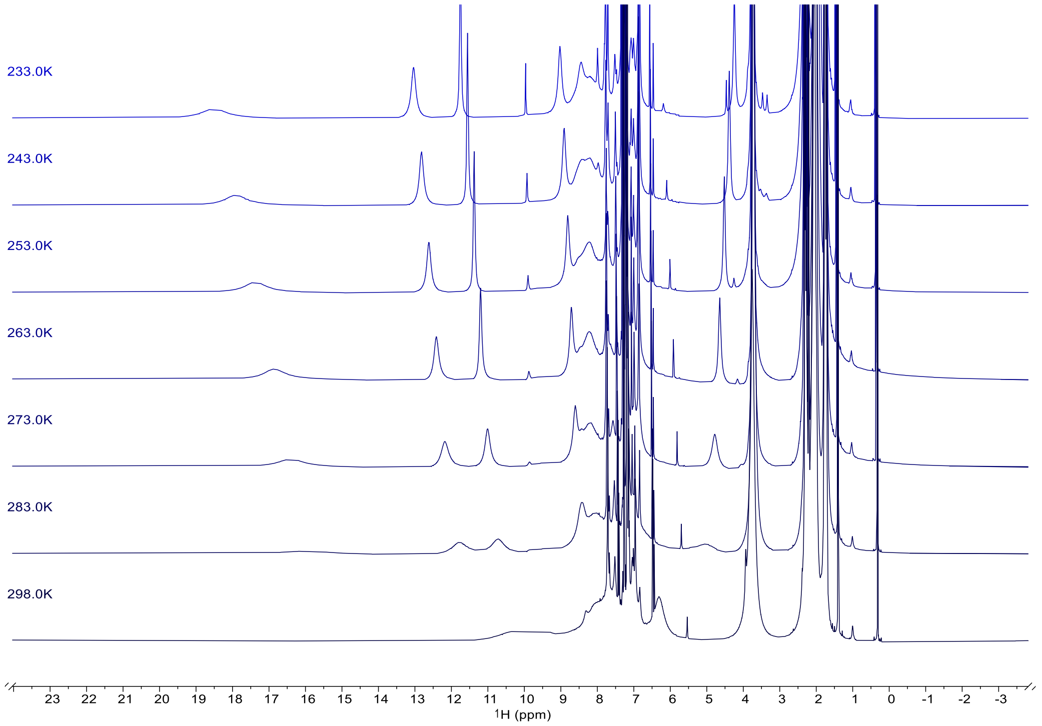

Figure S1. ^{1}H NMR spectra of a solution of complex [**12**·thf] and [D_8]-THF in [D_8]-toluene (233 K−298 K, 600 MHz)

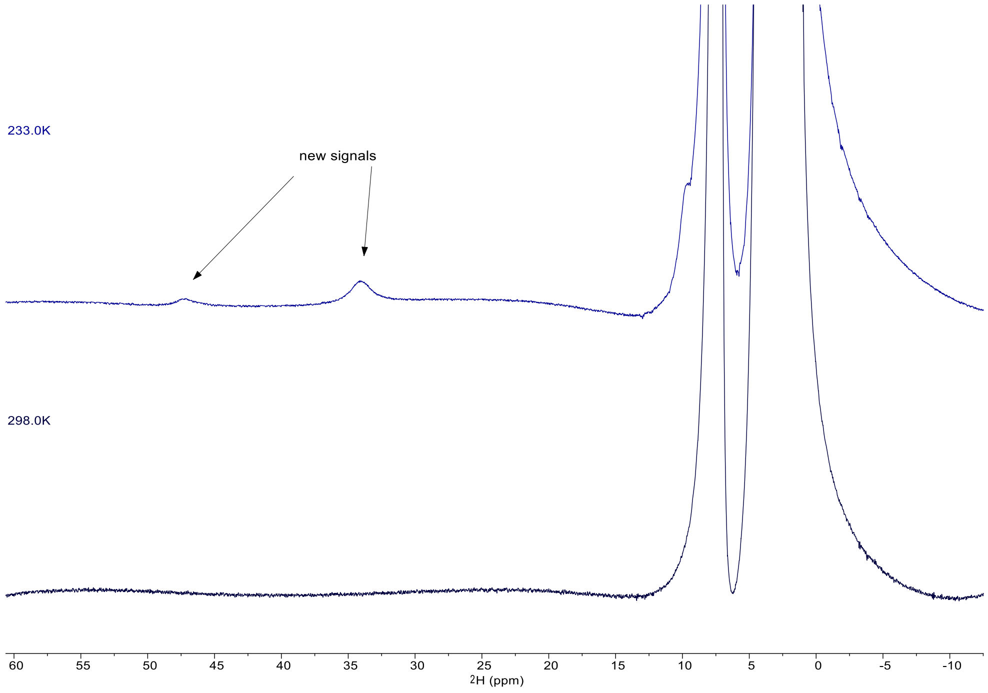

Figure S2. ^{2}H NMR spectra of a solution of complex [**12**·thf] and [D_8]-THF in [D_8]-toluene (233 K−298 K, 92 MHz)

Reaction of Complex [**12**·thf] with N_2O

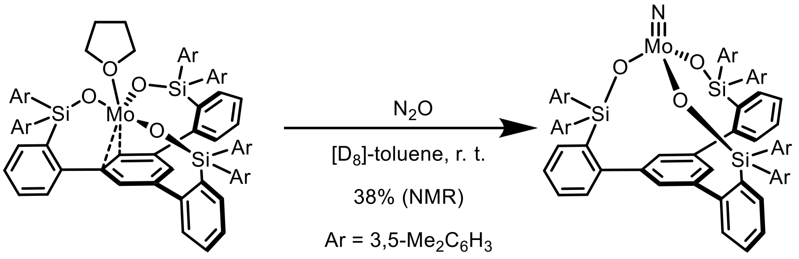

Complex [**12**·thf] (37.3 mg, 0.0302 mmol) was weighed into a flame-dried 10-mL Schlenk flask under argon. The flask was capped with a rubber septum and the atmosphere was exchanged for N_2O (1 bar). A solution of 1,2,4,5-tetramethylbenzene in [D_8]-toluene (1 mL, 0.1431 M) was then added, causing the immediate formation of a golden solution. After 2 min the solution was transferred into a J Young NMR tube and spectra of the mixture were recorded. It was determined *via* quantitative NMR studies that the nitrido complex **7b** had been formed in 38% yield (against 1,2,4,5-tetramethylbenzene as the internal standard).[10] While no other species besides the nitrido complex **7b** were detected, the sample exhibited a broad background, suggesting possible oligomer formation. The nitrido complex **7b** was also the only species identified by mass spectrometry, which gave no evidence for any nitrosyl containing species.

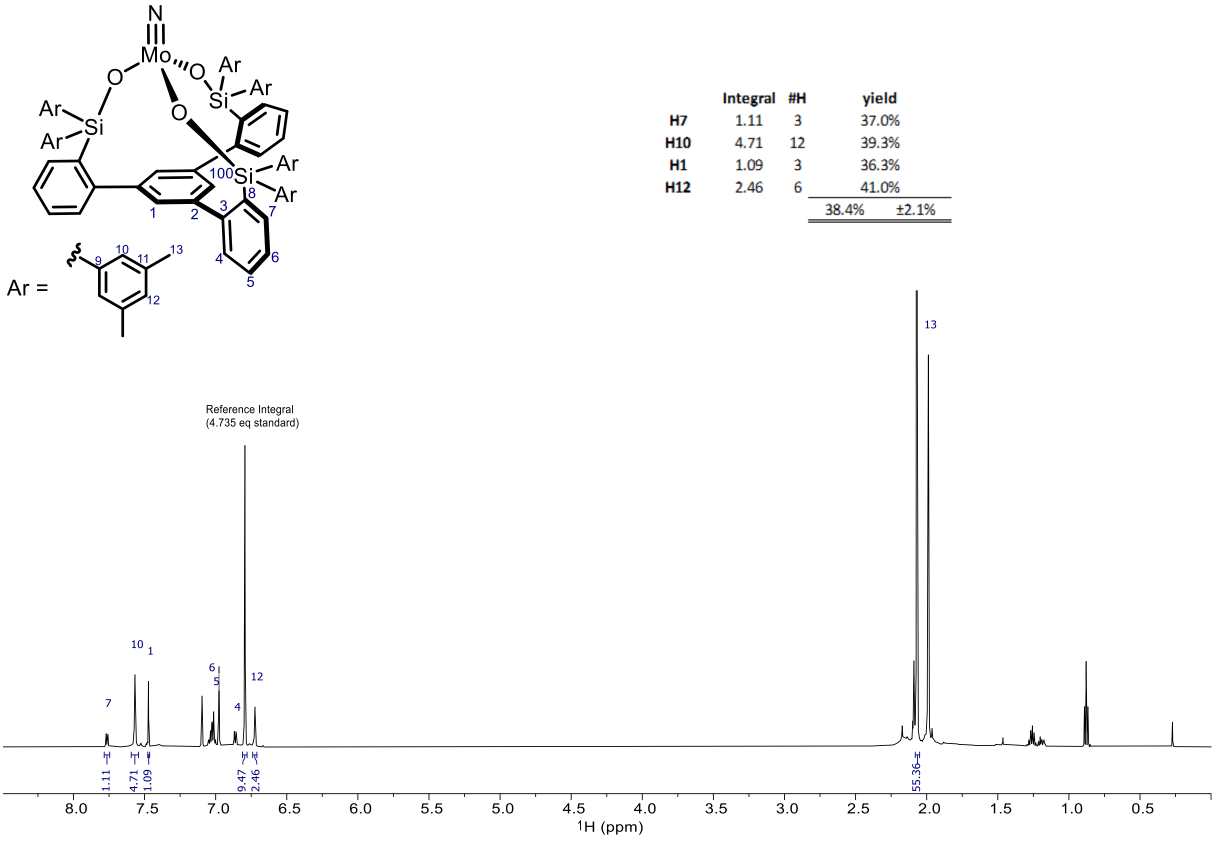

Figure S3. Quantitative ^{1}H NMR spectrum of the crude product formed upon reaction of complex [**12**·thf] and N_2O ([D_8]-toluene, 298 K, 600 MHz)

Reaction of Complex [**12**·thf] with 1,1-Dichloropropane

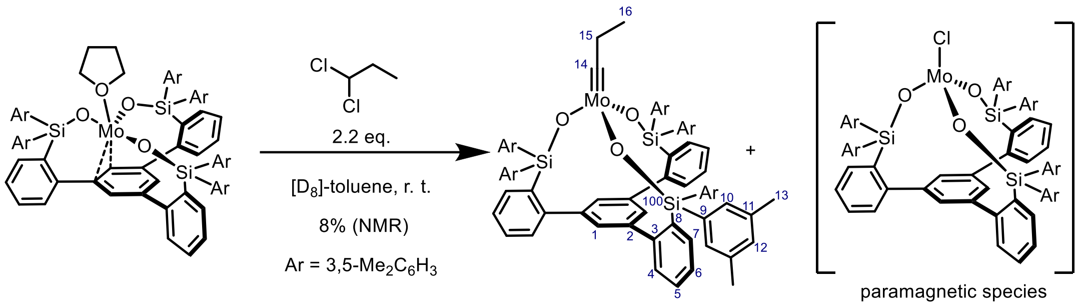

1,1-Dichloropropane (10 µL, 0.10 mmol) was added to a stirred red solution of complex [**12**·thf] (55.3 mg, 0.0448 mmol) in [D$_8$]-toluene (0.7 mL). The resulting dark brown solution was transferred into a J Young NMR tube and spectra were recorded, which showed that the alkylidyne **7a’** had been formed. Additionally, the NMR spectra indicated the presence of a paramagnetic species, which might be the corresponding Mo(+IV) chloro complex.[3] When 1,2,4,5-tetramethylbenzene was used as an internal standard, it was possible to determine the yield of alkylidyne **7a’** to be in the range of 10%.

NMR data of the alkylidyne **7a’**: ^{1}H NMR (600 MHz, [D$_8$]-toluene, 298 K): δ = 7.83 (d, *J* = 7.1 Hz, 3H; H-7), δ = 7.58 (s, 12H; H-10), δ = 7.47 (s, 3H; H-1), δ = 7.04 (t, *J* = 7.4 Hz, 3H; H-5), δ = 6.87 (d, *J* = 7.4 Hz, 3H; H-4), δ = 6.79 (s, 6H; H-12), δ = 2.84 (q, *J* = 7.4 Hz, 2H; H-15), δ = 2.04 (s, 36H; H-13), δ = 0.66 ppm (t, *J* = 7.4 Hz, 3H; H-16). The signal of H-6 overlapped with one of the solvent signals at 7.09 ppm. ^{13}C NMR (151 MHz, [D$_8$]-toluene, 298 K): δ = 322.8 (C-14), 149.4 (C-3), 143.8 (C-2), 138.6 (C-9), 137.2 (C-11), 136.3 (C-8), 132.7 (C-10), 131.6 (C-12), 130.1 (C-4), 129.7 (C-5), 129.5 (C-1), 126.1 (C-6), 44.5 (C-15), 21.4 (C-13), 13.7 ppm (C-16). The signal of C-7 overlapped with one of the solvent signals at 137.5 ppm. ^{29}Si NMR (119 MHz, [D$_8$]-toluene, 298 K): δ = −10.5 ppm (Si-100).

MS analysis of the crude product showed the presence of a cationic species that could result from loss of chloride from the putative chloro complex formed along with **7a’**:[3] HRMS (ESI^{+}): m/z calculated for $C_{72}H_{69}MoO_3Si_3$ [M]$^{+}$: 1163.36031, found: 1163.36019.

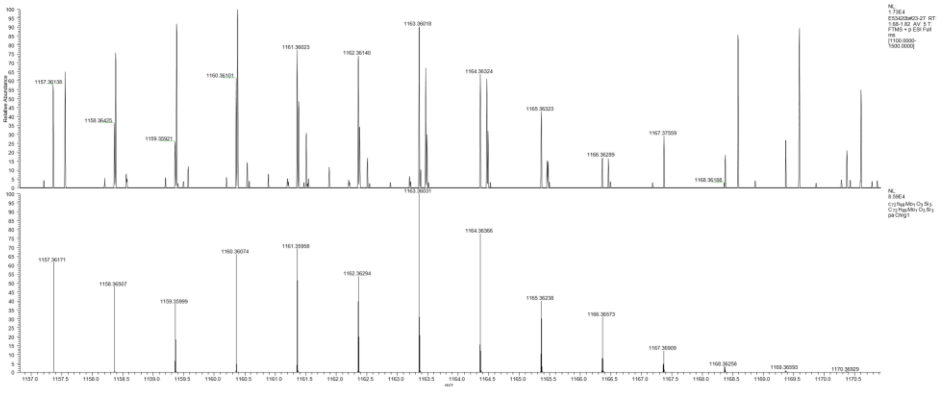

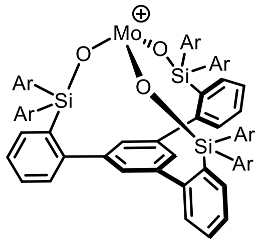

Ar = 3,5-Me$_2$C$_6$H$_3$

Figure S4. Recorded (top) and simulated (bottom) ESI^{+}-MS spectrum of a cationic molybdenum-containing species corresponding to a molecular formula of $C_{72}H_{69}MoO_3Si_3$.

¹H NMR spectrum of the crude product formed on reaction of complex [12·thf] with 1,1-dichloropropane: [D₈]-toluene, 298 K, 600 MHz.

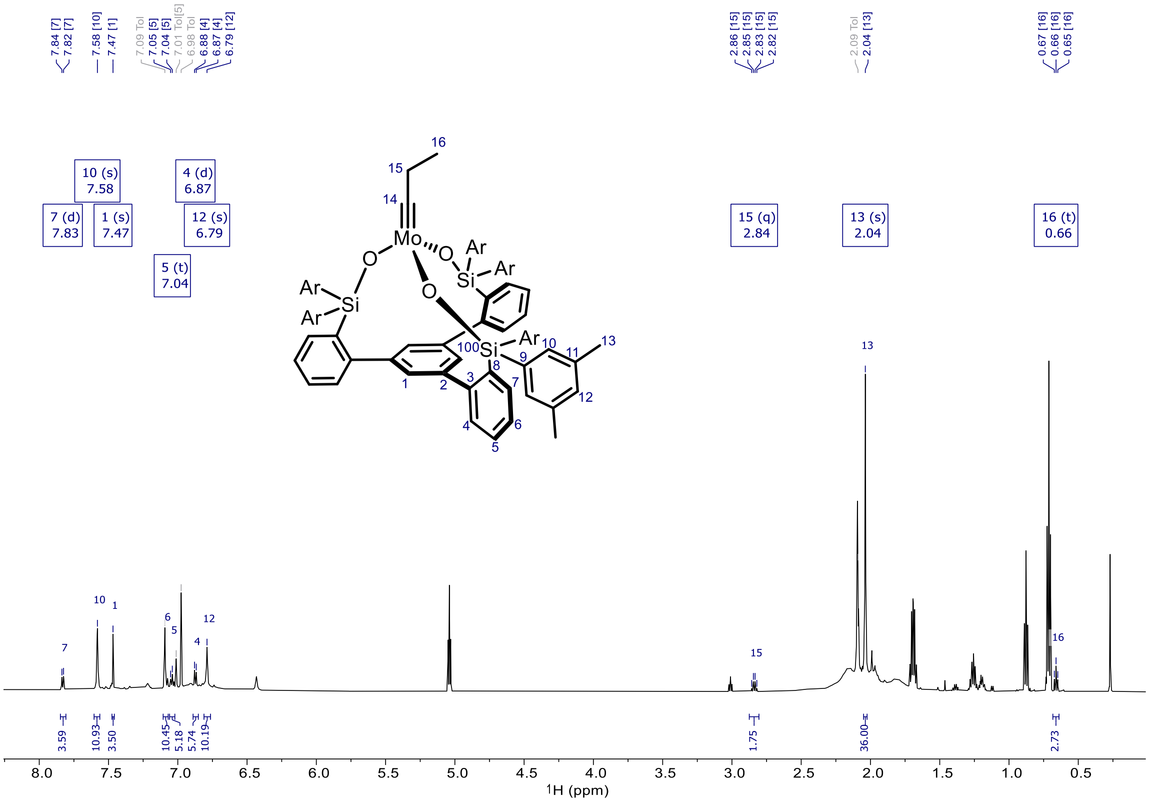

¹³C NMR spectrum spectrum of the crude product formed on reaction of complex [12·thf] with 1,1-dichloropropane: [D₈]-toluene, 298 K, 151 MHz.

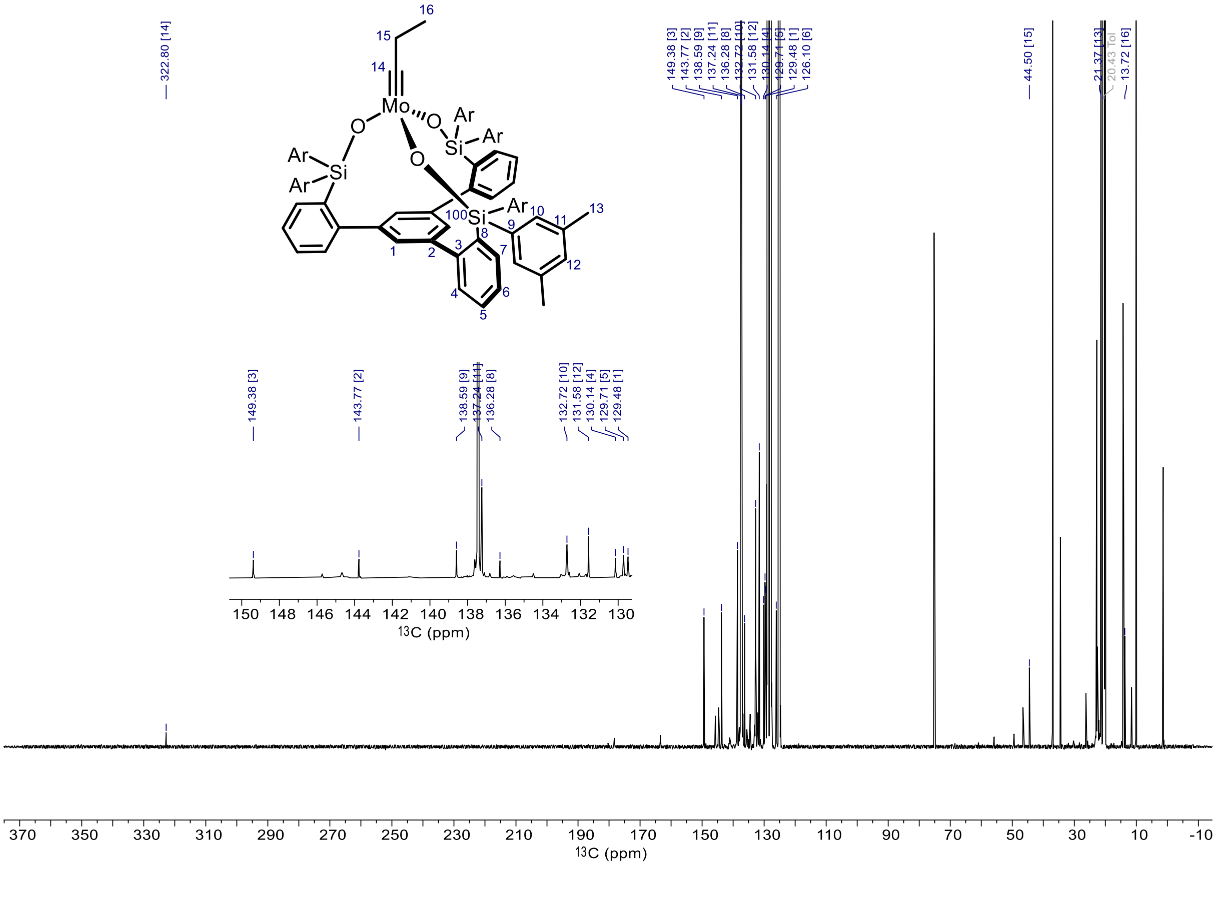

^{1}H-^{13}C HMBC NMR spectrum spectrum of the crude product formed on reaction of complex [12·thf] with 1,1-dichloropropane: [D$_8$]-toluene, 298 K, 600 MHz, 151 MHz.

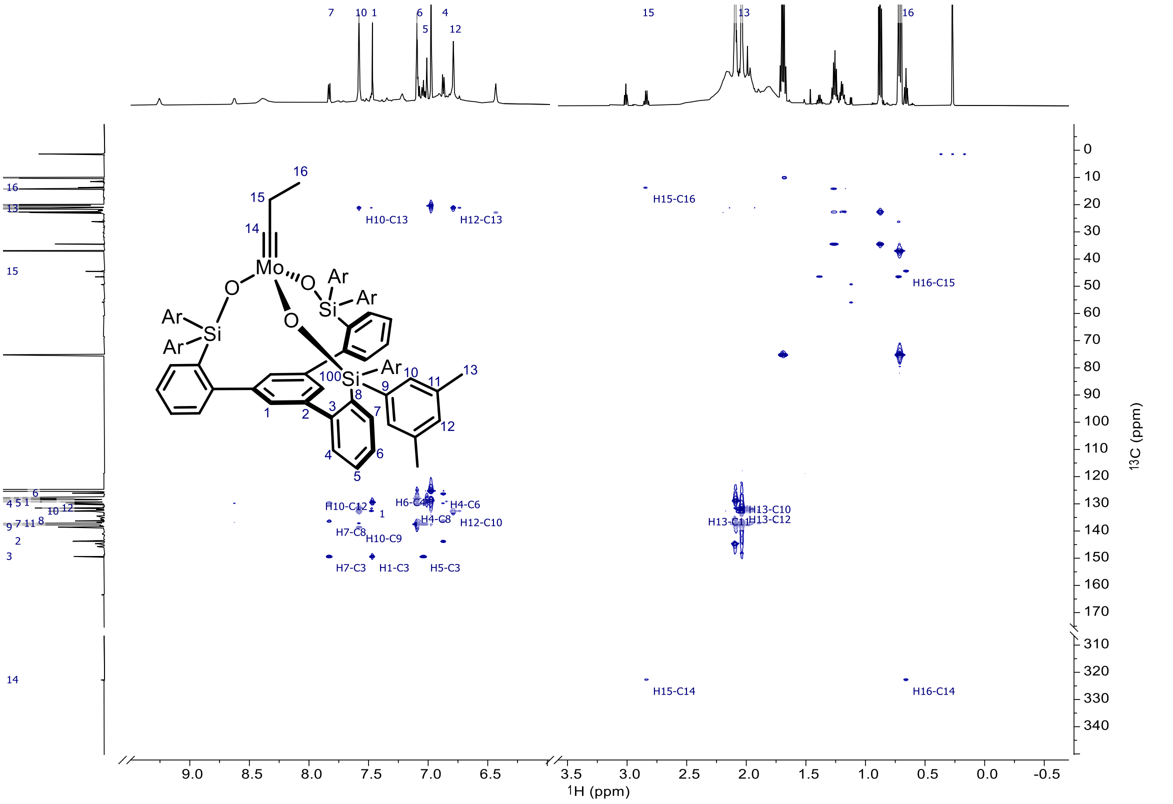

^{1}H-^{1}H COSY NMR spectrum spectrum of the crude product formed on reaction of complex [12·thf] with 1,1-dichloropropane: [D$_8$]-toluene, 298 K, 600 MHz, 600 MHz.

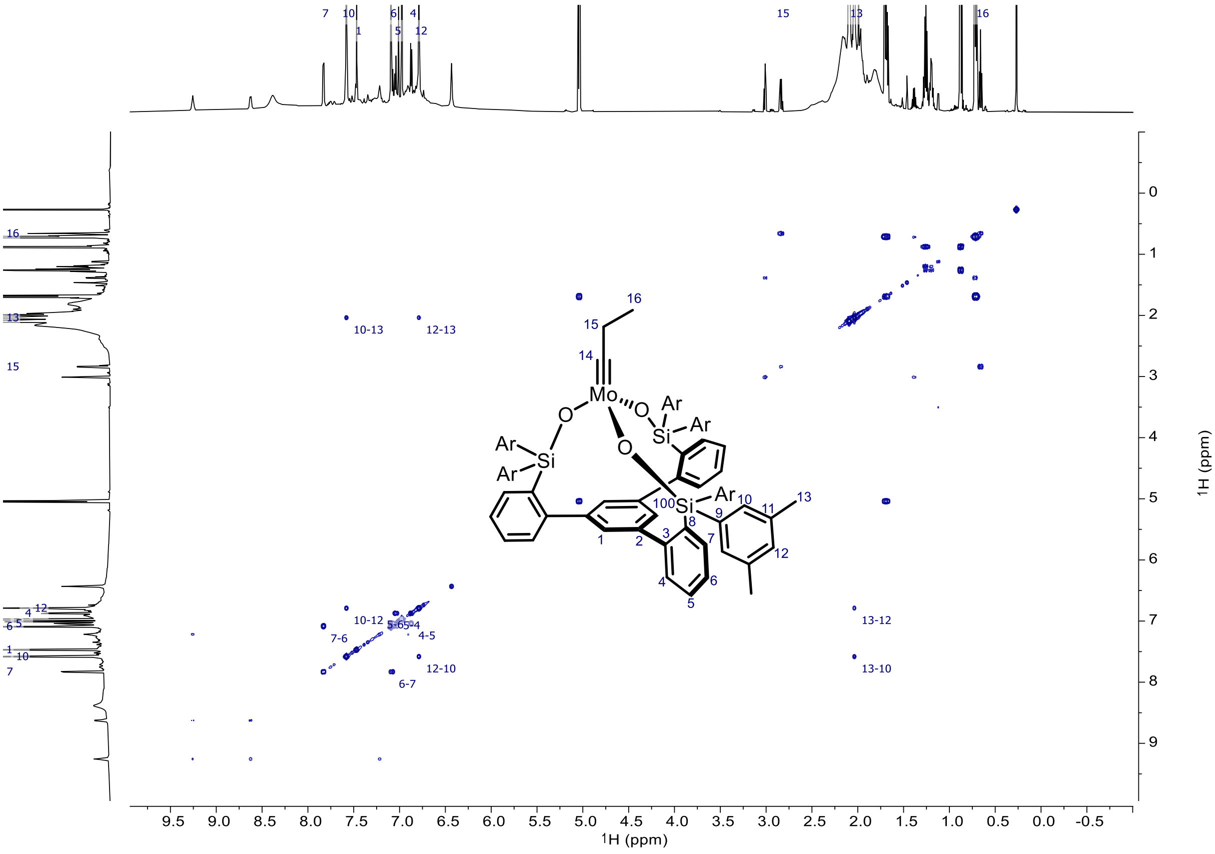

^{1}H-^{1}H ROESY NMR spectrum spectrum of the crude product formed on reaction of complex [12·thf] with 1,1-dichloropropane: [D$_8$]-toluene, 298 K, 600 MHz, 600 MHz.

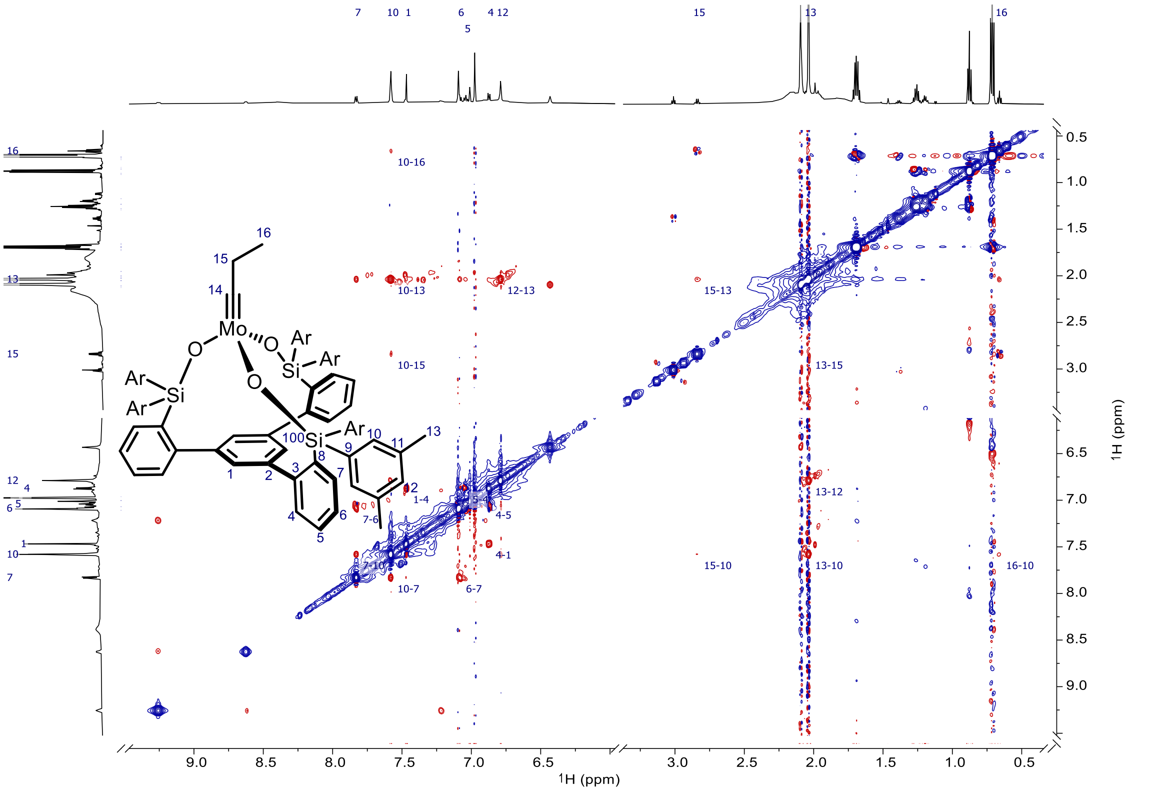

Top: ^{29}Si NMR spectrum of spectrum of the crude product formed on reaction of complex [12·thf] with 1,1-dichloropropane: [D$_8$]-toluene, 298 K, 119 MHz. **Bottom: ^{1}H-^{29}Si HMBC NMR spectrum:** [D$_8$]-toluene, 298 K, 600 MHz, 119 MHz.

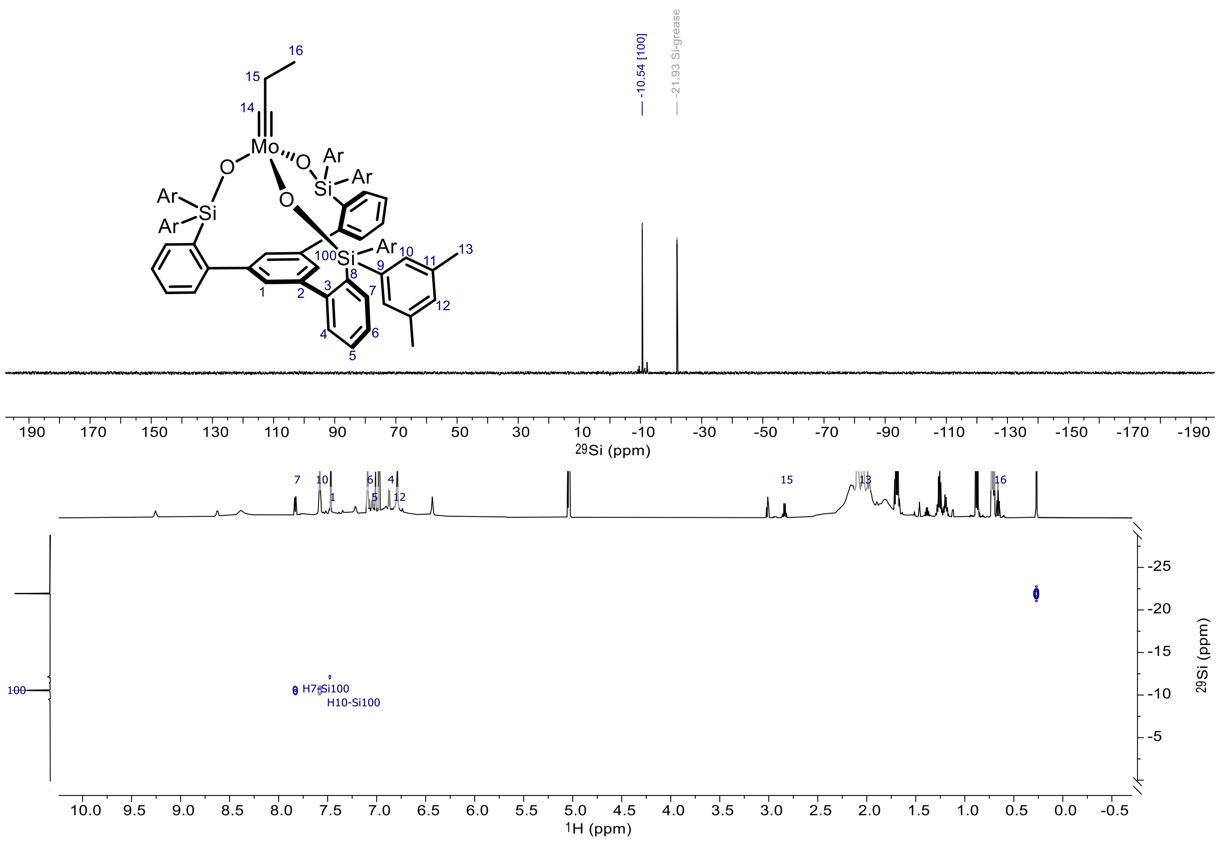

Heterodimer Formation by Reaction of Complex [**12**·thf] with Complex **1**

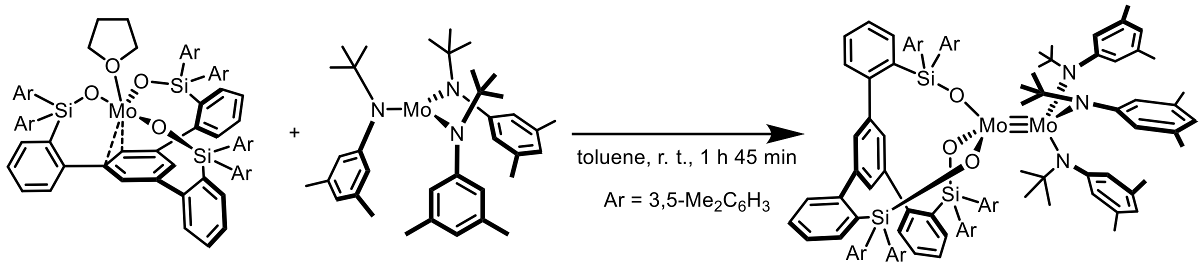

Toluene (1.2 mL) was added to a solid mixture of [**12**·thf] (19.5 mg, 0.0158 mmol) and the Cummins complex **1** (10.8 mg, 0.0173 mmol). The resulting dark brown mixture was stirred at room temperature for 1 h 45 min, before all volatile components were removed *in vacuo* (10^{-3} mbar). An NMR analysis of this crude product showed that the heterodimer **14** had been cleanly formed. In addition to **14**, excess of Cummins complex **1** and trace quantities of [HN(*t*Bu)Ar] (Ar = 3,5-dimthylphenyl) were detected (which was trace impurity in sample of **1** used to perform this experiment).

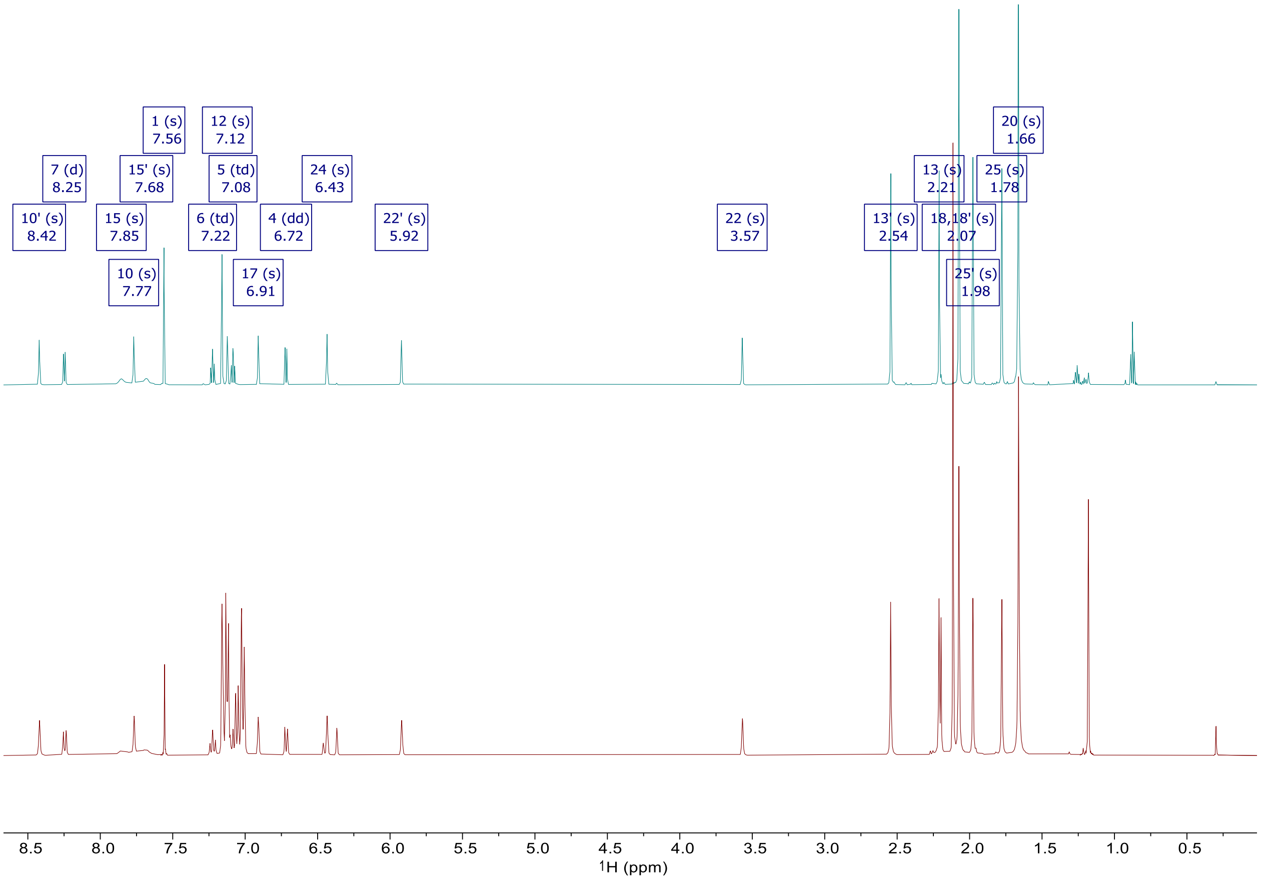

Figure S5. Top: ^{1}H NMR spectrum of authentic complex **14** ([D$_6$]-benzene, 298 K, 600 MHz); bottom: ^{1}H NMR spectrum of the crude product formed upon reaction of [**12**·thf] with **1** ([D$_6$]-benzene, 298 K, 400 MHz)

Complex **13**

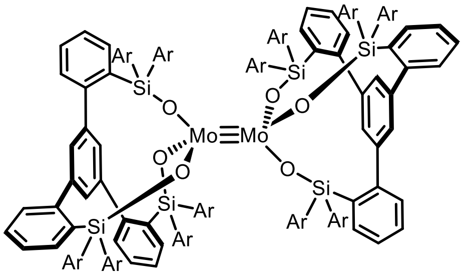

Ar = 3,5-$Me_2C_6H_3$

A dark red solution of complex **1** (190 mg, 0.304 mmol) in THF (18 mL) was added to a colorless solution of ligand **9b** (313 mg, 0.292 mmol) in THF (18 mL). The resulting dark red solution was concentrated to ca. 1/4 of its volume to give a dark red-brown suspension, which was filtered under Ar to give complex [**12**·3thf] as the pale orange filter cake. The pale orange filter cake was dissolved in *n*-pentane (8 mL) to form a deep red solution, which vigorously stirred for a few minutes to cause precipitation of a large amount of a red microcrystalline solid. The supernatant solvent was filtered off and the resulting red filter cake was dried under high vacuum to give complex [**12**·thf] as a red microcrystalline solid.

This product was dissolved in toluene (2 mL) and *n*-pentane (6 mL) was added while stirring, which led to the precipitation of a red crystalline material. All volatile components were removed *in vacuo* (10^{-3} mbar). The residual solid was again dissolved in toluene (15 mL) and then all volatile components were removed under high vacuum at RT to give a dark red oil. This dissolution/drying procedure was repeated two more times, before the residue was dissolved in toluene (2 mL) and the resulting red solution was filtered *via* cannula and left standing for 11 d, leading to the formation of orange single crystals. The supernatant solvent was filtered off *via* cannula and the large crystals were collected and used for crystal structure analysis. Drying of the crystals under high vacuum afforded the title complex as a purple powder (13.1 mg, 4%).

The title compound is almost insoluble in toluene, THF or CD_2Cl_2; therefore, no NMR data could be recorded.

Elemental analysis (%) calculated for $C_{144}H_{138}Mo_2O_6Si_6$: C 74.39, H 5.98, Mo 8.25, Si 7.25; found: C 74.27 H 6.02, Mo 8.21, Si 7.29.

HRMS (ESI$^+$): m/z calculated for $C_{148}H_{146}Mo_2O_7Si_6$ [M+THF]$^+$: 2398.77868, found: 2398.78455.

Complex **14**

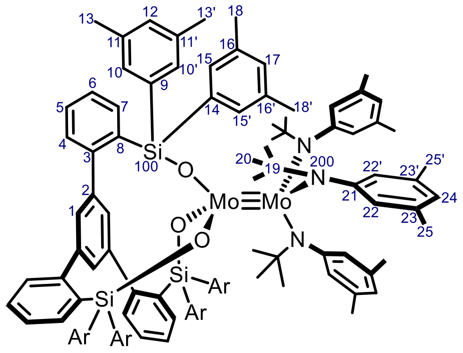

A colorless solution of ligand **9b** (162 mg, 0.152 mmol) in Et_2O (15 mL) was added *via* cannula over a period of 2 min to a dark red solution of complex **1** (207 mg, 0.331 mmol) in Et_2O (15 mL) at −78 °C. Stirring was continued for 30 min at −78 °C before the red brown suspension was allowed to reach ambient temperature. After a total reaction time of 1 h and 10 min, all volatile components were removed *in vacuo* (10^{-3} mbar) to give a brown sticky solid. This residue was triturated with *n*-pentane (3 x 10 mL) and the filter cake was dried under high vacuum (10^{-3} mbar) to give the title complex as a dark green-brown solid (143 mg, 53%). Orange single crystals suitable for X-ray diffraction were grown by slow evaporation of a solution of **14** in Et_2O.

^{1}H NMR (600 MHz, [D$_6$]-benzene, 298 K): δ = 8.42 (s, 3H; H-10'), 8.25 (d, *J* = 7.4 Hz, 3H ; H-7), 7.85 (bs, 3H; H-15), 7.77 (s, 3H; H-10), 7.68 (bs, 3H; H-15'), 7.56 (s, 3H; H-1), 7.22 (td, *J* = 7.6, 1.0 Hz, 3H; H-6), 7.12 (s, 3H; H-12), 7.08 (td, *J* = 7.5, 1.1 Hz, 3H; H-5), 6.91 (s, 3H; H-17), 6.72 (dd, *J* = 7.6, 1.5 Hz, 3H; H-4), 6.43 (s, 3H; H-24), 5.92 (s, 3H; H-22'), 3.57 (s, 3H; H-22), 2.54 (s, 9H; H-13'), 2.21 (s, 9H; H-13), 2.07 (s, 18H; H-18 and H-18'), 1.98 (s, 9H; H-25'), 1.78 (s, 9H; H-25), 1.66 (s, 27H; H-20).

^{13}C NMR (151 MHz, [D$_6$]-benzene, 298 K): δ = 151.9 (C-21), 150.2(C-3), 143.9 (C-2), 140.0 (C-14), 139.5 (C-10‘), 139.1 (C-10), 139.0 (C-7), 138.6 (C-9), 138.4 (C-8), 136.6 (bs, C-16‘), 136.0 (bs, C-16), 135.5 (C-11), 135.2 (bs, C-15), 135.1 (C-23), 134.7 (C-23‘), 134.6 (C-11‘), 134.5 (bs, C-15‘), 132.3 (C-22‘), 131.8 (C-12), 130.9 (C-17), 130.4 (C-22), 130.0 (C-4), 129.6 (C-5), 127.0 (C-24), 126.1 (C-6), 63.7 (C-19), 36.2 (C-20), 21.68 (C-13), 21.66 (C-25 and C13‘), 21.5 (bs, C-18 and C-18‘), 20.6 (C-25‘) ppm.

^{29}Si NMR (119 MHz, [D$_6$]-benzene, 298 K): δ = –5.0 ppm.

^{15}N NMR (61 MHz, [D$_6$]-benzene, 298 K): δ = −129 ppm.

^{95}Mo NMR (26 MHz, [D$_8$]-toluene, 333 K): 3314.1 ppm

IR (ATR): ṽ = 2915, 1598, 1584, 1456, 1413, 1375, 1355, 1266, 1177, 1142, 1087, 1042, 960, 928, 866, 823, 765, 741, 723, 699, 685, 636, 624, 584, 551, 536 cm^{-1}.

HRMS (ESI$^+$): *m/z* calculated for $C_{108}H_{123}Mo_2N_3O_3Si_3$ [M]$^+$: 1789.69749, found: 1789.69813.

Elemental analysis (%) calculated for $C_{108}H_{123}Mo_2N_3O_3Si_3$: C 72.58, H 6.94, Mo 10.74, N 2.35, Si 4.71; found: C 72.15, H 6.89, Mo 10.61, N 2.33, Si 4.66.

Complex **16**

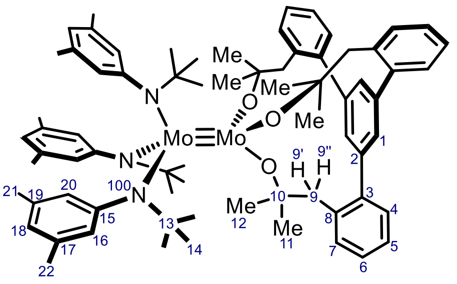

A dark red solution of complex **1** (964 mg, 1.54 mmol) in toluene (24 mL) was added dropwise over a period of 5 min to a colorless solution of ligand **15** (366 mg, 0.701 mmol) in toluene (35 mL). The resulting dark brown solution was stirred for 2 d at ambient temperature before all volatiles were removed *in vacuo* (10^{-3} mbar). The residue was washed with *n*-pentane (6 x 20 mL) and the filter cake dried under high vacuum (10^{-3} mbar) to give the title complex as a light brown solid (671 mg, 77%). Orange single crystals suitable for X-ray diffraction were grown by slow diffusion of *n*-pentane vapor into a solution of **16** in benzene.

^{1}H NMR (600 MHz, [D$_8$]-toluene, 298 K): δ = 7.41 (dd, *J* = 7.8, 1.2 Hz, 3H; H-7), 7.27 (dd, *J* = 7.6, 1.5 Hz, 3H; H-4), 7.25 (bs, $\Delta\nu_{1/2}$ = 10.0 Hz, 3H; H-16), 7.16 (td, *J* = 7.6, 1.5 Hz, 3H; H-6), 7.07 (td, *J* = 7.5, 1.2 Hz, 3H; H-5), 6.87 (s, 3H; H-1), 6.64 (s, 3H; H-18), 4.19 (bs, $\Delta\nu_{1/2}$ = 12.6 Hz, 3H; H-20), 2.92 (d, ^{2}J = 12.8 Hz, 3H; H-9’), 2.31 (s, 9H; H-22), 2.18 (s, 9H; H-12), 2.00 (s, 9H; H-21), 1.91 (d, ^{2}J = 12.9 Hz, 3H; H-9’’), 1.79 (s, 27H; H-14), 1.22 ppm (s, 9H; H-11).

^{13}C NMR (151 MHz, [D$_8$]-toluene, 298 K): δ = 151.4 (C-15), 143.3 (C-3), 142.3 (C-2), 138.0 (C-8), 135.7 (C-19), 135.0 (C-17), 133.6 (C-16), 132.6 (C-20), 129.7 (C-1), 129.3 (C-7), 128.4 (C-4), 127.6 (C-6), 126.9 (C-18), 125.8 (C-5), 85.1 (C-10), 62.6 (C-13), 53.4 (C-9), 33.5 (C-14), 31.9 (C-12), 29.0 (C-11), 21.9 (C-21), 21.4 ppm (C-22).

^{15}N NMR (61 MHz, [D$_8$]-toluene, 298 K): δ = −147 ppm.

IR (ATR): ṽ = 2976, 2921, 2861, 1584, 1484, 1456, 1417, 1373, 1358, 1318, 1283, 1208, 1174, 1126, 1116, 1035, 1016, 978, 953, 934, 908, 884, 851, 777, 753, 728, 720, 693, 682, 627, 582, 564, 548, 500, 483, 463, 420 cm^{-1}.

HRMS (ESI^{+}): *m/z* calculated for $C_{72}H_{93}Mo_2N_3O_3$ [M]$^{+}$: 1243.53196, found: 1243.53555.

Elemental analysis (%) calculated for $C_{72}H_{93}Mo_2N_3O_3$: C 69.71, H 7.56, Mo 15.47, N 3.39; found: C 69.57, H 7.49, Mo 15.39, N 3.41.

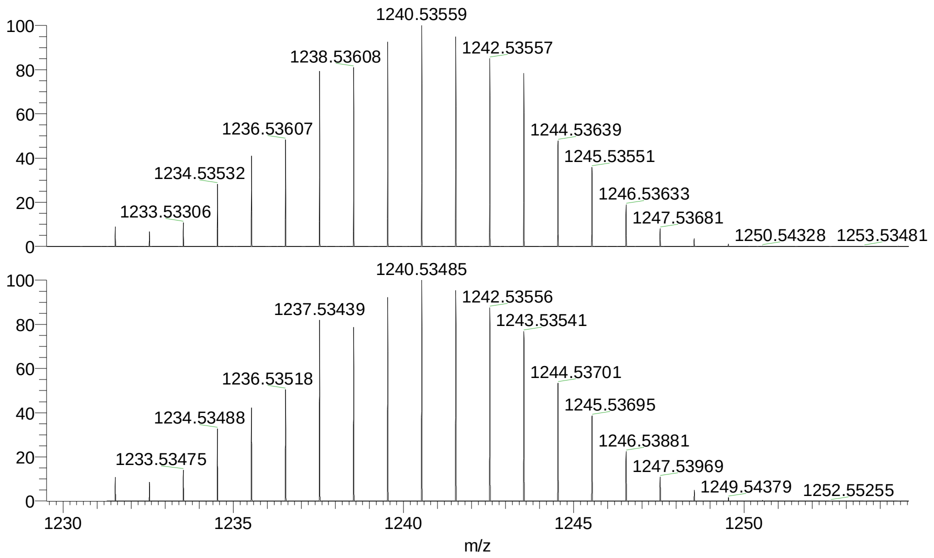

Figure S6. Recorded (top) and simulated (bottom) ESI$^+$-MS spectra of **16** considering the isotope distribution pattern; this analysis suggested that the molecular formula of the product is $C_{72}H_{93}Mo_2N_3O_3$.

Complex **17**

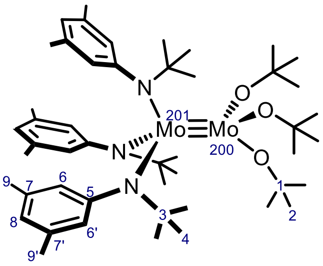

A colorless solution of *t*BuOH in toluene (0.0709 м, 21 mL, 1.5 mmol) was added to a dark red solution of complex **1** (580 mg, 0.928 mmol) in toluene (14 mL). The resulting dark red/brown solution was stirred for 3 d at ambient temperature before all volatile components were removed *in vacuo* (10^{-3} mbar). The flask was equipped with a short distillation bridge without a cooling jacket. To remove most of the aniline [(*t*Bu)(Ar)NH] (Ar = 3,5-dimethylphenyl), the residue was heated to 80°C (oil bath) under high vacuum (10^{-3} mbar) for 20 min (the distillation was assisted by occasional heating of the glassware with a heat gun). After cooling to room temperature, the dark red brown residue was extracted with hexamethyldisiloxane (HMDSO, 2 x 20 mL, 1 x 10 mL; *Note*: The amount of HMDSO can vary depending on how effectively the aniline had been removed by distillation) and filtered *via* cannula. The resulting dark red solution was cooled from 20°C to –35°C over the course of 4 h and kept overnight at –35°C, but no crystals were formed at this point. Therefore, the dark red solution was allowed to warm to room temperature; gentle agitaton led to the formation of a small amount of a dark red crystalline solid. The resulting dark red mixture was again cooled from 20°C to –35°C over the course of 4 h and then kept at –35°C for 3 d until a large amount of dark red crystals had precipitated. The supernatant solution was filtered off at –35°C and the residue was dissolved in *n*-pentane (5 mL). All volatile components were removed *in vacuo* (10^{-3} mbar) from the dark red solution to give the title complex as a dark red solid containing traces of (*t*Bu)ArNH (<3%, see NMR) (188 mg, 43%). Orange single crystals suitable for X-ray diffraction were grown by slow cooling of a saturated solution of **17** in HMDSO from 20°C to –35°C.

^{1}H NMR (600 MHz, [D_8]-toluene, 233 K): δ = 7.28 (s, 3H; H-6'), 6.60 (s, 3H; H-8), 4.12 (s, 3H; H-6), 2.28 (s, 9H; H-9'), 2.00 (s, 9H; H-9), 1.84 (s, 27H; H-4), 1.50 ppm (s, 27H; H-2).

^{13}C NMR (151 MHz, [D_8]-toluene, 233 K): δ = 151.2 (C-5), 135.5 (C-7), 134.7 (C-7'), 133.3 (C-6'), 132.4 (C-6), 126.8 (C-8), 79.5 (C-1), 62.3 (C-3), 33.7 (C-2), 33.3 (C-4), 21.9 (C-9), 21.5 ppm (C-9').

^{15}N NMR (61 MHz, [D_8]-toluene, 233 K): δ = –147 ppm.

^{95}Mo NMR (26 MHz, [D_8]-toluene, 333 K): δ = 3260 (Mo-200), 3126 ppm (Mo-201).

IR (ATR): $\tilde{\nu}$ = 2969, 2924, 2863, 1597, 1583, 1458, 1379, 1357, 1283, 1225, 1170, 1143, 1040, 1019, 957, 937, 923, 899, 883, 847, 776, 717, 682, 580, 567, 551, 501, 469, 438, 420 cm^{-1}.

HRMS (ESI^+): *m/z* calculated for $C_{48}H_{81}Mo_2N_3O_3$ $[M]^+$: 943.43806, found: 943.43914.

Elemental analysis (%) calculated for $C_{48}H_{81}Mo_2N_3O_3$: C 61.33, H 8.69, Mo 20.38, N 4.47; found: C 60.57, H 8.66, Mo 19.99, N 4.38

NMR SPECTRA

^{1}H NMR spectrum of Bis(3,5-dimethylphenyl)dimethoxysilane **(S1):** CD_2Cl_2, 298 K, 600 MHz.

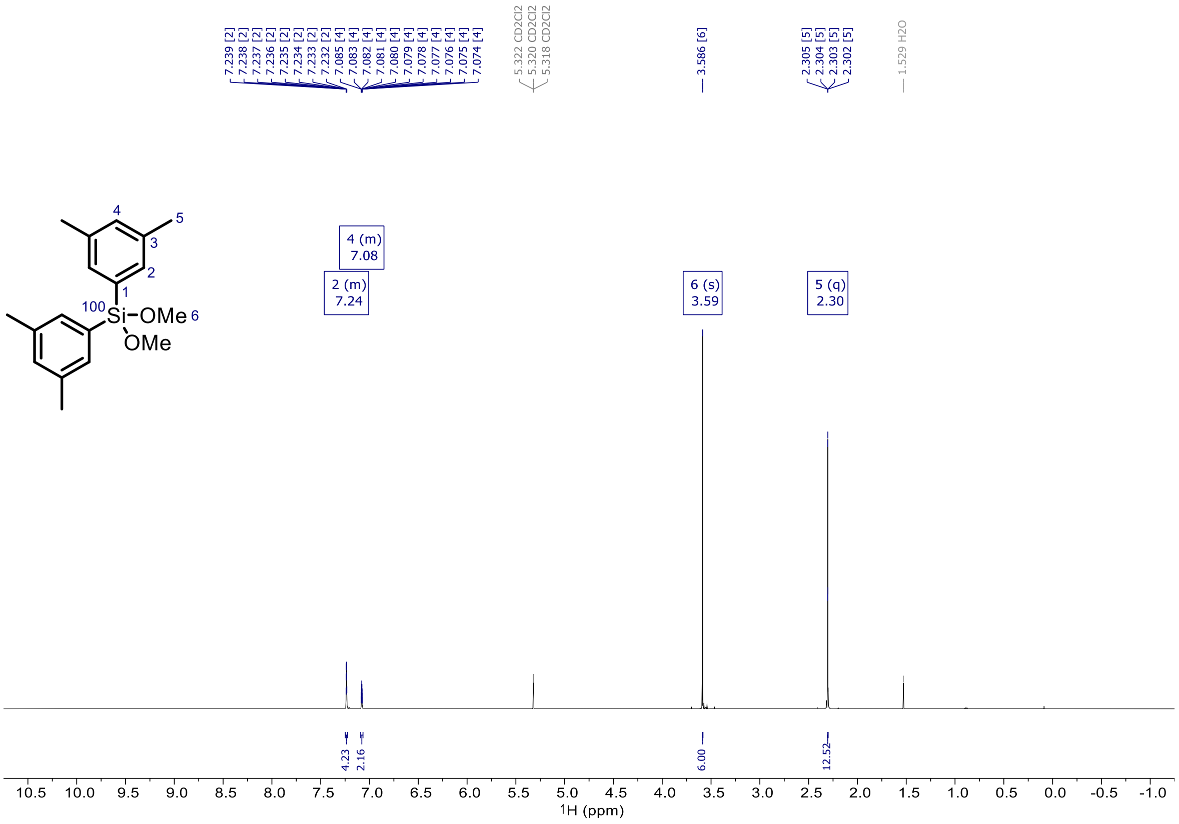

^{13}C NMR spectrum of Bis(3,5-dimethylphenyl)dimethoxysilane (S1): CD_2Cl_2, 298 K, 151 MHz.

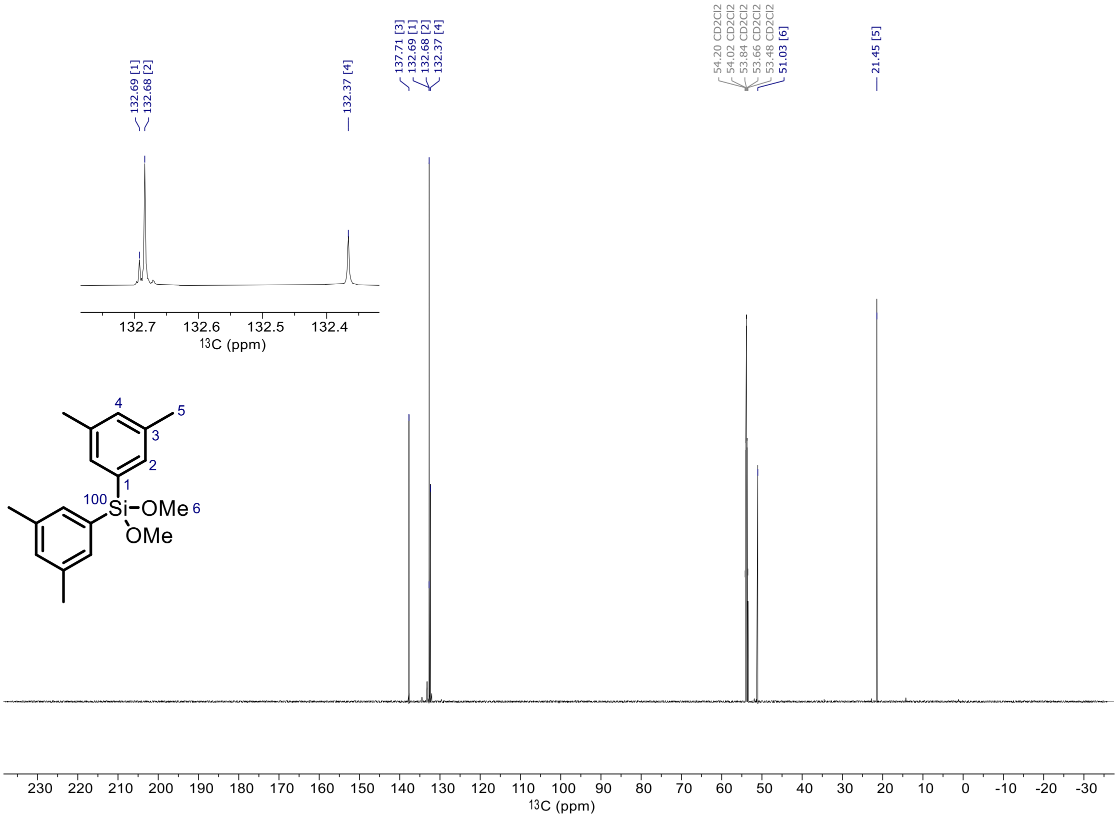

^{1}H-^{13}C HSQC NMR spectrum of Bis(3,5-dimethylphenyl)dimethoxysilane (S1): CD_2Cl_2, 298 K, 600 MHz, 151 MHz.

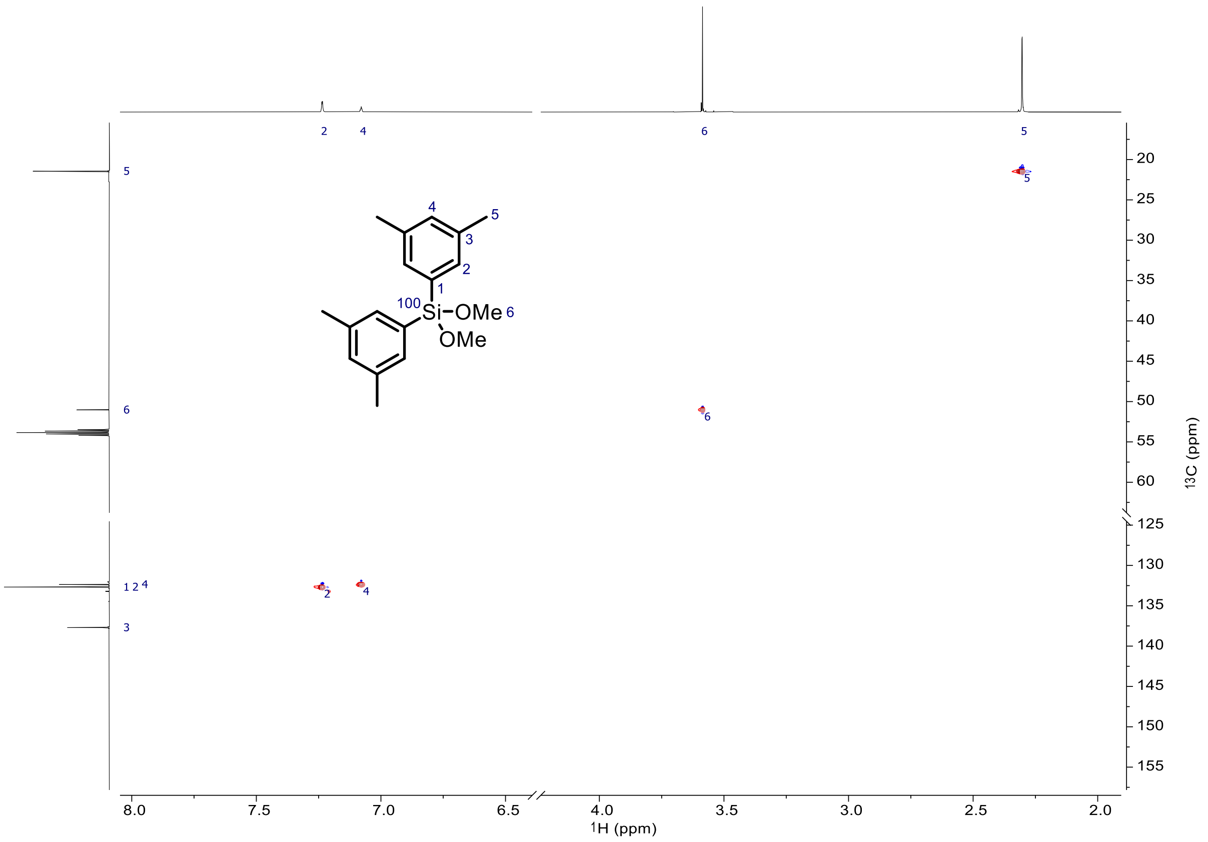

^{1}H-^{13}C HMBC NMR spectrum of Bis(3,5-dimethylphenyl)dimethoxysilane (S1): CD_2Cl_2, 298 K, 600 MHz, 151 MHz.

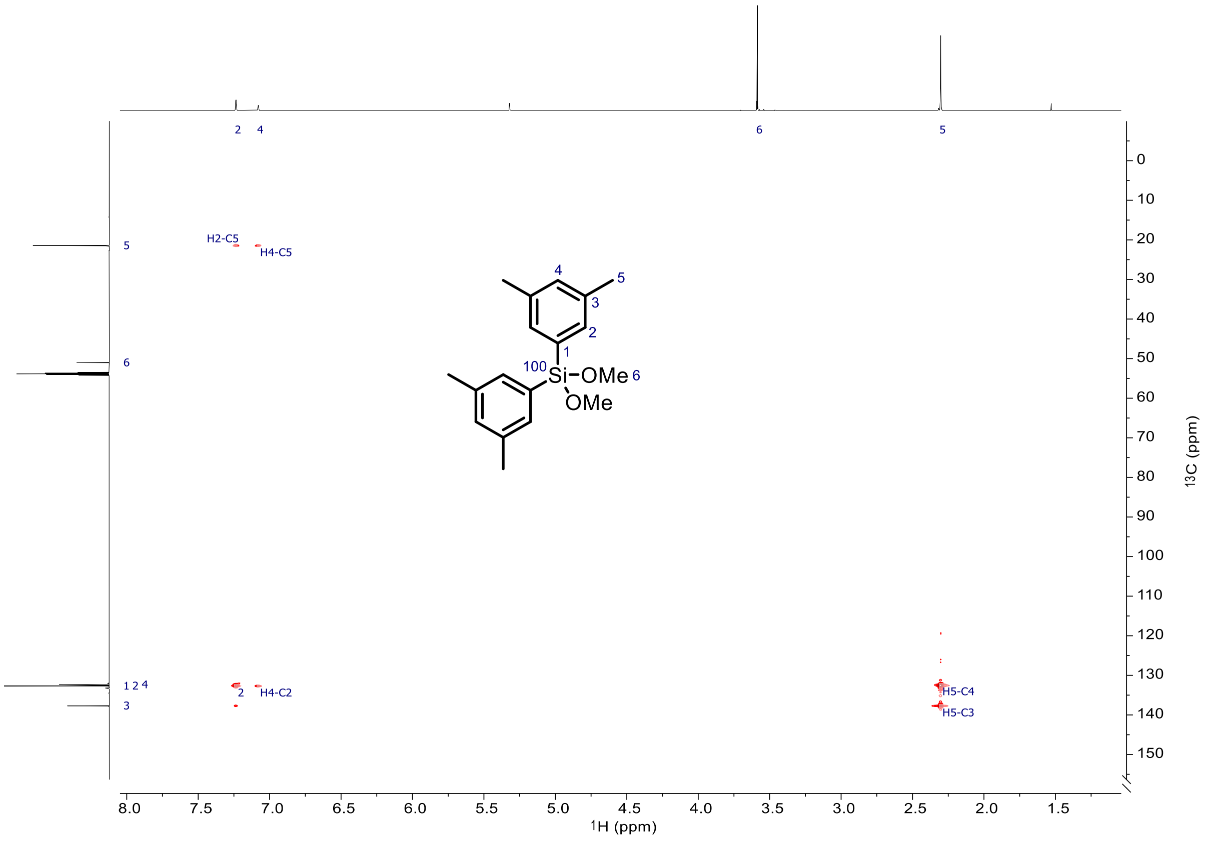

^{1}H-^{1}H COSY NMR spectrum of Bis(3,5-dimethylphenyl)dimethoxysilane (S1): CD_2Cl_2, 298 K, 600 MHz, 600 MHz.

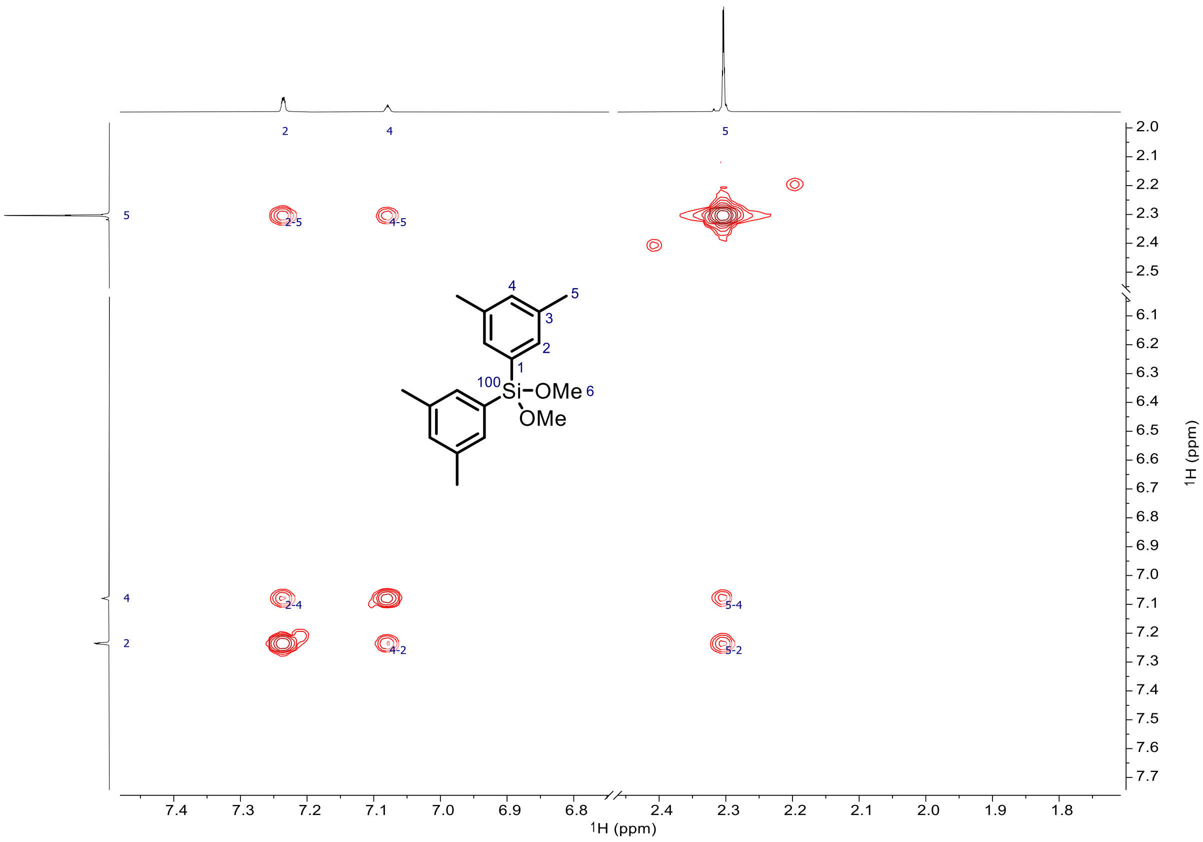

^{29}Si NMR spectrum (CD_2Cl_2, 298 K, 119 MHz) and **^{1}H-^{29}Si HMBC NMR spectrum of Bis(3,5-dimethylphenyl)dimethoxysilane (S1):** CD_2Cl_2, 298 K, 600 MHz, 119 MHz.

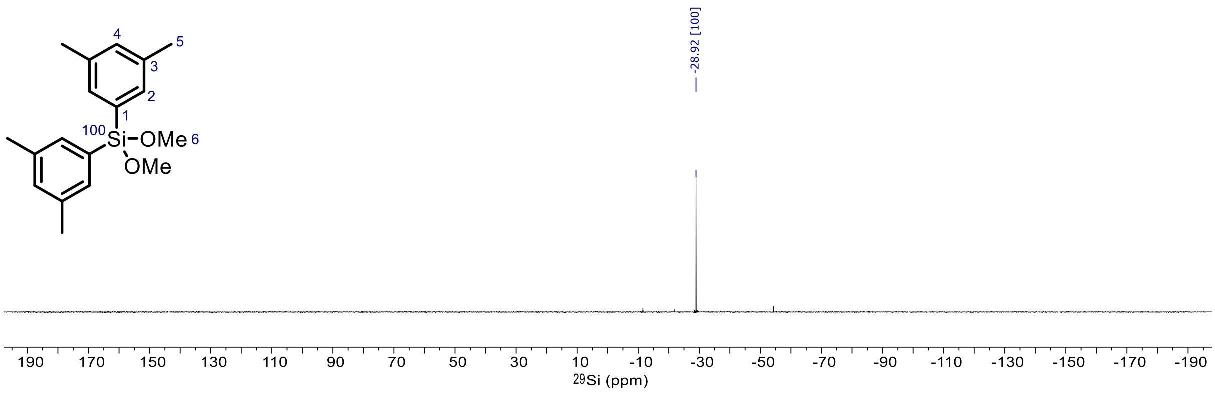

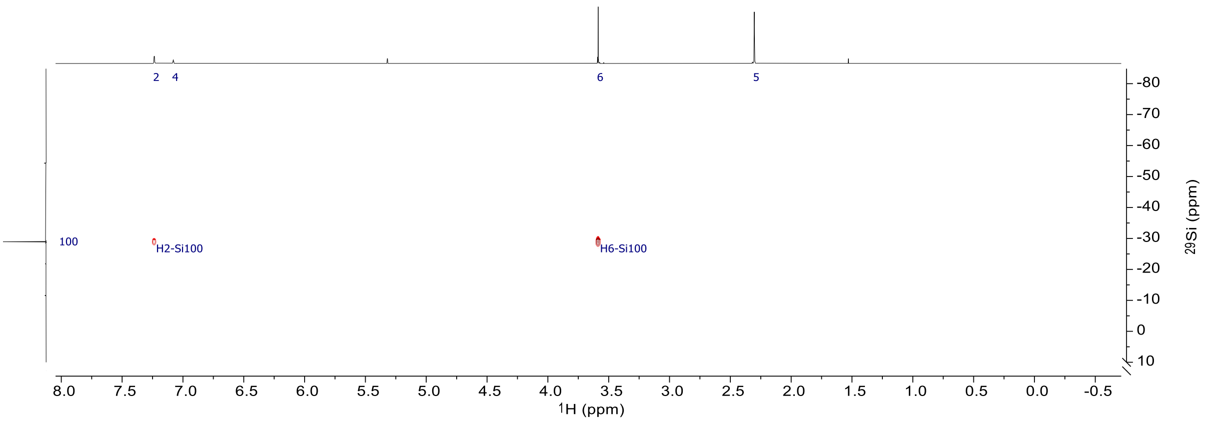

NMR Analysis of Ligand 9b. NMR analysis indicates that this ligand is C_3 symmetric on the NMR timescale at 298 K. It was not possible to assign C9, presumably because it is overlapping with C10, C11 or C12 and does not show a cross peak in the ^{1}H-^{13}C HMBC NMR spectrum.

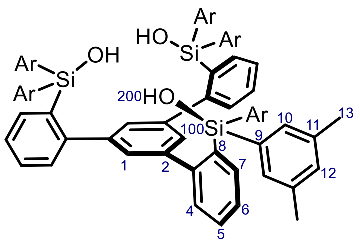

^{1}H NMR spectrum of ligand 9b: CD_2Cl_2, 298 K, 600 MHz.

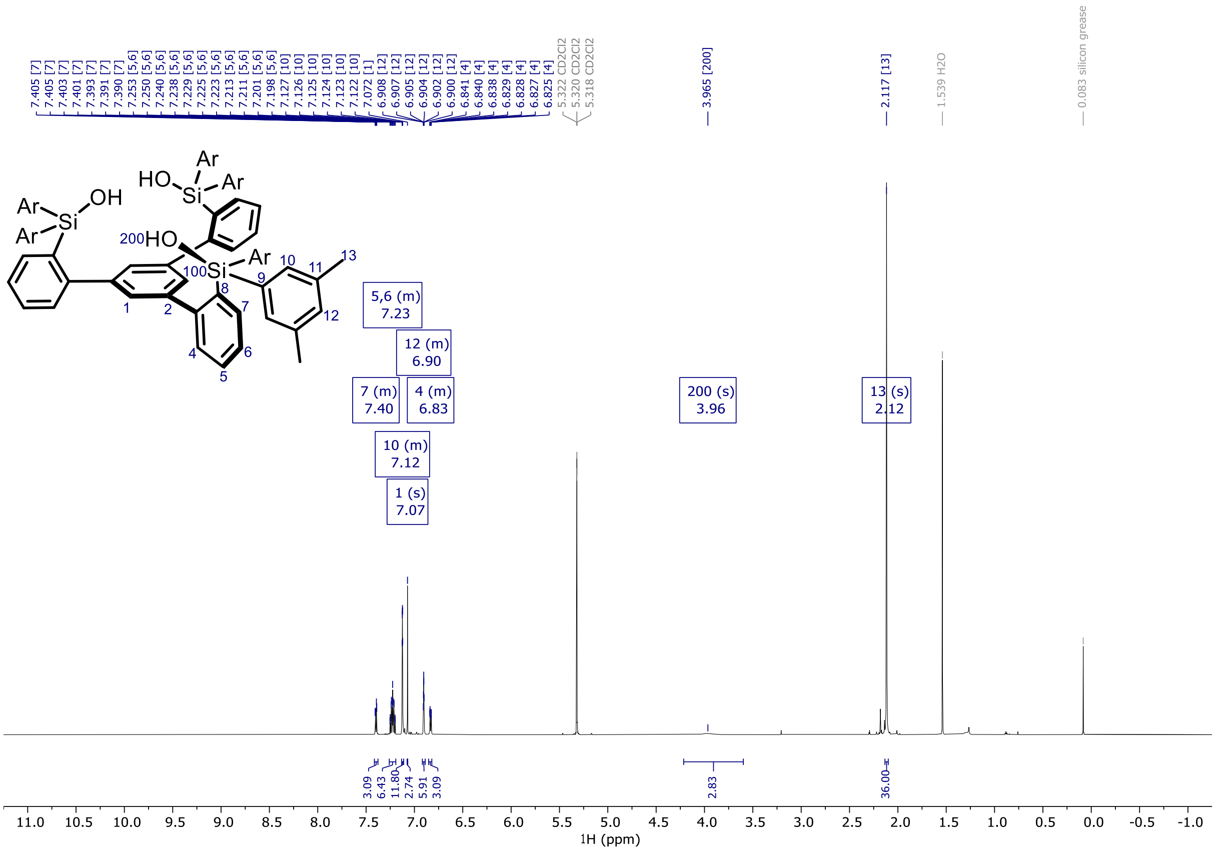

^{13}C NMR spectrum of ligand 9b: CD_2Cl_2, 298 K, 151 MHz.

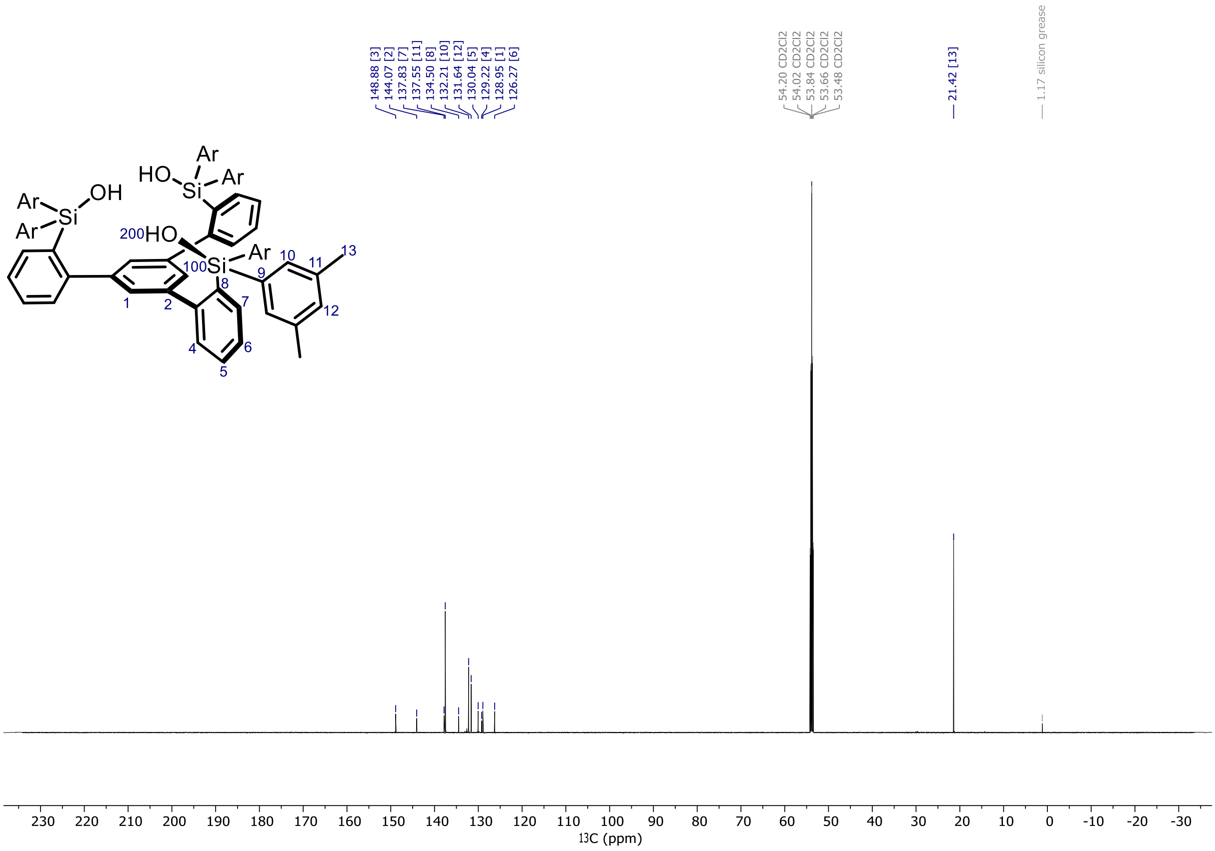

^{1}H-^{13}C HSQC NMR spectrum of ligand 9b: CD_2Cl_2, 298 K, 600 MHz, 151 MHz.

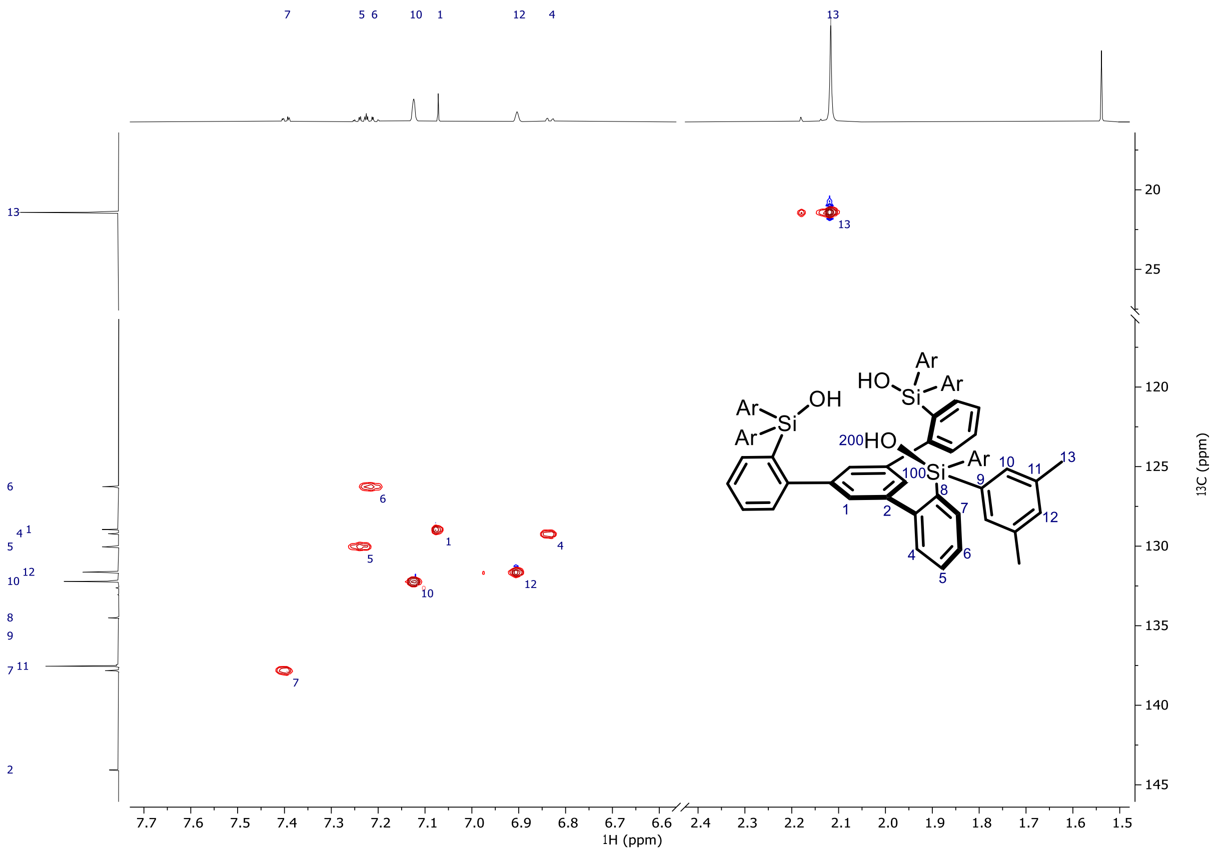

^{1}H-^{13}C HMBC NMR spectrum of ligand 9b: CD_2Cl_2, 298 K, 600 MHz, 151 MHz.

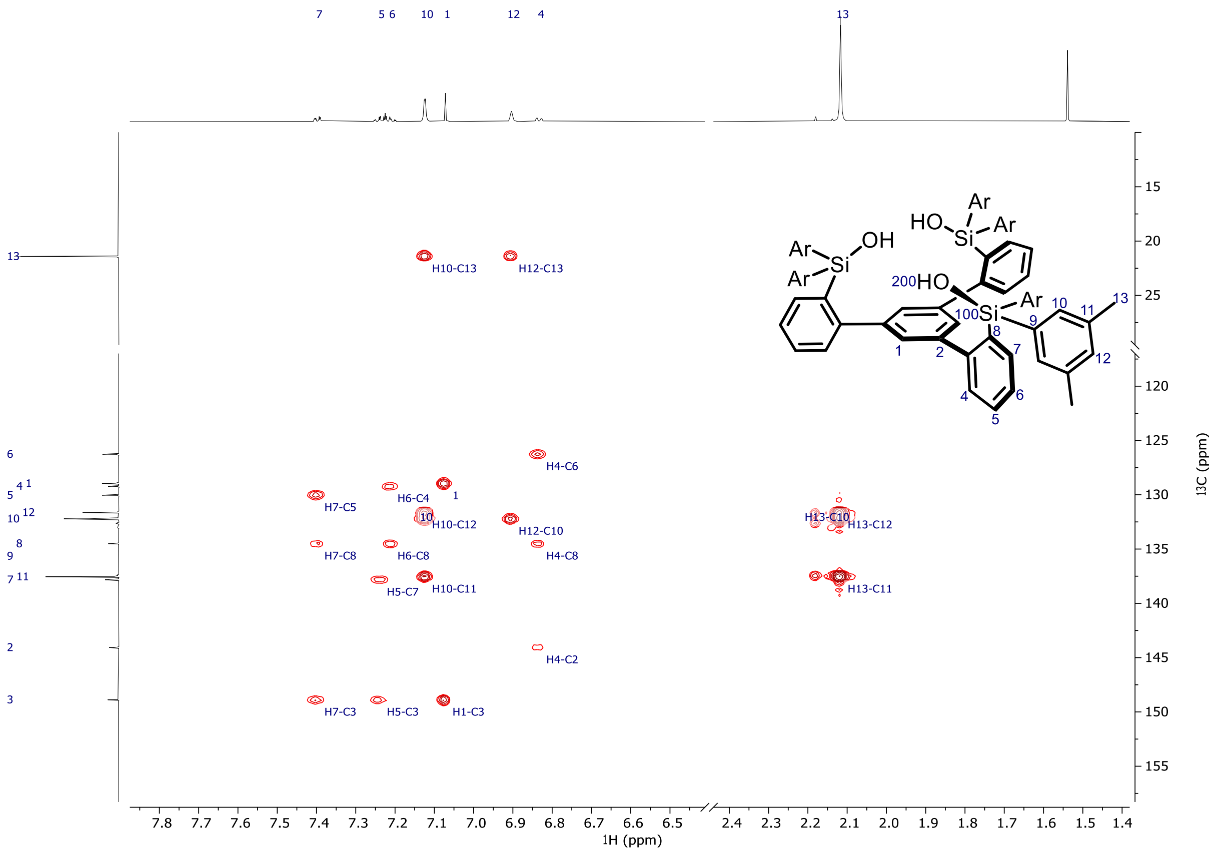

^{1}H-^{1}H COSY NMR spectrum of ligand 9b: CD_2Cl_2, 298 K, 600 MHz, 600 MHz.

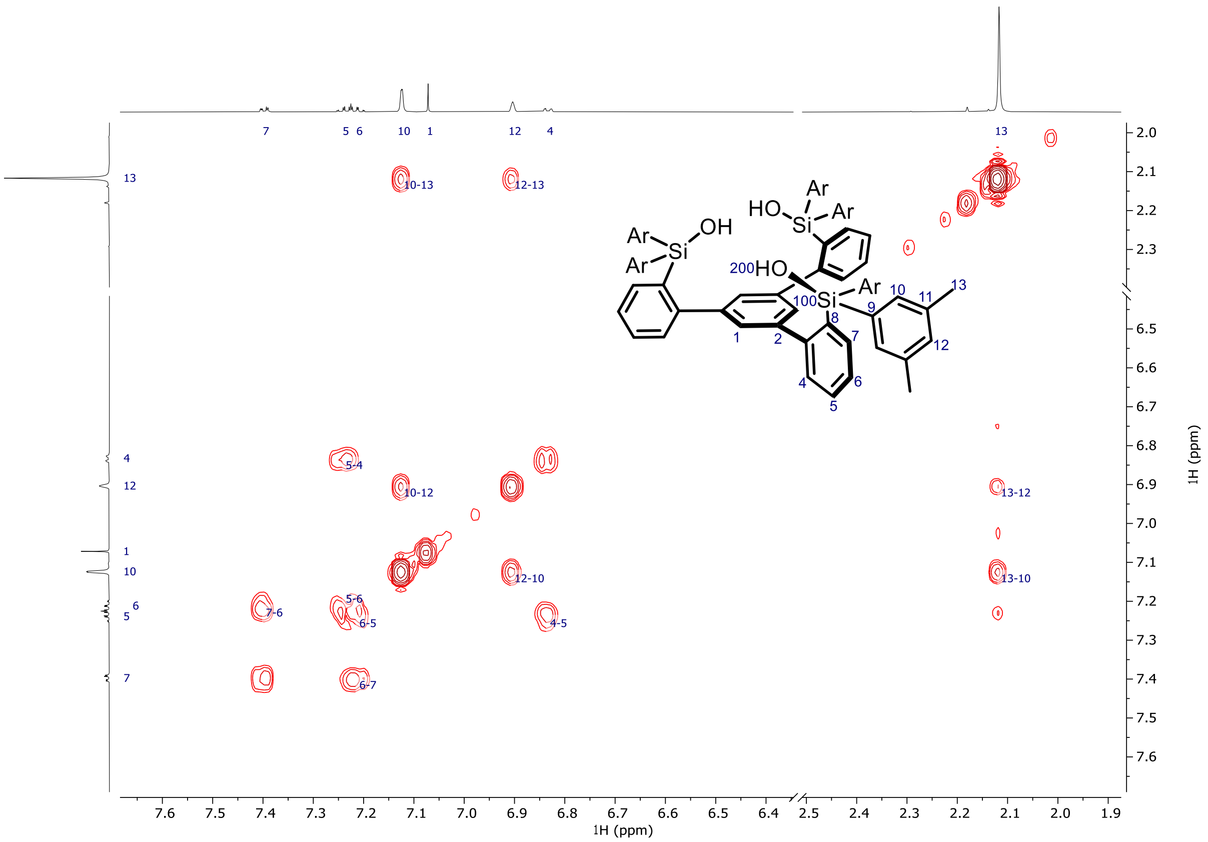

^{29}Si NMR spectrum of ligand 9b: CD_2Cl_2, 298 K, 119 MHz.

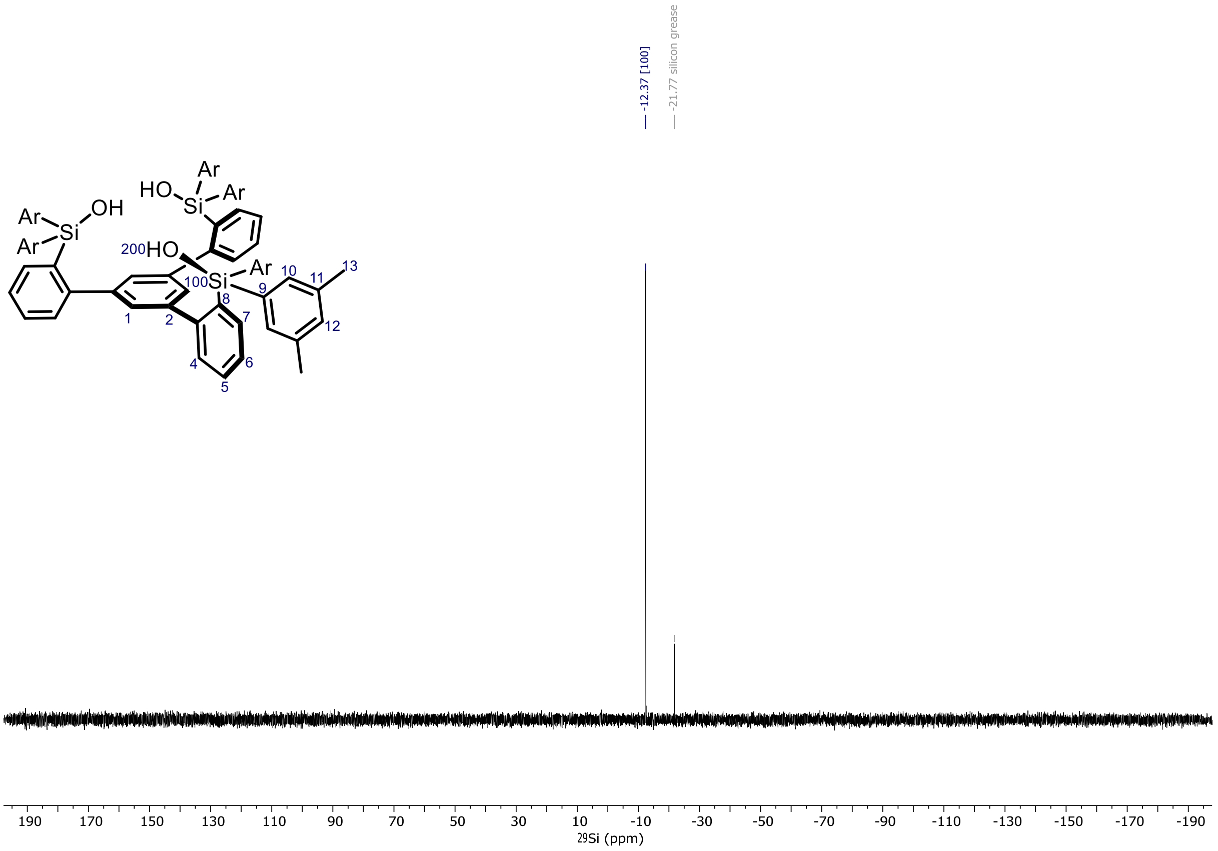

^{1}H-^{29}Si HMBC NMR spectrum of ligand 9b: CD_2Cl_2, 298 K, 600 MHz, 119 MHz.

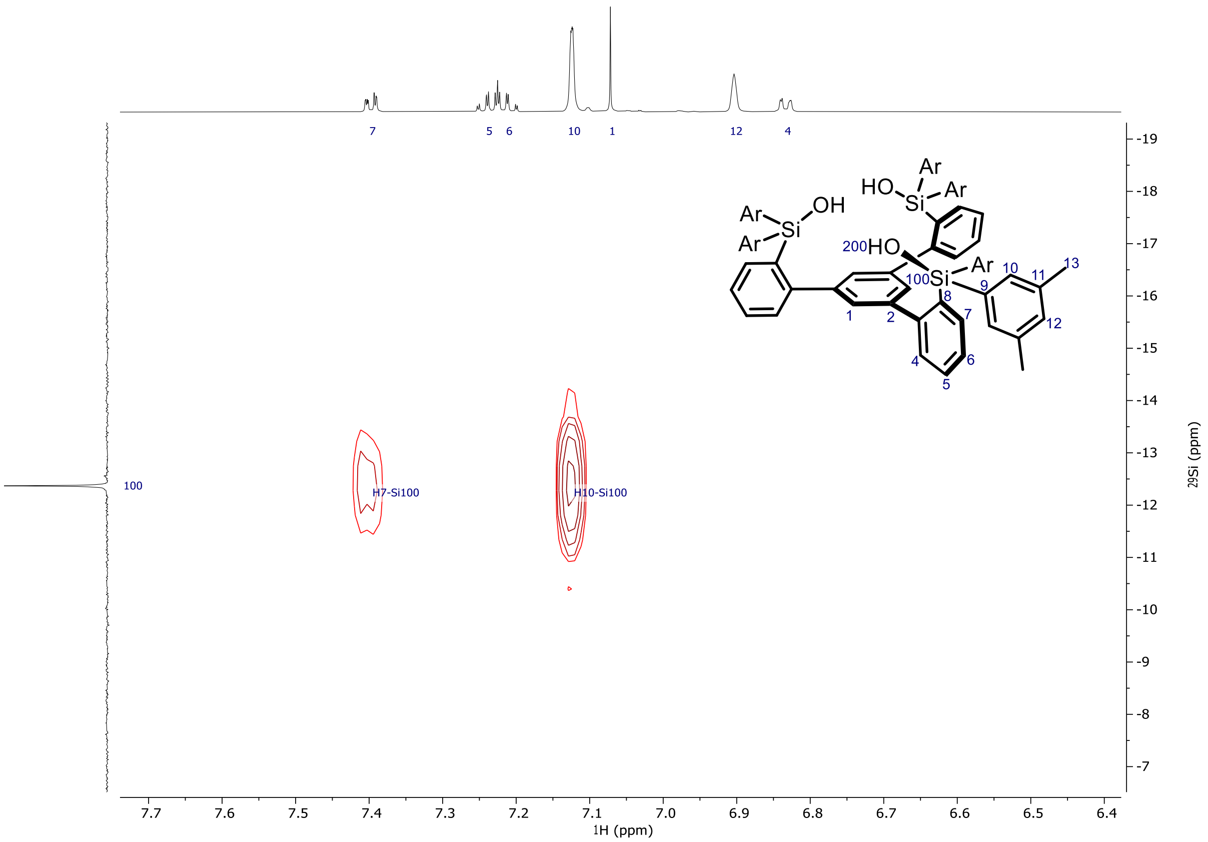

NMR Analysis of Complex 14. Most signals are sharp at 298 K. However, the signals of 15, 15', 16 and 16' are broadened due to a hindered rotation around the Si100−C14 bond. In contrast, the signals of the second aryl substituent (10, 10’, 11, 11’) are diastereotopic but much sharper, which could indicate an even higher rotational barrier. H10↔H10' and H13↔H13' show EXSY crosspeaks in the ^{1}H-^{1}H ROESY NMR experiment, which indicates that rotation is occurring at 298 K. No rotation was observed at 298 K about the N200−C21 bond as EXSY cross peaks are missing. Likely because of the problems caused by the quadrupolar nature of the ^{95}Mo nucleus, only one broad ^{95}Mo NMR signal could be observed. On the NMR timescale, the complex has C_3 symmetry about the Mo≡Mo triple bond because only one signal set is observed for the ligands.

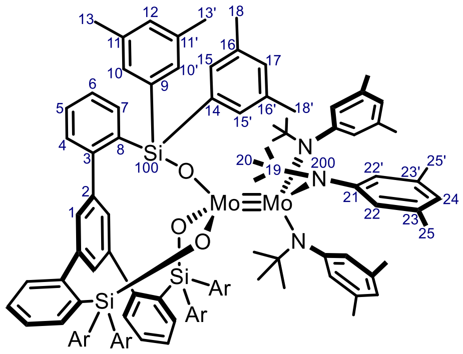

^{1}H NMR spectrum of complex 14: [D$_6$]-benzene, 298 K, 600 MHz.

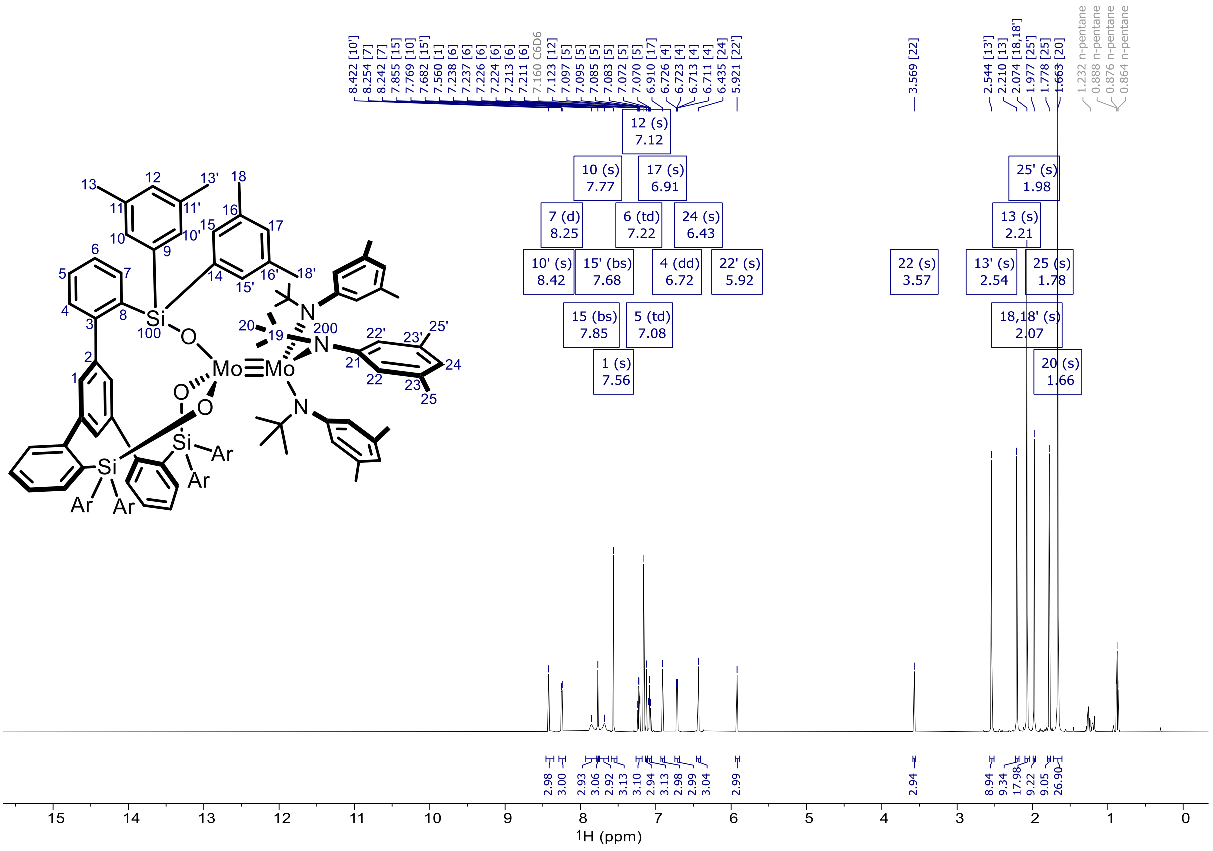

^{13}C NMR spectrum of complex 14: [D$_6$]-benzene, 298 K, 151 MHz.

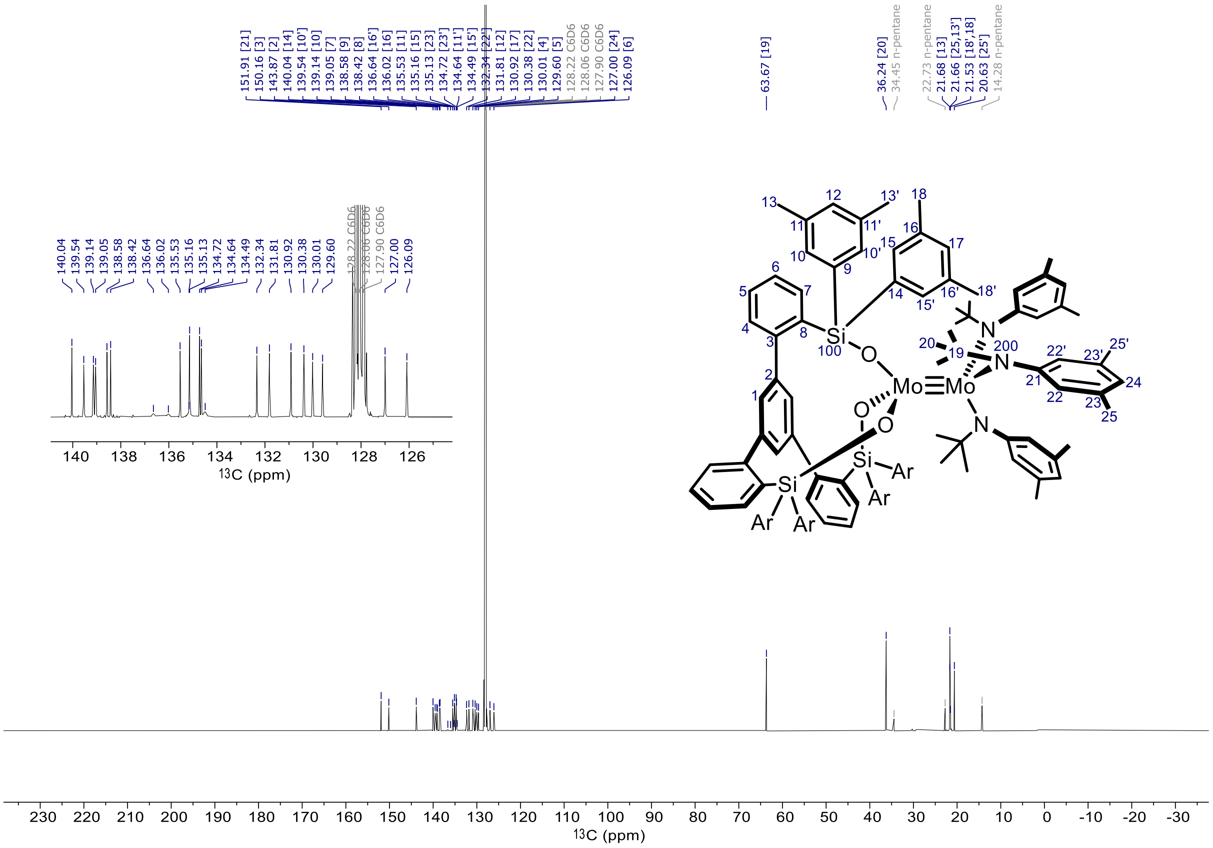

^{1}H-^{13}C HSQC NMR spectrum of complex 14: [D$_6$]-benzene, 298 K, 600 MHz, 151 MHz.

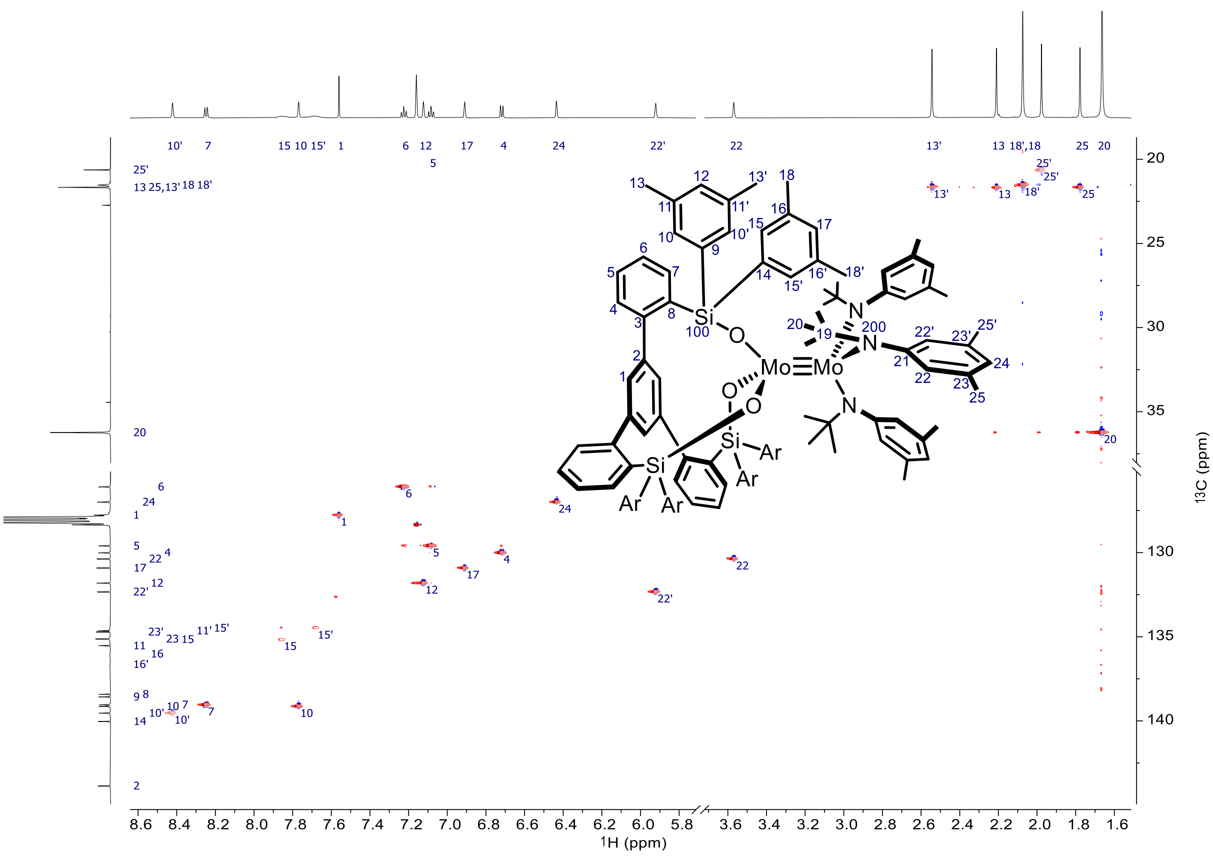

^{1}H-^{13}C HMBC NMR spectrum of complex 14: [D$_6$]-benzene, 298 K, 600 MHz, 151 MHz.

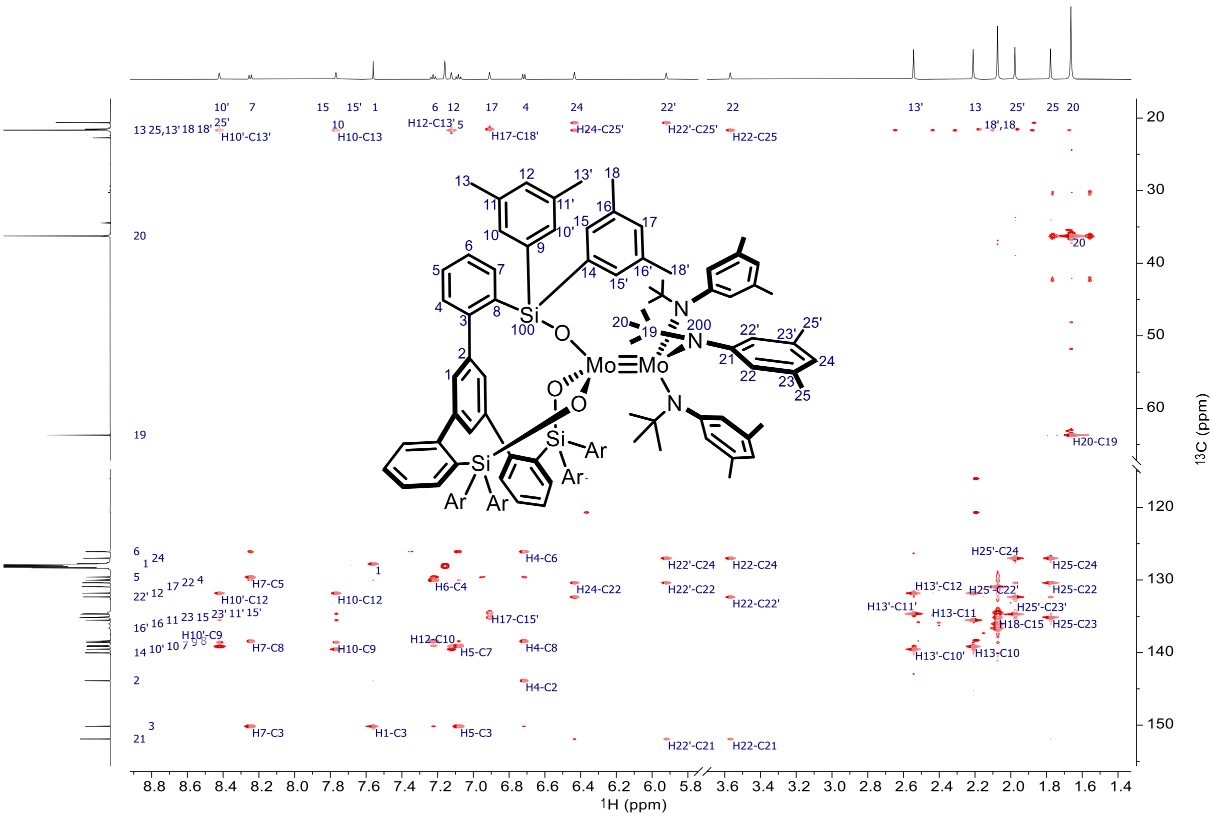

^{1}H-^{1}H COSY NMR spectrum of complex 14: [D$_6$]-benzene, 298 K, 600 MHz, 600 MHz.

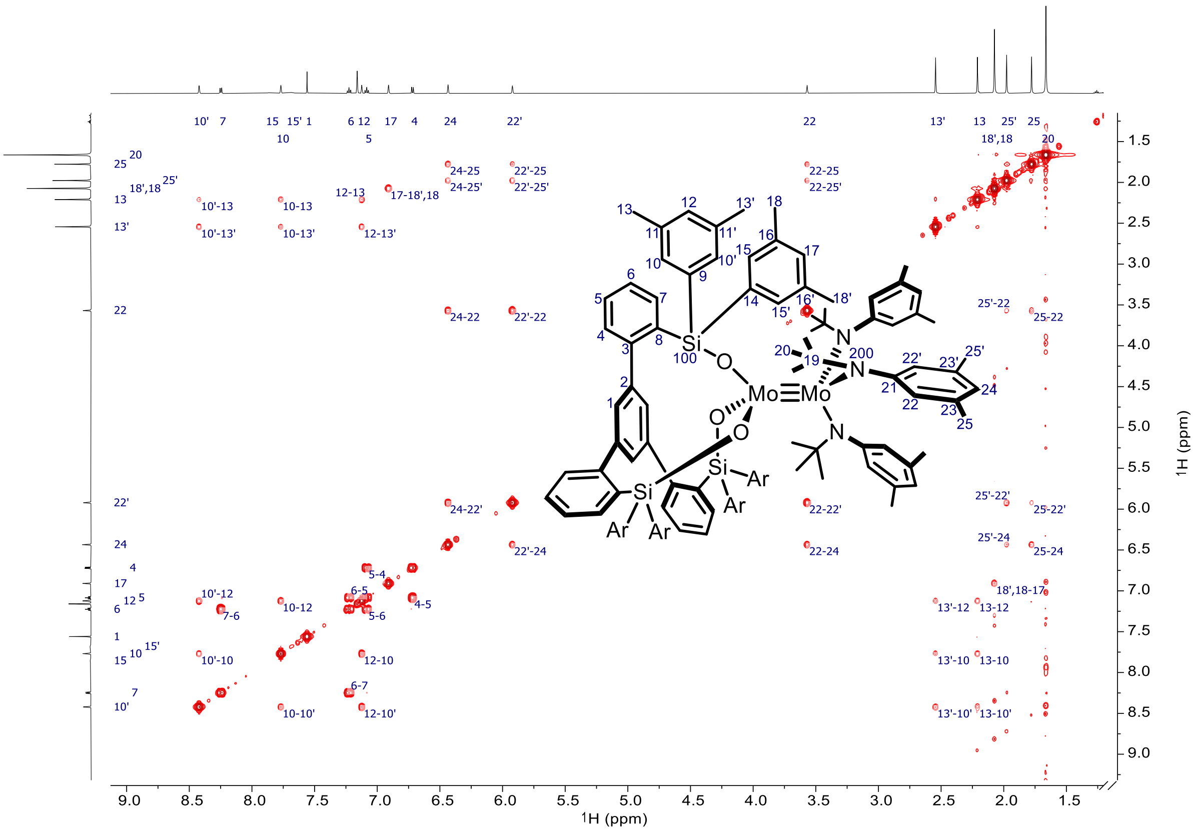

^{1}H-^{1}H ROESY NMR spectrum of complex 14: [D$_6$]-benzene, 298 K, 600 MHz, 600 MHz.

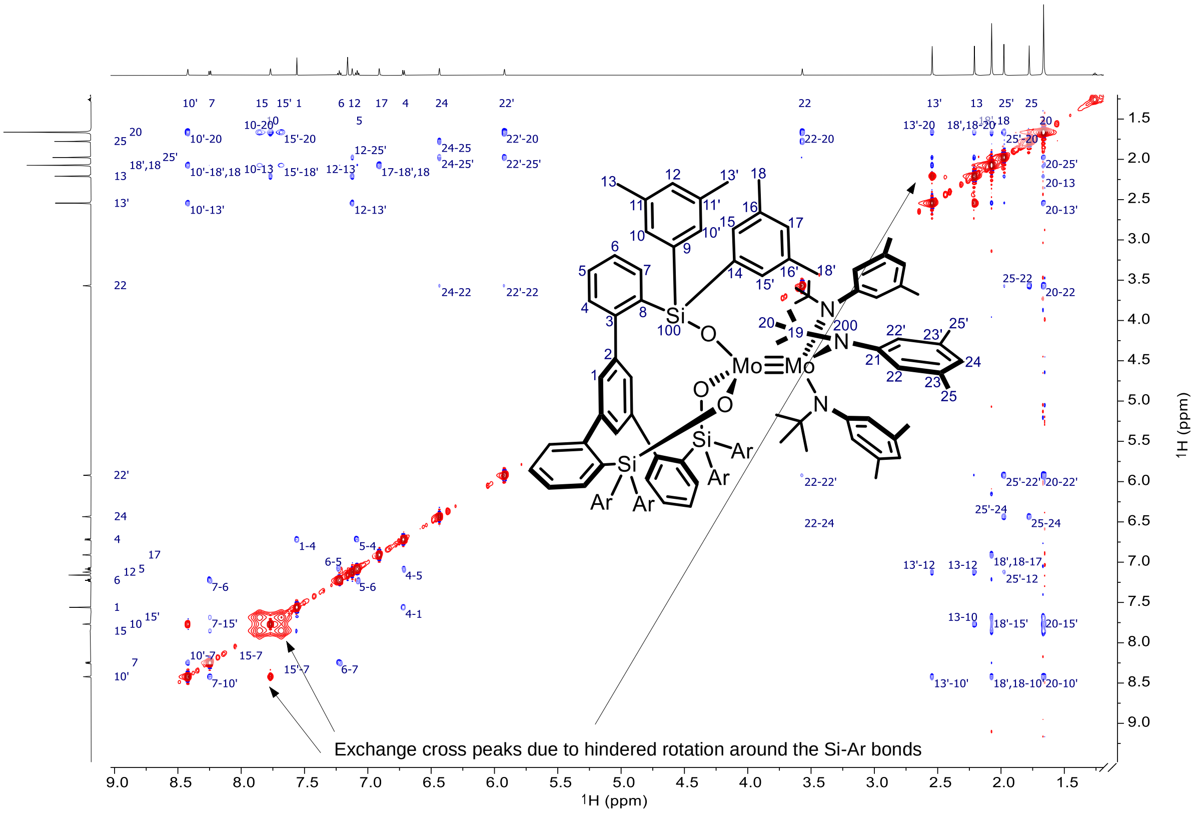

^{29}Si NMR spectrum ([D$_6$]-benzene, 298 K, 119 MHz) and **^{1}H-^{29}Si HMBC NMR spectrum of complex 14:** [D$_6$]-benzene, 298 K, 600 MHz, 119 MHz.

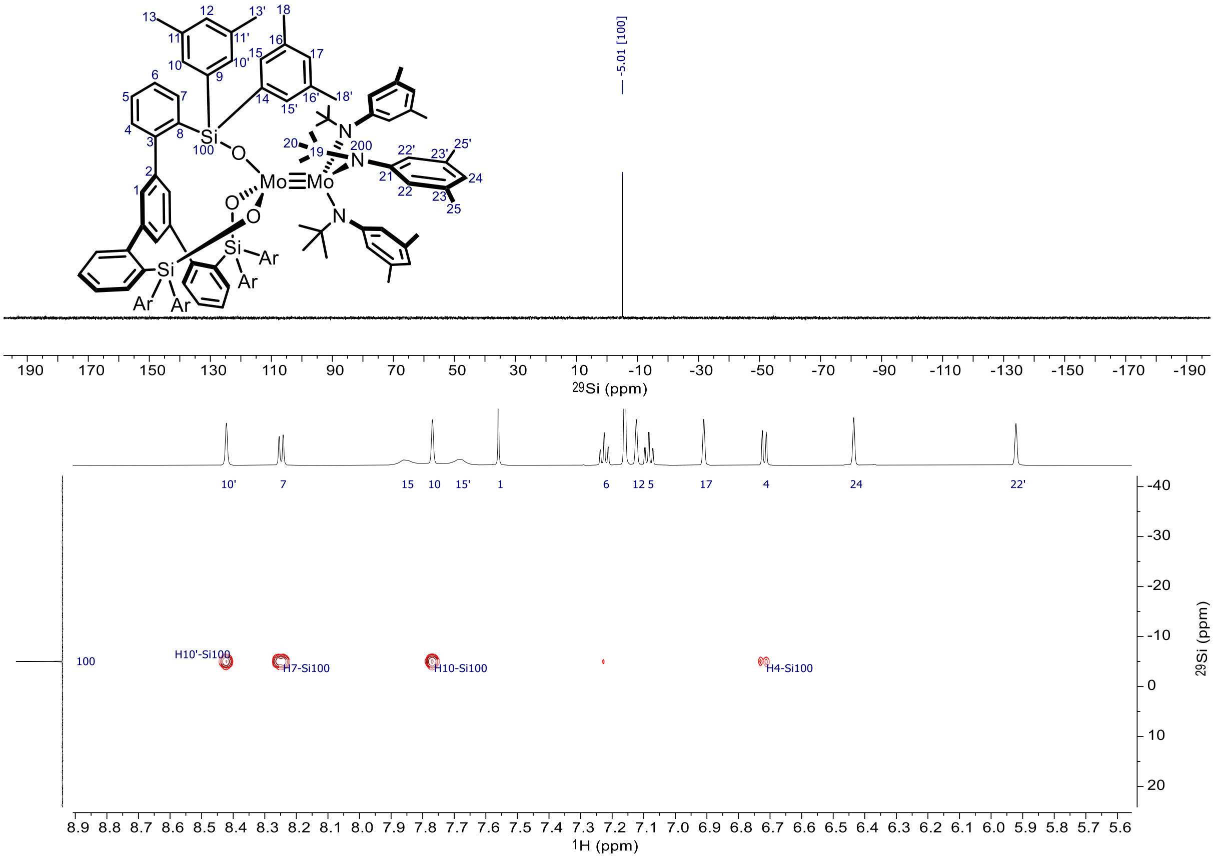

^{1}H-^{15}N HMBC NMR spectrum of complex 14: [D$_6$]-benzene, 298 K, 600 MHz, 61 MHz.

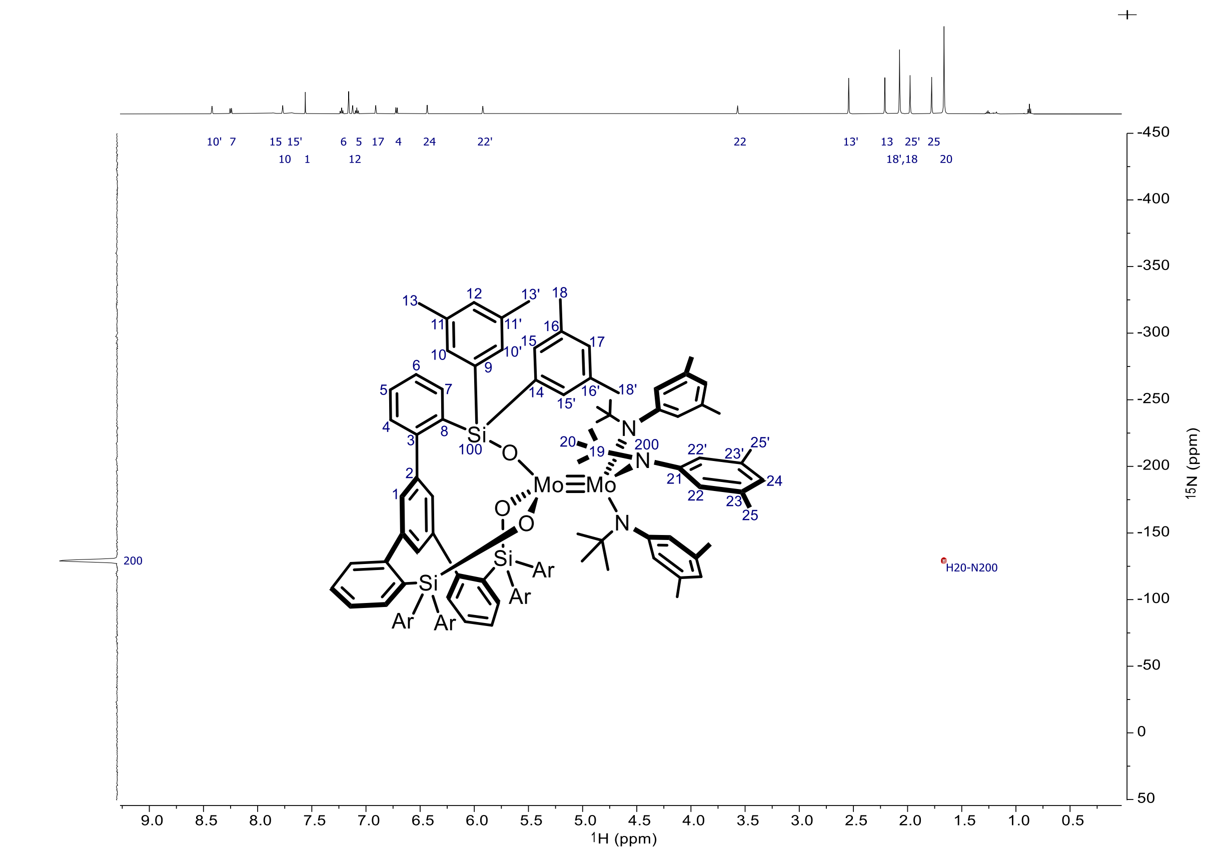

^{95}Mo NMR spectrum of complex 14: [D$_8$]-toluene, 333 K, 26 MHz.

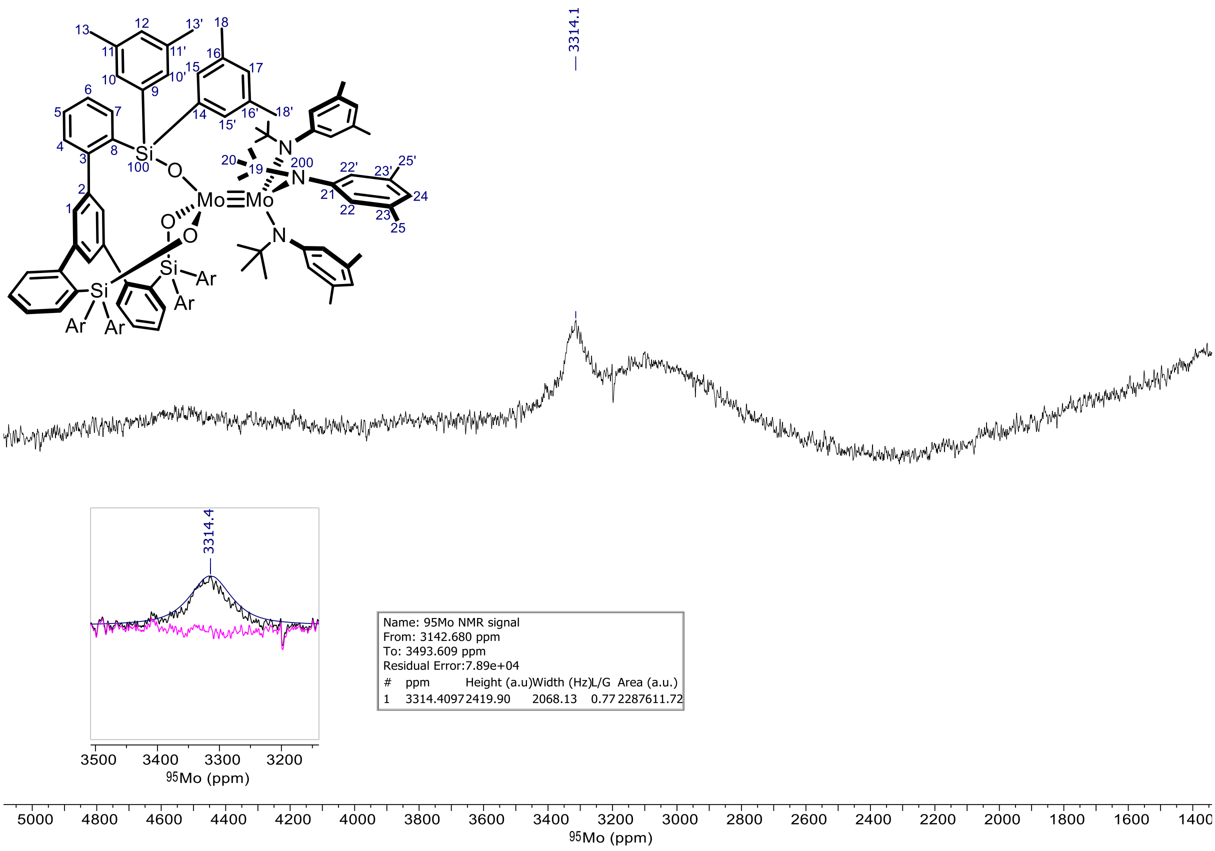

^{1}H NMR spectra of complex 14: [D$_6$]-benzene, 283 K−333 K, 600 MHz.

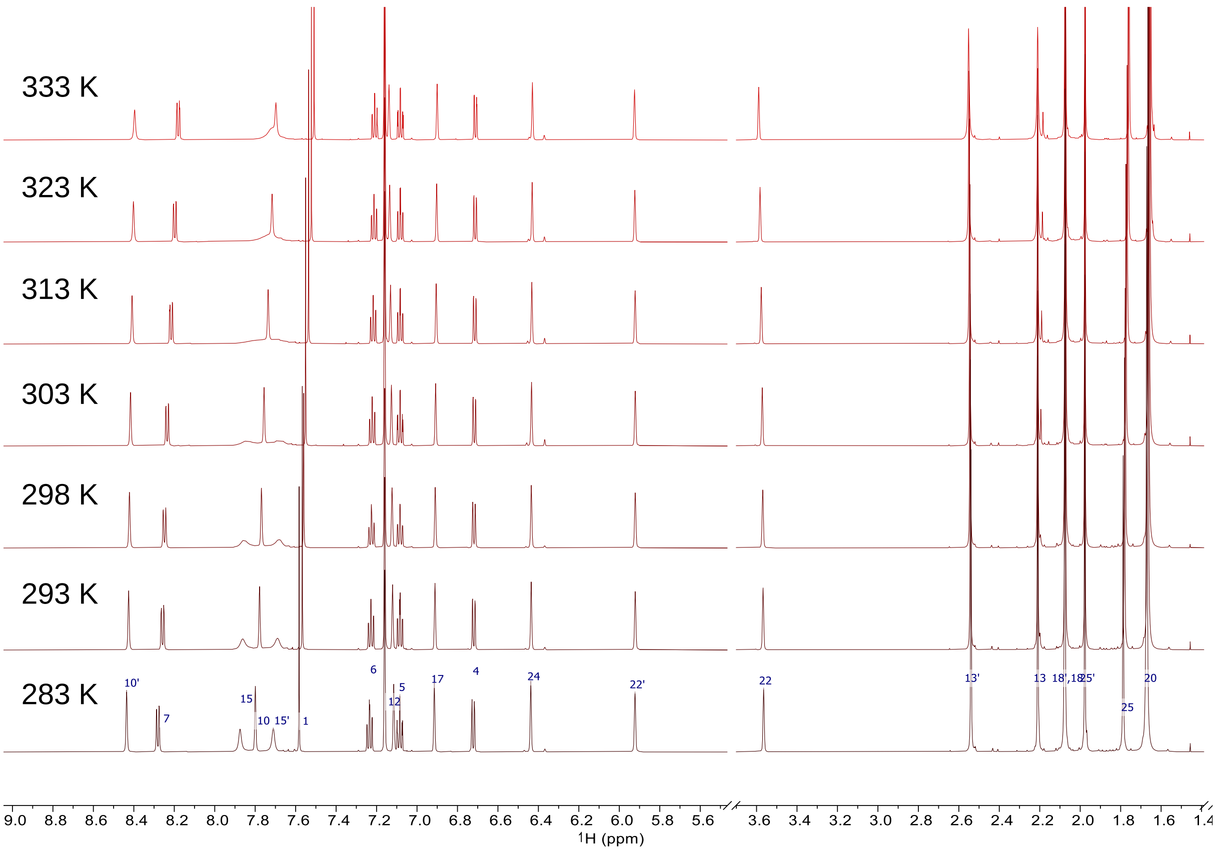

NMR Analysis of Complex 16. The ^{15}N NMR shift (−147 ppm) is significantly different from the shift of the free aniline (−289 ppm), which indicates ligation to the Mo(III) atom. Furthermore, ROESY cross peaks can be found which confirm that the two different ligand spheres are part of the same molecule. This is also supported by the ^{1}H NMR integrals (1:1). Further experiments were conducted to determine whether a high rotational barrier around the nitrogen-aryl bond of the amide ligands causes the diastereotopicity of H9', H9", H11, H12, H16, H20, H21, and H22. Based on a temperature-dependent series of measurements (233 K−298 K) this assumption was verified in view of a line sharpening of all diastereotopic protons (H9', H9", H11, H12, H16, H20, H21 and H22) with decreasing temperature. This can also be seen by EXSY cross peaks (H20↔H16) in the ^{1}H-^{1}H NMR ROESY spectrum. This hindered rotation likely causes the diastereotopicity of H9'/9" and H11/12, as EXSY cross peaks are observed for H9'↔H9" and H11↔H12. The signal of H16 is extremely shielded (4.19 ppm).

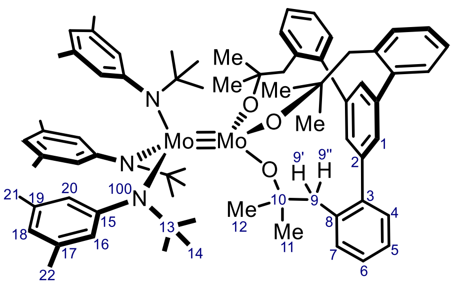

¹H NMR spectrum of complex 16: [D$_8$]-toluene, 298 K, 600 MHz.

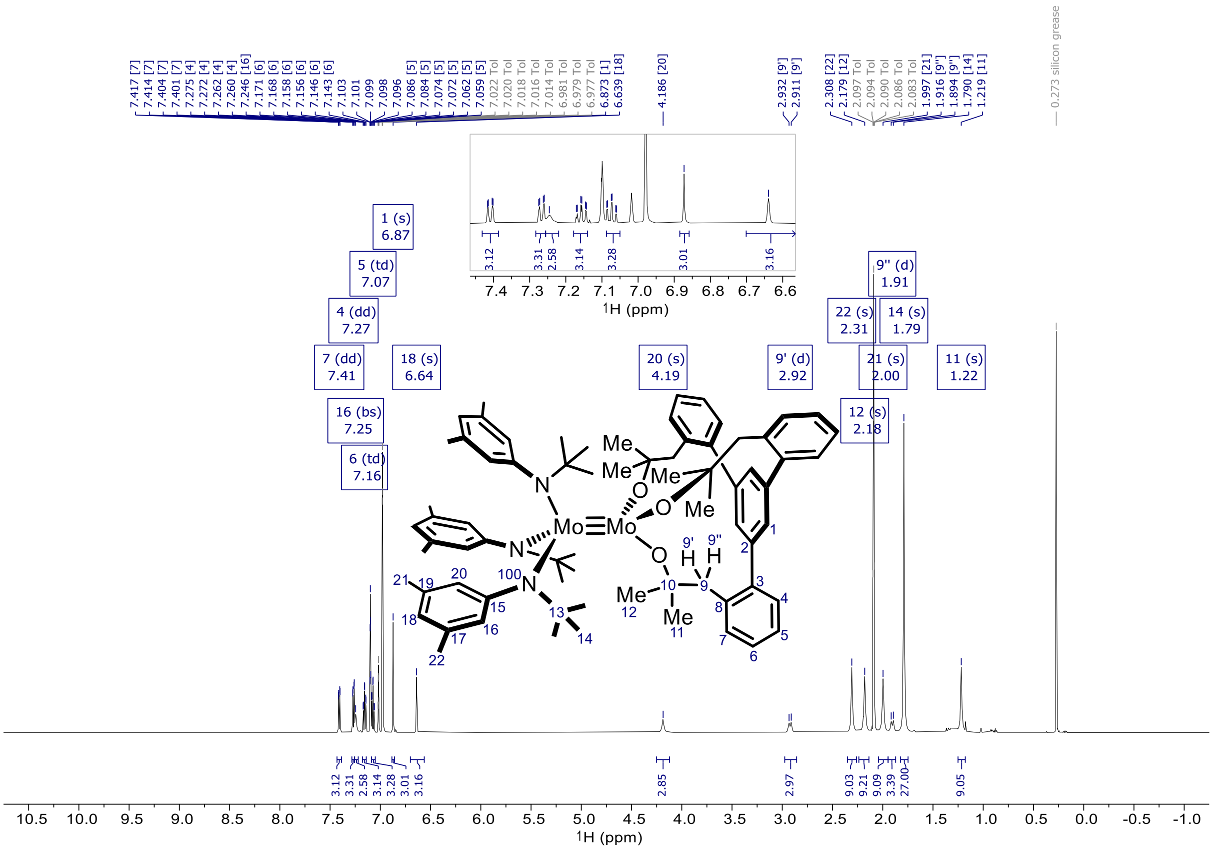

¹³C NMR spectrum of complex 16: [D$_8$]-toluene, 298 K, 151 MHz.

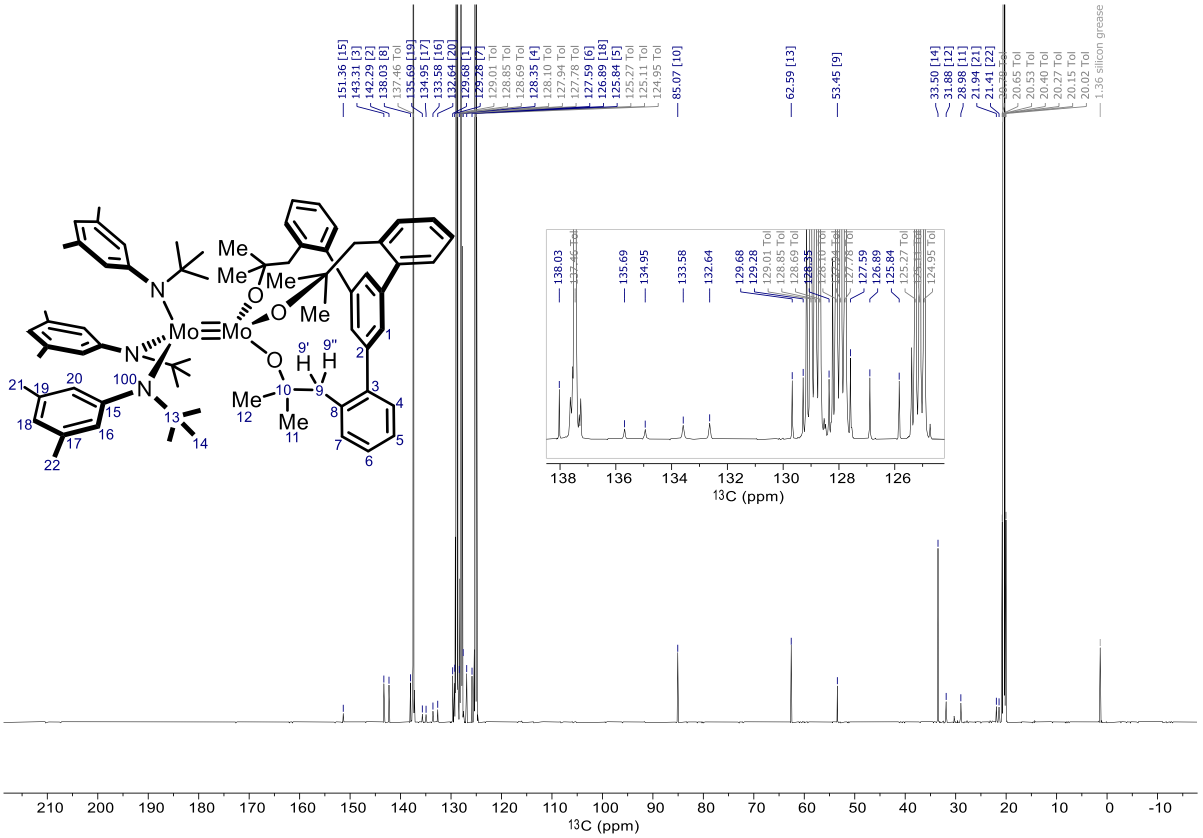

^{1}H-^{13}C HSQC NMR spectrum of complex 16: [D$_8$]-toluene, 298 K, 600 MHz, 151 MHz.

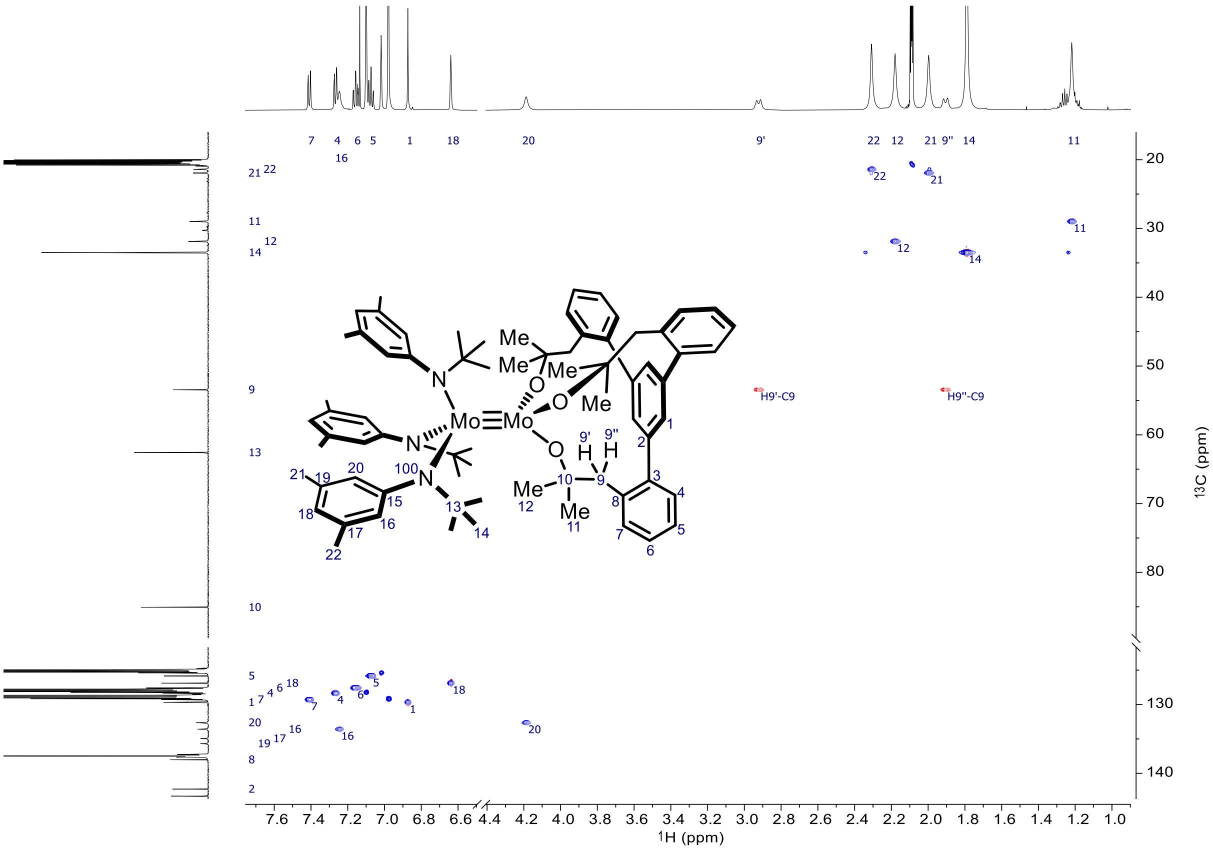

^{1}H-^{13}C HMBC NMR spectrum of complex 16: [D$_8$]-toluene, 298 K, 600 MHz, 151 MHz.

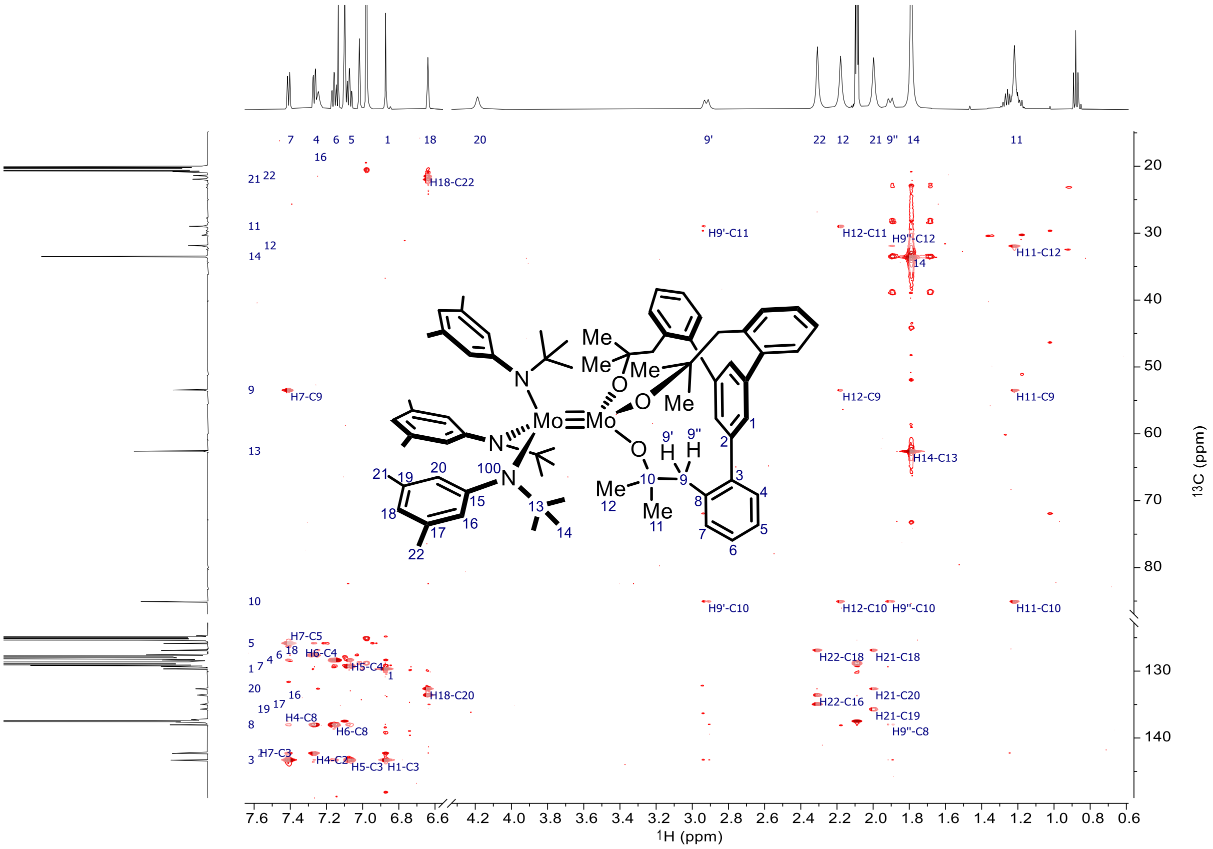

^{1}H-^{1}H COSY NMR spectrum of complex 16: [D_8]-toluene, 298 K, 600 MHz, 600 MHz.

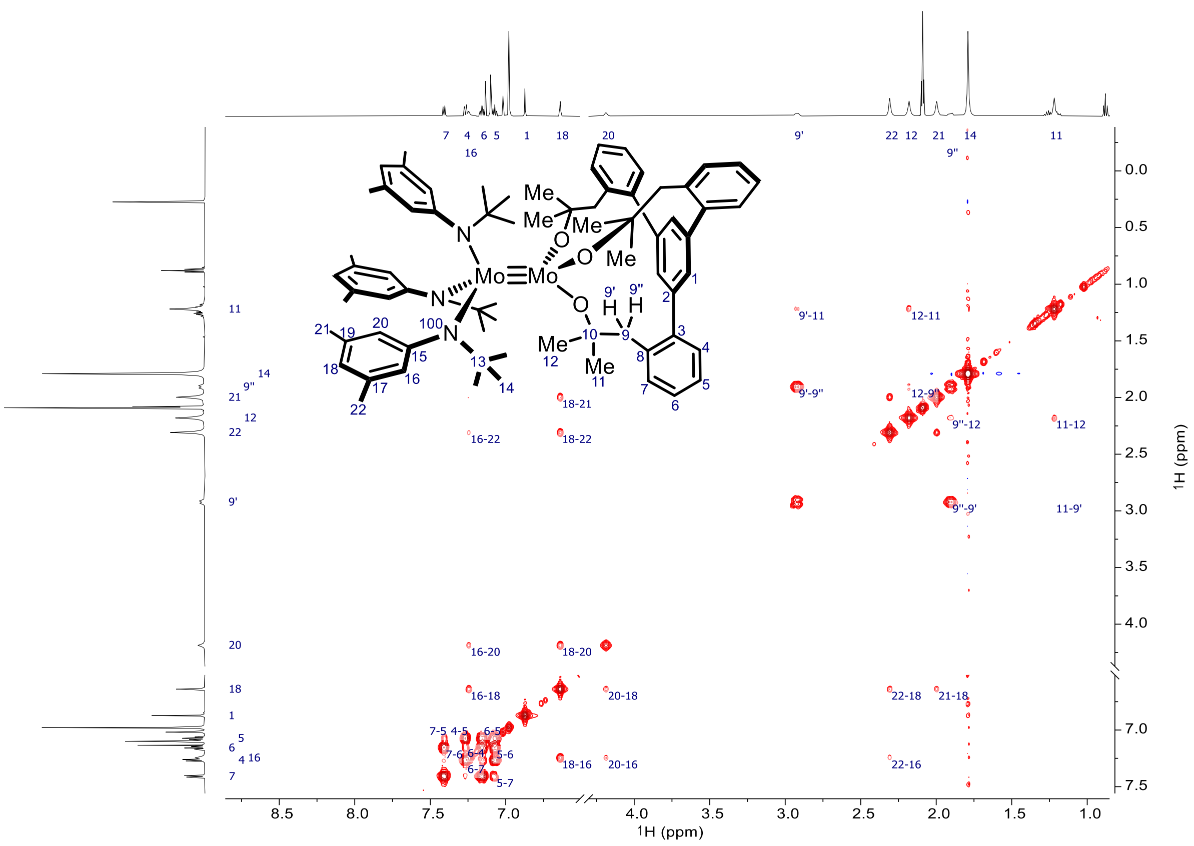

^{1}H-^{1}H ROESY NMR spectrum of complex 16: [D_8]-toluene, 298 K, 600 MHz, 600 MHz.

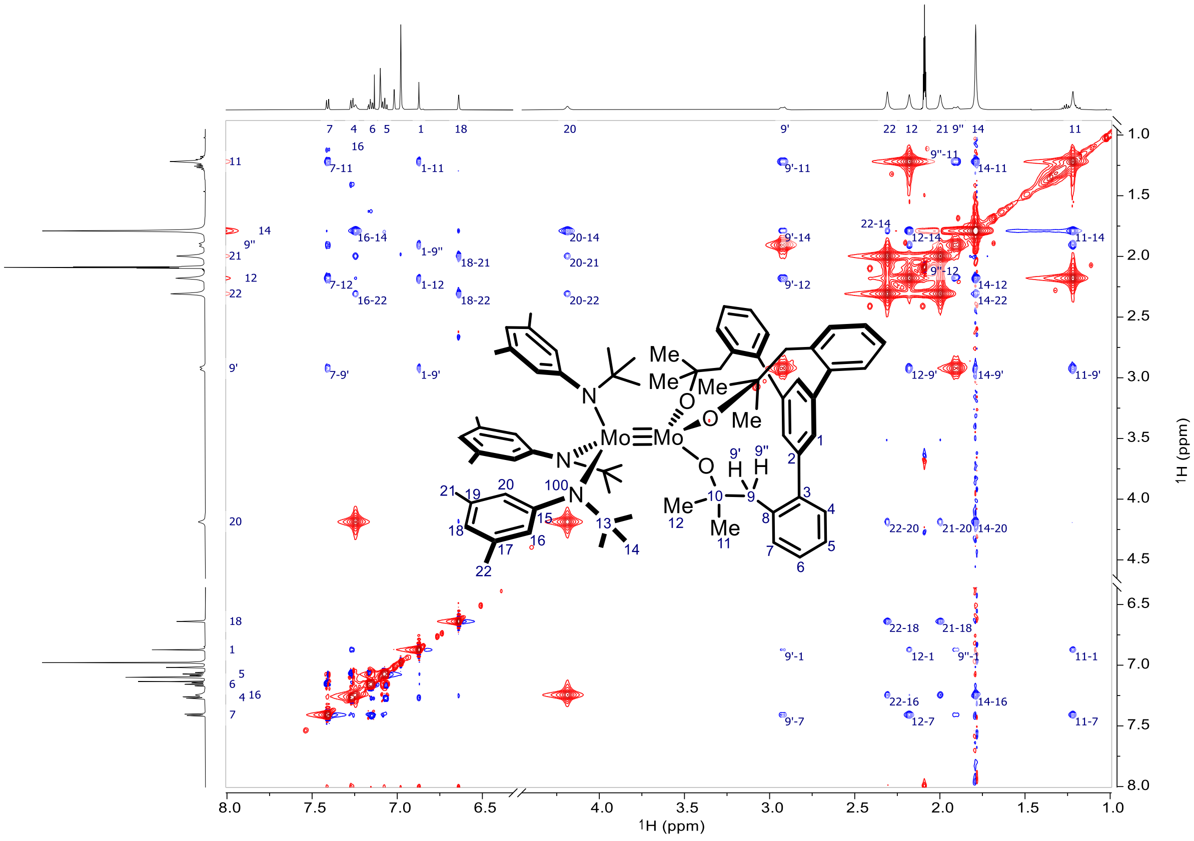

^{1}H-^{15}N HMBC NMR spectrum of complex 16: [D$_8$]-toluene, 298 K, 600 MHz, 61 MHz.

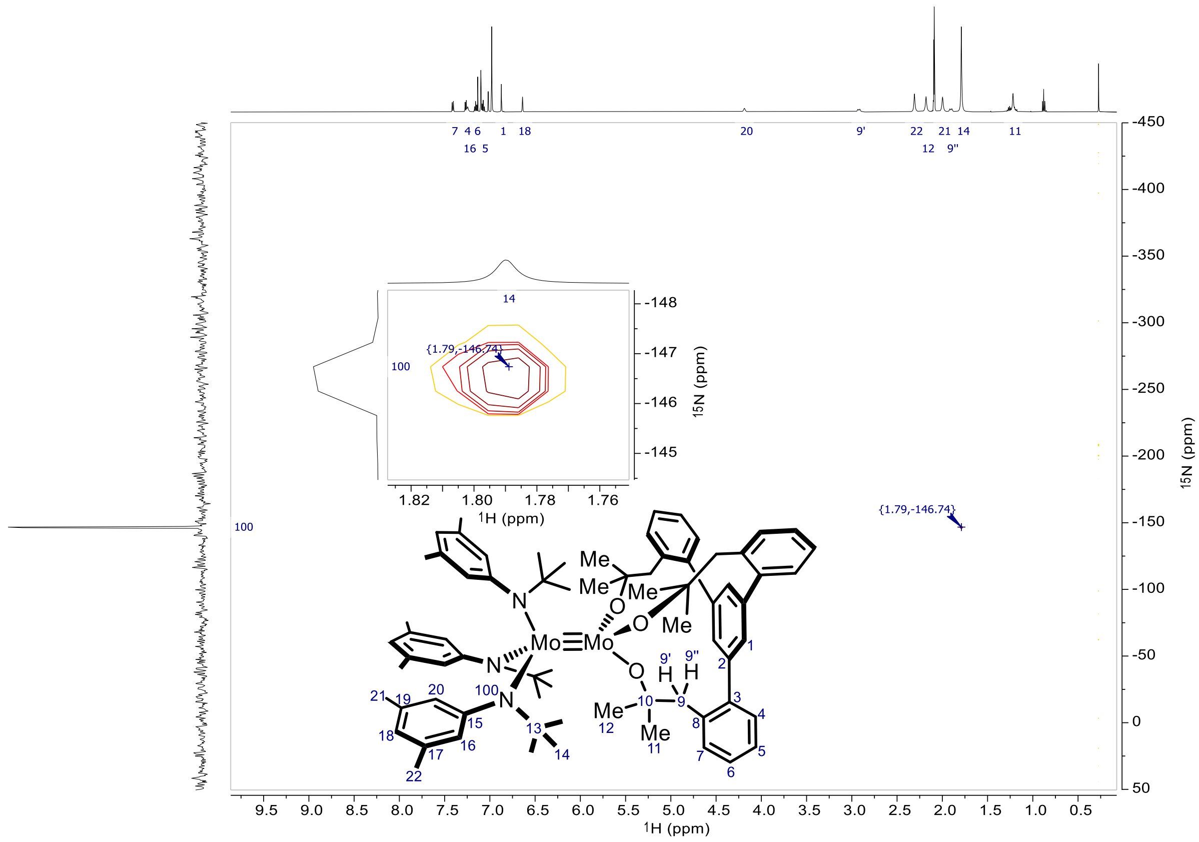

^{1}H NMR spectra of complex 16: [D$_8$]-toluene, 233 K−298 K, 600 MHz. Sharpening of the ^{1}H NMR signals indicates the reduced rotational exchange.

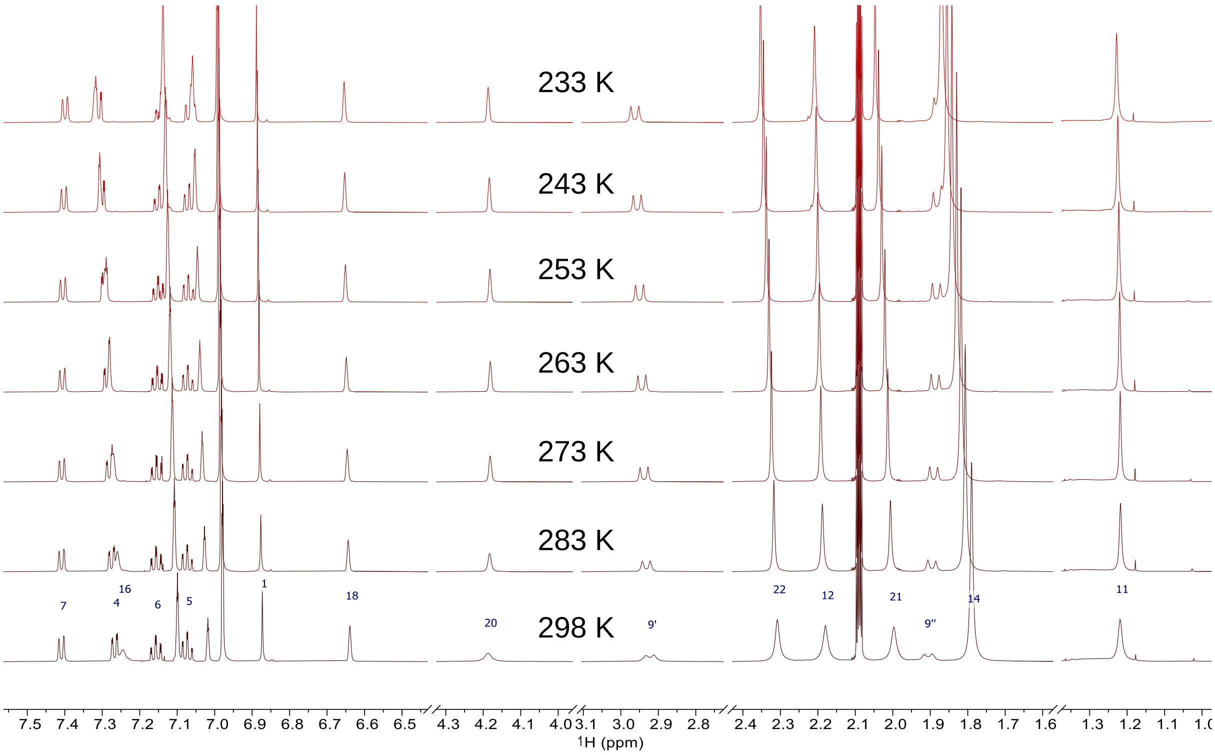

NMR Analysis of Complex 17. Correlations between the protons H4 and H2 in the ^{1}H-^{1}H ROESY NMR spectrum support that both independent ligand spheres are part of the same C_3 symmetrical molecule. At room temperature the N–Ar signals are broadened due to a hindered rotation around the N–Ar bond. Therefore, the compound was characterised at 233 K where most of the signals were sharp. The signal of H6 is strongly shielded compared to H8 and H6'. This observation is explained by the anisotropic ring current effect by a neighboured N–Ar ring; this notion is in excellent agreement with the particular orientation that H6 adopts in the solid state as shown by single crystal X-ray diffraction (Figure S7). The coalescence temperature of the H6-H6' exchange is at around 333 K, which allows us to estimate that the rotational barrier $\Delta G^{\ddagger}_{Tc}$ is ~12.5 kcal/mol. A ^{95}Mo NMR measurement at 333 K was performed successfully. The signals (ratio 1:1) were tentatively assigned based on their relative line width (by comparison with the line widths of $Mo_2(OtBu)_6$ and $Mo_2(NMe_2)_6$ reported in the literature).[9] Both values are shifted by 600-800 ppm compared to the homodimeric complexes $Mo_2(OtBu)_6$ and $Mo_2(NMe_2)_6$, see Table 1 in the main Text. The complex is stable over several days at 333 K in [D_8]-toluene.

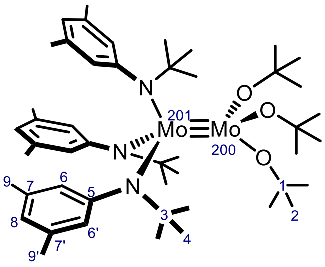

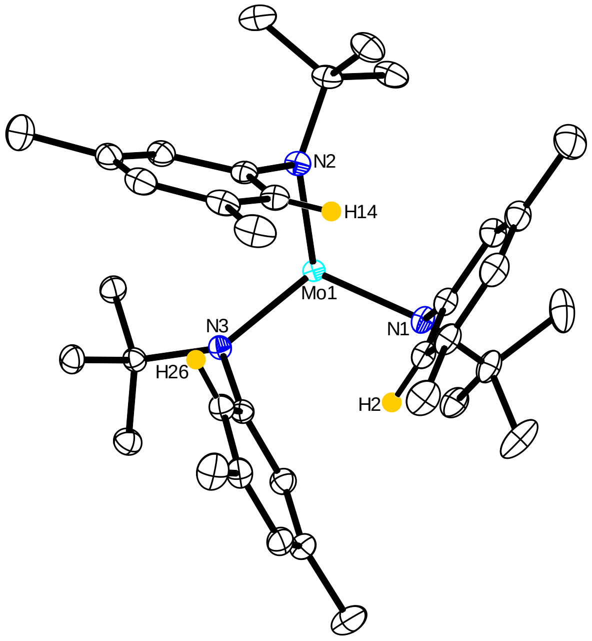

Figure S7. Side-view of the”Cummins fragment” comprised in the crystal structure of complex **17** (only one Mo(N(tBu)(Ar))$_3$ face is shown for clarity). The notion that the shift of the *ortho* protons H6 in the ^{1}H NMR spectrum of complex **17** (named H2, H14 and H26 in the single crystal structure and highlighted in yellow) towards a lower frequency is caused by the anisotropic ring current effect of a neighboured N–Ar ring is supported by the orientation of these protons in the molecular structure of **17** in the solid state.

^{1}H NMR spectrum of complex 17: [D$_8$]-toluene, 233 K, 600 MHz.

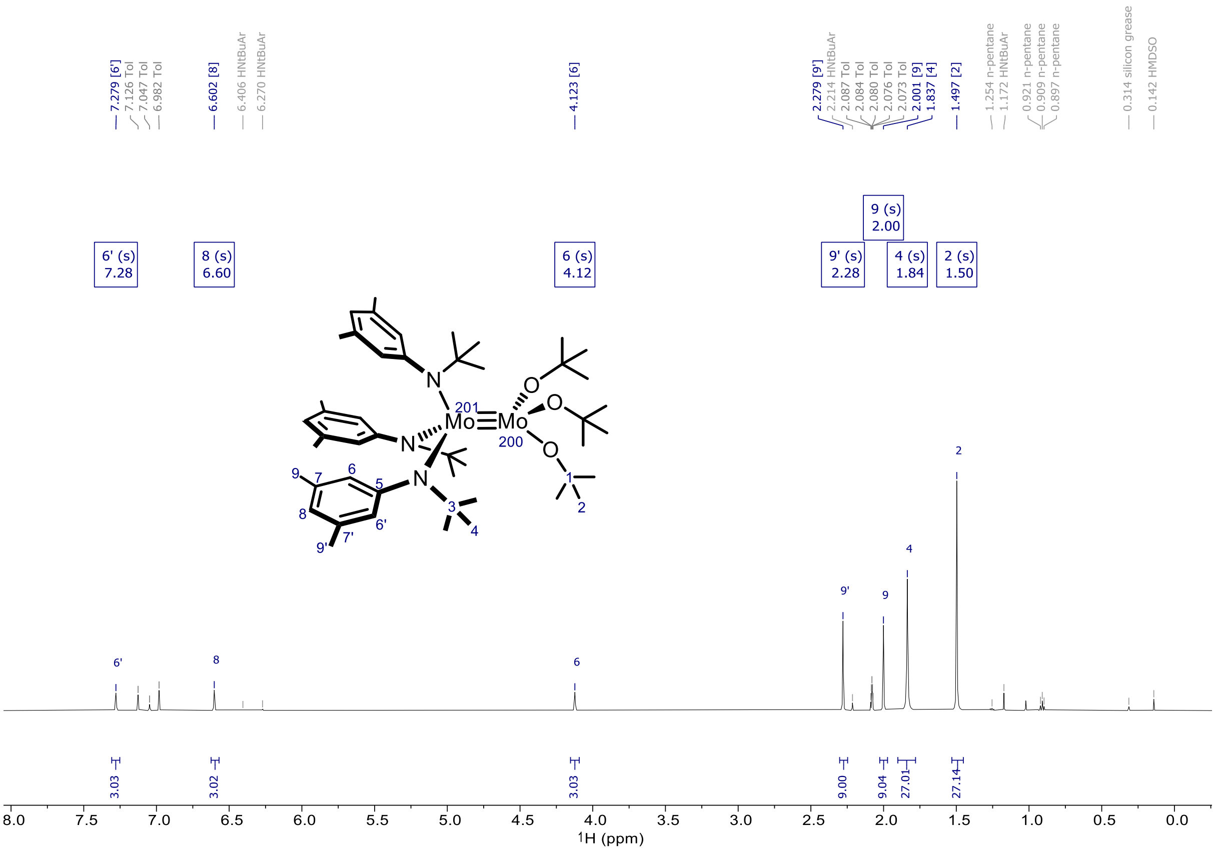

^{13}C NMR spectrum of complex 17: [D$_8$]-toluene, 233 K, 151 MHz.

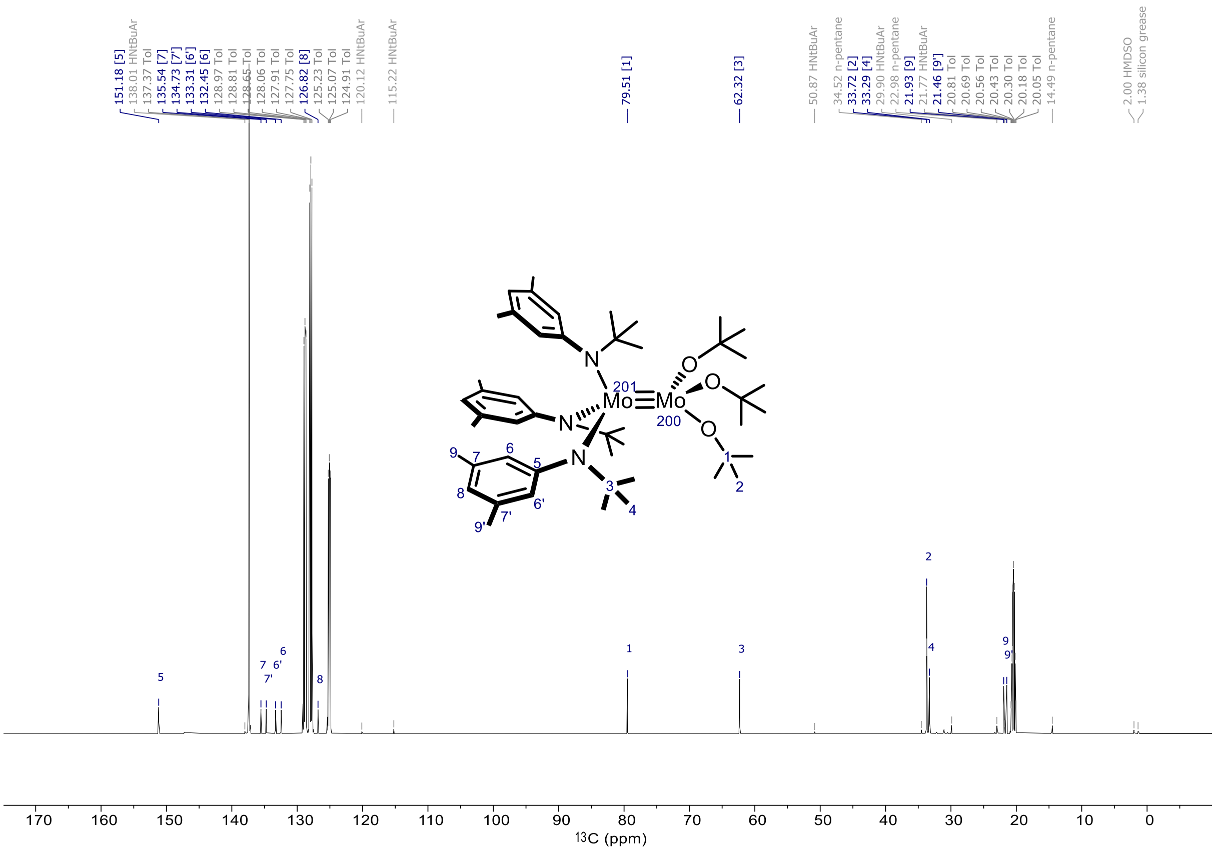

^{1}H-^{13}C HSQC NMR spectrum of complex 17: [D$_8$]-toluene, 233 K, 600 MHz, 151 MHz.

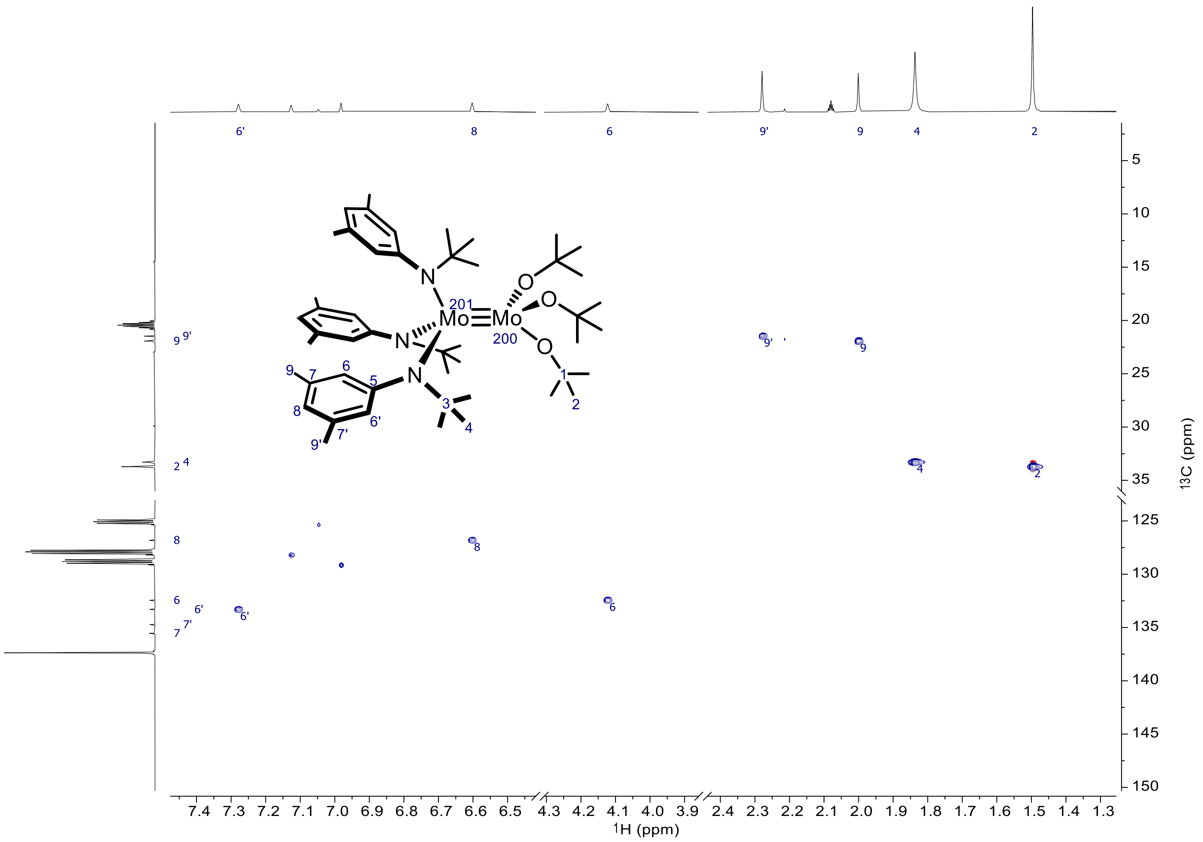

^{1}H-^{13}C HMBC NMR spectrum of complex 17: [D$_8$]-toluene, 233 K, 600 MHz, 151 MHz.

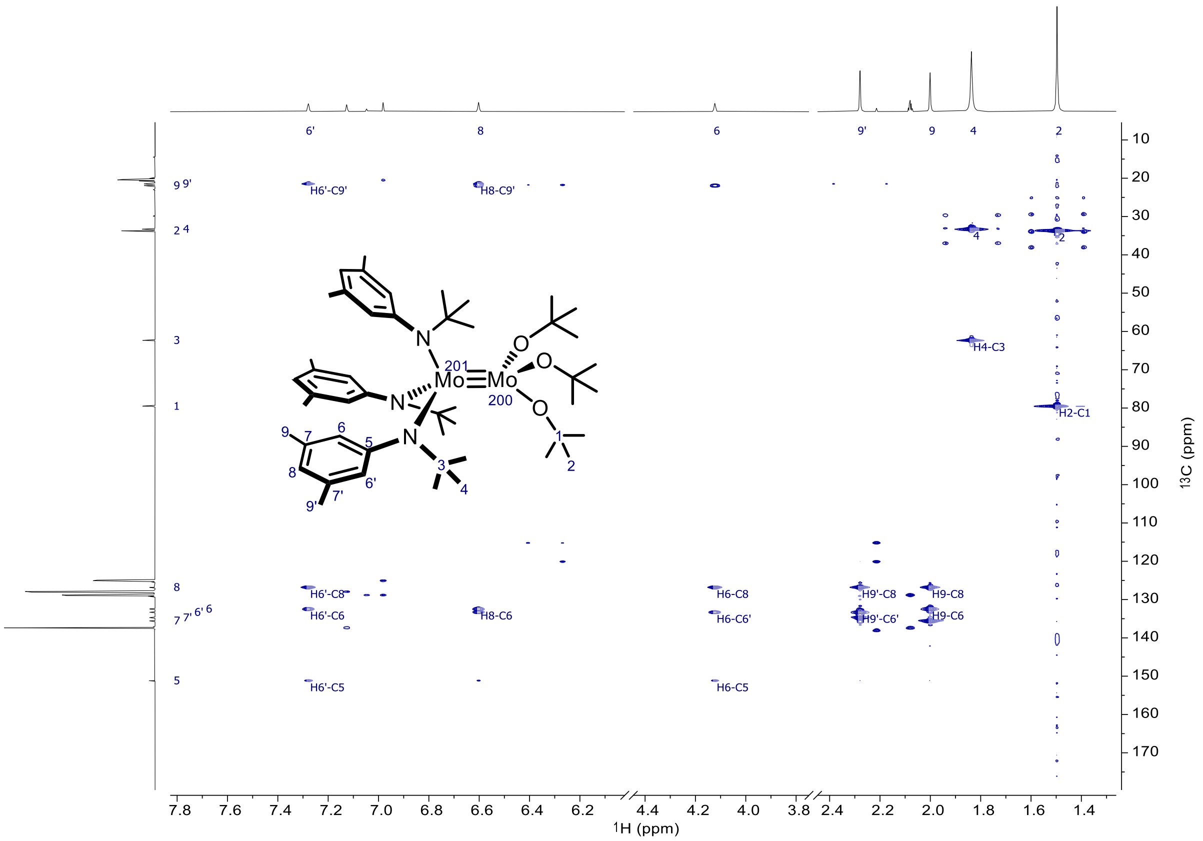

^{1}H-^{1}H COSY NMR spectrum of complex 17: [D$_8$]-toluene, 233 K, 600 MHz, 600 MHz.

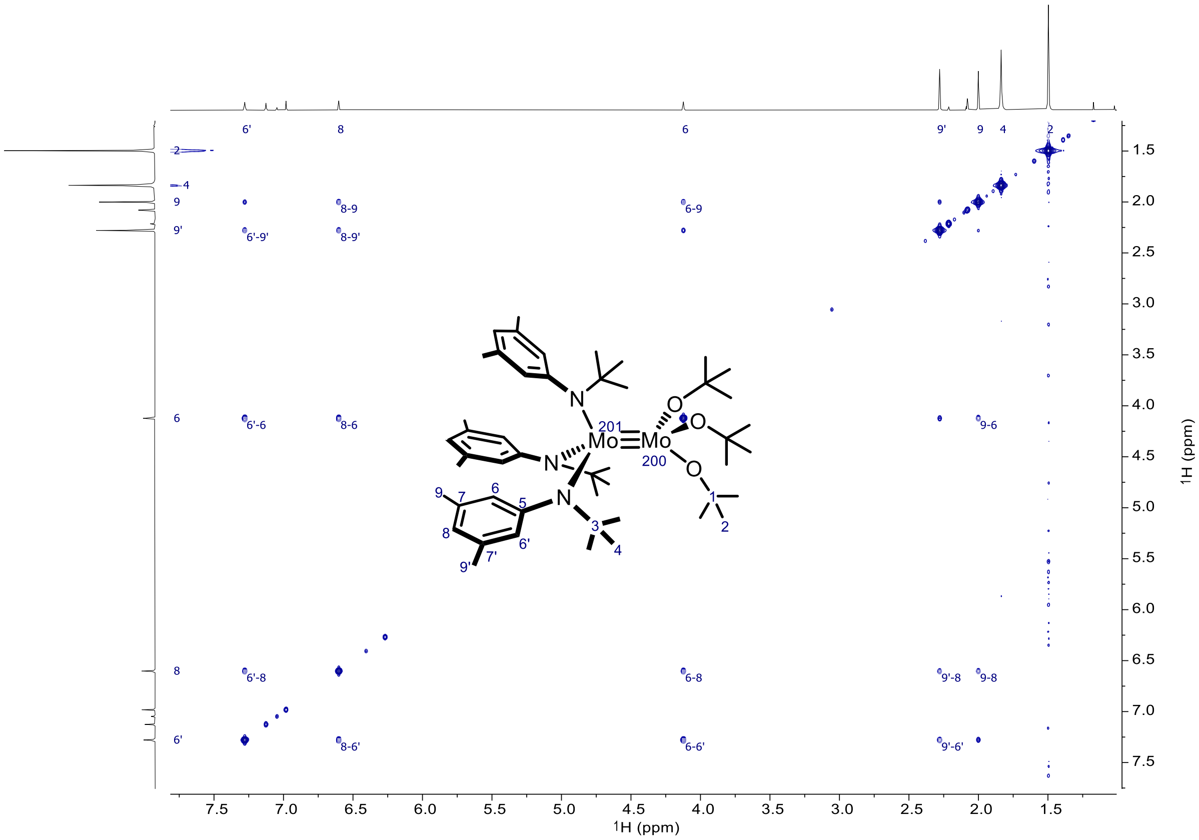

^{1}H-^{1}H ROESY NMR spectrum of complex 17: [D$_8$]-toluene, 233 K, 600 MHz, 600 MHz.

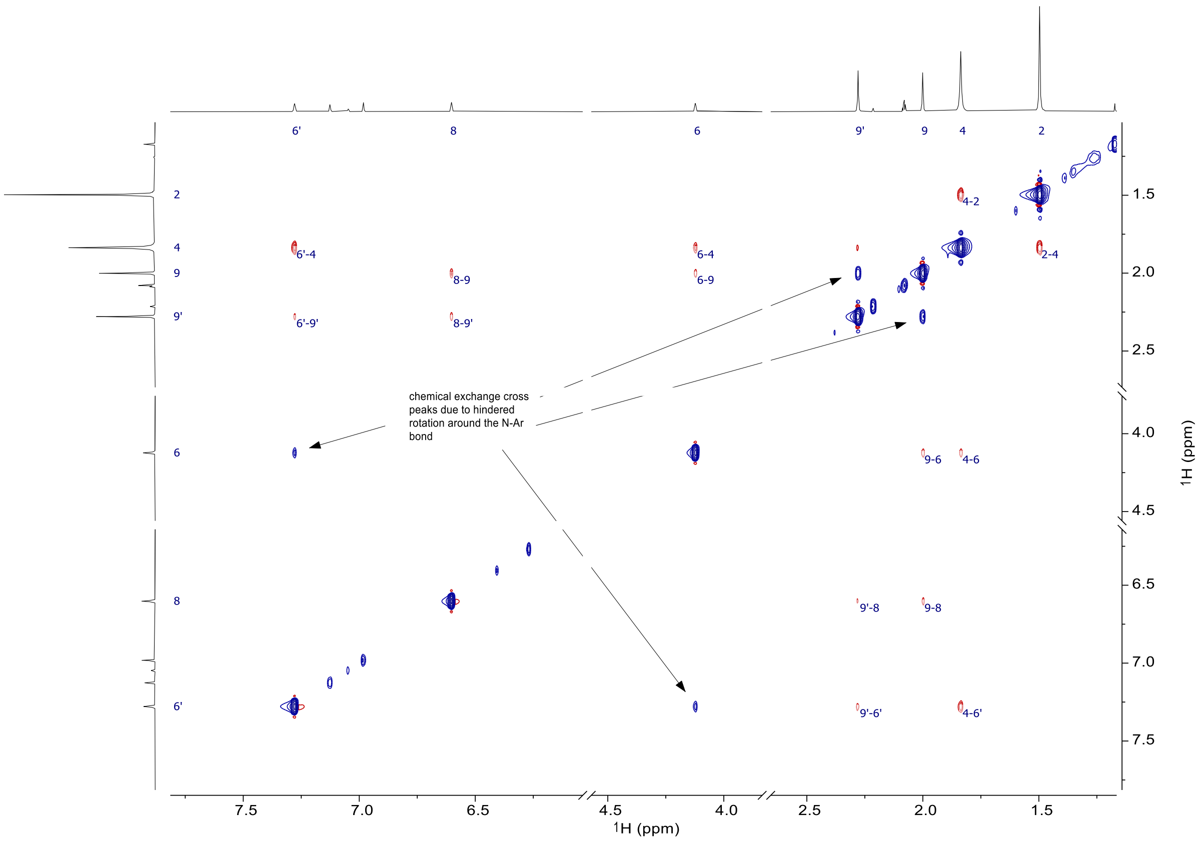

^{1}H-^{15}N HMBC NMR spectrum of complex 17: [D$_8$]-toluene, 233 K, 600 MHz, 61 MHz.

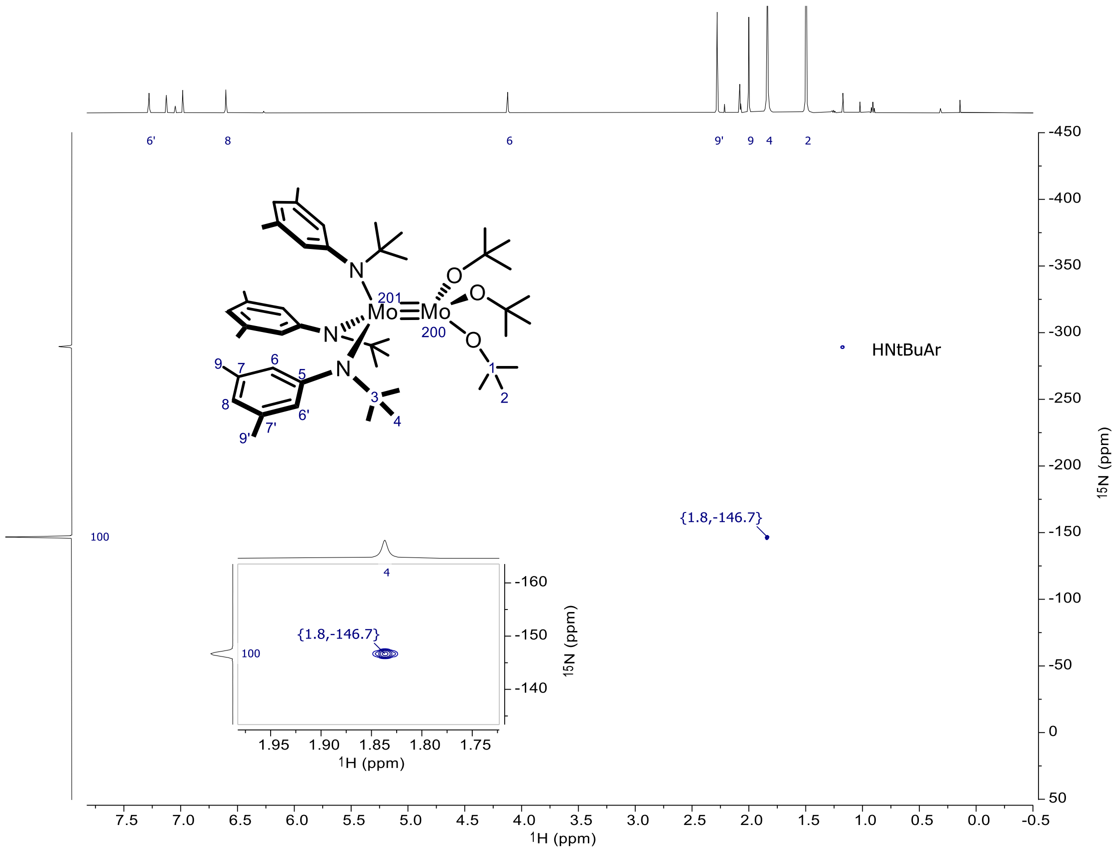

^{95}Mo NMR spectrum of complex 17: [D$_8$]-toluene, 333 K, 26 MHz. *Note*: To obtain quantitative data the pulse offset was set between the 2 peaks (3200 ppm); when the offset was set to 3400 ppm, only the signal of Mo-200 was visible; when set to 3000, Mo-201 was more intense than Mo-200

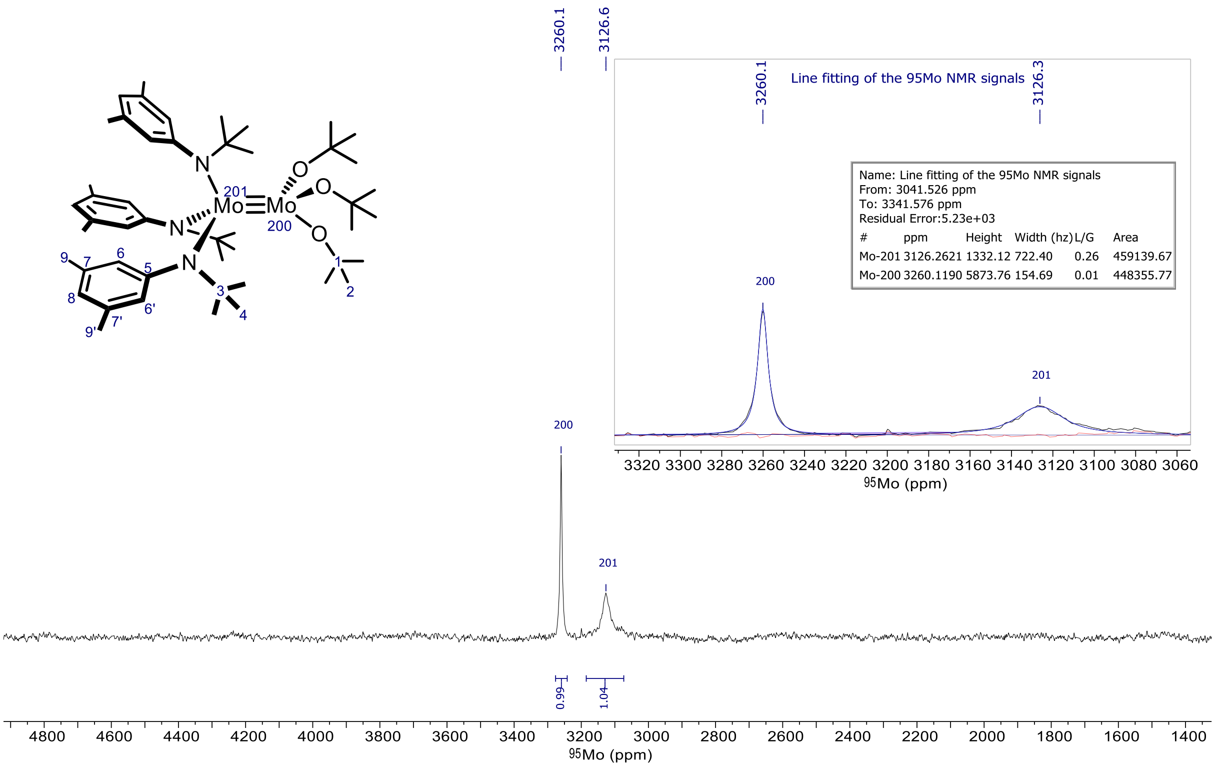

¹H NMR spectra of complex 17: [D$_8$]-toluene, 233 K−353 K, 600 MHz. Sharpening of the ¹H NMR signals indicates the reduced rotational exchange.

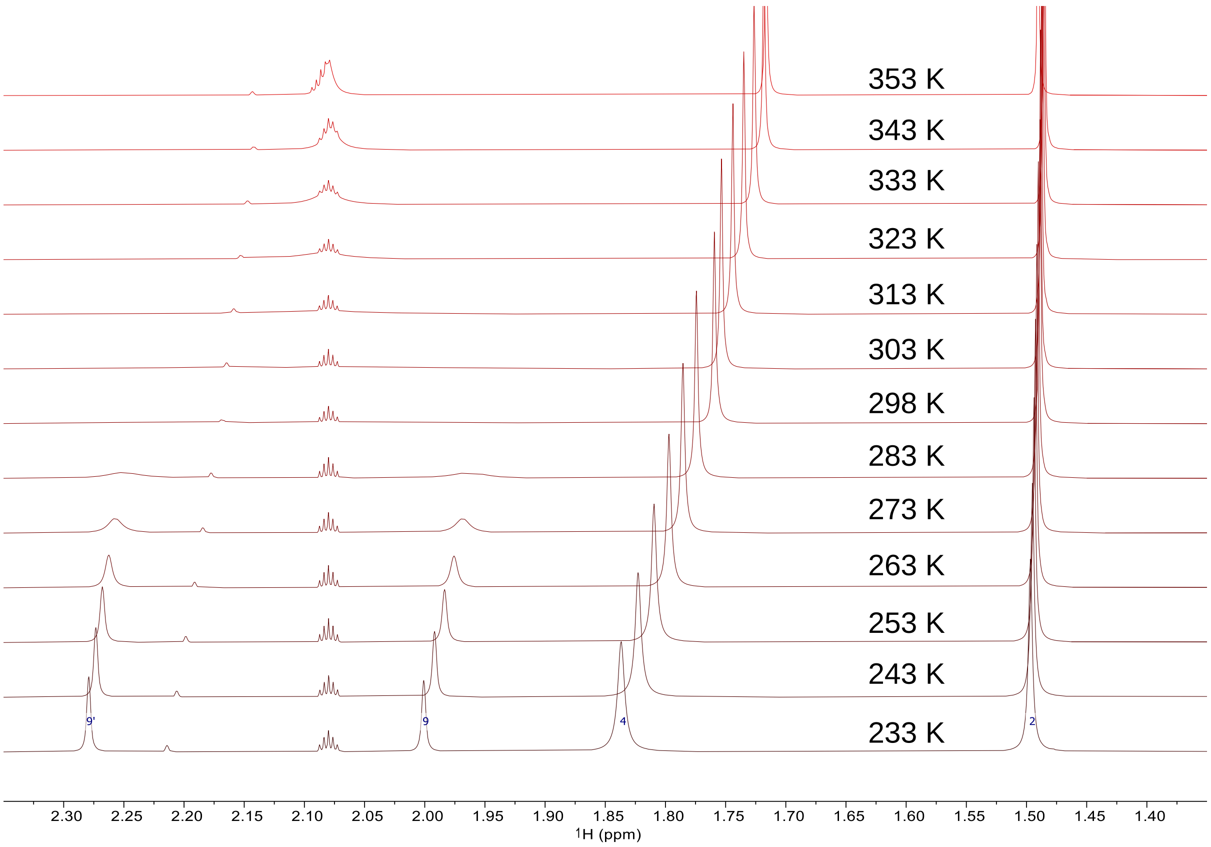

^{1}H NMR spectra of complex 17: [D_8]-toluene, 233 K–353 K, 600 MHz. Coalescence temperature of the H6-H6' exchange and estimation of the rotational barrier about the N–Ar bond.

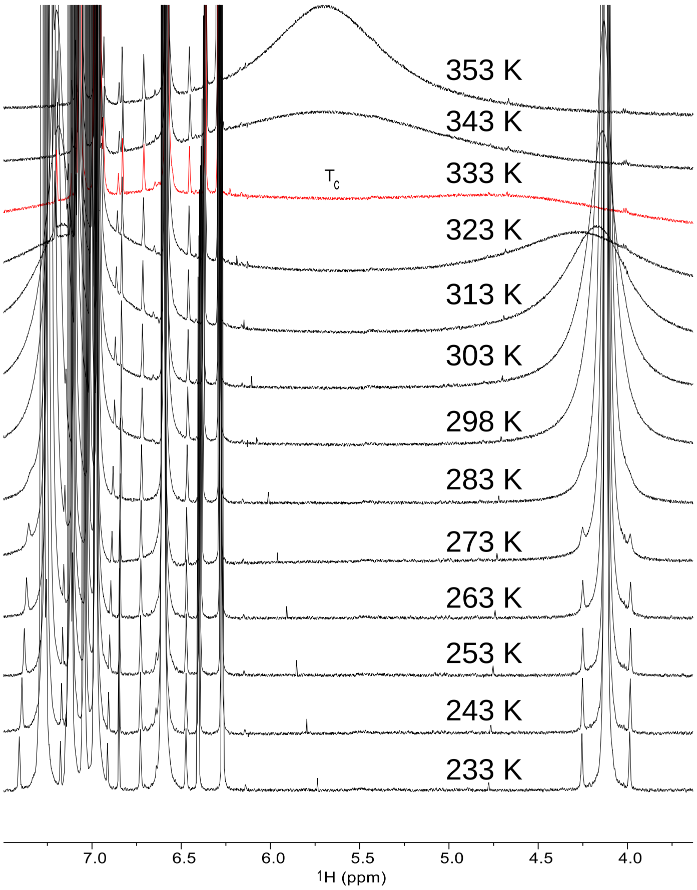

$T_c = 333\ K$

$\Delta\nu = 18884\ Hz$

$R = 8.314\ \frac{J}{K \cdot mol}$

$h = 6.626 \cdot 10^{-34}\ J \cdot s$

$k_B = 1.381 \cdot 10^{-23} \frac{J}{K}$

$At\ 333\ K:$

$$k_{exch} = \frac{\pi \cdot \Delta\nu}{\sqrt{2}} = 41949.7\ Hz$$

$$\Delta G^{\ddagger}{}_{T_c} = -ln\left(\frac{k_{exch} \cdot h}{k_B \cdot T_c}\right) \cdot R \cdot T = 52.4\ \frac{kJ}{mol} = 12.5\ \frac{kcal}{mol}$$

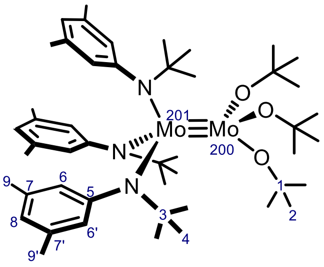

REFERENCES

[1] C. E. Laplaza, M. J. A. Johnson, J. C. Peters, A. L. Odom, E. Kim, C. C. Cummins, G. N. George, I. J. Pickering, *J. Am. Chem. Soc.* **1996**, *118*, 8623-8638.

[2] C. E. Laplaza, A. L. Odom, W. M. Davis, C. C. Cummins, J. D. Protasiewicz, *J. Am. Chem. Soc.* **1995**, *117*, 4999-5000.

[3] A. Fürstner, C. Mathes, C. W. Lehmann, *Chem. Eur. J.* **2001**, *7*, 5299-5317.

[4] J. J. Curley, Dissertation, Massachusetts Institute of Technology, **2009**.

[5] J. Hillenbrand, M. Leutzsch, A. Fürstner, *Angew. Chem.* **2019**, *131*, 15837-15843; *Angew. Chem. Int. Ed.* **2019**, *58*, 15690-15696.

[6] J. Hillenbrand, M. Leutzsch, E. Yiannakas, C. P. Gordon, C. Wille, N. Nöthling, C. Copéret, A. Fürstner, *J. Am. Chem. Soc.* **2020**, *142*, 11279-11294.

[7] J. Hillenbrand, M. Leutzsch, C. P. Gordon, C. Copéret, A. Fürstner, *Angew. Chem.* **2020**, *132*, 21942-21952; *Angew. Chem. Int. Ed.* **2020**, *59*, 21758-21768.

[8] R. K. Harris, E. D. Becker, S. M. C. d. Menezes, P. Granger, R. E. Hoffman, K. W. Zilm, *Pure Appl. Chem.* **2008**, *80*, 59-84.

[9] C. G. Young, E. M. Kober, J. H. Enemark, *Polyhedron* **1987**, *6*, 255-259.

[10] D. Rütter, D., M. van Gastel, M. Leutzsch, N. Nöthling, D. SantaLucia, F. Neese, A. Fürstner, *Inorg. Chem.* **2024,** *63* (18), 8376-8389.
