# Supplementary material for: Tripodal Silanolate Ligands Expand [MoX3] Chemistry Beyond Its Traditional Borders
Source: J Am Chem Soc. 2025 Apr 11;147(16):13871–84. doi: 10.1021/jacs.5c02178 (PMC12022994; doi:10.1021/jacs.5c02178)
Supplement: Supplementary file 2 — ja5c02178_si_002.pdf [file ja5c02178_si_002.pdf]

Supporting Crystallographic Information

Tripodal Silanolate Ligands Expand [MoX_3] Chemistry Beyond Its Traditional Borders

Daniel Rütter, Nils Nöthling, Markus Leutzsch, Alexander A. Auer, Alois Fürstner*

Max-Planck-Institut für Kohlenforschung, 45470 Mülheim/Ruhr, Germany

Email: fuerstner@kofo.mpg.de

Table of Contents

Single Crystal Structure Analysis of Complex [**12**·3thf] S2
Single Crystal Structure Analysis of Complex [**12**·thf] S11
Crystallographic Discussion of the Structure of Complex [**12**·thf].......... S23
Single Crystal Structure Analysis of the Homodimeric Complex **13** S35
Single Crystal Structure Analysis of Complex **14**.......... S46
Single Crystal Structure Analysis of Complex **16**.......... S60
Single Crystal Structure Analysis of Complex **17**.......... S67
Plot of the Mo≡Mo Bond Distances Determined by X-Ray Diffraction Analysis S78
References S94

Single Crystal Structure Analysis of Complex [12·3thf]

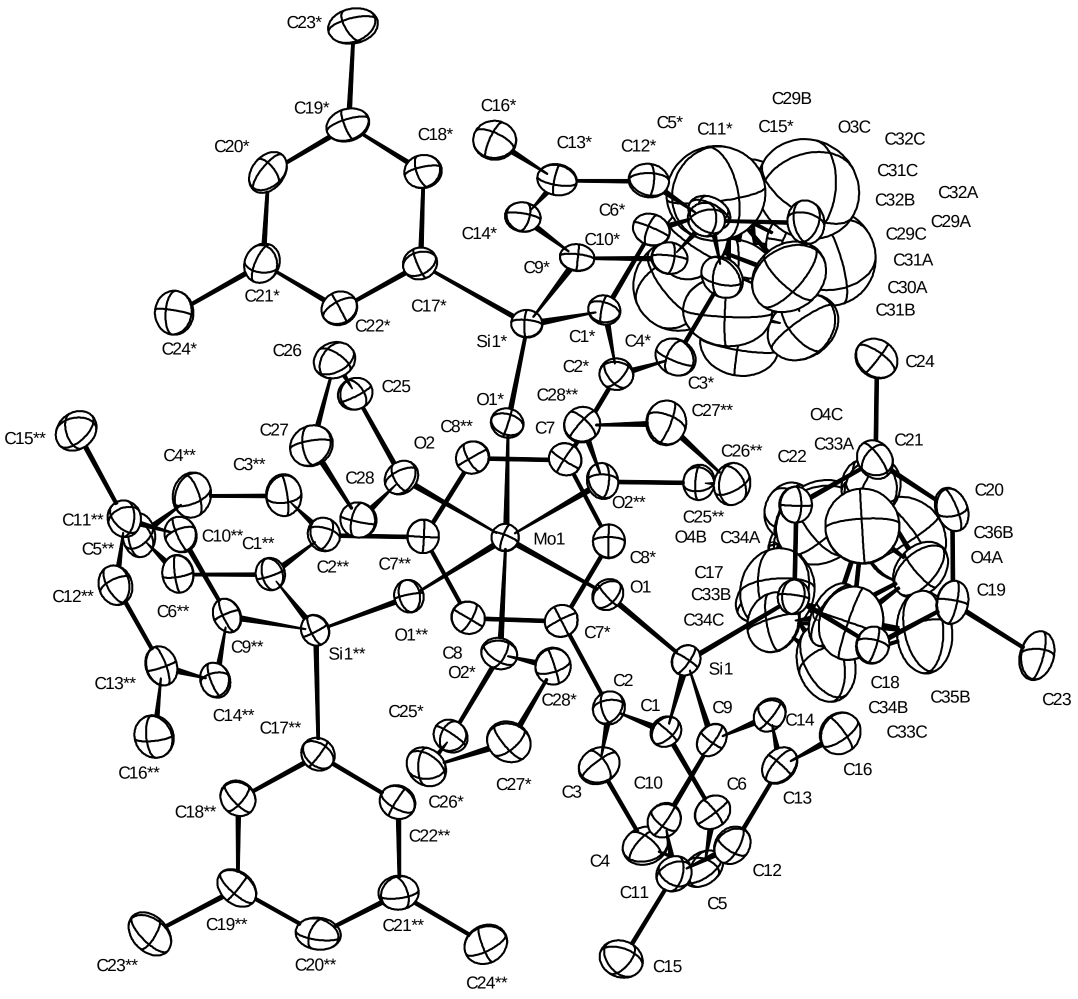

Figure S1. The molecular structure of complex [**12**·3thf]; H atoms have been removed for clarity.

X-ray Crystal Structure Analysis of Complex [12·3thf**]**: C_{113} H_{149} Mo $O_{13.25}$ Si_3, M_r = 1899.52 g mol^{-1}, yellow prism, crystal size 0.147 x 0.090 x 0.088 mm^3, Cubic, space group $P2_13$ [198], a = 21.9285(6) Å, V = 10544.5(9) Å^3, T = 150(2) K, Z = 4, D_{calc} = 1.197 g·cm^3, λ = 0.71073 Å, μ(*Mo-Kα*) = 0.218 mm^{-1}, Numerical absorption correction ($T_{\min}$ = 0.9213, $T_{\max}$ = 0.9805), 2.077 < θ < 28.303°, Bruker AXS D8-Venture diffractometer with IµS Diamond Mo-anode X-ray source and PHOTON III detector, 416339 measured reflections, 8748 independent reflections, 7907 reflections with $I > 2\sigma(I)$, R_{int} = 0.1252. The structure was solved by *SHELXT* and refined by full-matrix least-squares (*SHELXL*) against F^2 to R_1 = 0.0331 [$I > 2\sigma(I)$], wR_2 = 0.0869 [all data], 560 parameters and 631 restraints. Full .cif data for the compound are available under CCDC-**2418345**.

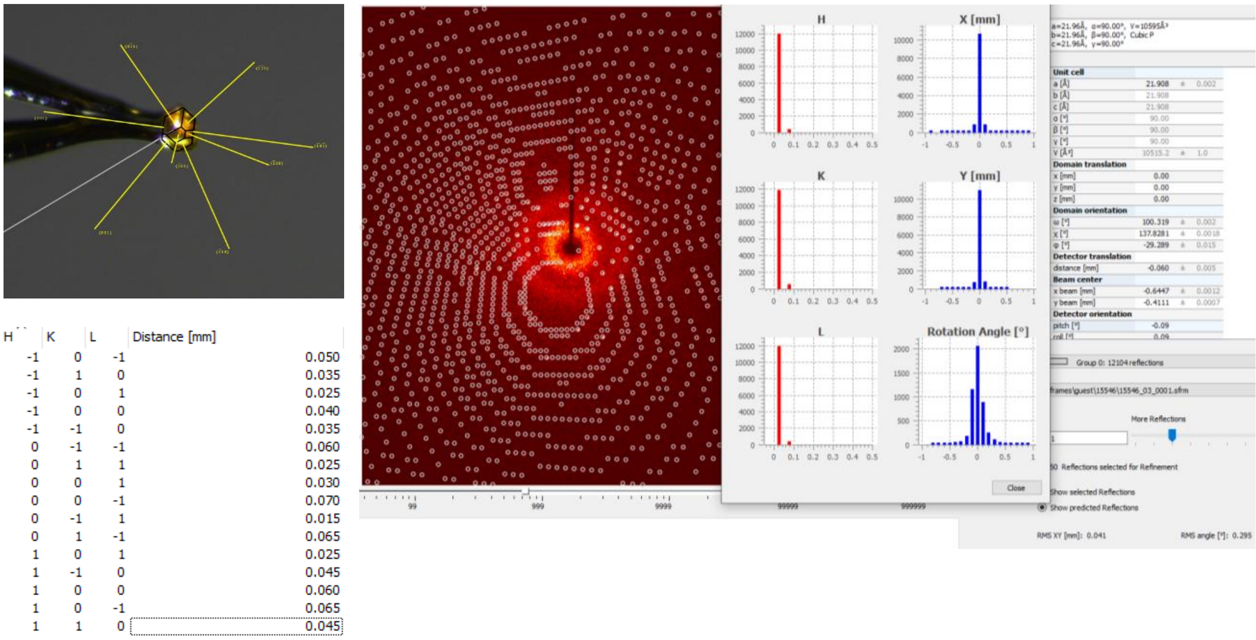

Figure S2. Crystal faces and unit cell determination/refinement of [**12**·3thf].

INTENSITY STATISTICS FOR DATASET

Resolution	#Data	#Theory	%Complete	Redundancy		Mean I	Mean I/s	Rmerge	Rsigma
Inf - 3.26	73	76	96.1	97.5	86.22	1.86	0.0151	0.0301	0.0030
3.26 - 2.12	165	165	100.0	100.0	107.16	1.64	0.0235	0.0515	0.0049
2.12 - 1.66	243	243	100.0	100.0	110.02	1.50	0.0254	0.0631	0.0059
1.66 - 1.44	231	231	100.0	100.0	112.13	1.25	0.0266	0.0867	0.0081
1.44 - 1.30	239	239	100.0	100.0	113.52	1.42	0.0351	0.1081	0.0101
1.30 - 1.20	252	252	100.0	100.0	113.19	1.51	0.0452	0.1285	0.0120
1.20 - 1.13	231	231	100.0	100.0	110.57	1.19	0.0374	0.1437	0.0136
1.13 - 1.07	245	245	100.0	100.0	111.18	1.19	0.0408	0.1538	0.0145
1.07 - 1.02	257	257	100.0	100.0	104.30	1.09	0.0446	0.1667	0.0162
1.02 - 0.98	226	226	100.0	100.0	92.04	1.07	0.0551	0.1963	0.0204
0.98 - 0.95	203	203	100.0	100.0	87.14	0.99	0.0527	0.2123	0.0226
0.95 - 0.92	230	230	100.0	100.0	79.76	0.93	0.0600	0.2350	0.0262
0.92 - 0.89	266	266	100.0	100.0	76.93	1.07	0.0737	0.2436	0.0275
0.89 - 0.86	308	308	100.0	100.0	73.95	0.96	0.0787	0.2771	0.0321
0.86 - 0.84	217	217	100.0	100.0	72.35	0.89	0.0754	0.2944	0.0344
0.84 - 0.82	252	252	100.0	100.0	70.86	0.86	0.0820	0.3123	0.0370
0.82 - 0.80	271	271	100.0	100.0	68.53	0.97	0.1100	0.3614	0.0434
0.80 - 0.79	140	140	100.0	100.0	66.99	0.88	0.0961	0.3571	0.0433
0.79 - 0.77	333	333	100.0	100.0	66.13	0.89	0.1198	0.4024	0.0492
0.77 - 0.76	142	142	100.0	100.0	65.72	0.83	0.1280	0.4419	0.0542
0.76 - 0.75	203	204	99.5	98.9	61.62	0.82	0.1462	0.4636	0.0583
0.85 - 0.75	1454	1455	99.9	99.8	67.16	0.88	0.1056	0.3668	0.0444
Inf - 0.75	4727	4731	99.9	99.9	88.27	1.09	0.0427	0.1217	0.0126

A number of reflections showed a high I/σI and have been removed from the data set prior to the final cycles of refinement.

The disordered THF molecules were modelled using the DSR tool implemented in Olex2 and the 'THF' fragment was selected.[1-2] In addition, a solvent mask (SQUEEZE) was applied to eliminate the residual electron density caused by unrefinable THF molecules. Solvent masking was performed by using the BYPASS implementation of Olex2.[3-4] This results in a solvent-accessible void of 580.54 Å³ (5.5% of the unit cell volume) at a probe radius of 1.2 Å and an approximate lattice spacing of 0.3 Å.

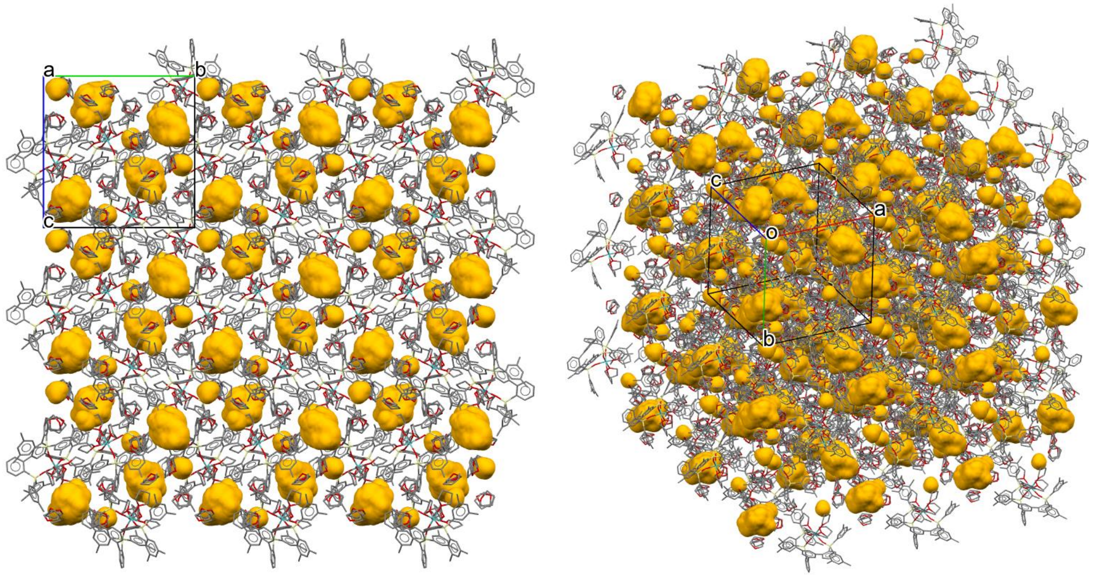

Figure S3. Solvent accessible voids in the crystal structure of [**12**·3thf] along the crystallographic a-axis (left) and in a random orientation (right).

Table S1. Crystal data and structure refinement of complex [**12**·3thf].

Identification code	15546	
Empirical formula	$C_{113} H_{149} Mo O_{13.25} Si_3$	
Color	yellow	
Formula weight	1899.52 g·mol^{-1}	
Temperature	150(2) K	
Wavelength	0.71073 Å	
Crystal system	Cubic	
Space group	$P2_13$, (no. 198)	
Unit cell dimensions	a = 21.9285(6) Å	α = 90°.
	b = 21.9285(6) Å	β = 90°.
	c = 21.9285(6) Å	γ = 90°
Volume	10544.5(9) Å^3	
Z	4	
Density (calculated)	1.197 Mg·m^{-3}	
Absorption coefficient	0.218 mm^{-1}	
F(000)	4068 e	
Crystal size	0.147 x 0.090 x 0.088 mm^3	
θ range for data collection	2.077 to 28.303°.	
Index ranges	$-29 \le h \le 29$, $-29 \le k \le 29$, $-29 \le l \le 29$	
Reflections collected	416339	
Independent reflections	8748 [R_{int} = 0.1252]	
Reflections with I>2σ(I)	7907	
Completeness to θ = 25.242°	99.5 %	
Absorption correction	Numerical	
Max. and min. transmission	0.9805 and 0.9213	
Refinement method	Full-matrix least-squares on F^2	
Data / restraints / parameters	8748 / 631 / 560	
Goodness-of-fit on F^2	1.089	
Final R indices [I>2σ(I)]	R_1 = 0.0331	wR^2 = 0.0812
R indices (all data)	R_1 = 0.0416	wR^2 = 0.0869
Absolute structure parameter	-0.043(9)	
Extinction coefficient	n/a	
Largest diff. peak and hole	0.316 and -0.307 e·Å^{-3}	

Table S2. Bond lengths [Å] and angles [°] of complex [**12**·3thf]

Mo(1)-O(1)#1	2.0146(19)	Mo(1)-O(1)#2	2.0146(19)
Mo(1)-O(1)	2.0146(18)	Mo(1)-O(2)#2	2.2407(19)
Mo(1)-O(2)#1	2.2407(19)	Mo(1)-O(2)	2.2407(19)
Si(1)-O(1)	1.585(2)	Si(1)-C(1)	1.898(3)
Si(1)-C(9)	1.892(3)	Si(1)-C(17)	1.895(3)
O(2)-C(25)	1.449(3)	O(2)-C(28)	1.454(3)
C(1)-C(2)	1.411(4)	C(1)-C(6)	1.397(4)
C(2)-C(3)	1.398(4)	C(2)-C(7)	1.486(4)
C(3)-H(3)	0.9500	C(3)-C(4)	1.383(5)
C(4)-H(4)	0.9500	C(4)-C(5)	1.375(5)
C(5)-H(5)	0.9500	C(5)-C(6)	1.393(4)
C(6)-H(6)	0.9500	C(7)-C(8)	1.396(4)
C(7)-C(8)#2	1.390(4)	C(8)-H(8)	0.9500
C(9)-C(10)	1.405(4)	C(9)-C(14)	1.397(4)
C(10)-H(10)	0.9500	C(10)-C(11)	1.395(4)
C(11)-C(12)	1.394(4)	C(11)-C(15)	1.506(4)
C(12)-H(12)	0.9500	C(12)-C(13)	1.390(4)
C(13)-C(14)	1.392(4)	C(13)-C(16)	1.511(4)
C(14)-H(14)	0.9500	C(15)-H(15A)	0.9800
C(15)-H(15B)	0.9800	C(15)-H(15C)	0.9800
C(16)-H(16A)	0.9800	C(16)-H(16B)	0.9800
C(16)-H(16C)	0.9800	C(17)-C(18)	1.401(4)
C(17)-C(22)	1.389(4)	C(18)-H(18)	0.9500
C(18)-C(19)	1.393(5)	C(19)-C(20)	1.396(5)
C(19)-C(23)	1.518(5)	C(20)-H(20)	0.9500
C(20)-C(21)	1.387(5)	C(21)-C(22)	1.401(4)
C(21)-C(24)	1.515(5)	C(22)-H(22)	0.9500
C(23)-H(23A)	0.9800	C(23)-H(23B)	0.9800
C(23)-H(23C)	0.9800	C(24)-H(24A)	0.9800
C(24)-H(24B)	0.9800	C(24)-H(24C)	0.9800
C(25)-H(25A)	0.9900	C(25)-H(25B)	0.9900
C(25)-C(26)	1.509(4)	C(26)-H(26A)	0.9900
C(26)-H(26B)	0.9900	C(26)-C(27)	1.523(5)
C(27)-H(27A)	0.9900	C(27)-H(27B)	0.9900
C(27)-C(28)	1.525(4)	C(28)-H(28A)	0.9900
C(28)-H(28B)	0.9900	O(3A)-C(29A)	1.465(19)
O(3A)-C(32A)	1.48(2)	C(29A)-H(29A)	0.9900
C(29A)-H(29B)	0.9900	C(29A)-C(30A)	1.542(18)
C(30A)-H(30A)	0.9900	C(30A)-H(30B)	0.9900
C(30A)-C(31A)	1.536(19)	C(31A)-H(31A)	0.9900
C(31A)-H(31B)	0.9900	C(31A)-C(32A)	1.567(19)
C(32A)-H(32A)	0.9900	C(32A)-H(32B)	0.9900
O(4A)-C(33A)	1.504(18)	O(4A)-C(36A)	1.502(19)
C(33A)-H(33A)	0.9900	C(33A)-H(33B)	0.9900

C(33A)-C(34A)	1.536(18)	C(34A)-H(34A)	0.9900
C(34A)-H(34B)	0.9900	C(34A)-C(35A)	1.473(17)
C(35A)-H(35A)	0.9900	C(35A)-H(35B)	0.9900
C(35A)-C(36A)	1.517(17)	C(36A)-H(36A)	0.9900
C(36A)-H(36B)	0.9900	O(3B)-C(29B)	1.433(15)
O(3B)-C(32B)	1.387(14)	C(29B)-H(29C)	0.9900
C(29B)-H(29D)	0.9900	C(29B)-C(30B)	1.515(15)
C(30B)-H(30C)	0.9900	C(30B)-H(30D)	0.9900
C(30B)-C(31B)	1.563(15)	C(31B)-H(31C)	0.9900
C(31B)-H(31D)	0.9900	C(31B)-C(32B)	1.494(14)
C(32B)-H(32C)	0.9900	C(32B)-H(32D)	0.9900
O(4B)-C(33B)	1.370(18)	O(4B)-C(36B)	1.459(19)
C(33B)-H(33C)	0.9900	C(33B)-H(33D)	0.9900
C(33B)-C(34B)	1.541(17)	C(34B)-H(34C)	0.9900
C(34B)-H(34D)	0.9900	C(34B)-C(35B)	1.543(15)
C(35B)-H(35C)	0.9900	C(35B)-H(35D)	0.9900
C(35B)-C(36B)	1.569(17)	C(36B)-H(36C)	0.9900
C(36B)-H(36D)	0.9900	O(3C)-C(29C)	1.45(2)
O(3C)-C(32C)	1.45(2)	C(29C)-H(29E)	0.9900
C(29C)-H(29F)	0.9900	C(29C)-C(30C)	1.57(2)
C(30C)-H(30E)	0.9900	C(30C)-H(30F)	0.9900
C(30C)-C(31C)	1.57(2)	C(31C)-H(31E)	0.9900
C(31C)-H(31F)	0.9900	C(31C)-C(32C)	1.58(2)
C(32C)-H(32E)	0.9900	C(32C)-H(32F)	0.9900
O(4C)-C(33C)	1.46(2)	O(4C)-C(36C)	1.481(19)
C(33C)-H(33E)	0.9900	C(33C)-H(33F)	0.9900
C(33C)-C(34C)	1.505(18)	C(34C)-H(34E)	0.9900
C(34C)-H(34F)	0.9900	C(34C)-C(35C)	1.518(16)
C(35C)-H(35E)	0.9900	C(35C)-H(35F)	0.9900
C(35C)-C(36C)	1.528(17)	C(36C)-H(36E)	0.9900
C(36C)-H(36F)	0.9900		

O(1)#1-Mo(1)-O(1)#2	95.46(7)	O(1)#1-Mo(1)-O(1)	95.46(7)
O(1)#2-Mo(1)-O(1)	95.46(7)	O(1)#2-Mo(1)-O(2)#2	172.19(8)
O(1)-Mo(1)-O(2)#1	88.91(7)	O(1)#1-Mo(1)-O(2)#1	172.19(8)
O(1)-Mo(1)-O(2)#2	90.54(8)	O(1)#1-Mo(1)-O(2)	90.55(8)
O(1)#2-Mo(1)-O(2)#1	90.54(8)	O(1)#2-Mo(1)-O(2)	88.91(7)
O(1)-Mo(1)-O(2)	172.19(8)	O(1)#1-Mo(1)-O(2)#2	88.91(7)
O(2)-Mo(1)-O(2)#1	84.57(8)	O(2)#2-Mo(1)-O(2)#1	84.57(8)
O(2)-Mo(1)-O(2)#2	84.57(8)	O(1)-Si(1)-C(1)	116.69(11)
O(1)-Si(1)-C(9)	109.71(11)	O(1)-Si(1)-C(17)	111.43(12)
C(9)-Si(1)-C(1)	106.74(12)	C(9)-Si(1)-C(17)	106.22(13)
C(17)-Si(1)-C(1)	105.43(12)	Si(1)-O(1)-Mo(1)	163.16(12)
C(25)-O(2)-Mo(1)	125.55(16)	C(25)-O(2)-C(28)	107.1(2)
C(28)-O(2)-Mo(1)	126.77(16)	C(2)-C(1)-Si(1)	124.7(2)
C(6)-C(1)-Si(1)	118.2(2)	C(6)-C(1)-C(2)	117.0(3)
C(1)-C(2)-C(7)	121.5(3)	C(3)-C(2)-C(1)	120.0(3)

C(3)-C(2)-C(7)	118.5(3)	C(2)-C(3)-H(3)	119.2
C(4)-C(3)-C(2)	121.5(3)	C(4)-C(3)-H(3)	119.2
C(3)-C(4)-H(4)	120.4	C(5)-C(4)-C(3)	119.3(3)
C(5)-C(4)-H(4)	120.4	C(4)-C(5)-H(5)	120.1
C(4)-C(5)-C(6)	119.8(3)	C(6)-C(5)-H(5)	120.1
C(1)-C(6)-H(6)	118.8	C(5)-C(6)-C(1)	122.4(3)
C(5)-C(6)-H(6)	118.8	C(8)#2-C(7)-C(2)	120.0(2)
C(8)-C(7)-C(2)	120.8(2)	C(8)#2-C(7)-C(8)	119.1(3)
C(7)#1-C(8)-C(7)	120.9(3)	C(7)#1-C(8)-H(8)	119.6
C(7)-C(8)-H(8)	119.6	C(10)-C(9)-Si(1)	121.5(2)
C(14)-C(9)-Si(1)	120.8(2)	C(14)-C(9)-C(10)	117.1(3)
C(9)-C(10)-H(10)	118.9	C(11)-C(10)-C(9)	122.2(3)
C(11)-C(10)-H(10)	118.9	C(10)-C(11)-C(15)	120.3(3)
C(12)-C(11)-C(10)	118.3(3)	C(12)-C(11)-C(15)	121.3(3)
C(11)-C(12)-H(12)	119.2	C(13)-C(12)-C(11)	121.5(3)
C(13)-C(12)-H(12)	119.2	C(12)-C(13)-C(14)	118.5(3)
C(12)-C(13)-C(16)	121.3(3)	C(14)-C(13)-C(16)	120.2(3)
C(9)-C(14)-H(14)	118.8	C(13)-C(14)-C(9)	122.4(3)
C(13)-C(14)-H(14)	118.8	C(11)-C(15)-H(15A)	109.5
C(11)-C(15)-H(15B)	109.5	C(11)-C(15)-H(15C)	109.5
H(15A)-C(15)-H(15B)	109.5	H(15A)-C(15)-H(15C)	109.5
H(15B)-C(15)-H(15C)	109.5	C(13)-C(16)-H(16A)	109.5
C(13)-C(16)-H(16B)	109.5	C(13)-C(16)-H(16C)	109.5
H(16A)-C(16)-H(16B)	109.5	H(16A)-C(16)-H(16C)	109.5
H(16B)-C(16)-H(16C)	109.5	C(18)-C(17)-Si(1)	119.2(2)
C(22)-C(17)-Si(1)	122.2(2)	C(22)-C(17)-C(18)	118.6(3)
C(17)-C(18)-H(18)	119.2	C(19)-C(18)-C(17)	121.6(3)
C(19)-C(18)-H(18)	119.2	C(18)-C(19)-C(20)	118.1(3)
C(18)-C(19)-C(23)	121.0(3)	C(20)-C(19)-C(23)	120.9(3)
C(19)-C(20)-H(20)	119.0	C(21)-C(20)-C(19)	122.0(3)
C(21)-C(20)-H(20)	119.0	C(20)-C(21)-C(22)	118.4(3)
C(20)-C(21)-C(24)	121.0(3)	C(22)-C(21)-C(24)	120.6(3)
C(17)-C(22)-C(21)	121.3(3)	C(17)-C(22)-H(22)	119.3
C(21)-C(22)-H(22)	119.3	C(19)-C(23)-H(23A)	109.5
C(19)-C(23)-H(23B)	109.5	C(19)-C(23)-H(23C)	109.5
H(23A)-C(23)-H(23B)	109.5	H(23A)-C(23)-H(23C)	109.5
H(23B)-C(23)-H(23C)	109.5	C(21)-C(24)-H(24A)	109.5
C(21)-C(24)-H(24B)	109.5	C(21)-C(24)-H(24C)	109.5
H(24A)-C(24)-H(24B)	109.5	H(24A)-C(24)-H(24C)	109.5
H(24B)-C(24)-H(24C)	109.5	O(2)-C(25)-H(25A)	111.0
O(2)-C(25)-H(25B)	111.0	O(2)-C(25)-C(26)	103.7(2)
H(25A)-C(25)-H(25B)	109.0	C(26)-C(25)-H(25A)	111.0
C(26)-C(25)-H(25B)	111.0	C(25)-C(26)-H(26A)	111.2
C(25)-C(26)-H(26B)	111.2	C(25)-C(26)-C(27)	102.9(3)
H(26A)-C(26)-H(26B)	109.1	C(27)-C(26)-H(26A)	111.2
C(27)-C(26)-H(26B)	111.2	C(26)-C(27)-H(27A)	110.8
C(26)-C(27)-H(27B)	110.8	C(26)-C(27)-C(28)	104.9(2)

H(27A)-C(27)-H(27B)	108.8	C(28)-C(27)-H(27A)	110.8
C(28)-C(27)-H(27B)	110.8	O(2)-C(28)-C(27)	106.2(2)
O(2)-C(28)-H(28A)	110.5	O(2)-C(28)-H(28B)	110.5
C(27)-C(28)-H(28A)	110.5	C(27)-C(28)-H(28B)	110.5
H(28A)-C(28)-H(28B)	108.7	C(29A)-O(3A)-C(32A)	104.5(17)
O(3A)-C(29A)-H(29A)	110.9	O(3A)-C(29A)-H(29B)	110.9
O(3A)-C(29A)-C(30A)	104.1(17)	H(29A)-C(29A)-H(29B)	108.9
C(30A)-C(29A)-H(29A)	110.9	C(30A)-C(29A)-H(29B)	110.9
C(29A)-C(30A)-H(30A)	112.8	C(29A)-C(30A)-H(30B)	112.8
H(30A)-C(30A)-H(30B)	110.2	C(31A)-C(30A)-C(29A)	94.7(16)
C(31A)-C(30A)-H(30A)	112.8	C(31A)-C(30A)-H(30B)	112.8
C(30A)-C(31A)-H(31A)	113.0	C(30A)-C(31A)-H(31B)	113.0
C(30A)-C(31A)-C(32A)	93.6(18)	H(31A)-C(31A)-H(31B)	110.4
C(32A)-C(31A)-H(31A)	113.0	C(32A)-C(31A)-H(31B)	113.0
O(3A)-C(32A)-C(31A)	102.9(17)	O(3A)-C(32A)-H(32A)	111.2
O(3A)-C(32A)-H(32B)	111.2	C(31A)-C(32A)-H(32A)	111.2
C(31A)-C(32A)-H(32B)	111.2	H(32A)-C(32A)-H(32B)	109.1
C(36A)-O(4A)-C(33A)	90.5(16)	O(4A)-C(33A)-H(33A)	108.9
O(4A)-C(33A)-H(33B)	108.9	O(4A)-C(33A)-C(34A)	113.5(16)
H(33A)-C(33A)-H(33B)	107.7	C(34A)-C(33A)-H(33A)	108.9
C(34A)-C(33A)-H(33B)	108.9	C(33A)-C(34A)-H(34A)	111.4
C(33A)-C(34A)-H(34B)	111.4	H(34A)-C(34A)-H(34B)	109.3
C(35A)-C(34A)-C(33A)	101.7(14)	C(35A)-C(34A)-H(34A)	111.4
C(35A)-C(34A)-H(34B)	111.4	C(34A)-C(35A)-H(35A)	111.1
C(34A)-C(35A)-H(35B)	111.1	C(34A)-C(35A)-C(36A)	103.4(13)
H(35A)-C(35A)-H(35B)	109.1	C(36A)-C(35A)-H(35A)	111.1
C(36A)-C(35A)-H(35B)	111.1	O(4A)-C(36A)-C(35A)	113.9(16)
O(4A)-C(36A)-H(36A)	108.8	O(4A)-C(36A)-H(36B)	108.8
C(35A)-C(36A)-H(36A)	108.8	C(35A)-C(36A)-H(36B)	108.8
H(36A)-C(36A)-H(36B)	107.7	C(32B)-O(3B)-C(29B)	113.8(10)
O(3B)-C(29B)-H(29C)	110.2	O(3B)-C(29B)-H(29D)	110.2
O(3B)-C(29B)-C(30B)	107.4(10)	H(29C)-C(29B)-H(29D)	108.5
C(30B)-C(29B)-H(29C)	110.2	C(30B)-C(29B)-H(29D)	110.2
C(29B)-C(30B)-H(30C)	111.0	C(29B)-C(30B)-H(30D)	111.0
C(29B)-C(30B)-C(31B)	103.9(10)	H(30C)-C(30B)-H(30D)	109.0
C(31B)-C(30B)-H(30C)	111.0	C(31B)-C(30B)-H(30D)	111.0
C(30B)-C(31B)-H(31C)	110.3	C(30B)-C(31B)-H(31D)	110.3
H(31C)-C(31B)-H(31D)	108.6	C(32B)-C(31B)-C(30B)	106.9(10)
C(32B)-C(31B)-H(31C)	110.3	C(32B)-C(31B)-H(31D)	110.3
O(3B)-C(32B)-C(31B)	107.4(11)	O(3B)-C(32B)-H(32C)	110.2
O(3B)-C(32B)-H(32D)	110.2	C(31B)-C(32B)-H(32C)	110.2
C(31B)-C(32B)-H(32D)	110.2	H(32C)-C(32B)-H(32D)	108.5
C(33B)-O(4B)-C(36B)	92(2)	O(4B)-C(33B)-H(33C)	106.4
O(4B)-C(33B)-H(33D)	106.4	O(4B)-C(33B)-C(34B)	124(2)
H(33C)-C(33B)-H(33D)	106.5	C(34B)-C(33B)-H(33C)	106.4
C(34B)-C(33B)-H(33D)	106.4	C(33B)-C(34B)-H(34C)	111.3
C(33B)-C(34B)-H(34D)	111.3	C(33B)-C(34B)-C(35B)	102.3(13)

H(34C)-C(34B)-H(34D)	109.2	C(35B)-C(34B)-H(34C)	111.3
C(35B)-C(34B)-H(34D)	111.3	C(34B)-C(35B)-H(35C)	112.6
C(34B)-C(35B)-H(35D)	112.6	C(34B)-C(35B)-C(36B)	95.7(13)
H(35C)-C(35B)-H(35D)	110.1	C(36B)-C(35B)-H(35C)	112.6
C(36B)-C(35B)-H(35D)	112.6	O(4B)-C(36B)-C(35B)	122.2(19)
O(4B)-C(36B)-H(36C)	106.8	O(4B)-C(36B)-H(36D)	106.8
C(35B)-C(36B)-H(36C)	106.8	C(35B)-C(36B)-H(36D)	106.8
H(36C)-C(36B)-H(36D)	106.6	C(32C)-O(3C)-C(29C)	111(3)
O(3C)-C(29C)-H(29E)	111.3	O(3C)-C(29C)-H(29F)	111.3
O(3C)-C(29C)-C(30C)	102(3)	H(29E)-C(29C)-H(29F)	109.2
C(30C)-C(29C)-H(29E)	111.3	C(30C)-C(29C)-H(29F)	111.3
C(29C)-C(30C)-H(30E)	111.8	C(29C)-C(30C)-H(30F)	111.8
H(30E)-C(30C)-H(30F)	109.5	C(31C)-C(30C)-C(29C)	100(2)
C(31C)-C(30C)-H(30E)	111.8	C(31C)-C(30C)-H(30F)	111.8
C(30C)-C(31C)-H(31E)	112.4	C(30C)-C(31C)-H(31F)	112.4
C(30C)-C(31C)-C(32C)	97(2)	H(31E)-C(31C)-H(31F)	109.9
C(32C)-C(31C)-H(31E)	112.4	C(32C)-C(31C)-H(31F)	112.4
O(3C)-C(32C)-C(31C)	105(3)	O(3C)-C(32C)-H(32E)	110.7
O(3C)-C(32C)-H(32F)	110.7	C(31C)-C(32C)-H(32E)	110.7
C(31C)-C(32C)-H(32F)	110.7	H(32E)-C(32C)-H(32F)	108.8
C(33C)-O(4C)-C(36C)	115(2)	O(4C)-C(33C)-H(33E)	113.2
O(4C)-C(33C)-H(33F)	113.2	O(4C)-C(33C)-C(34C)	92.3(17)
H(33E)-C(33C)-H(33F)	110.6	C(34C)-C(33C)-H(33E)	113.2
C(34C)-C(33C)-H(33F)	113.2	C(33C)-C(34C)-H(34E)	109.9
C(33C)-C(34C)-H(34F)	109.9	C(33C)-C(34C)-C(35C)	108.7(15)
H(34E)-C(34C)-H(34F)	108.3	C(35C)-C(34C)-H(34E)	109.9
C(35C)-C(34C)-H(34F)	109.9	C(34C)-C(35C)-H(35E)	110.2
C(34C)-C(35C)-H(35F)	110.2	C(34C)-C(35C)-C(36C)	107.6(13)
H(35E)-C(35C)-H(35F)	108.5	C(36C)-C(35C)-H(35E)	110.2
C(36C)-C(35C)-H(35F)	110.2	O(4C)-C(36C)-C(35C)	94.2(14)
O(4C)-C(36C)-H(36E)	112.9	O(4C)-C(36C)-H(36F)	112.9
C(35C)-C(36C)-H(36E)	112.9	C(35C)-C(36C)-H(36F)	112.9
H(36E)-C(36C)-H(36F)	110.3		

Symmetry transformations used to generate equivalent atoms:
#1 z,x,y   #2 y,z,x

Single Crystal Structure Analysis of Complex [12·thf]

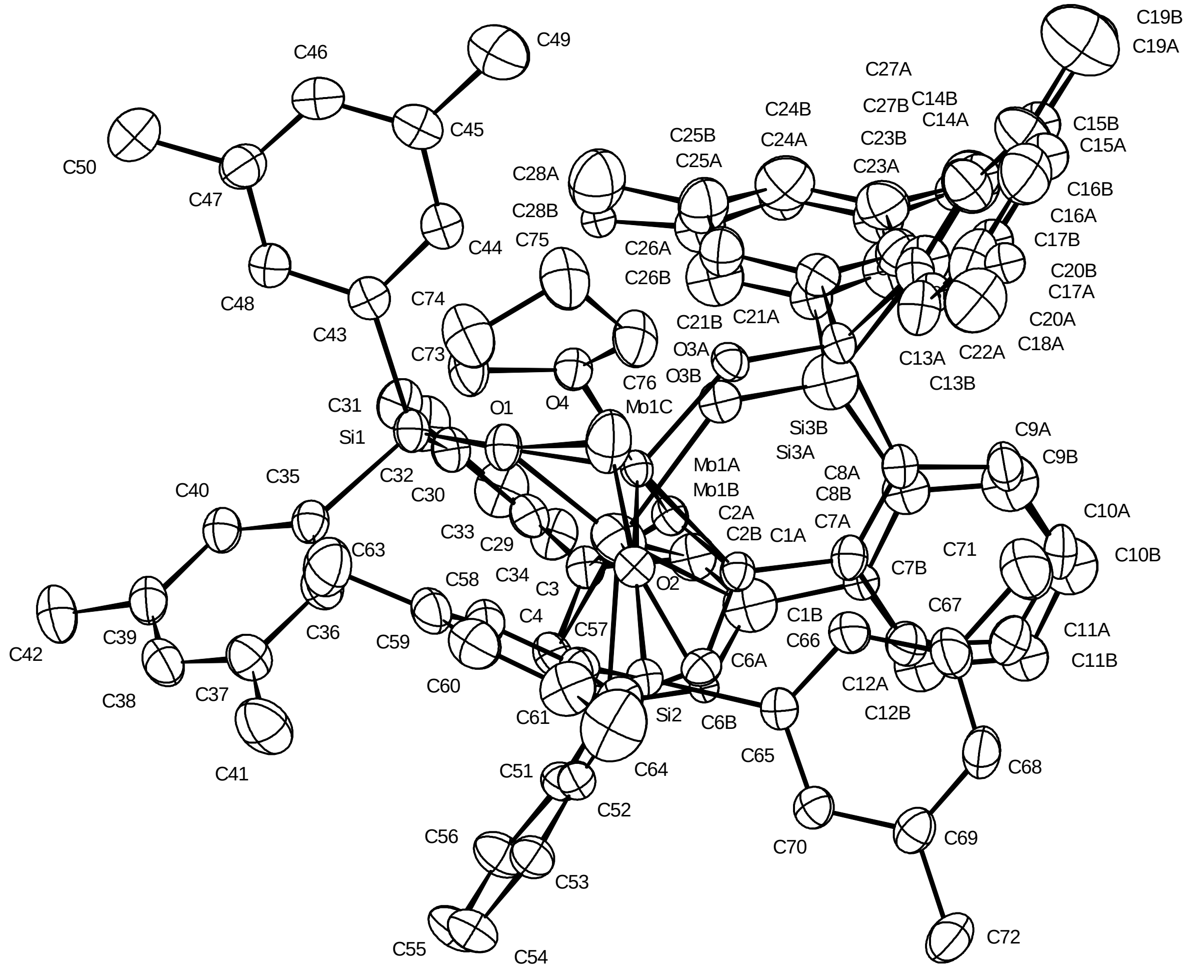

Figure S4. The molecular structure of complex [**12**·thf]; H atoms have been removed for clarity.

X-ray Crystal Structure Analysis of Complex [12·thf**]**: $C_{83.10}$ $H_{94.20}$ Mo $O_{3.90}$ Si_3, M_r = 1335.58 g mol^{-1}, orange-brown plate, crystal size 0.283 x 0.110 x 0.030 mm^3, Monoclinic, space group $P2_1/c$ [14], a = 27.9711(15) Å, b = 16.0465(8) Å, c = 17.2062(9) Å, γ = 106.550(2)°, V = 7402.9(7) Å^3, T = 150(2) K, Z = 4, D_{calc} = 1.198 g·cm^3, λ = 0.71073 Å, $\mu(Mo\text{-}K_\alpha)$ = 0.274 mm^{-1}, Numerical absorption correction ($T_{\min}$ = 0.9491, $T_{\max}$ = 0.9936), 1.979 < θ < 29.130°, Bruker AXS D8-Venture diffractometer with IμS Diamond Mo-anode X-ray source and PHOTON III detector, 458250 measured reflections, 19899 independent reflections, 14760 reflections with $I > 2\sigma(I)$, R_{int} = 0.1172. The structure was solved by *SHELXT* and refined by full-matrix least-squares (*SHELXL*) against F^2 to R_1 = 0.0425 [$I > 2\sigma(I)$], wR_2 = 0.1129 [all data], 866 parameters and 57 restraints.

Full .cif data for the compound are available under CCDC-**2418346**.

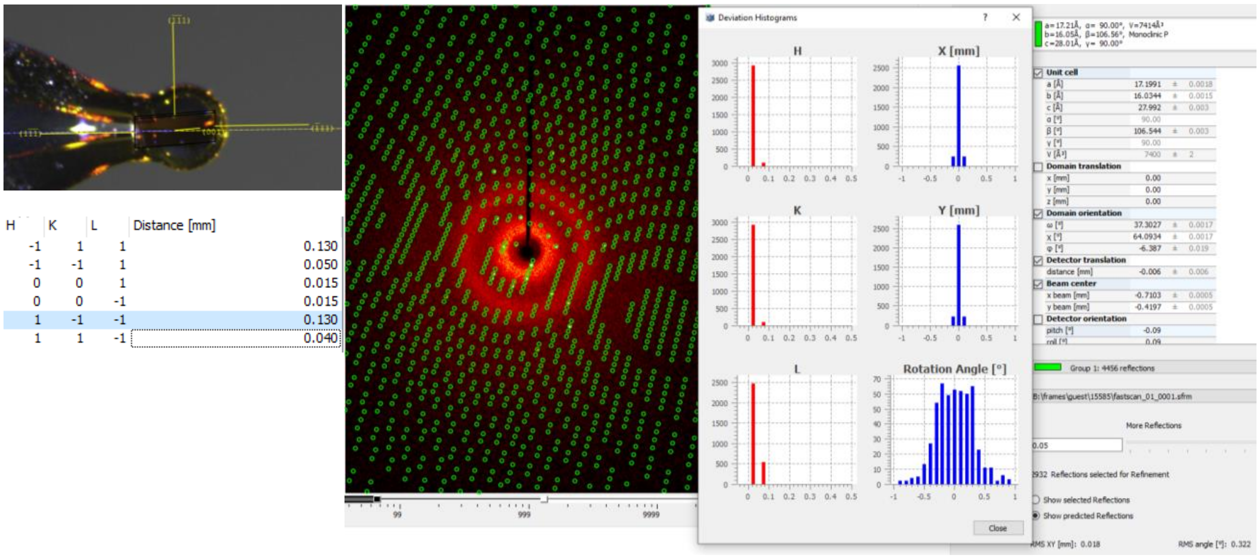

Figure S5. Crystal faces and unit cell determination/refinement of complex [**12**·thf].

INTENSITY STATISTICS FOR DATASET

Resolution	#Data	#Theory	%Complete	Redundancy	Mean I	Mean I/s	Rmerge	Rsigma
Inf - 3.03	309	322	96.0	28.62	89.06	89.70	0.0281	0.0095
3.03 - 2.01	721	721	100.0	31.91	39.19	66.51	0.0391	0.0095
2.01 - 1.59	1033	1033	100.0	30.06	24.29	53.42	0.0543	0.0124
1.59 - 1.38	1074	1074	100.0	29.96	16.95	43.82	0.0698	0.0155
1.38 - 1.25	1052	1052	100.0	29.94	14.03	36.86	0.0829	0.0185
1.25 - 1.16	1033	1033	100.0	28.83	10.21	28.28	0.1089	0.0249
1.16 - 1.09	1059	1059	100.0	28.99	8.22	23.99	0.1291	0.0301
1.09 - 1.04	942	942	100.0	27.60	8.19	22.87	0.1390	0.0324
1.04 - 0.99	1132	1132	100.0	24.45	6.49	18.52	0.1661	0.0415
0.99 - 0.95	1072	1072	100.0	23.30	5.31	15.32	0.1965	0.0508
0.95 - 0.92	960	960	100.0	22.45	4.75	13.57	0.2180	0.0580
0.92 - 0.89	1067	1067	100.0	21.20	4.14	12.16	0.2443	0.0678
0.89 - 0.86	1227	1227	100.0	20.53	3.65	10.52	0.2717	0.0777
0.86 - 0.84	907	907	100.0	19.49	3.48	9.64	0.2969	0.0856
0.84 - 0.82	1007	1007	100.0	19.22	2.92	8.24	0.3305	0.1015
0.82 - 0.80	1110	1110	100.0	18.57	2.96	8.04	0.3465	0.1046
0.80 - 0.78	1235	1235	100.0	17.60	2.39	6.56	0.4099	0.1328
0.78 - 0.77	665	665	100.0	16.56	2.06	5.48	0.4469	0.1565
0.77 - 0.75	1425	1425	100.0	14.68	1.88	4.63	0.4742	0.1900
0.75 - 0.74	764	764	100.0	13.29	1.65	3.86	0.5117	0.2325
0.74 - 0.73	797	797	100.0	12.39	1.44	3.23	0.5534	0.2791
0.83 - 0.73	6498	6498	100.0	15.99	2.18	5.69	0.4169	0.1568
Inf - 0.73	20591	20604	99.9	22.65	9.04	20.35	0.1067	0.0340

Three reflections (-6 3 3; -3 1 3; 0 0 2) showed a high I/σI and have been omitted from the data set prior to the final cycles of refinement. The SUMP instruction was used to combine the individual PARTs of the structure. Some parts of the disorder in PART2 were modeled with SADI and DFIX instructions. The ISOR instruction was applied to some of the terminal methyl groups of the ligand to treat the anisotropic displacement parameters. In addition, a solvent mask (SQUEEZE) was applied to eliminate the residual electron density caused by toluene molecules that could not be refined. Solvent masking was performed by using the BYPASS implementation of Olex2.[3-4] This results in a solvent-accessible void of 1315.53 Å³ (17.8% of the unit cell volume) at a probe radius of 1.2 Å and an approximate lattice spacing of 0.3 Å.

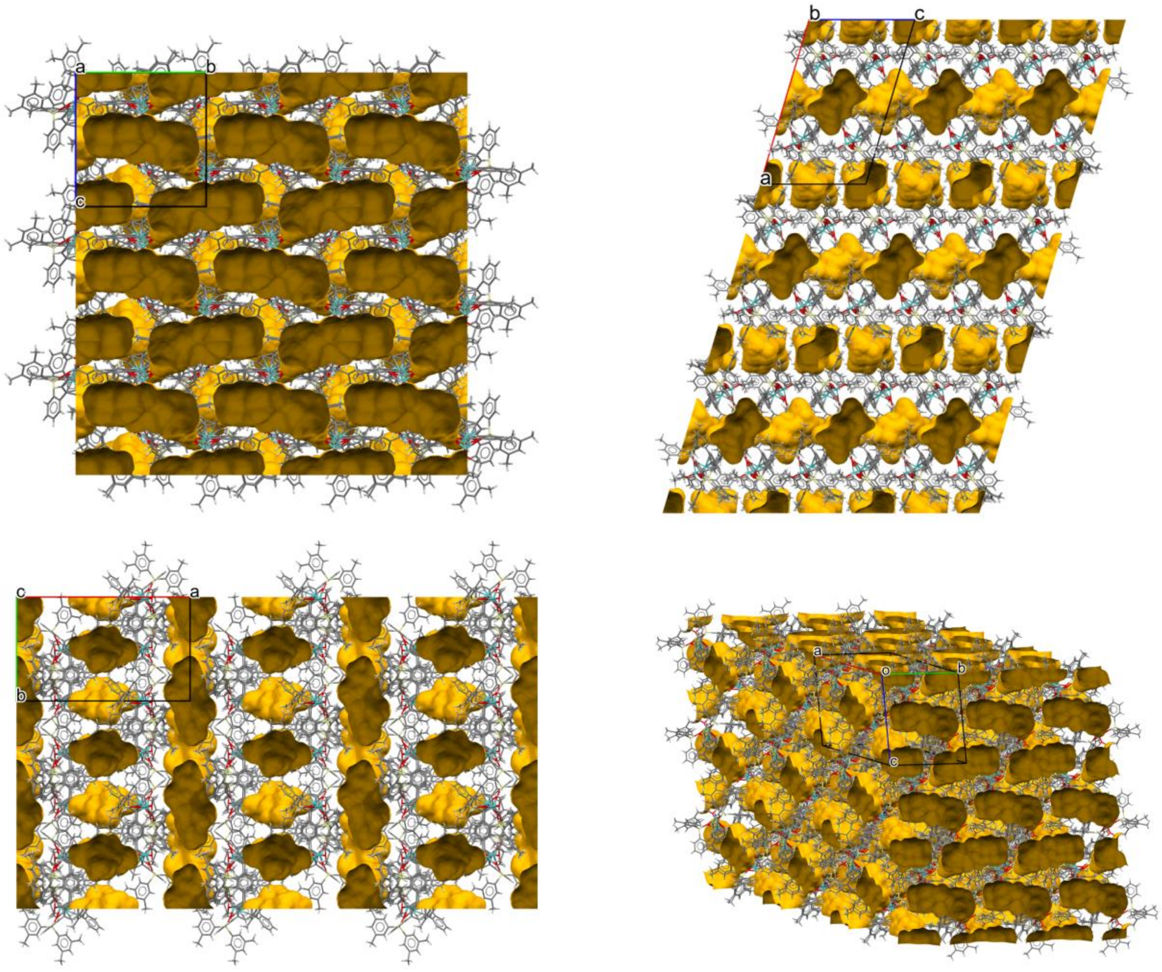

Figure S6. Solvent accessible voids in the crystal structure of [**12**·thf] along the crystallographic a-axis (top left), b-axis (top right), c-axis (bottom left) and in a random orientation (bottom right).

Table S3. Crystal data and structure refinement of complex [**12**·thf].

Identification code	15585	
Empirical formula	$C_{83.10} H_{94.20} Mo O_{3.90} Si_3$	
Color	orange-brown	
Formula weight	1335.58 g·mol^{-1}	
Temperature	150(2) K	
Wavelength	0.71073 Å	
Crystal system	Monoclinic	
Space group	$P2_1/c$, (no. 14)	
Unit cell dimensions	a = 27.9711(15) Å	α = 90°
	b = 16.0465(8) Å	β = 106.550(2)°
	c = 17.2062(9) Å	γ = 90°
Volume	7402.9(7) Å^3	
Z	4	
Density (calculated)	1.198 Mg·m^{-3}	
Absorption coefficient	0.274 mm^{-1}	
F(000)	2832 e	
Crystal size	0.283 x 0.110 x 0.030 mm^3	
θ range for data collection	1.979 to 29.130°.	
Index ranges	-38 ≤ h ≤ 38, -21≤ k ≤ 21, -23 ≤ l ≤ 23	
Reflections collected	458250	
Independent reflections	19899 [R_{int} = 0.1172]	
Reflections with I>2σ(I)	14760	
Completeness to θ = 25.242°	99.9 %	
Absorption correction	Numerical	
Max. and min. transmission	0.9936 and 0.9491	
Refinement method	Full-matrix least-squares on F^2	
Data / restraints / parameters	19899 / 57 / 866	
Goodness-of-fit on F^2	1.024	
Final R indices [I>2σ(I)]	R_1 = 0.0425	wR^2 = 0.1004
R indices (all data)	R_1 = 0.0666	wR^2 = 0.1129
Extinction coefficient	n/a	
Largest diff. peak and hole	0.590 and -0.595 e·Å^{-3}	

Table S4. Bond lengths [Å] and angles [°] of complex [**12**·thf].

Si(1)-O(1)	1.6168(14)	Si(1)-C(30)	1.876(2)
Si(1)-C(35)	1.869(2)	Si(1)-C(43)	1.876(2)
Si(2)-O(2)	1.6349(15)	Si(2)-C(52)	1.892(2)
Si(2)-C(57)	1.872(2)	Si(2)-C(65)	1.868(2)
O(1)-Mo(1A)	1.9162(14)	O(1)-Mo(1B)	1.965(9)
O(1)-Mo(1C)	1.949(5)	O(2)-Mo(1A)	1.9134(14)
O(2)-Mo(1B)	2.163(8)	O(2)-Mo(1C)	1.844(4)
C(3)-C(4)	1.351(3)	C(3)-C(29)	1.494(3)
C(3)-C(2A)	1.453(3)	C(3)-Mo(1B)	2.050(7)
C(3)-C(2B)	1.44(2)	C(4)-H(4)	0.9500
C(4)-C(5)	1.438(3)	C(4)-Mo(1B)	2.274(7)
C(5)-C(51)	1.471(3)	C(5)-C(6A)	1.375(4)
C(5)-Mo(1B)	2.213(8)	C(5)-C(6B)	1.31(3)
C(29)-C(30)	1.404(3)	C(29)-C(34)	1.392(3)
C(30)-C(31)	1.400(3)	C(31)-H(31)	0.9500
C(31)-C(32)	1.388(3)	C(32)-H(32)	0.9500
C(32)-C(33)	1.385(4)	C(33)-H(33)	0.9500
C(33)-C(34)	1.382(3)	C(34)-H(34)	0.9500
C(35)-C(36)	1.395(3)	C(35)-C(40)	1.402(3)
C(36)-H(36)	0.9500	C(36)-C(37)	1.397(3)
C(37)-C(38)	1.388(3)	C(37)-C(41)	1.505(3)
C(38)-H(38)	0.9500	C(38)-C(39)	1.387(3)
C(39)-C(40)	1.391(3)	C(39)-C(42)	1.513(3)
C(40)-H(40)	0.9500	C(41)-H(41A)	0.9800
C(41)-H(41B)	0.9800	C(41)-H(41C)	0.9800
C(42)-H(42A)	0.9800	C(42)-H(42B)	0.9800
C(42)-H(42C)	0.9800	C(43)-C(44)	1.407(3)
C(43)-C(48)	1.395(3)	C(44)-H(44)	0.9500
C(44)-C(45)	1.386(3)	C(45)-C(46)	1.381(4)
C(45)-C(49)	1.517(4)	C(46)-H(46)	0.9500
C(46)-C(47)	1.391(3)	C(47)-C(48)	1.391(3)
C(47)-C(50)	1.510(3)	C(48)-H(48)	0.9500
C(49)-H(49A)	0.9800	C(49)-H(49B)	0.9800
C(49)-H(49C)	0.9800	C(50)-H(50A)	0.9800
C(50)-H(50B)	0.9800	C(50)-H(50C)	0.9800
C(51)-C(52)	1.416(3)	C(51)-C(56)	1.397(3)
C(52)-C(53)	1.401(3)	C(53)-H(53)	0.9500
C(53)-C(54)	1.392(3)	C(54)-H(54)	0.9500
C(54)-C(55)	1.379(3)	C(55)-H(55)	0.9500
C(55)-C(56)	1.383(3)	C(56)-H(56)	0.9500
C(57)-C(58)	1.392(3)	C(57)-C(62)	1.395(3)
C(58)-H(58)	0.9500	C(58)-C(59)	1.388(3)
C(59)-C(60)	1.380(4)	C(59)-C(63)	1.503(3)
C(60)-H(60)	0.9500	C(60)-C(61)	1.379(4)

C(61)-C(62)	1.396(3)	C(61)-C(64)	1.512(4)
C(62)-H(62)	0.9500	C(63)-H(63A)	0.9800
C(63)-H(63B)	0.9800	C(63)-H(63C)	0.9800
C(64)-H(64A)	0.9800	C(64)-H(64B)	0.9800
C(64)-H(64C)	0.9800	C(65)-C(66)	1.390(3)
C(65)-C(70)	1.396(3)	C(66)-H(66)	0.9500
C(66)-C(67)	1.394(3)	C(67)-C(68)	1.384(3)
C(67)-C(71)	1.508(4)	C(68)-H(68)	0.9500
C(68)-C(69)	1.383(3)	C(69)-C(70)	1.390(3)
C(69)-C(72)	1.509(3)	C(70)-H(70)	0.9500
C(71)-H(71A)	0.9800	C(71)-H(71B)	0.9800
C(71)-H(71C)	0.9800	C(72)-H(72A)	0.9800
C(72)-H(72B)	0.9800	C(72)-H(72C)	0.9800
Mo(1A)-O(3A)	1.8979(18)	Mo(1A)-O(4)	2.2524(15)
Mo(1A)-C(1A)	2.276(2)	Mo(1A)-C(2A)	2.224(2)
Si(3A)-O(3A)	1.6308(17)	Si(3A)-C(8A)	1.862(4)
Si(3A)-C(13A)	1.858(3)	Si(3A)-C(21A)	1.860(3)
O(4)-C(73)	1.455(3)	O(4)-C(76)	1.433(3)
C(1A)-C(2A)	1.466(3)	C(1A)-C(6A)	1.429(4)
C(1A)-C(7A)	1.503(3)	C(2A)-H(2A)	1.0000
C(6A)-H(6A)	0.9500	C(7A)-C(8A)	1.403(4)
C(7A)-C(12A)	1.402(4)	C(8A)-C(9A)	1.402(3)
C(9A)-H(9A)	0.9500	C(9A)-C(10A)	1.381(7)
C(10A)-H(10A)	0.9500	C(10A)-C(11A)	1.372(5)
C(11A)-H(11A)	0.9500	C(11A)-C(12A)	1.388(4)
C(12A)-H(12A)	0.9500	C(13A)-C(14A)	1.381(4)
C(13A)-C(18A)	1.406(5)	C(14A)-H(14A)	0.9500
C(14A)-C(15A)	1.406(5)	C(15A)-C(16A)	1.377(6)
C(15A)-C(19A)	1.511(6)	C(16A)-H(16A)	0.9500
C(16A)-C(17A)	1.375(5)	C(17A)-C(18A)	1.388(4)
C(17A)-C(20A)	1.502(5)	C(18A)-H(18A)	0.9500
C(19A)-H(19A)	0.9800	C(19A)-H(19B)	0.9800
C(19A)-H(19C)	0.9800	C(20A)-H(20A)	0.9800
C(20A)-H(20B)	0.9800	C(20A)-H(20C)	0.9800
C(21A)-C(22A)	1.397(3)	C(21A)-C(26A)	1.394(4)
C(22A)-H(22A)	0.9500	C(22A)-C(23A)	1.390(4)
C(23A)-C(24A)	1.388(4)	C(23A)-C(27A)	1.501(4)
C(24A)-H(24A)	0.9500	C(24A)-C(25A)	1.377(4)
C(25A)-C(26A)	1.390(4)	C(25A)-C(28A)	1.519(5)
C(26A)-H(26A)	0.9500	C(27A)-H(27A)	0.9800
C(27A)-H(27C)	0.9800	C(27A)-H(27B)	0.9800
C(28A)-H(28A)	0.9800	C(28A)-H(28C)	0.9800
C(28A)-H(28B)	0.9800	C(73)-H(73A)	0.9900
C(73)-H(73B)	0.9900	C(73)-C(74)	1.489(4)
C(74)-H(74B)	0.9900	C(74)-H(74A)	0.9900
C(74)-C(75)	1.500(4)	C(75)-H(75A)	0.9900
C(75)-H(75B)	0.9900	C(75)-C(76)	1.514(4)

C(76)-H(76B)	0.9900	C(76)-H(76A)	0.9900
Mo(1B)-O(3B)	2.34(2)	Mo(1B)-C(1B)	2.22(3)
Mo(1B)-C(2B)	2.24(3)	Mo(1B)-C(6B)	2.31(3)
Si(3B)-O(3B)	1.68(2)	Si(3B)-C(8B)	1.807(9)
Si(3B)-C(13B)	2.299(17)	Si(3B)-C(21B)	2.01(3)
C(1B)-C(2B)	1.40(4)	C(1B)-C(6B)	1.46(4)
C(1B)-C(7B)	1.52(3)	C(2B)-H(2B)	1.0000
C(6B)-H(6B)	1.0000	C(12B)-H(12B)	0.9500
C(12B)-C(11B)	1.3900	C(12B)-C(7B)	1.3900
C(11B)-H(11B)	0.9500	C(11B)-C(10B)	1.3900
C(10B)-H(10B)	0.9500	C(10B)-C(9B)	1.3900
C(9B)-H(9B)	0.9500	C(9B)-C(8B)	1.3900
C(8B)-C(7B)	1.3900	C(13B)-C(14B)	1.3900
C(13B)-C(18B)	1.3900	C(14B)-H(14B)	0.9500
C(14B)-C(15B)	1.3900	C(15B)-C(16B)	1.3900
C(15B)-C(19B)	1.503(9)	C(16B)-H(16B)	0.9500
C(16B)-C(17B)	1.3900	C(17B)-C(18B)	1.3900
C(17B)-C(20B)	1.520(9)	C(18B)-H(18B)	0.9500
C(19B)-H(19D)	0.9800	C(19B)-H(19E)	0.9800
C(19B)-H(19F)	0.9800	C(20B)-H(20E)	0.9800
C(20B)-H(20D)	0.9800	C(20B)-H(20F)	0.9800
C(21B)-C(26B)	1.3900	C(21B)-C(88)	1.3900
C(26B)-H(26B)	0.9500	C(26B)-C(25B)	1.3900
C(25B)-C(24B)	1.3900	C(25B)-C(28B)	1.46(3)
C(24B)-H(24B)	0.9500	C(24B)-C(23B)	1.3900
C(23B)-C(88)	1.3900	C(23B)-C(27B)	1.569(17)
C(88)-H(88)	0.9500	C(27B)-H(27E)	0.9800
C(27B)-H(27D)	0.9800	C(27B)-H(27F)	0.9800
C(28B)-H(28E)	0.9800	C(28B)-H(28F)	0.9800
C(28B)-H(28D)	0.9800		

O(1)-Si(1)-C(30)	112.39(8)	O(1)-Si(1)-C(35)	108.03(8)
O(1)-Si(1)-C(43)	105.88(8)	C(30)-Si(1)-C(43)	109.67(9)
C(35)-Si(1)-C(30)	109.41(9)	C(35)-Si(1)-C(43)	111.43(9)
O(2)-Si(2)-C(52)	114.12(8)	O(2)-Si(2)-C(57)	104.75(8)
O(2)-Si(2)-C(65)	109.92(8)	C(57)-Si(2)-C(52)	106.04(9)
C(65)-Si(2)-C(52)	111.90(9)	C(65)-Si(2)-C(57)	109.73(9)
Si(1)-O(1)-Mo(1A)	160.29(9)	Si(1)-O(1)-Mo(1B)	130.7(2)
Si(1)-O(1)-Mo(1C)	169.2(2)	Si(2)-O(2)-Mo(1A)	157.11(9)
Si(2)-O(2)-Mo(1B)	124.6(2)	Si(2)-O(2)-Mo(1C)	173.79(17)
C(4)-C(3)-C(29)	121.61(18)	C(4)-C(3)-C(2A)	119.35(18)
C(4)-C(3)-Mo(1B)	81.1(3)	C(4)-C(3)-C(2B)	114.2(10)
C(29)-C(3)-Mo(1B)	136.8(3)	C(2A)-C(3)-C(29)	118.59(18)
C(2B)-C(3)-C(29)	116.1(10)	C(2B)-C(3)-Mo(1B)	77.7(10)
C(3)-C(4)-H(4)	119.6	C(3)-C(4)-C(5)	120.83(18)
C(3)-C(4)-Mo(1B)	63.0(2)	C(5)-C(4)-H(4)	119.6
C(5)-C(4)-Mo(1B)	69.0(2)	Mo(1B)-C(4)-H(4)	145.3

C(4)-C(5)-C(51)	121.40(18)	C(4)-C(5)-Mo(1B)	73.6(2)
C(51)-C(5)-Mo(1B)	129.5(2)	C(6A)-C(5)-C(4)	118.7(2)
C(6A)-C(5)-C(51)	119.6(2)	C(6B)-C(5)-C(4)	119.0(14)
C(6B)-C(5)-C(51)	118.7(14)	C(6B)-C(5)-Mo(1B)	77.1(12)
C(30)-C(29)-C(3)	119.93(17)	C(34)-C(29)-C(3)	120.16(19)
C(34)-C(29)-C(30)	119.91(19)	C(29)-C(30)-Si(1)	120.15(15)
C(31)-C(30)-Si(1)	121.53(16)	C(31)-C(30)-C(29)	118.20(19)
C(30)-C(31)-H(31)	119.3	C(32)-C(31)-C(30)	121.5(2)
C(32)-C(31)-H(31)	119.3	C(31)-C(32)-H(32)	120.2
C(33)-C(32)-C(31)	119.5(2)	C(33)-C(32)-H(32)	120.2
C(32)-C(33)-H(33)	120.0	C(34)-C(33)-C(32)	120.0(2)
C(34)-C(33)-H(33)	120.0	C(29)-C(34)-H(34)	119.6
C(33)-C(34)-C(29)	120.9(2)	C(33)-C(34)-H(34)	119.6
C(36)-C(35)-Si(1)	120.12(15)	C(36)-C(35)-C(40)	117.46(19)
C(40)-C(35)-Si(1)	122.39(16)	C(35)-C(36)-H(36)	119.0
C(35)-C(36)-C(37)	122.1(2)	C(37)-C(36)-H(36)	119.0
C(36)-C(37)-C(41)	120.7(2)	C(38)-C(37)-C(36)	118.0(2)
C(38)-C(37)-C(41)	121.2(2)	C(37)-C(38)-H(38)	118.9
C(39)-C(38)-C(37)	122.2(2)	C(39)-C(38)-H(38)	118.9
C(38)-C(39)-C(40)	118.2(2)	C(38)-C(39)-C(42)	120.1(2)
C(40)-C(39)-C(42)	121.5(2)	C(35)-C(40)-H(40)	119.0
C(39)-C(40)-C(35)	122.0(2)	C(39)-C(40)-H(40)	119.0
C(37)-C(41)-H(41A)	109.5	C(37)-C(41)-H(41B)	109.5
C(37)-C(41)-H(41C)	109.5	H(41A)-C(41)-H(41B)	109.5
H(41A)-C(41)-H(41C)	109.5	H(41B)-C(41)-H(41C)	109.5
C(39)-C(42)-H(42A)	109.5	C(39)-C(42)-H(42B)	109.5
C(39)-C(42)-H(42C)	109.5	H(42A)-C(42)-H(42B)	109.5
H(42A)-C(42)-H(42C)	109.5	H(42B)-C(42)-H(42C)	109.5
C(44)-C(43)-Si(1)	117.82(16)	C(48)-C(43)-Si(1)	125.17(16)
C(48)-C(43)-C(44)	116.9(2)	C(43)-C(44)-H(44)	118.9
C(45)-C(44)-C(43)	122.3(2)	C(45)-C(44)-H(44)	118.9
C(44)-C(45)-C(49)	120.4(2)	C(46)-C(45)-C(44)	118.4(2)
C(46)-C(45)-C(49)	121.2(2)	C(45)-C(46)-H(46)	119.1
C(45)-C(46)-C(47)	121.9(2)	C(47)-C(46)-H(46)	119.1
C(46)-C(47)-C(50)	121.0(2)	C(48)-C(47)-C(46)	118.3(2)
C(48)-C(47)-C(50)	120.6(2)	C(43)-C(48)-H(48)	118.9
C(47)-C(48)-C(43)	122.2(2)	C(47)-C(48)-H(48)	118.9
C(45)-C(49)-H(49A)	109.5	C(45)-C(49)-H(49B)	109.5
C(45)-C(49)-H(49C)	109.5	H(49A)-C(49)-H(49B)	109.5
H(49A)-C(49)-H(49C)	109.5	H(49B)-C(49)-H(49C)	109.5
C(47)-C(50)-H(50A)	109.5	C(47)-C(50)-H(50B)	109.5
C(47)-C(50)-H(50C)	109.5	H(50A)-C(50)-H(50B)	109.5
H(50A)-C(50)-H(50C)	109.5	H(50B)-C(50)-H(50C)	109.5
C(52)-C(51)-C(5)	119.31(17)	C(56)-C(51)-C(5)	121.24(19)
C(56)-C(51)-C(52)	119.39(19)	C(51)-C(52)-Si(2)	124.63(15)
C(53)-C(52)-Si(2)	117.58(15)	C(53)-C(52)-C(51)	117.64(18)
C(52)-C(53)-H(53)	118.9	C(54)-C(53)-C(52)	122.2(2)

C(54)-C(53)-H(53)	118.9	C(53)-C(54)-H(54)	120.3
C(55)-C(54)-C(53)	119.4(2)	C(55)-C(54)-H(54)	120.3
C(54)-C(55)-H(55)	120.0	C(54)-C(55)-C(56)	119.9(2)
C(56)-C(55)-H(55)	120.0	C(51)-C(56)-H(56)	119.3
C(55)-C(56)-C(51)	121.4(2)	C(55)-C(56)-H(56)	119.3
C(58)-C(57)-Si(2)	118.35(16)	C(58)-C(57)-C(62)	117.35(19)
C(62)-C(57)-Si(2)	124.30(16)	C(57)-C(58)-H(58)	118.8
C(59)-C(58)-C(57)	122.4(2)	C(59)-C(58)-H(58)	118.8
C(58)-C(59)-C(63)	120.2(2)	C(60)-C(59)-C(58)	118.1(2)
C(60)-C(59)-C(63)	121.7(2)	C(59)-C(60)-H(60)	118.9
C(61)-C(60)-C(59)	122.2(2)	C(61)-C(60)-H(60)	118.9
C(60)-C(61)-C(62)	118.2(2)	C(60)-C(61)-C(64)	121.5(2)
C(62)-C(61)-C(64)	120.3(3)	C(57)-C(62)-C(61)	121.7(2)
C(57)-C(62)-H(62)	119.1	C(61)-C(62)-H(62)	119.1
C(59)-C(63)-H(63A)	109.5	C(59)-C(63)-H(63B)	109.5
C(59)-C(63)-H(63C)	109.5	H(63A)-C(63)-H(63B)	109.5
H(63A)-C(63)-H(63C)	109.5	H(63B)-C(63)-H(63C)	109.5
C(61)-C(64)-H(64A)	109.5	C(61)-C(64)-H(64B)	109.5
C(61)-C(64)-H(64C)	109.5	H(64A)-C(64)-H(64B)	109.5
H(64A)-C(64)-H(64C)	109.5	H(64B)-C(64)-H(64C)	109.5
C(66)-C(65)-Si(2)	120.35(16)	C(66)-C(65)-C(70)	117.88(18)
C(70)-C(65)-Si(2)	121.76(15)	C(65)-C(66)-H(66)	119.1
C(65)-C(66)-C(67)	121.9(2)	C(67)-C(66)-H(66)	119.1
C(66)-C(67)-C(71)	120.9(2)	C(68)-C(67)-C(66)	118.2(2)
C(68)-C(67)-C(71)	120.9(2)	C(67)-C(68)-H(68)	119.1
C(69)-C(68)-C(67)	121.9(2)	C(69)-C(68)-H(68)	119.1
C(68)-C(69)-C(70)	118.6(2)	C(68)-C(69)-C(72)	120.6(2)
C(70)-C(69)-C(72)	120.8(2)	C(65)-C(70)-H(70)	119.2
C(69)-C(70)-C(65)	121.57(19)	C(69)-C(70)-H(70)	119.2
C(67)-C(71)-H(71A)	109.5	C(67)-C(71)-H(71B)	109.5
C(67)-C(71)-H(71C)	109.5	H(71A)-C(71)-H(71B)	109.5
H(71A)-C(71)-H(71C)	109.5	H(71B)-C(71)-H(71C)	109.5
C(69)-C(72)-H(72A)	109.5	C(69)-C(72)-H(72B)	109.5
C(69)-C(72)-H(72C)	109.5	H(72A)-C(72)-H(72B)	109.5
H(72A)-C(72)-H(72C)	109.5	H(72B)-C(72)-H(72C)	109.5
O(1)-Mo(1A)-O(4)	79.18(6)	O(1)-Mo(1A)-C(1A)	117.11(7)
O(1)-Mo(1A)-C(2A)	84.43(7)	O(2)-Mo(1A)-O(1)	109.68(6)
O(2)-Mo(1A)-O(4)	82.86(6)	O(2)-Mo(1A)-C(1A)	88.95(7)
O(2)-Mo(1A)-C(2A)	119.45(7)	O(3A)-Mo(1A)-O(1)	118.83(7)
O(3A)-Mo(1A)-O(2)	124.30(7)	O(3A)-Mo(1A)-O(4)	81.01(7)
O(3A)-Mo(1A)-C(1A)	92.02(8)	O(3A)-Mo(1A)-C(2A)	91.84(7)
O(4)-Mo(1A)-C(1A)	163.55(7)	C(2A)-Mo(1A)-O(4)	155.98(7)
C(2A)-Mo(1A)-C(1A)	38.01(8)	O(3A)-Si(3A)-C(8A)	109.01(11)
O(3A)-Si(3A)-C(13A)	105.88(14)	O(3A)-Si(3A)-C(21A)	108.88(12)
C(13A)-Si(3A)-C(8A)	108.50(13)	C(13A)-Si(3A)-C(21A)	113.48(18)
C(21A)-Si(3A)-C(8A)	110.90(15)	Si(3A)-O(3A)-Mo(1A)	136.20(12)
C(73)-O(4)-Mo(1A)	124.16(13)	C(76)-O(4)-Mo(1A)	120.04(14)

C(76)-O(4)-C(73)	109.51(17)	C(2A)-C(1A)-Mo(1A)	69.09(12)
C(2A)-C(1A)-C(7A)	121.83(19)	C(6A)-C(1A)-Mo(1A)	100.43(17)
C(6A)-C(1A)-C(2A)	115.0(2)	C(6A)-C(1A)-C(7A)	117.3(2)
C(7A)-C(1A)-Mo(1A)	122.19(17)	C(3)-C(2A)-Mo(1A)	111.90(13)
C(3)-C(2A)-C(1A)	119.29(19)	C(3)-C(2A)-H(2A)	115.3
Mo(1A)-C(2A)-H(2A)	115.3	C(1A)-C(2A)-Mo(1A)	72.91(12)
C(1A)-C(2A)-H(2A)	115.3	C(5)-C(6A)-C(1A)	123.0(3)
C(5)-C(6A)-H(6A)	118.5	C(1A)-C(6A)-H(6A)	118.5
C(8A)-C(7A)-C(1A)	125.6(2)	C(12A)-C(7A)-C(1A)	116.2(2)
C(12A)-C(7A)-C(8A)	118.2(2)	C(7A)-C(8A)-Si(3A)	124.95(19)
C(9A)-C(8A)-Si(3A)	116.8(3)	C(9A)-C(8A)-C(7A)	118.3(4)
C(8A)-C(9A)-H(9A)	118.5	C(10A)-C(9A)-C(8A)	123.0(4)
C(10A)-C(9A)-H(9A)	118.5	C(9A)-C(10A)-H(10A)	120.8
C(11A)-C(10A)-C(9A)	118.4(3)	C(11A)-C(10A)-H(10A)	120.8
C(10A)-C(11A)-H(11A)	119.8	C(10A)-C(11A)-C(12A)	120.3(4)
C(12A)-C(11A)-H(11A)	119.8	C(7A)-C(12A)-H(12A)	119.1
C(11A)-C(12A)-C(7A)	121.9(3)	C(11A)-C(12A)-H(12A)	119.1
C(14A)-C(13A)-Si(3A)	124.6(3)	C(14A)-C(13A)-C(18A)	117.6(3)
C(18A)-C(13A)-Si(3A)	117.8(3)	C(13A)-C(14A)-H(14A)	119.4
C(13A)-C(14A)-C(15A)	121.3(4)	C(15A)-C(14A)-H(14A)	119.4
C(14A)-C(15A)-C(19A)	120.3(4)	C(16A)-C(15A)-C(14A)	118.4(3)
C(16A)-C(15A)-C(19A)	121.2(4)	C(15A)-C(16A)-H(16A)	118.7
C(17A)-C(16A)-C(15A)	122.7(3)	C(17A)-C(16A)-H(16A)	118.7
C(16A)-C(17A)-C(18A)	117.6(4)	C(16A)-C(17A)-C(20A)	121.6(3)
C(18A)-C(17A)-C(20A)	120.8(4)	C(13A)-C(18A)-H(18A)	118.8
C(17A)-C(18A)-C(13A)	122.3(4)	C(17A)-C(18A)-H(18A)	118.8
C(15A)-C(19A)-H(19A)	109.5	C(15A)-C(19A)-H(19B)	109.5
C(15A)-C(19A)-H(19C)	109.5	H(19A)-C(19A)-H(19B)	109.5
H(19A)-C(19A)-H(19C)	109.5	H(19B)-C(19A)-H(19C)	109.5
C(17A)-C(20A)-H(20A)	109.5	C(17A)-C(20A)-H(20B)	109.5
C(17A)-C(20A)-H(20C)	109.5	H(20A)-C(20A)-H(20B)	109.5
H(20A)-C(20A)-H(20C)	109.5	H(20B)-C(20A)-H(20C)	109.5
C(22A)-C(21A)-Si(3A)	121.7(2)	C(26A)-C(21A)-Si(3A)	120.8(2)
C(26A)-C(21A)-C(22A)	117.4(2)	C(21A)-C(22A)-H(22A)	119.0
C(23A)-C(22A)-C(21A)	122.1(3)	C(23A)-C(22A)-H(22A)	119.0
C(22A)-C(23A)-C(27A)	119.9(3)	C(24A)-C(23A)-C(22A)	117.5(3)
C(24A)-C(23A)-C(27A)	122.6(3)	C(23A)-C(24A)-H(24A)	118.4
C(25A)-C(24A)-C(23A)	123.2(3)	C(25A)-C(24A)-H(24A)	118.4
C(24A)-C(25A)-C(26A)	117.4(3)	C(24A)-C(25A)-C(28A)	122.1(3)
C(26A)-C(25A)-C(28A)	120.5(3)	C(21A)-C(26A)-H(26A)	118.8
C(25A)-C(26A)-C(21A)	122.5(2)	C(25A)-C(26A)-H(26A)	118.8
C(23A)-C(27A)-H(27A)	109.5	C(23A)-C(27A)-H(27C)	109.5
C(23A)-C(27A)-H(27B)	109.5	H(27A)-C(27A)-H(27C)	109.5
H(27A)-C(27A)-H(27B)	109.5	H(27C)-C(27A)-H(27B)	109.5
C(25A)-C(28A)-H(28A)	109.5	C(25A)-C(28A)-H(28C)	109.5
C(25A)-C(28A)-H(28B)	109.5	H(28A)-C(28A)-H(28C)	109.5
H(28A)-C(28A)-H(28B)	109.5	H(28C)-C(28A)-H(28B)	109.5

O(4)-C(73)-H(73A)	110.6	O(4)-C(73)-H(73B)	110.6
O(4)-C(73)-C(74)	105.8(2)	H(73A)-C(73)-H(73B)	108.7
C(74)-C(73)-H(73A)	110.6	C(74)-C(73)-H(73B)	110.6
C(73)-C(74)-H(74B)	110.9	C(73)-C(74)-H(74A)	110.9
C(73)-C(74)-C(75)	104.0(2)	H(74B)-C(74)-H(74A)	109.0
C(75)-C(74)-H(74B)	110.9	C(75)-C(74)-H(74A)	110.9
C(74)-C(75)-H(75A)	111.4	C(74)-C(75)-H(75B)	111.4
C(74)-C(75)-C(76)	101.8(2)	H(75A)-C(75)-H(75B)	109.3
C(76)-C(75)-H(75A)	111.4	C(76)-C(75)-H(75B)	111.4
O(4)-C(76)-C(75)	103.8(2)	O(4)-C(76)-H(76B)	111.0
O(4)-C(76)-H(76A)	111.0	C(75)-C(76)-H(76B)	111.0
C(75)-C(76)-H(76A)	111.0	H(76B)-C(76)-H(76A)	109.0
O(1)-Mo(1B)-O(2)	98.5(3)	O(1)-Mo(1B)-C(3)	88.8(3)
O(1)-Mo(1B)-C(4)	98.2(3)	O(1)-Mo(1B)-C(5)	124.7(4)
O(1)-Mo(1B)-O(3B)	87.4(5)	O(1)-Mo(1B)-C(1B)	152.1(9)
O(1)-Mo(1B)-C(2B)	116.3(7)	O(1)-Mo(1B)-C(6B)	158.3(9)
O(2)-Mo(1B)-C(4)	122.7(4)	O(2)-Mo(1B)-C(5)	90.3(3)
O(2)-Mo(1B)-O(3B)	82.4(5)	O(2)-Mo(1B)-C(1B)	107.7(9)
O(2)-Mo(1B)-C(2B)	144.2(8)	O(2)-Mo(1B)-C(6B)	85.8(8)
C(3)-Mo(1B)-O(2)	158.6(4)	C(3)-Mo(1B)-C(4)	35.94(13)
C(3)-Mo(1B)-C(5)	69.2(2)	C(3)-Mo(1B)-O(3B)	118.1(6)
C(3)-Mo(1B)-C(1B)	70.0(9)	C(3)-Mo(1B)-C(2B)	38.9(6)
C(3)-Mo(1B)-C(6B)	80.4(8)	C(4)-Mo(1B)-O(3B)	152.5(6)
C(4)-Mo(1B)-C(6B)	62.3(8)	C(5)-Mo(1B)-C(4)	37.36(14)
C(5)-Mo(1B)-O(3B)	147.9(6)	C(5)-Mo(1B)-C(1B)	65.5(8)
C(5)-Mo(1B)-C(2B)	77.0(7)	C(5)-Mo(1B)-C(6B)	33.6(8)
C(1B)-Mo(1B)-C(4)	75.5(9)	C(1B)-Mo(1B)-O(3B)	87.0(10)
C(1B)-Mo(1B)-C(2B)	36.6(10)	C(1B)-Mo(1B)-C(6B)	37.6(11)
C(2B)-Mo(1B)-C(4)	62.6(7)	C(2B)-Mo(1B)-O(3B)	90.8(8)
C(2B)-Mo(1B)-C(6B)	65.2(10)	C(6B)-Mo(1B)-O(3B)	114.4(10)
O(3B)-Si(3B)-C(8B)	116.2(11)	O(3B)-Si(3B)-C(13B)	112.9(10)
O(3B)-Si(3B)-C(21B)	110.3(11)	C(8B)-Si(3B)-C(13B)	110.4(9)
C(8B)-Si(3B)-C(21B)	111.5(14)	C(21B)-Si(3B)-C(13B)	93.3(9)
Si(3B)-O(3B)-Mo(1B)	122.8(10)	C(2B)-C(1B)-Mo(1B)	72.7(17)
C(2B)-C(1B)-C(6B)	118(3)	C(2B)-C(1B)-C(7B)	119(3)
C(6B)-C(1B)-Mo(1B)	74.6(17)	C(6B)-C(1B)-C(7B)	122(3)
C(7B)-C(1B)-Mo(1B)	131(2)	C(3)-C(2B)-Mo(1B)	63.4(10)
C(3)-C(2B)-H(2B)	117.8	Mo(1B)-C(2B)-H(2B)	117.8
C(1B)-C(2B)-C(3)	119(2)	C(1B)-C(2B)-Mo(1B)	70.7(17)
C(1B)-C(2B)-H(2B)	117.8	C(5)-C(6B)-Mo(1B)	69.3(12)
C(5)-C(6B)-C(1B)	120(3)	C(5)-C(6B)-H(6B)	118.6
Mo(1B)-C(6B)-H(6B)	118.6	C(1B)-C(6B)-Mo(1B)	67.9(17)
C(1B)-C(6B)-H(6B)	118.6	C(11B)-C(12B)-H(12B)	120.0
C(11B)-C(12B)-C(7B)	120.0	C(7B)-C(12B)-H(12B)	120.0
C(12B)-C(11B)-H(11B)	120.0	C(12B)-C(11B)-C(10B)	120.0
C(10B)-C(11B)-H(11B)	120.0	C(11B)-C(10B)-H(10B)	120.0
C(9B)-C(10B)-C(11B)	120.0	C(9B)-C(10B)-H(10B)	120.0

C(10B)-C(9B)-H(9B)	120.0	C(8B)-C(9B)-C(10B)	120.0
C(8B)-C(9B)-H(9B)	120.0	C(9B)-C(8B)-Si(3B)	119.2(7)
C(7B)-C(8B)-Si(3B)	120.8(7)	C(7B)-C(8B)-C(9B)	120.0
C(12B)-C(7B)-C(1B)	112.1(15)	C(8B)-C(7B)-C(1B)	127.9(15)
C(8B)-C(7B)-C(12B)	120.0	C(14B)-C(13B)-Si(3B)	135.2(9)
C(14B)-C(13B)-C(18B)	120.0	C(18B)-C(13B)-Si(3B)	104.5(9)
C(13B)-C(14B)-H(14B)	120.0	C(13B)-C(14B)-C(15B)	120.0
C(15B)-C(14B)-H(14B)	120.0	C(14B)-C(15B)-C(19B)	114.1(11)
C(16B)-C(15B)-C(14B)	120.0	C(16B)-C(15B)-C(19B)	125.8(11)
C(15B)-C(16B)-H(16B)	120.0	C(15B)-C(16B)-C(17B)	120.0
C(17B)-C(16B)-H(16B)	120.0	C(16B)-C(17B)-C(18B)	120.0
C(16B)-C(17B)-C(20B)	112.9(11)	C(18B)-C(17B)-C(20B)	127.1(11)
C(13B)-C(18B)-H(18B)	120.0	C(17B)-C(18B)-C(13B)	120.0
C(17B)-C(18B)-H(18B)	120.0	C(15B)-C(19B)-H(19D)	109.5
C(15B)-C(19B)-H(19E)	109.5	C(15B)-C(19B)-H(19F)	109.5
H(19D)-C(19B)-H(19E)	109.5	H(19D)-C(19B)-H(19F)	109.5
H(19E)-C(19B)-H(19F)	109.5	C(17B)-C(20B)-H(20E)	109.5
C(17B)-C(20B)-H(20D)	109.5	C(17B)-C(20B)-H(20F)	109.5
H(20E)-C(20B)-H(20D)	109.5	H(20E)-C(20B)-H(20F)	109.5
H(20D)-C(20B)-H(20F)	109.5	C(26B)-C(21B)-Si(3B)	119.2(12)
C(26B)-C(21B)-C(88)	120.0	C(88)-C(21B)-Si(3B)	120.6(12)
C(21B)-C(26B)-H(26B)	120.0	C(25B)-C(26B)-C(21B)	120.0
C(25B)-C(26B)-H(26B)	120.0	C(26B)-C(25B)-C(24B)	120.0
C(26B)-C(25B)-C(28B)	118.3(17)	C(24B)-C(25B)-C(28B)	121.1(16)
C(25B)-C(24B)-H(24B)	120.0	C(23B)-C(24B)-C(25B)	120.0
C(23B)-C(24B)-H(24B)	120.0	C(24B)-C(23B)-C(27B)	108.8(9)
C(88)-C(23B)-C(24B)	120.0	C(88)-C(23B)-C(27B)	130.5(11)
C(21B)-C(88)-H(88)	120.0	C(23B)-C(88)-C(21B)	120.0
C(23B)-C(88)-H(88)	120.0	C(23B)-C(27B)-H(27E)	109.5
C(23B)-C(27B)-H(27D)	109.5	C(23B)-C(27B)-H(27F)	109.5
H(27E)-C(27B)-H(27D)	109.5	H(27E)-C(27B)-H(27F)	109.5
H(27D)-C(27B)-H(27F)	109.5	C(25B)-C(28B)-H(28E)	109.5
C(25B)-C(28B)-H(28F)	109.5	C(25B)-C(28B)-H(28D)	109.5
H(28E)-C(28B)-H(28F)	109.5	H(28E)-C(28B)-H(28D)	109.5
H(28F)-C(28B)-H(28D)	109.5	O(2)-Mo(1C)-O(1)	111.2(3)

Crystallographic Discussion of the Structure of Complex [12·thf]

Determining the crystal structure of this complex proved challenging. Several crystals from different preparations were measured. The best result is discussed below.

During sample preparation on a transmission microscope with a polarisation filter under an inert stream of N_2 gas, it was noticed that the crystals changed over time and become amorphous. Initially, many crystallites were intact, as manifested in a clear structure, color and optical transparency. After some time, many fine cracks are formed and the crystallites became opaque.

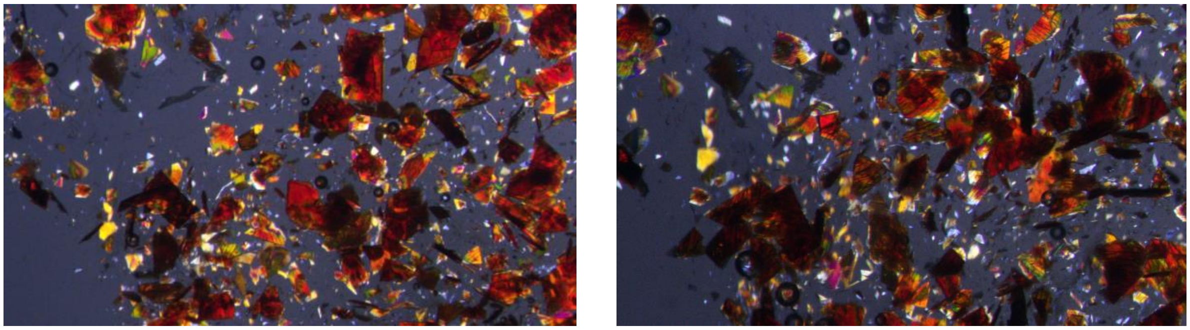

Figure S7. Fresh crystals immediately after removal from the Schlenk flask (left) and crystals after approximately 30 min under the microscope (right).

After data collection, the refinement of the structures always revealed the same structural motif. In addition to the main component, high residual electron densities were found in the region of the central Mo atom. Residual electron density was also found on one of the side arms of the tripodal ligand. After splitting the central metal atom into several PARTs, the residual electron densities at the ligand were particularly pronounced. Analysis of the data revealed no evidence of twinning or other problems.

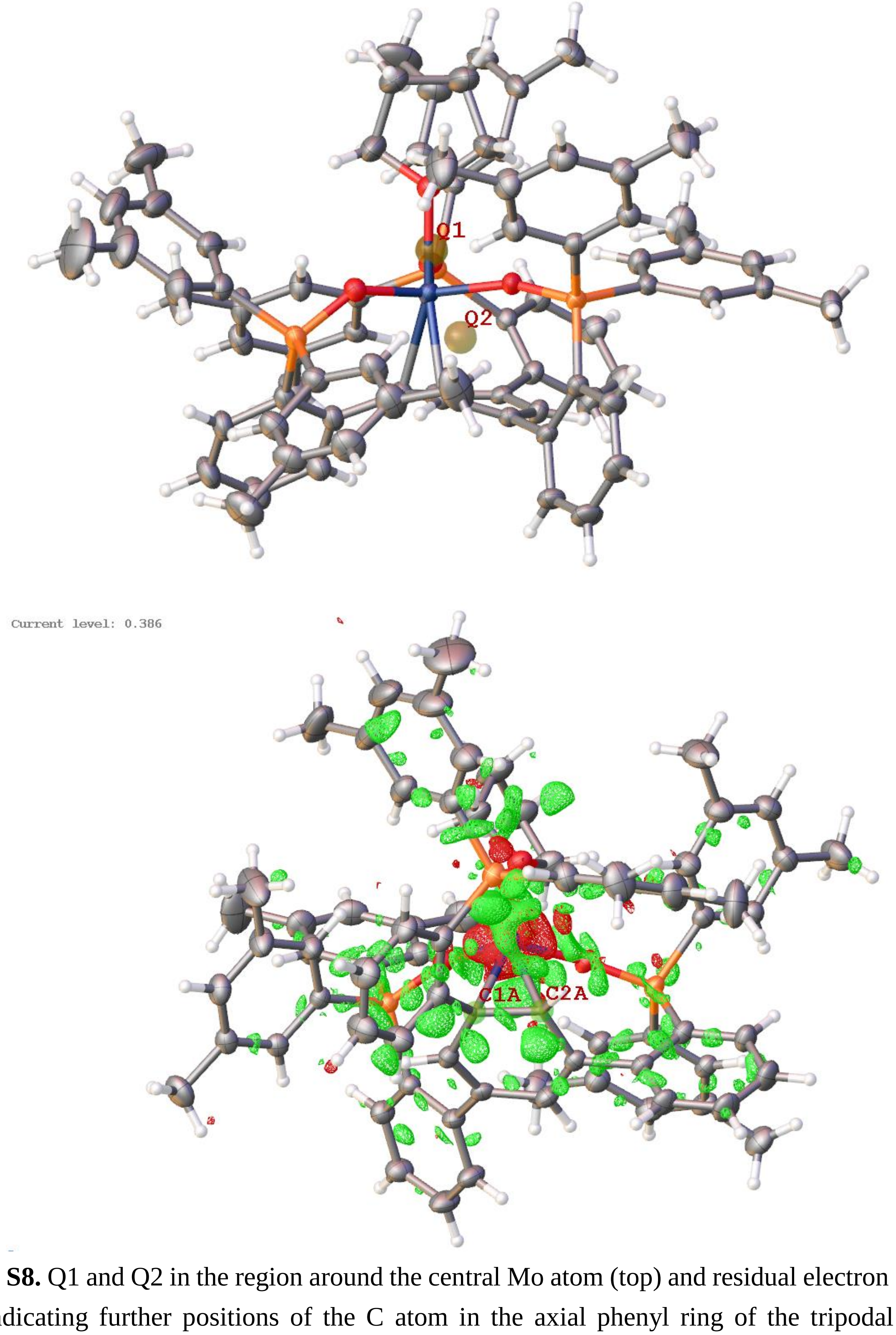

Figure S8. Q1 and Q2 in the region around the central Mo atom (top) and residual electron density map indicating further positions of the C atom in the axial phenyl ring of the tripodal ligand (bottom).

As the refinement progressed, more and more details of the other constituents in the unit cell became apparent. In particular, the position of the second Mo1B atom in combination with the other two atoms, C1B and C2B, suggested that there was a second chemical species in the crystal. It is likely an η^6-coordinate Mo1B atom comprised within the tripodal ligand. Such a complex **12** could potentially be formed by loss of the labile THF molecule from the coordination sphere of the central Mo atom of [**12**·thf].

After modelling the structure with the two different complexes [**12**·thf] and the η^6-species **12**, further residual electron density was found above the Mo1A atom. Another Mo1C atom could be refined here. Combined with observed diffuse residual density near the axial THF of PART 1 ([**12**·thf]), a molybdenum complex presumably ligated with *two* THF molecules is present in the unit cell. However, further attempts did not allow this third species to be properly refined due to medium resolution and high positional correlations. In the current model, the occupancies of all three Mo atoms are refined with a free variable combined by the SUMP instruction, resulting in an occupancy distribution of 89.976:3.017:6.994% for PART 1, 2 and 3 respectively.

In the series of crystals analyzed, somewhat different occupation ratios of the three different species were found (see the Table below). In the paper, we show the best data set with the highest free refined occupation of the *mono*-THF η^2-complex [**12**·thf].

Table S5. Overview of the experimental results of the measured crystals of compound [**12**·thf].

**Req. No.**	**T / K**	**R1 factor / %**	**Rint factor / %**	**residual electron density / e·Å-3**	**occupancy PART 1**	**occupancy PART 2**	**occupancy PART 3**	**d / $Å^{-1}$**
15571	100,00	8,65	10,48	6,00	0,85	0,05	0,05	0,73
15543	100,00	7,30	7,96	0,50	0,47	0,23	0,30	0,70
15585	100,00	4,53	11,72	1,10	0,94	0,04	0,02	0,73
15601	150,00	3,90	10,75	0,50	0,88	0,07	0,05	0,75
15592	150,00	7,33	21,05	1,90	0,80	0,10	0,10	0,75
15597	150,00	9,04	13,99	1,40	0,80	0,10	0,10	0,79
15598	100,00	8,59	11,44	1,90	0,75	0,10	0,10	0,75
15883	150,00	7,36	17,47	1,90	0,89	0,06	0,04	0,70
	100,00	11,66	23,07	5,80	1,00			0,68
15871	200,00	6,69	11,45	1,10	0,84	0,07	0,05	0,70
	253,00	7,55	15,65	1,60	0,83	0,07	0,05	0,70
15838	100,00	12,00	8,85	5,20	0,81	0,08	0,05	0,68
	150,00	4,95	7,17	0,70	0,84	0,06	0,04	0,70

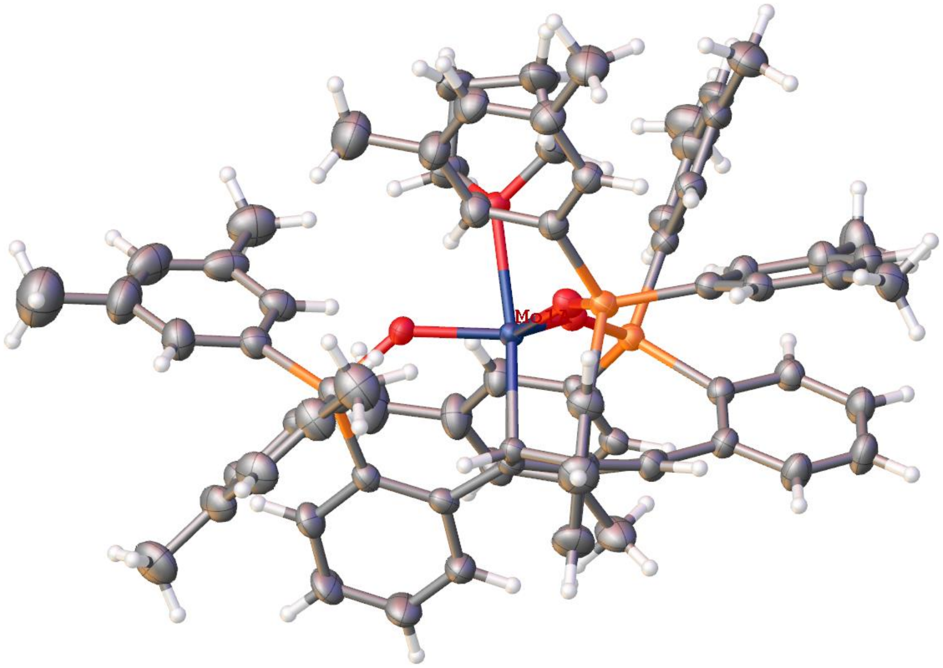

Figure S9. The main component *mono*-THF η^2-complex [**12**·thf] of the unit cell with a refined occupancy of about 90%.

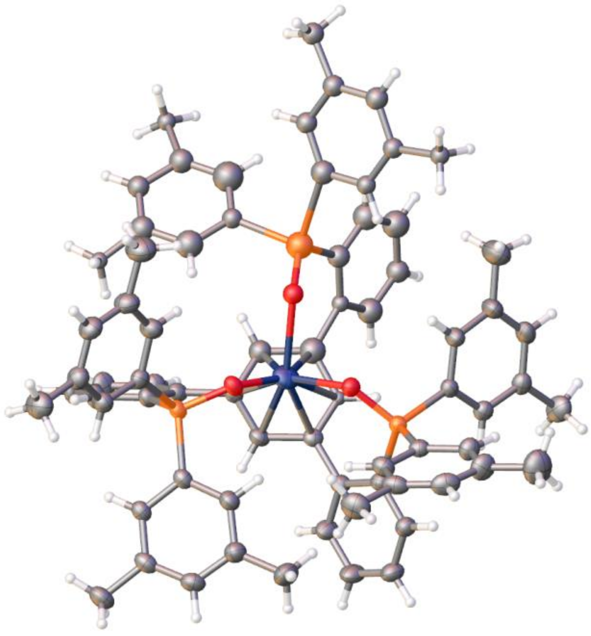

Figure S10. The first minor component of the unit cell with a refined occupancy of about 3%, *i. e.* the THF-free η^6- complex **12**.

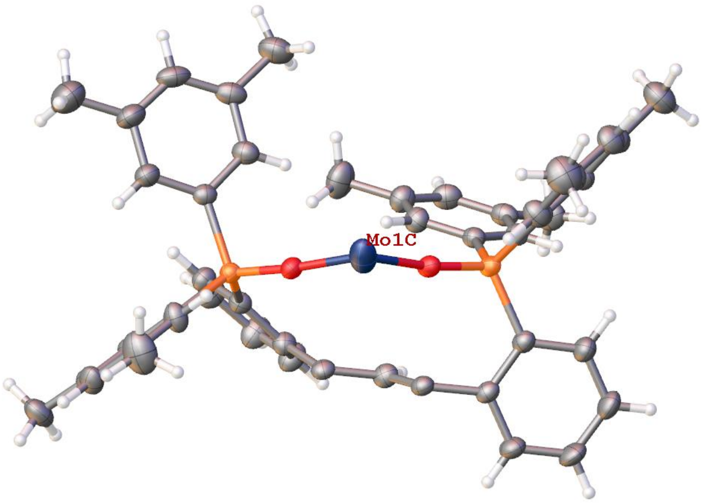

Figure S11. The second minor component of the unit cell with a occupancy of around 7%; although the structure could not be unambiguously assigned, it might be a *bis*-THF species [**12**·2thf].

To corroborate the experimental observations, geometries of the presumed constituents of the unit cell, that is the *mono*-THF η^2-complex [**12**·thf] and the THF-free η^6-complex **12**, were optimized at the B3LYP-D/def2-TZVP level of theory (D3BJ). Importantly, both calculations converged, allowing a comparison between the crystal structures and the computationally optimized structures. The calculations were carried out in the gas phase of the isolated molecule. Packing effects and potentially stabilizing inter-/intra-molecular interactions have not been taken into account.

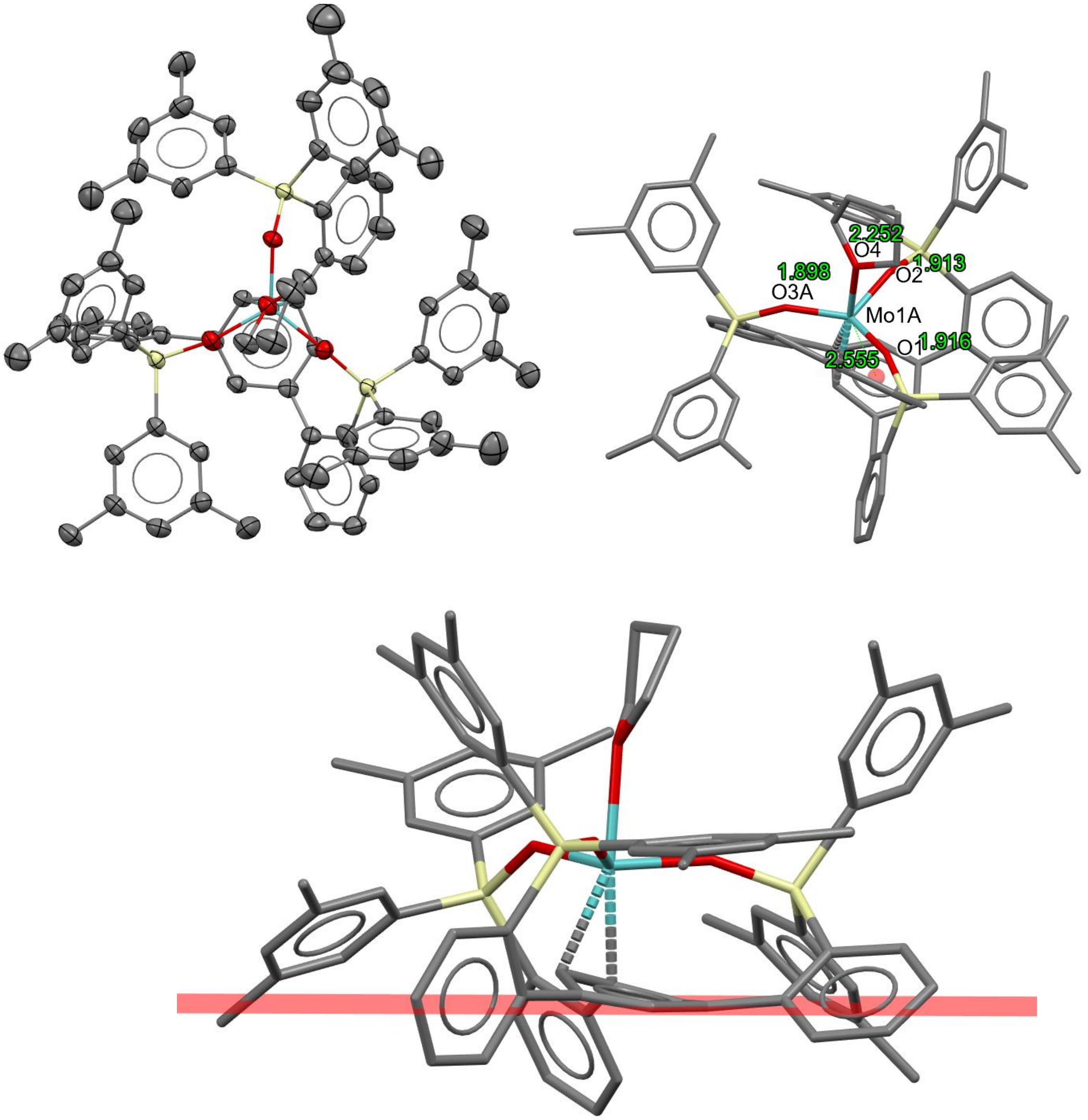

Figure S12. The *mono*-THF η^2-complex [**12**·thf] seen from above (top left) and important bond lengths as well as the Mo centroid distance (top right). The out-of-plane deflection of two C-atoms of the aryl ring (bottom).

The major component in the crystal structure is the *mono*-THF η^2-complex [**12**·thf]. This complex has been freely refined with a population of up to ~90%. Some unusual structural features can be seen. The flexibility of the tripodal ligand allows the Mo atom to be displaced from the central axis of the lower aryl ring, which results in a slight asymmetry. The Mo atom adopts a distorted trigonal bipyramidal coordination geometry. The coordination of the lower aryl ring is made possible by lifting two C atoms out of the ring plane. In addition, the Mo1A atom moves slightly downwards so that the O bonds of the ligand are also tilted. In the upper part of the structure, a THF molecule coordinates the central Mo1A atom.

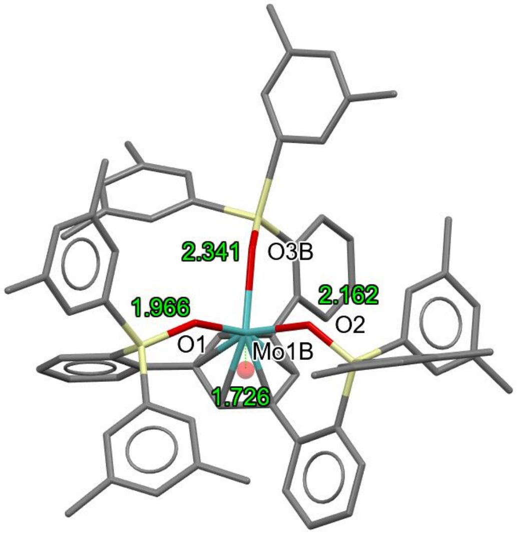

Figure S13. Distances (right) and C-Si-O-Mo torsion angles (right) of the THF-free η^6 complex **12**.

The second constituent of the unit cell appears to be a η^6-bound complex, which was also freely refined with 3% occupancy. Due to the loss of the THF ligand, the Mo atom is stabilised by the π-electron system of the basal phenyl ring of the tripodal ligand. The Mo atom is located on the central threefold axis of the molecule. The coordination results in an almost planar structure of the lower aryl ring. The Mo atom is also pulled far down ($d_{\text{aryl centroid/Mo1B}}$ = 1.726 Å) and is now below the plane of the silyloxy ligands. The Mo–O distances are longer than those of to *mono*-THF η^2-complex [**12**·thf].

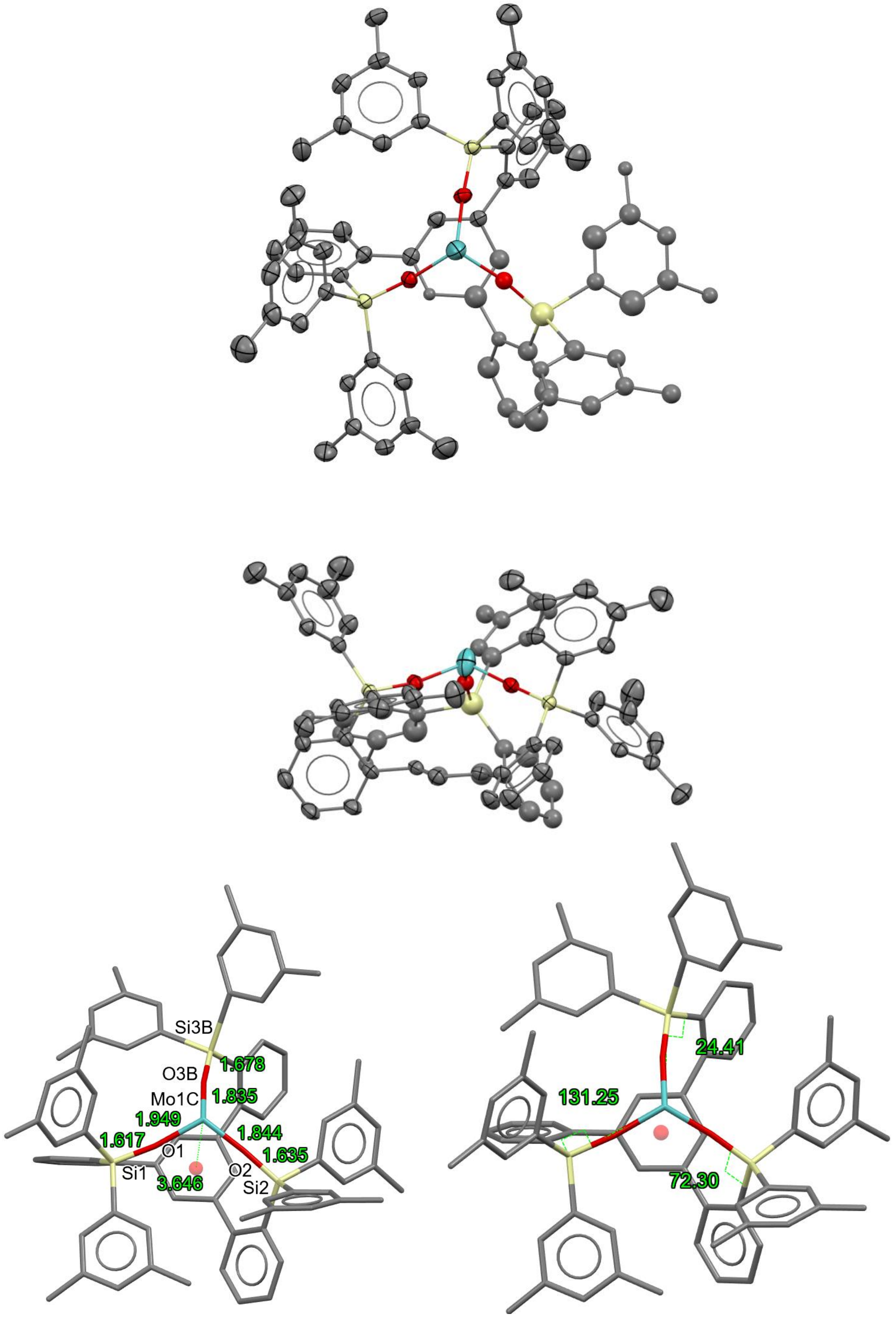

Figure S14. The geometry of the third species present in the unit cell from different angles (top); selected distances (bottom left) and C-Si-O-Mo torsion angles (bottom right).

The third complex present in the unit cell cannot be clearly identified, even though it could be a *bis*-THF complex [**12**·2thf]. Especially in the region of the THF molecule of the *mono*-THF η^2-complex [**12**·thf] there is still a low residual electron density after refinement, but the modelling of the two THF molecules was not successful due to high correlations. The geometry of this complex is similar to that of the *tris*-THF complex [**12**·3thf], as the central Mo is raised above the plane of the silyloxy groups. Based on the geometrical considerations that the upper pocket of the complex in principle offers enough space for up to three THF molecules, the formation of a *bis*-THF complex is conceivable and chemically plausible. It should be noted that theoretical calculations of the *bis*-THF complex converged.

Comparison between the Experimental Structures and the Computational Results

To confirm the proposed structures, the *mono*-THF η^2-complex [**12**·thf] and the THF-free η^6-complex **12** were computed at the B3LYP-D/def2-TZVP level of theory (D3BJ) level of theory. The atomic coordinates of the crystal structures were used as starting geometries. The structures were calculated as doublet and quartet spin states (for the Cartesian Coordinates, see the file: Supporting Information: Computational Studies). In all cases, the calculations converged and no negative frequencies were found.

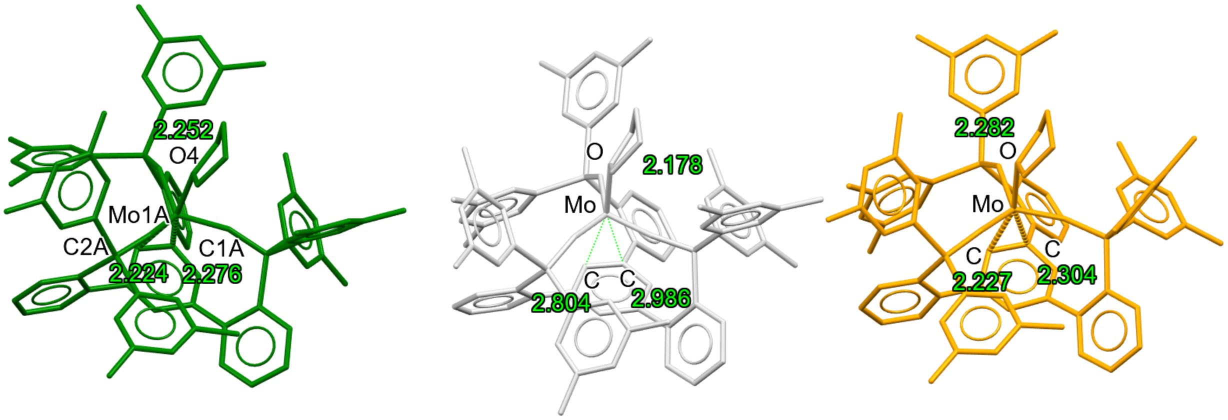

Figure S15. The experimental structure of the *mono*-THF η^{2}complex [**12**·thf] (green), the computed quartet (grey) and the doublet (orange) structures with selected distances.

Comparison of the experimental geometry of the *mono*-THF complex [**12**·thf] with the computed structures shows it to be likely a doublet species. The Mo···C distances (2.227 and 2.304 Å) and the Mo···O distance (2.282 Å) of the theoretical doublet structure are consistent with the experimental data. Moreover, the overlay ('structure overlay' option in the Mercury 2023.2.0 programme) also showed a better agreement: thus, the RMS of the doublet/experiment overlay (RMS = 0.112) indicates a better overall fit compared to the quartet/experiment overlay (RMS = 0.307).

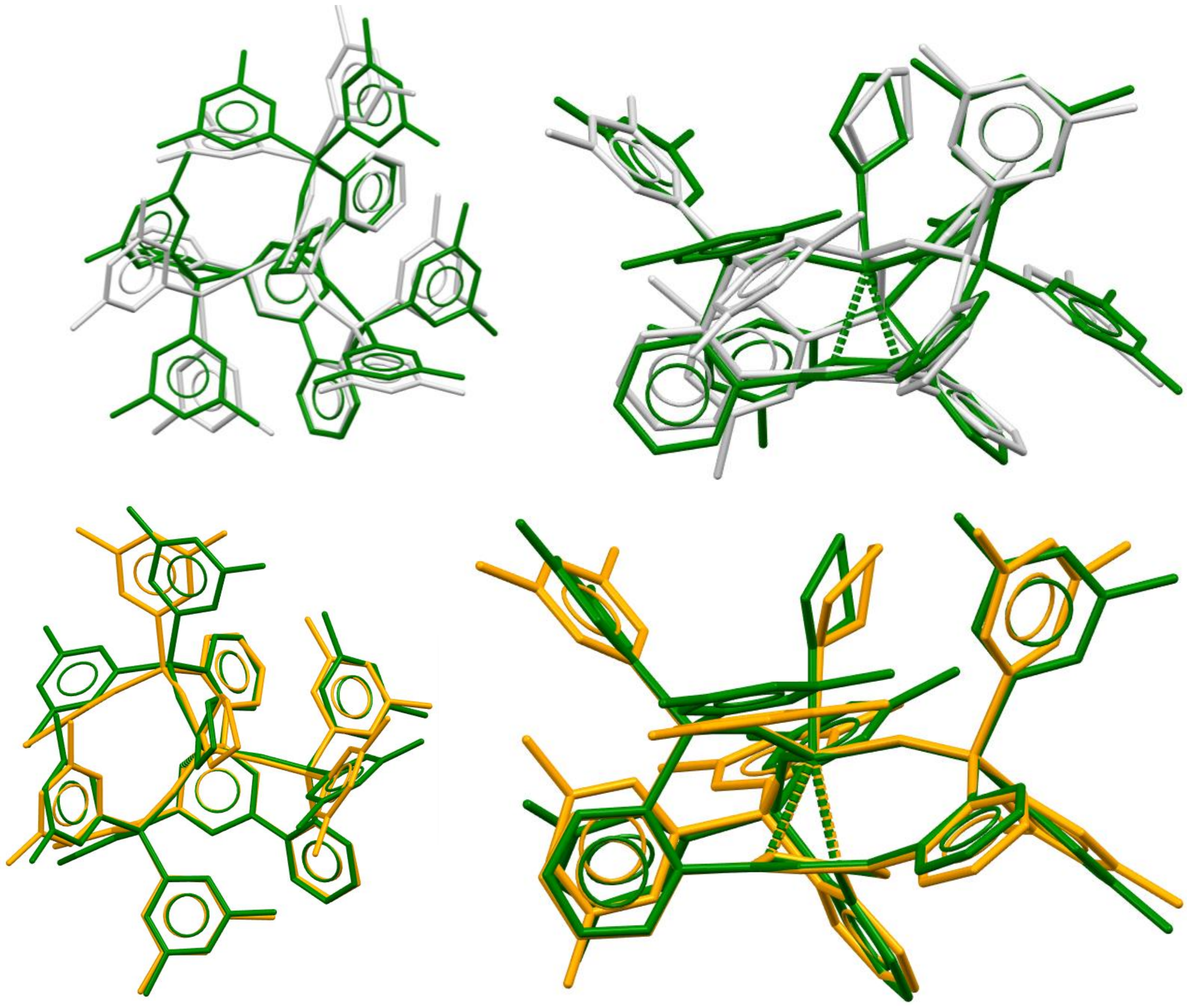

Figure S16. Superposition of the experimental structure of [**12**·thf] (green) with the calculated quartet (grey) and doublet (orange) structures in different orientations.

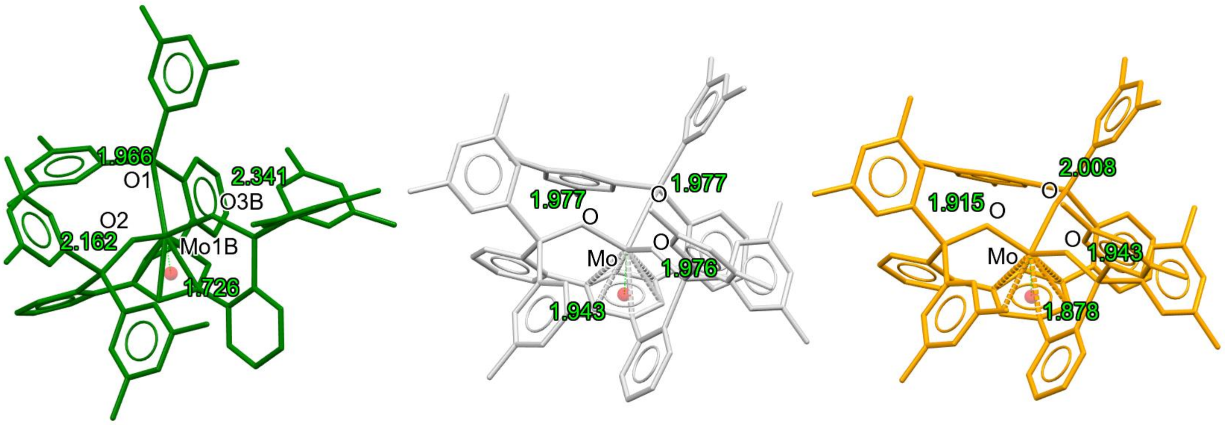

Figure S17. The experimental structure of the THF-free η^6-complex **12** (green), the theoretically calculated quartet (grey) and the doublet (orange) structures with selected distances.

A similar comparison between theory and experiment was made for the THF-free η^6-complex **12**. In this case, the calculated quartet structure describes the experimental data somewhat better. The distance between the central Mo atom and the centroid of the basal phenyl ring is closer to that in the crystal structure. The superpositioning shows that the experimental structure is described slightly better by the theoretical quartet structure (RMS = 0.179) than by the corresponding doublet structure (RMS = 0.243).

In this context, however, it should be noted that the accuracy of the crystal structure must not be overestimated. It accounts only for 3% occupation of the unit cell and the atomic positions cannot be determined very accurately due to the close proximity to the other PARTs. Packing effects and molecular interactions also play a role in the solid state but have been disregarded in the computations.

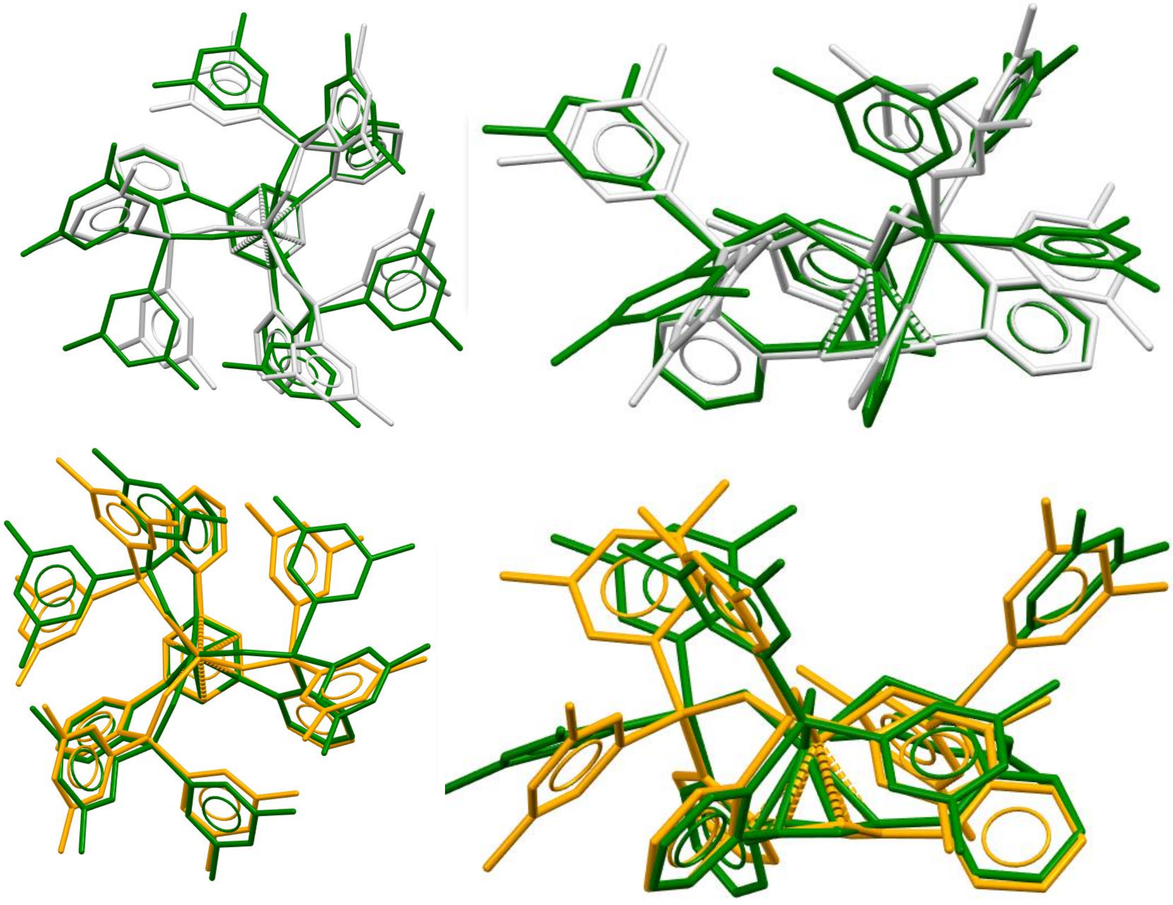

Figure S18. Superposition of the experimental structure of the THF-free η^6-complex **12** (green) with the calculated quartet (grey) and doublet (orange) structures in different orientations.

Single Crystal Structure Analysis of the Homodimeric Complex 13

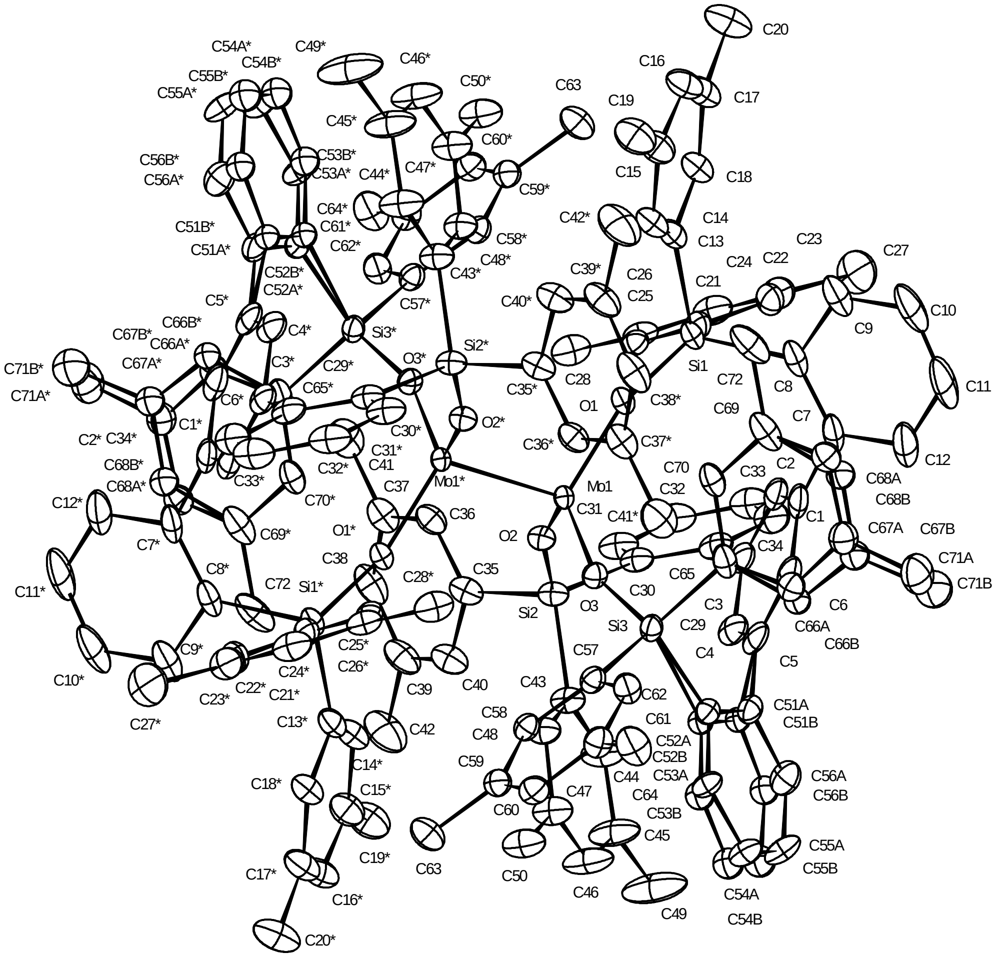

Figure S19. The molecular structure of complex **13**; H atoms have been removed for clarity.

X-ray Crystal Structure Analysis of Complex 13: $C_{89.50}$ H_{89} Mo O_3 Si_3, M_r = 1392.81 g mol^{-1}, orange block, crystal size 0.192 x 0.128 x 0.127 mm^3, Triclinic, space group *P*-1 [2], a = 16.0426(8) Å, b = 16.2382(7) Å, c = 16.4587(8) Å, α = 82.583(2)°, β = 61.746(2)°, γ = 76.552(2)°, V = 3672.3(3) Å^3, T = 296(2) K, Z = 2, D_{calc} = 1.260 g·cm^3, λ = 0.71073 Å, $\mu(Mo\text{-}K_\alpha)$ = 0.278 mm^{-1}, Numerical absorption correction ($T_{\min}$ = 0.9107, $T_{\max}$ = 1.0000), 1.290 < θ < 30.508°, Bruker-AXS Kappa Mach3 with APEX-II detector and IµS microfocus Mo-anode X-ray source, 113885 measured reflections, 22393 independent reflections, 16613 reflections with $I > 2\sigma(I)$, R_{int} = 0.0613. The structure was solved by *SHELXT* and refined by full-matrix least-squares (*SHELXL*) against F^2 to R_1 = 0.0444 [$I > 2\sigma(I)$], wR_2 = 0.1171 [all data], 766 parameters and 60 restraints. Full .cif data for the compound are available under CCDC-**2418344**.

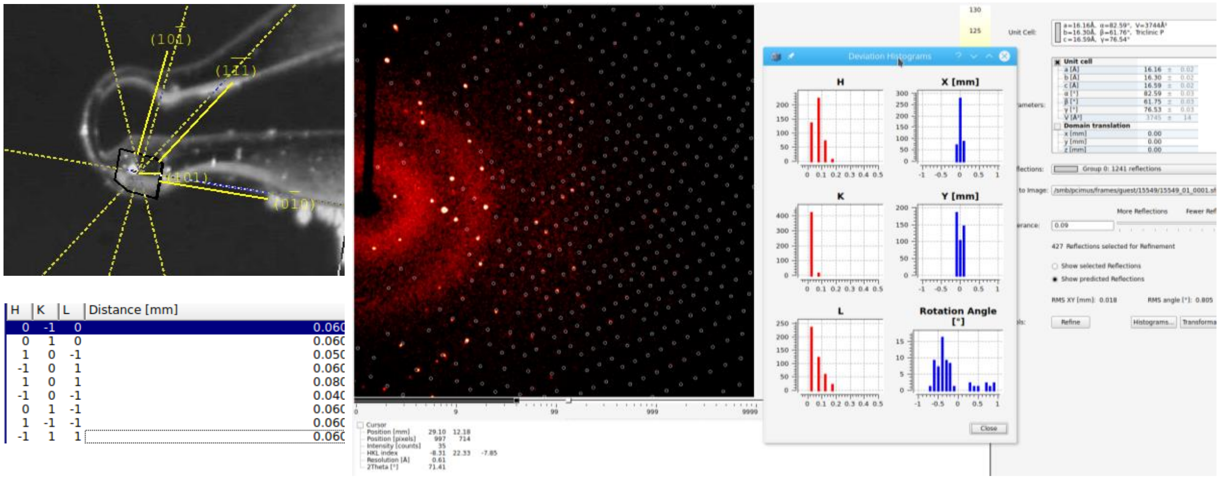

Figure S20. Crystal faces and unit cell determination/refinement of complex **13**

INTENSITY STATISTICS FOR DATASET

Resolution	#Data	#Theory	%Complete	Redundancy	Mean I	Mean I/s	Rmerge	Rsigma
Inf - 2.74	364	364	100.0	8.89	114.83	76.12	0.0225	0.0106
2.74 - 1.85	846	846	100.0	8.87	44.55	52.15	0.0291	0.0142
1.85 - 1.47	1204	1204	100.0	8.82	33.58	42.48	0.0349	0.0172
1.47 - 1.28	1254	1254	100.0	8.64	23.78	33.19	0.0444	0.0217
1.28 - 1.16	1240	1240	100.0	8.36	18.15	26.21	0.0568	0.0278
1.16 - 1.08	1197	1197	100.0	7.54	16.06	21.97	0.0681	0.0339
1.08 - 1.02	1131	1131	100.0	5.88	14.36	16.83	0.0759	0.0452
1.02 - 0.97	1193	1193	100.0	5.10	11.61	13.56	0.0933	0.0586
0.97 - 0.92	1452	1452	100.0	4.52	9.94	11.15	0.1060	0.0720
0.92 - 0.89	1037	1037	100.0	4.05	9.32	9.97	0.1174	0.0823
0.89 - 0.86	1170	1171	99.9	3.87	8.28	8.67	0.1244	0.0937
0.86 - 0.83	1361	1361	100.0	3.70	6.96	7.23	0.1466	0.1133
0.83 - 0.81	1010	1013	99.7	3.55	6.15	6.12	0.1650	0.1325
0.81 - 0.78	1761	1764	99.8	3.47	6.28	6.01	0.1768	0.1388
0.78 - 0.76	1291	1292	99.9	3.35	5.54	5.21	0.2024	0.1636
0.76 - 0.75	720	721	99.9	3.20	5.16	4.70	0.2217	0.1836
0.75 - 0.73	1528	1535	99.5	3.21	4.18	3.90	0.2458	0.2229
0.73 - 0.72	833	838	99.4	3.03	3.82	3.49	0.2621	0.2543
0.72 - 0.70	1805	1817	99.3	3.02	3.55	3.22	0.2919	0.2808
0.70 - 0.69	981	985	99.6	2.92	3.38	3.02	0.2915	0.3053
0.69 - 0.68	674	835	80.7	2.05	3.18	2.52	0.3122	0.3729
0.78 - 0.68	7832	8023	97.6	3.01	4.12	3.76	0.2517	0.2382
Inf - 0.68	24052	24250	99.2	4.89	13.07	14.61	0.0616	0.0605

A number of reflections showed a high I/σI and have been removed from the data set prior to the final cycles of refinement. A SHEL card was applied to exclude poorly determined intensities at higher diffraction angles.

In addition, a solvent mask (SQUEEZE) was applied to eliminate the residual electron density caused by unrefinable toluene molecules. Solvent masking was performed by using the BYPASS implementation of Olex2.[3-4] This results in a solvent-accessible void of 1064.38 Å³ (29.0% of the unit cell volume) at a probe radius of 1.2 Å and an approximate lattice spacing of 0.3 Å.

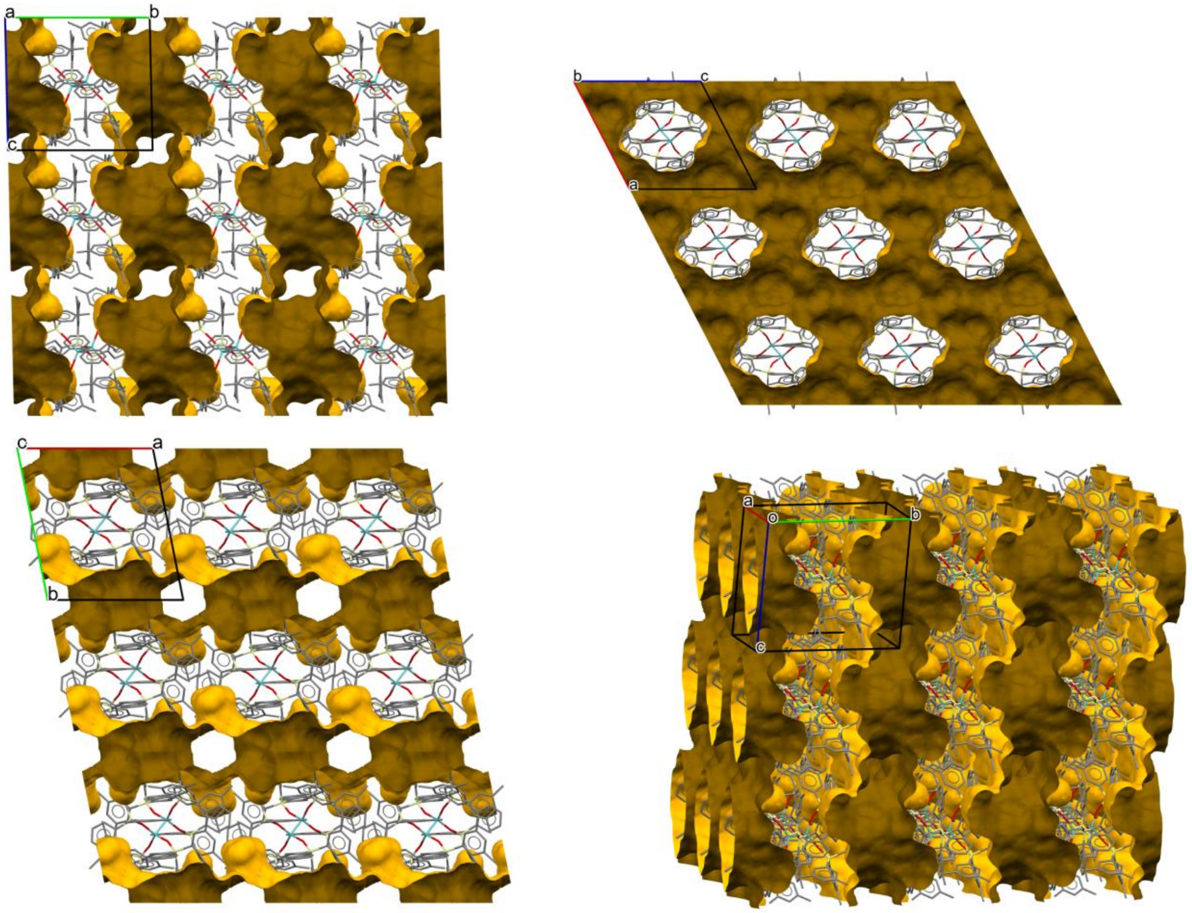

Figure S21. Solvent accessible voids in the crystal structure of complex **13** along the crystallographic a-axis (top left), b-axis (top right), c-axis (bottom left) and in a random orientation (bottom right).

Table S6. Crystal data and structure refinement of complex **13**

Identification code	15549	
Empirical formula	$C_{89.50} H_{89} Mo O_3 Si_3$	
Color	orange	
Formula weight	1392.81 g·mol^{-1}	
Temperature	296.15 K	
Wavelength	0.71073 Å	
Crystal system	Triclinic	
Space group	*P*-1, (no. 2)	
Unit cell dimensions	a = 16.0426(8) Å	α = 82.583(2)°
	b = 16.2382(7) Å	β = 61.746(2)°
	c = 16.4587(8) Å	γ = 76.552(2)°
Volume	3672.3(3) Å^3	
Z	2	
Density (calculated)	1.260 Mg·m^{-3}	
Absorption coefficient	0.278 mm^{-1}	
F(000)	1468 e	
Crystal size	0.192 x 0.128 x 0.127 mm^3	
θ range for data collection	1.290 to 30.508°.	
Index ranges	$-22 \le h \le 22$, $-23 \le k \le 23$, $-23 \le l \le 23$	
Reflections collected	113885	
Independent reflections	22393 [R_{int} = 0.0613]	
Reflections with I>2σ(I)	16613	
Completeness to θ = 25.242°	100.0 %	
Absorption correction	None	
Max. and min. transmission	1.0000 and 0.9107	
Refinement method	Full-matrix least-squares on F^2	
Data / restraints / parameters	22393 / 60 / 766	
Goodness-of-fit on F^2	1.068	
Final R indices [I>2σ(I)]	R_1 = 0.0444	wR^2 = 0.1073
R indices (all data)	R_1 = 0.0669	wR^2 = 0.1171
Extinction coefficient	n/a	
Largest diff. peak and hole	0.888 and -1.302 e·Å^{-3}	

Table S7. Bond lengths [Å] and angles [°] of complex **13**

Mo(1)-Mo(1)#1	2.2873(3)	Mo(1)-O(1)	1.9107(12)
Mo(1)-O(2)	1.9043(12)	Mo(1)-O(3)	1.9148(12)
Si(1)-O(1)	1.6525(13)	Si(1)-C(8)	1.910(2)
Si(1)-C(13)	1.879(2)	Si(1)-C(21)	1.8752(19)
Si(2)-O(2)	1.6470(13)	Si(2)-C(30)	1.905(2)
Si(2)-C(35)	1.874(2)	Si(2)-C(43)	1.874(2)
Si(3)-O(3)	1.6489(13)	Si(3)-C(57)	1.875(2)
Si(3)-C(65)	1.871(2)	Si(3)-C(52A)	1.838(8)
Si(3)-C(52B)	2.001(10)	C(1)-C(2)	1.387(3)
C(1)-C(6)	1.390(3)	C(1)-C(7)	1.479(3)
C(2)-H(2)	0.9500	C(2)-C(3)	1.394(3)
C(3)-C(4)	1.383(3)	C(3)-C(29)	1.483(3)
C(4)-H(4)	0.9500	C(4)-C(5)	1.395(3)
C(5)-C(6)	1.376(3)	C(5)-C(51A)	1.459(6)
C(5)-C(51B)	1.521(8)	C(6)-H(6)	0.9500
C(7)-C(8)	1.406(3)	C(7)-C(12)	1.392(3)
C(8)-C(9)	1.410(3)	C(9)-H(9)	0.9500
C(9)-C(10)	1.389(3)	C(10)-H(10)	0.9500
C(10)-C(11)	1.371(4)	C(11)-H(11)	0.9500
C(11)-C(12)	1.371(4)	C(12)-H(12)	0.9500
C(13)-C(14)	1.395(3)	C(13)-C(18)	1.403(3)
C(14)-H(14)	0.9500	C(14)-C(15)	1.405(3)
C(15)-C(16)	1.387(3)	C(15)-C(19)	1.506(3)
C(16)-H(16)	0.9500	C(16)-C(17)	1.383(3)
C(17)-C(18)	1.398(3)	C(17)-C(20)	1.506(3)
C(18)-H(18)	0.9500	C(19)-H(19A)	0.9800
C(19)-H(19B)	0.9800	C(19)-H(19C)	0.9800
C(20)-H(20A)	0.9800	C(20)-H(20B)	0.9800
C(20)-H(20C)	0.9800	C(21)-C(22)	1.411(3)
C(21)-C(26)	1.389(3)	C(22)-H(22)	0.9500
C(22)-C(23)	1.382(3)	C(23)-C(24)	1.388(3)
C(23)-C(27)	1.510(3)	C(24)-H(24)	0.9500
C(24)-C(25)	1.383(3)	C(25)-C(26)	1.395(3)
C(25)-C(28)	1.495(3)	C(26)-H(26)	0.9500
C(27)-H(27A)	0.9800	C(27)-H(27B)	0.9800
C(27)-H(27C)	0.9800	C(28)-H(28A)	0.9800
C(28)-H(28B)	0.9800	C(28)-H(28C)	0.9800
C(29)-C(30)	1.407(3)	C(29)-C(34)	1.393(3)
C(30)-C(31)	1.403(3)	C(31)-H(31)	0.9500
C(31)-C(32)	1.385(3)	C(32)-H(32)	0.9500
C(32)-C(33)	1.366(4)	C(33)-H(33)	0.9500
C(33)-C(34)	1.381(3)	C(34)-H(34)	0.9500
C(35)-C(36)	1.395(3)	C(35)-C(40)	1.405(3)
C(36)-H(36)	0.9500	C(36)-C(37)	1.398(3)

C(37)-C(38)	1.388(3)	C(37)-C(41)	1.506(3)
C(38)-H(38)	0.9500	C(38)-C(39)	1.379(3)
C(39)-C(40)	1.387(3)	C(39)-C(42)	1.507(3)
C(40)-H(40)	0.9500	C(41)-H(41A)	0.9800
C(41)-H(41B)	0.9800	C(41)-H(41C)	0.9800
C(42)-H(42A)	0.9800	C(42)-H(42B)	0.9800
C(42)-H(42C)	0.9800	C(43)-C(44)	1.405(3)
C(43)-C(48)	1.393(3)	C(44)-H(44)	0.9500
C(44)-C(45)	1.380(3)	C(45)-C(46)	1.391(3)
C(45)-C(49)	1.515(3)	C(46)-H(46)	0.9500
C(46)-C(47)	1.394(3)	C(47)-C(48)	1.390(3)
C(47)-C(50)	1.507(3)	C(48)-H(48)	0.9500
C(49)-H(49A)	0.9800	C(49)-H(49B)	0.9800
C(49)-H(49C)	0.9800	C(50)-H(50A)	0.9800
C(50)-H(50B)	0.9800	C(50)-H(50C)	0.9800
C(57)-C(58)	1.400(3)	C(57)-C(62)	1.402(3)
C(58)-H(58)	0.9500	C(58)-C(59)	1.388(3)
C(59)-C(60)	1.394(3)	C(59)-C(63)	1.503(3)
C(60)-H(60)	0.9500	C(60)-C(61)	1.392(3)
C(61)-C(62)	1.394(3)	C(61)-C(64)	1.507(3)
C(62)-H(62)	0.9500	C(63)-H(63A)	0.9800
C(63)-H(63B)	0.9800	C(63)-H(63C)	0.9800
C(64)-H(64A)	0.9800	C(64)-H(64B)	0.9800
C(64)-H(64C)	0.9800	C(65)-C(70)	1.390(3)
C(65)-C(66A)	1.385(5)	C(65)-C(66B)	1.449(6)
C(69)-C(70)	1.397(3)	C(69)-C(72)	1.501(3)
C(69)-C(68A)	1.286(5)	C(69)-C(68B)	1.509(6)
C(70)-H(70)	0.9500	C(72)-H(72A)	0.9800
C(72)-H(72B)	0.9800	C(72)-H(72C)	0.9800
C(51A)-C(52A)	1.414(10)	C(51A)-C(56A)	1.395(7)
C(52A)-C(53A)	1.410(10)	C(53A)-H(53A)	0.9500
C(53A)-C(54A)	1.406(8)	C(54A)-H(54A)	0.9500
C(54A)-C(55A)	1.388(7)	C(55A)-H(55A)	0.9500
C(55A)-C(56A)	1.394(7)	C(56A)-H(56A)	0.9500
C(66A)-H(66A)	0.9500	C(66A)-C(67A)	1.391(7)
C(67A)-C(68A)	1.402(7)	C(67A)-C(71A)	1.529(6)
C(68A)-H(68A)	0.9500	C(71A)-H(71A)	0.9800
C(71A)-H(71B)	0.9800	C(71A)-H(71C)	0.9800
C(51B)-C(52B)	1.413(13)	C(51B)-C(56B)	1.395(9)
C(52B)-C(53B)	1.391(14)	C(53B)-H(53B)	0.9500
C(53B)-C(54B)	1.365(11)	C(54B)-H(54B)	0.9500
C(54B)-C(55B)	1.367(9)	C(55B)-H(55B)	0.9500
C(55B)-C(56B)	1.362(8)	C(56B)-H(56B)	0.9500
C(66B)-H(66B)	0.9500	C(66B)-C(67B)	1.390(7)
C(67B)-C(68B)	1.386(7)	C(67B)-C(71B)	1.511(8)
C(68B)-H(68B)	0.9500	C(71B)-H(71D)	0.9800
C(71B)-H(71E)	0.9800	C(71B)-H(71F)	0.9800

O(1)-Mo(1)-Mo(1)#1	103.23(4)	O(1)-Mo(1)-O(3)	115.70(5)
O(2)-Mo(1)-Mo(1)#1	102.06(4)	O(2)-Mo(1)-O(1)	115.00(5)
O(2)-Mo(1)-O(3)	115.00(5)	O(3)-Mo(1)-Mo(1)#1	103.11(4)
O(1)-Si(1)-C(8)	114.23(8)	O(1)-Si(1)-C(13)	112.60(8)
O(1)-Si(1)-C(21)	110.29(8)	C(13)-Si(1)-C(8)	101.67(9)
C(21)-Si(1)-C(8)	109.59(9)	C(21)-Si(1)-C(13)	108.01(9)
O(2)-Si(2)-C(30)	114.18(8)	O(2)-Si(2)-C(35)	113.09(8)
O(2)-Si(2)-C(43)	109.65(8)	C(35)-Si(2)-C(30)	101.24(9)
C(35)-Si(2)-C(43)	107.37(9)	C(43)-Si(2)-C(30)	110.90(9)
O(3)-Si(3)-C(57)	112.65(8)	O(3)-Si(3)-C(65)	109.72(8)
O(3)-Si(3)-C(52A)	117.8(2)	O(3)-Si(3)-C(52B)	112.3(3)
C(57)-Si(3)-C(52B)	100.1(3)	C(65)-Si(3)-C(57)	107.71(9)
C(65)-Si(3)-C(52B)	114.1(3)	C(52A)-Si(3)-C(57)	101.1(3)
C(52A)-Si(3)-C(65)	107.2(3)	Si(1)-O(1)-Mo(1)	158.67(8)
Si(2)-O(2)-Mo(1)	157.37(8)	Si(3)-O(3)-Mo(1)	160.09(8)
C(2)-C(1)-C(6)	118.7(2)	C(2)-C(1)-C(7)	120.41(19)
C(6)-C(1)-C(7)	120.87(19)	C(1)-C(2)-H(2)	119.5
C(1)-C(2)-C(3)	120.92(19)	C(3)-C(2)-H(2)	119.5
C(2)-C(3)-C(29)	118.72(18)	C(4)-C(3)-C(2)	119.2(2)
C(4)-C(3)-C(29)	122.1(2)	C(3)-C(4)-H(4)	119.7
C(3)-C(4)-C(5)	120.5(2)	C(5)-C(4)-H(4)	119.7
C(4)-C(5)-C(51A)	127.2(3)	C(4)-C(5)-C(51B)	113.6(3)
C(6)-C(5)-C(4)	119.4(2)	C(6)-C(5)-C(51A)	113.4(3)
C(6)-C(5)-C(51B)	127.1(3)	C(1)-C(6)-H(6)	119.4
C(5)-C(6)-C(1)	121.3(2)	C(5)-C(6)-H(6)	119.4
C(8)-C(7)-C(1)	121.37(17)	C(12)-C(7)-C(1)	117.8(2)
C(12)-C(7)-C(8)	120.8(2)	C(7)-C(8)-Si(1)	125.95(15)
C(7)-C(8)-C(9)	116.38(19)	C(9)-C(8)-Si(1)	117.66(17)
C(8)-C(9)-H(9)	119.2	C(10)-C(9)-C(8)	121.6(2)
C(10)-C(9)-H(9)	119.2	C(9)-C(10)-H(10)	119.8
C(11)-C(10)-C(9)	120.4(2)	C(11)-C(10)-H(10)	119.8
C(10)-C(11)-H(11)	120.3	C(10)-C(11)-C(12)	119.5(2)
C(12)-C(11)-H(11)	120.3	C(7)-C(12)-H(12)	119.5
C(11)-C(12)-C(7)	121.1(2)	C(11)-C(12)-H(12)	119.5
C(14)-C(13)-Si(1)	122.58(15)	C(14)-C(13)-C(18)	117.82(18)
C(18)-C(13)-Si(1)	117.91(16)	C(13)-C(14)-H(14)	119.3

C(13)-C(14)-C(15)	121.47(19)	C(15)-C(14)-H(14)	119.3
C(14)-C(15)-C(19)	120.6(2)	C(16)-C(15)-C(14)	118.3(2)
C(16)-C(15)-C(19)	121.09(19)	C(15)-C(16)-H(16)	118.9
C(17)-C(16)-C(15)	122.3(2)	C(17)-C(16)-H(16)	118.9
C(16)-C(17)-C(18)	118.1(2)	C(16)-C(17)-C(20)	121.2(2)
C(18)-C(17)-C(20)	120.7(2)	C(13)-C(18)-H(18)	119.0
C(17)-C(18)-C(13)	121.9(2)	C(17)-C(18)-H(18)	119.0
C(15)-C(19)-H(19A)	109.5	C(15)-C(19)-H(19B)	109.5
C(15)-C(19)-H(19C)	109.5	H(19A)-C(19)-H(19B)	109.5
H(19A)-C(19)-H(19C)	109.5	H(19B)-C(19)-H(19C)	109.5
C(17)-C(20)-H(20A)	109.5	C(17)-C(20)-H(20B)	109.5
C(17)-C(20)-H(20C)	109.5	H(20A)-C(20)-H(20B)	109.5
H(20A)-C(20)-H(20C)	109.5	H(20B)-C(20)-H(20C)	109.5
C(22)-C(21)-Si(1)	119.50(15)	C(26)-C(21)-Si(1)	122.95(15)
C(26)-C(21)-C(22)	117.54(18)	C(21)-C(22)-H(22)	119.2
C(23)-C(22)-C(21)	121.7(2)	C(23)-C(22)-H(22)	119.2
C(22)-C(23)-C(24)	118.4(2)	C(22)-C(23)-C(27)	121.3(2)
C(24)-C(23)-C(27)	120.2(2)	C(23)-C(24)-H(24)	118.9
C(25)-C(24)-C(23)	122.2(2)	C(25)-C(24)-H(24)	118.9
C(24)-C(25)-C(26)	118.08(19)	C(24)-C(25)-C(28)	120.46(19)
C(26)-C(25)-C(28)	121.46(19)	C(21)-C(26)-C(25)	122.05(18)
C(21)-C(26)-H(26)	119.0	C(25)-C(26)-H(26)	119.0
C(23)-C(27)-H(27A)	109.5	C(23)-C(27)-H(27B)	109.5
C(23)-C(27)-H(27C)	109.5	H(27A)-C(27)-H(27B)	109.5
H(27A)-C(27)-H(27C)	109.5	H(27B)-C(27)-H(27C)	109.5
C(25)-C(28)-H(28A)	109.5	C(25)-C(28)-H(28B)	109.5
C(25)-C(28)-H(28C)	109.5	H(28A)-C(28)-H(28B)	109.5
H(28A)-C(28)-H(28C)	109.5	H(28B)-C(28)-H(28C)	109.5
C(30)-C(29)-C(3)	121.19(18)	C(34)-C(29)-C(3)	118.1(2)
C(34)-C(29)-C(30)	120.7(2)	C(29)-C(30)-Si(2)	125.33(15)
C(31)-C(30)-Si(2)	118.23(17)	C(31)-C(30)-C(29)	116.43(19)
C(30)-C(31)-H(31)	118.9	C(32)-C(31)-C(30)	122.3(2)
C(32)-C(31)-H(31)	118.9	C(31)-C(32)-H(32)	119.9
C(33)-C(32)-C(31)	120.1(2)	C(33)-C(32)-H(32)	119.9
C(32)-C(33)-H(33)	120.2	C(32)-C(33)-C(34)	119.6(2)
C(34)-C(33)-H(33)	120.2	C(29)-C(34)-H(34)	119.5

C(33)-C(34)-C(29)	120.9(2)	C(33)-C(34)-H(34)	119.5
C(36)-C(35)-Si(2)	122.36(14)	C(36)-C(35)-C(40)	117.61(18)
C(40)-C(35)-Si(2)	117.79(16)	C(35)-C(36)-H(36)	119.3
C(35)-C(36)-C(37)	121.42(18)	C(37)-C(36)-H(36)	119.3
C(36)-C(37)-C(41)	120.65(19)	C(38)-C(37)-C(36)	118.3(2)
C(38)-C(37)-C(41)	121.07(19)	C(37)-C(38)-H(38)	118.8
C(39)-C(38)-C(37)	122.4(2)	C(39)-C(38)-H(38)	118.8
C(38)-C(39)-C(40)	118.00(19)	C(38)-C(39)-C(42)	121.3(2)
C(40)-C(39)-C(42)	120.7(2)	C(35)-C(40)-H(40)	118.9
C(39)-C(40)-C(35)	122.2(2)	C(39)-C(40)-H(40)	118.9
C(37)-C(41)-H(41A)	109.5	C(37)-C(41)-H(41B)	109.5
C(37)-C(41)-H(41C)	109.5	H(41A)-C(41)-H(41B)	109.5
H(41A)-C(41)-H(41C)	109.5	H(41B)-C(41)-H(41C)	109.5
C(39)-C(42)-H(42A)	109.5	C(39)-C(42)-H(42B)	109.5
C(39)-C(42)-H(42C)	109.5	H(42A)-C(42)-H(42B)	109.5
H(42A)-C(42)-H(42C)	109.5	H(42B)-C(42)-H(42C)	109.5
C(44)-C(43)-Si(2)	118.82(15)	C(48)-C(43)-Si(2)	123.80(14)
C(48)-C(43)-C(44)	117.36(18)	C(43)-C(44)-H(44)	118.8
C(45)-C(44)-C(43)	122.48(19)	C(45)-C(44)-H(44)	118.8
C(44)-C(45)-C(46)	118.06(19)	C(44)-C(45)-C(49)	121.1(2)
C(46)-C(45)-C(49)	120.9(2)	C(45)-C(46)-H(46)	119.1
C(45)-C(46)-C(47)	121.8(2)	C(47)-C(46)-H(46)	119.1
C(46)-C(47)-C(50)	120.59(19)	C(48)-C(47)-C(46)	118.36(19)
C(48)-C(47)-C(50)	121.04(18)	C(43)-C(48)-H(48)	119.0
C(47)-C(48)-C(43)	121.90(18)	C(47)-C(48)-H(48)	119.0
C(45)-C(49)-H(49A)	109.5	C(45)-C(49)-H(49B)	109.5
C(45)-C(49)-H(49C)	109.5	H(49A)-C(49)-H(49B)	109.5
H(49A)-C(49)-H(49C)	109.5	H(49B)-C(49)-H(49C)	109.5
C(47)-C(50)-H(50A)	109.5	C(47)-C(50)-H(50B)	109.5
C(47)-C(50)-H(50C)	109.5	H(50A)-C(50)-H(50B)	109.5
H(50A)-C(50)-H(50C)	109.5	H(50B)-C(50)-H(50C)	109.5
C(58)-C(57)-Si(3)	122.23(14)	C(58)-C(57)-C(62)	117.38(18)
C(62)-C(57)-Si(3)	118.39(15)	C(57)-C(58)-H(58)	119.0
C(59)-C(58)-C(57)	121.93(17)	C(59)-C(58)-H(58)	119.0
C(58)-C(59)-C(60)	118.80(18)	C(58)-C(59)-C(63)	120.84(17)
C(60)-C(59)-C(63)	120.35(18)	C(59)-C(60)-H(60)	119.3

C(61)-C(60)-C(59)	121.38(18)	C(61)-C(60)-H(60)	119.3
C(60)-C(61)-C(62)	118.30(18)	C(60)-C(61)-C(64)	121.13(19)
C(62)-C(61)-C(64)	120.52(19)	C(57)-C(62)-H(62)	118.9
C(61)-C(62)-C(57)	122.12(19)	C(61)-C(62)-H(62)	118.9
C(59)-C(63)-H(63A)	109.5	C(59)-C(63)-H(63B)	109.5
C(59)-C(63)-H(63C)	109.5	H(63A)-C(63)-H(63B)	109.5
H(63A)-C(63)-H(63C)	109.5	H(63B)-C(63)-H(63C)	109.5
C(61)-C(64)-H(64A)	109.5	C(61)-C(64)-H(64B)	109.5
C(61)-C(64)-H(64C)	109.5	H(64A)-C(64)-H(64B)	109.5
H(64A)-C(64)-H(64C)	109.5	H(64B)-C(64)-H(64C)	109.5
C(70)-C(65)-Si(3)	122.25(14)	C(70)-C(65)-C(66B)	123.8(3)
C(66A)-C(65)-Si(3)	124.8(3)	C(66A)-C(65)-C(70)	112.4(3)
C(66B)-C(65)-Si(3)	113.8(3)	C(70)-C(69)-C(72)	120.8(2)
C(70)-C(69)-C(68B)	112.5(3)	C(72)-C(69)-C(68B)	126.4(3)
C(68A)-C(69)-C(70)	124.7(3)	C(68A)-C(69)-C(72)	114.1(3)
C(65)-C(70)-C(69)	121.58(19)	C(65)-C(70)-H(70)	119.2
C(69)-C(70)-H(70)	119.2	C(69)-C(72)-H(72A)	109.5
C(69)-C(72)-H(72B)	109.5	C(69)-C(72)-H(72C)	109.5
H(72A)-C(72)-H(72B)	109.5	H(72A)-C(72)-H(72C)	109.5
H(72B)-C(72)-H(72C)	109.5	C(52A)-C(51A)-C(5)	122.7(5)
C(56A)-C(51A)-C(5)	116.3(5)	C(56A)-C(51A)-C(52A)	120.9(5)
C(51A)-C(52A)-Si(3)	125.2(5)	C(53A)-C(52A)-Si(3)	118.0(5)
C(53A)-C(52A)-C(51A)	116.8(6)	C(52A)-C(53A)-H(53A)	119.0
C(54A)-C(53A)-C(52A)	122.1(6)	C(54A)-C(53A)-H(53A)	119.0
C(53A)-C(54A)-H(54A)	120.2	C(55A)-C(54A)-C(53A)	119.6(5)
C(55A)-C(54A)-H(54A)	120.2	C(54A)-C(55A)-H(55A)	120.3
C(54A)-C(55A)-C(56A)	119.4(4)	C(56A)-C(55A)-H(55A)	120.3
C(51A)-C(56A)-H(56A)	119.4	C(55A)-C(56A)-C(51A)	121.1(5)
C(55A)-C(56A)-H(56A)	119.4	C(65)-C(66A)-H(66A)	117.3
C(65)-C(66A)-C(67A)	125.4(4)	C(67A)-C(66A)-H(66A)	117.3
C(66A)-C(67A)-C(68A)	118.3(4)	C(66A)-C(67A)-C(71A)	119.8(4)
C(68A)-C(67A)-C(71A)	121.8(4)	C(69)-C(68A)-C(67A)	117.2(4)
C(69)-C(68A)-H(68A)	121.4	C(67A)-C(68A)-H(68A)	121.4
C(67A)-C(71A)-H(71A)	109.5	C(67A)-C(71A)-H(71B)	109.5
C(67A)-C(71A)-H(71C)	109.5	H(71A)-C(71A)-H(71B)	109.5
H(71A)-C(71A)-H(71C)	109.5	H(71B)-C(71A)-H(71C)	109.5

C(52B)-C(51B)-C(5)	116.9(6)	C(56B)-C(51B)-C(5)	122.9(6)
C(56B)-C(51B)-C(52B)	119.8(7)	C(51B)-C(52B)-Si(3)	124.9(7)
C(53B)-C(52B)-Si(3)	119.4(7)	C(53B)-C(52B)-C(51B)	115.8(8)
C(52B)-C(53B)-H(53B)	118.3	C(54B)-C(53B)-C(52B)	123.4(8)
C(54B)-C(53B)-H(53B)	118.3	C(53B)-C(54B)-H(54B)	120.0
C(53B)-C(54B)-C(55B)	120.0(7)	C(55B)-C(54B)-H(54B)	120.0
C(54B)-C(55B)-H(55B)	120.4	C(56B)-C(55B)-C(54B)	119.1(6)
C(56B)-C(55B)-H(55B)	120.4	C(51B)-C(56B)-H(56B)	119.1
C(55B)-C(56B)-C(51B)	121.7(6)	C(55B)-C(56B)-H(56B)	119.1
C(65)-C(66B)-H(66B)	121.3	C(67B)-C(66B)-C(65)	117.3(4)
C(67B)-C(66B)-H(66B)	121.3	C(66B)-C(67B)-C(71B)	122.6(5)
C(68B)-C(67B)-C(66B)	118.4(5)	C(68B)-C(67B)-C(71B)	118.9(5)
C(69)-C(68B)-H(68B)	117.1	C(67B)-C(68B)-C(69)	125.9(5)
C(67B)-C(68B)-H(68B)	117.1	C(67B)-C(71B)-H(71D)	109.5
C(67B)-C(71B)-H(71E)	109.5	C(67B)-C(71B)-H(71F)	109.5
H(71D)-C(71B)-H(71E)	109.5	H(71D)-C(71B)-H(71F)	109.5
H(71E)-C(71B)-H(71F)	109.5		

Symmetry transformations used to generate equivalent atoms:
#1 -x+1,-y+1,-z+1

Single Crystal Structure Analysis of Complex 14

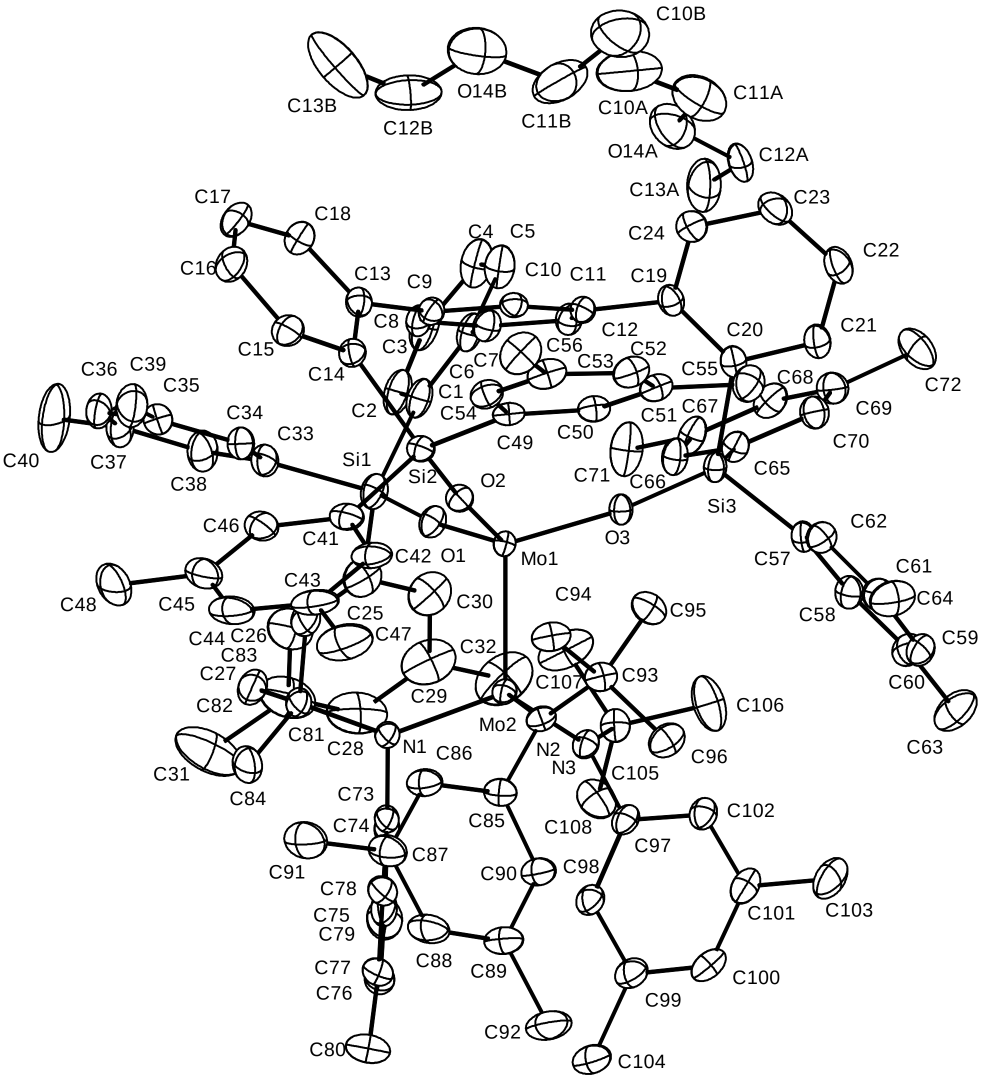

Figure S22. The molecular structure of complex **14**; H atoms removed for clarity.

X-ray Crystal Structure Analysis of 14: C_{112} H_{133} Mo_2 N_3 O_4 Si_3, M_r = 1861.36 g mol^{-1}, orange plate, crystal size 0.242 x 0.106 x 0.06 mm^3, Monoclinic, space group $P2_1/n$ [14], a = 14.5704(6) Å, b = 27.4246(13) Å, c = 25.0257(12) Å, β = 90.816(2)°, V = 9998.9(8) Å^3, T = 100(2) K, Z = 4, D_{calc} = 1.236 g·cm^3, λ = 0.71073 Å, $\mu(Mo\text{-}K_\alpha)$ = 0.340 mm^{-1}, Gaussian absorption correction ($T_{\min}$ = 0.95950, $T_{\max}$ = 0.98820), Bruker-AXS Kappa Mach3 with APEX-II detector and IµS microfocus Mo-anode X-ray source, 1.583 < θ < 30.034°, 379265 measured reflections, 29260 independent reflections, 22287 reflections with $I > 2\sigma(I)$, R_{int} = 0.0813. The structure was solved by *SHELXT* and refined by full-matrix least-squares (*SHELXL*) against F^2 to R_1 = 0.0414 [$I > 2\sigma(I)$], wR_2 = 0.1130 [all data], 1208 parameters and 0 restraints. **CCDC-2303032**.

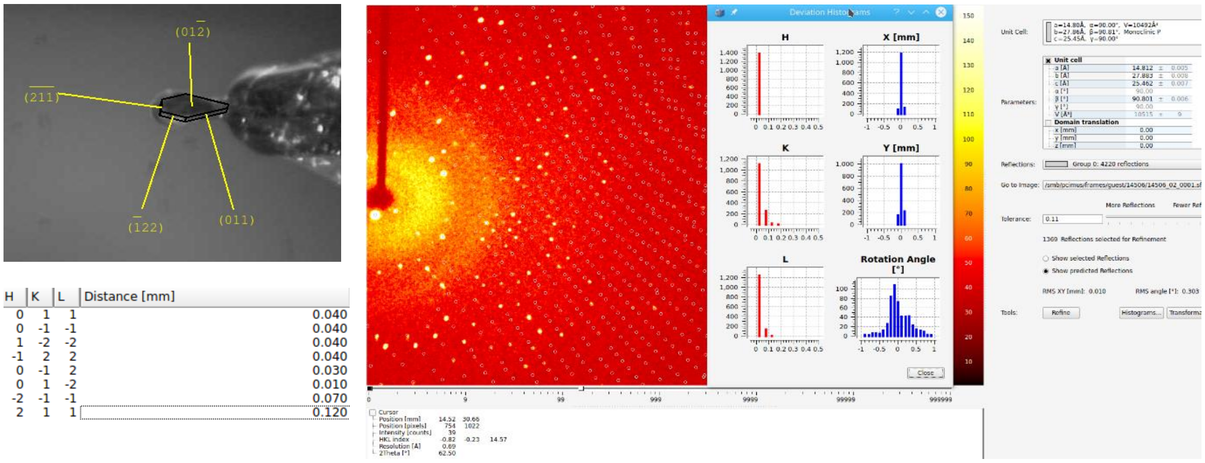

Figure S23. Crystal faces and unit cell determination/refinement of complex **14**.

INTENSITY STATISTICS FOR DATASET

Resolution	#Data	#Theory	%Complete	Redundancy	Mean I	Mean I/s	Rmerge	Rsigma
Inf - 2.83	498	500	99.6	20.40	74.70	68.00	0.0279	0.0111
2.83 - 1.88	1173	1173	100.0	21.80	32.28	58.57	0.0389	0.0121
1.88 - 1.48	1681	1681	100.0	21.83	24.07	50.48	0.0456	0.0135
1.48 - 1.29	1711	1711	100.0	21.65	15.56	41.03	0.0596	0.0168
1.29 - 1.17	1697	1697	100.0	20.69	11.91	34.60	0.0733	0.0204
1.17 - 1.09	1586	1586	100.0	18.92	12.08	30.73	0.0764	0.0226
1.09 - 1.02	1809	1809	100.0	14.89	9.18	22.24	0.0970	0.0322
1.02 - 0.97	1616	1616	100.0	12.80	7.64	18.01	0.1155	0.0412
0.97 - 0.93	1578	1578	100.0	11.34	6.87	15.39	0.1317	0.0493
0.93 - 0.89	1872	1872	100.0	10.24	6.00	12.88	0.1508	0.0593
0.89 - 0.86	1628	1628	100.0	9.64	5.46	11.31	0.1641	0.0683
0.86 - 0.83	1898	1898	100.0	9.17	5.10	10.06	0.1800	0.0771
0.83 - 0.81	1391	1391	100.0	8.99	4.38	8.75	0.2040	0.0903
0.81 - 0.79	1586	1586	100.0	8.65	4.27	8.13	0.2167	0.0971
0.79 - 0.77	1704	1704	100.0	8.48	3.56	6.97	0.2509	0.1177
0.77 - 0.75	1935	1935	100.0	8.12	3.60	6.43	0.2633	0.1239
0.75 - 0.73	2097	2097	100.0	7.93	3.07	5.57	0.3097	0.1498
0.73 - 0.72	1155	1155	100.0	7.61	3.07	5.35	0.3116	0.1569
0.72 - 0.71	1250	1250	100.0	7.56	2.60	4.59	0.3647	0.1872
0.71 - 0.69	2611	2623	99.5	6.58	2.45	3.94	0.3892	0.2292
0.69 - 0.68	566	1152	49.1	0.91	1.88	1.74	0.3421	0.5314
0.78 - 0.68	10488	11086	94.6	6.88	2.92	5.07	0.3144	0.1759
Inf - 0.68	33042	33642	98.2	11.95	9.01	18.30	0.0794	0.0445

The examined crystal shows pure/weak diffraction intensities. Therefore, a longer exposure time per frame was used. A resolution cut-off (SHEL 999 0.71) was applied to exclude poorly determined reflections at high diffraction angles. Several reflections were omitted from the data set before the final refinement cycles due to high I/σI (> 10). One ^{t}Bu group shows a rotational disorder over two positions. It was described with fixed occupancies of 80:20% and isotropic displacement parameters were applied to the minor part. In addition, a positionally disordered diethyl ether solute molecule is present in the unit cell. It has been described with fixed occupancies of 50:50%.

Table S8. Crystal data and structure refinement of complex **14**.

Identification code	14506	
Empirical formula	$C_{112} H_{133} Mo_2 N_3 O_4 Si_3$	
Color	orange	
Formula weight	1861.36 g·mol^{-1}	
Temperature	100(2) K	
Wavelength	0.71073 Å	
Crystal system	Monoclinic	
Space group	$P2_1/n$, (no. 14)	
Unit cell dimensions	a = 14.5704(6) Å	α= 90°.
	b = 27.4246(13) Å	β= 90.816(2)°.
	c = 25.0257(12) Å	γ = 90°.
Volume	9998.9(8) Å^3	
Z	4	
Density (calculated)	1.236 Mg·m^{-3}	
Absorption coefficient	0.340 mm^{-1}	
F(000)	3936 e	
Crystal size	0.242 x 0.106 x 0.06 mm^3	
θ range for data collection	1.583 to 30.034°.	
Index ranges	-20 ≤ h ≤ 20, -38≤ k ≤ 38, -35 ≤ l ≤ 35	
Reflections collected	379265	
Independent reflections	29260 [R_{int} = 0.0813]	
Reflections with I>2σ(I)	22287	
Completeness to θ = 25.242°	99.9 %	
Absorption correction	Gaussian	
Max. and min. transmission	0.98820 and 0.95950	
Refinement method	Full-matrix least-squares on F^2	
Data / restraints / parameters	29260 / 0 / 1208	
Goodness-of-fit on F^2	1.062	
Final R indices [I>2σ(I)]	R_1 = 0.0414	wR^2 = 0.1011
R indices (all data)	R_1 = 0.0662	wR^2 = 0.1130
Extinction coefficient	n/a	
Largest diff. peak and hole	1.373 and -0.704 e·Å^{-3}	

Table S9. Bond lengths [Å] and angles [°] of complex **14**.

Mo(1)-Mo(2)	2.3440(3)	Mo(1)-O(1)	1.9305(15)
Mo(1)-O(2)	1.9455(14)	Mo(1)-O(3)	1.9409(14)
Mo(2)-N(1)	1.9885(18)	Mo(2)-N(2)	1.9810(18)
Mo(2)-N(3)	1.9954(18)	Si(1)-O(1)	1.6390(16)
Si(1)-C(37)	1.897(3)	Si(1)-C(61)	1.895(3)
Si(1)-C(69)	1.879(3)	Si(2)-O(2)	1.6417(16)
Si(2)-C(54)	1.903(2)	Si(2)-C(77)	1.902(2)
Si(2)-C(85)	1.887(2)	Si(3)-O(3)	1.6420(15)
Si(3)-C(56)	1.902(2)	Si(3)-C(93)	1.890(2)
Si(3)-C(101)	1.876(2)	N(1)-C(1)	1.448(3)
N(1)-C(9)	1.505(3)	N(2)-C(13)	1.451(3)
N(2)-C(21)	1.504(3)	N(3)-C(25)	1.442(3)
N(3)-C(33)	1.504(3)	C(1)-C(2)	1.404(3)
C(1)-C(6)	1.390(3)	C(2)-H(2)	0.9500
C(2)-C(3)	1.394(3)	C(3)-C(4)	1.387(4)
C(3)-C(7)	1.512(4)	C(4)-H(4)	0.9500
C(4)-C(5)	1.389(4)	C(5)-C(6)	1.403(3)
C(5)-C(8)	1.510(4)	C(6)-H(6)	0.9500
C(7)-H(7A)	0.9800	C(7)-H(7B)	0.9800
C(7)-H(7C)	0.9800	C(8)-H(8A)	0.9800
C(8)-H(8B)	0.9800	C(8)-H(8C)	0.9800
C(9)-C(10)	1.537(3)	C(9)-C(11)	1.516(3)
C(9)-C(12)	1.510(3)	C(10)-H(10A)	0.9800
C(10)-H(10B)	0.9800	C(10)-H(10C)	0.9800
C(11)-H(11A)	0.9800	C(11)-H(11B)	0.9800
C(11)-H(11C)	0.9800	C(12)-H(12A)	0.9800
C(12)-H(12B)	0.9800	C(12)-H(12C)	0.9800
C(13)-C(14)	1.391(3)	C(13)-C(18)	1.393(3)
C(14)-H(14)	0.9500	C(14)-C(15)	1.395(3)
C(15)-C(16)	1.387(4)	C(15)-C(19)	1.515(4)
C(16)-H(16)	0.9500	C(16)-C(17)	1.391(4)
C(17)-C(18)	1.393(3)	C(17)-C(20)	1.502(4)
C(18)-H(18)	0.9500	C(19)-H(19A)	0.9800
C(19)-H(19B)	0.9800	C(19)-H(19C)	0.9800
C(20)-H(20A)	0.9800	C(20)-H(20B)	0.9800
C(20)-H(20C)	0.9800	C(21)-C(22)	1.539(3)
C(21)-C(23)	1.519(3)	C(21)-C(24)	1.505(3)
C(22)-H(22A)	0.9800	C(22)-H(22B)	0.9800
C(22)-H(22C)	0.9800	C(23)-H(23A)	0.9800
C(23)-H(23B)	0.9800	C(23)-H(23C)	0.9800
C(24)-H(24A)	0.9800	C(24)-H(24B)	0.9800
C(24)-H(24C)	0.9800	C(25)-C(26)	1.397(3)
C(25)-C(30)	1.399(3)	C(26)-H(26)	0.9500
C(26)-C(27)	1.394(3)	C(27)-C(28)	1.390(4)

C(27)-C(31)	1.511(4)	C(28)-H(28)	0.9500
C(28)-C(29)	1.391(4)	C(29)-C(30)	1.389(3)
C(29)-C(32)	1.512(3)	C(30)-H(30)	0.9500
C(31)-H(31A)	0.9800	C(31)-H(31B)	0.9800
C(31)-H(31C)	0.9800	C(32)-H(32A)	0.9800
C(32)-H(32B)	0.9800	C(32)-H(32C)	0.9800
C(33)-C(34A)	1.523(5)	C(33)-C(34B)	1.57(2)
C(33)-C(35A)	1.518(4)	C(33)-C(35B)	1.440(16)
C(33)-C(36A)	1.513(4)	C(33)-C(36B)	1.612(17)
C(34A)-H(34A)	0.9800	C(34A)-H(34B)	0.9800
C(34A)-H(34C)	0.9800	C(34B)-H(34D)	0.9800
C(34B)-H(34E)	0.9800	C(34B)-H(34F)	0.9800
C(35A)-H(35A)	0.9800	C(35A)-H(35B)	0.9800
C(35A)-H(35C)	0.9800	C(35B)-H(35D)	0.9800
C(35B)-H(35E)	0.9800	C(35B)-H(35F)	0.9800
C(36A)-H(36A)	0.9800	C(36A)-H(36B)	0.9800
C(36A)-H(36C)	0.9800	C(36B)-H(36D)	0.9800
C(36B)-H(36E)	0.9800	C(36B)-H(36F)	0.9800
C(37)-C(38)	1.412(3)	C(37)-C(42)	1.406(4)
C(38)-H(38)	0.9500	C(38)-C(39)	1.379(4)
C(39)-H(39)	0.9500	C(39)-C(40)	1.382(4)
C(40)-H(40)	0.9500	C(40)-C(41)	1.389(4)
C(41)-H(41)	0.9500	C(41)-C(42)	1.402(4)
C(42)-C(43)	1.484(3)	C(43)-C(44)	1.391(3)
C(43)-C(48)	1.394(3)	C(44)-H(44)	0.9500
C(44)-C(45)	1.388(3)	C(45)-C(46)	1.395(3)
C(45)-C(49)	1.483(3)	C(46)-H(46)	0.9500
C(46)-C(47)	1.390(3)	C(47)-C(48)	1.397(3)
C(47)-C(55)	1.486(3)	C(48)-H(48)	0.9500
C(49)-C(50)	1.399(3)	C(49)-C(54)	1.401(3)
C(50)-H(50)	0.9500	C(50)-C(51)	1.382(3)
C(51)-H(51)	0.9500	C(51)-C(52)	1.376(4)
C(52)-H(52)	0.9500	C(52)-C(53)	1.391(3)
C(53)-H(53)	0.9500	C(53)-C(54)	1.411(3)
C(55)-C(56)	1.406(3)	C(55)-C(60)	1.392(3)
C(56)-C(57)	1.403(3)	C(57)-H(57)	0.9500
C(57)-C(58)	1.391(4)	C(58)-H(58)	0.9500
C(58)-C(59)	1.382(4)	C(59)-H(59)	0.9500
C(59)-C(60)	1.388(3)	C(60)-H(60)	0.9500
C(61)-C(62)	1.399(4)	C(61)-C(66)	1.398(4)
C(62)-H(62)	0.9500	C(62)-C(63)	1.402(4)
C(63)-C(64)	1.376(6)	C(63)-C(67)	1.509(5)
C(64)-H(64)	0.9500	C(64)-C(65)	1.384(5)
C(65)-C(66)	1.404(4)	C(65)-C(68)	1.505(5)
C(66)-H(66)	0.9500	C(67)-H(67A)	0.9800
C(67)-H(67B)	0.9800	C(67)-H(67C)	0.9800
C(68)-H(68A)	0.9800	C(68)-H(68B)	0.9800

C(68)-H(68C)	0.9800	C(69)-C(70)	1.391(4)
C(69)-C(74)	1.399(3)	C(70)-H(70)	0.9500
C(70)-C(71)	1.402(3)	C(71)-C(72)	1.387(4)
C(71)-C(75)	1.494(4)	C(72)-H(72)	0.9500
C(72)-C(73)	1.372(4)	C(73)-C(74)	1.390(4)
C(73)-C(76)	1.501(4)	C(74)-H(74)	0.9500
C(75)-H(75A)	0.9800	C(75)-H(75B)	0.9800
C(75)-H(75C)	0.9800	C(76)-H(76A)	0.9800
C(76)-H(76B)	0.9800	C(76)-H(76C)	0.9800
C(77)-C(78)	1.396(4)	C(77)-C(82)	1.402(4)
C(78)-H(78)	0.9500	C(78)-C(79)	1.401(3)
C(79)-C(80)	1.385(5)	C(79)-C(84)	1.508(4)
C(80)-H(80)	0.9500	C(80)-C(81)	1.386(5)
C(81)-C(82)	1.394(4)	C(81)-C(83)	1.505(5)
C(82)-H(82)	0.9500	C(83)-H(83A)	0.9800
C(83)-H(83B)	0.9800	C(83)-H(83C)	0.9800
C(84)-H(84A)	0.9800	C(84)-H(84B)	0.9800
C(84)-H(84C)	0.9800	C(85)-C(86)	1.399(3)
C(85)-C(90)	1.393(3)	C(86)-H(86)	0.9500
C(86)-C(87)	1.392(3)	C(87)-C(88)	1.380(4)
C(87)-C(91)	1.514(3)	C(88)-H(88)	0.9500
C(88)-C(89)	1.394(3)	C(89)-C(90)	1.395(3)
C(89)-C(92)	1.507(3)	C(90)-H(90)	0.9500
C(91)-H(91A)	0.9800	C(91)-H(91B)	0.9800
C(91)-H(91C)	0.9800	C(92)-H(92A)	0.9800
C(92)-H(92B)	0.9800	C(92)-H(92C)	0.9800
C(93)-C(94)	1.400(3)	C(93)-C(98)	1.393(3)
C(94)-H(94)	0.9500	C(94)-C(95)	1.395(3)
C(95)-C(96)	1.379(4)	C(95)-C(99)	1.514(3)
C(96)-H(96)	0.9500	C(96)-C(97)	1.379(4)
C(97)-C(98)	1.400(3)	C(97)-C(100)	1.501(4)
C(98)-H(98)	0.9500	C(99)-H(99A)	0.9800
C(99)-H(99B)	0.9800	C(99)-H(99C)	0.9800
C(100)-H(10D)	0.9800	C(100)-H(10E)	0.9800
C(100)-H(10F)	0.9800	C(101)-C(102)	1.402(3)
C(101)-C(106)	1.394(3)	C(102)-H(102)	0.9500
C(102)-C(103)	1.390(3)	C(103)-C(104)	1.389(4)
C(103)-C(107)	1.510(3)	C(104)-H(104)	0.9500
C(104)-C(105)	1.381(3)	C(105)-C(106)	1.394(3)
C(105)-C(108)	1.503(4)	C(106)-H(106)	0.9500
C(107)-H(10G)	0.9800	C(107)-H(10H)	0.9800
C(107)-H(10I)	0.9800	C(108)-H(10J)	0.9800
C(108)-H(10K)	0.9800	C(108)-H(10L)	0.9800
O(4A)-C(22A)	1.425(7)	O(4A)-C(23A)	1.373(9)
C(21A)-H(21A)	0.9800	C(21A)-H(21B)	0.9800
C(21A)-H(21C)	0.9800	C(21A)-C(22A)	1.468(10)
C(22A)-H(22D)	0.9900	C(22A)-H(22E)	0.9900

C(23A)-H(23D)	0.9900	C(23A)-H(23E)	0.9900
C(23A)-C(24A)	1.392(14)	C(24A)-H(24D)	0.9800
C(24A)-H(24E)	0.9800	C(24A)-H(24F)	0.9800
O(4B)-C(22B)	1.491(10)	O(4B)-C(23B)	1.398(11)
C(21B)-H(21D)	0.9800	C(21B)-H(21E)	0.9800
C(21B)-H(21F)	0.9800	C(21B)-C(22B)	1.197(12)
C(22B)-H(22F)	0.9900	C(22B)-H(22G)	0.9900
C(23B)-H(23F)	0.9900	C(23B)-H(23G)	0.9900
C(23B)-C(24B)	1.389(14)	C(24B)-H(24G)	0.9800
C(24B)-H(24H)	0.9800	C(24B)-H(24I)	0.9800

O(1)-Mo(1)-Mo(2)	105.10(5)	O(1)-Mo(1)-O(2)	113.04(6)
O(1)-Mo(1)-O(3)	111.62(6)	O(2)-Mo(1)-Mo(2)	106.20(5)
O(3)-Mo(1)-Mo(2)	107.29(5)	O(3)-Mo(1)-O(2)	112.94(6)
N(1)-Mo(2)-Mo(1)	109.03(5)	N(1)-Mo(2)-N(3)	111.22(7)
N(2)-Mo(2)-Mo(1)	108.13(5)	N(2)-Mo(2)-N(1)	109.50(8)
N(2)-Mo(2)-N(3)	108.75(7)	N(3)-Mo(2)-Mo(1)	110.15(5)
O(1)-Si(1)-C(37)	114.32(9)	O(1)-Si(1)-C(61)	112.43(10)
O(1)-Si(1)-C(69)	110.95(10)	C(61)-Si(1)-C(37)	103.93(12)
C(69)-Si(1)-C(37)	107.10(11)	C(69)-Si(1)-C(61)	107.62(12)
O(2)-Si(2)-C(54)	114.05(9)	O(2)-Si(2)-C(77)	113.73(9)
O(2)-Si(2)-C(85)	110.81(9)	C(77)-Si(2)-C(54)	102.94(10)
C(85)-Si(2)-C(54)	108.92(10)	C(85)-Si(2)-C(77)	105.78(11)
O(3)-Si(3)-C(56)	115.29(9)	O(3)-Si(3)-C(93)	112.18(9)
O(3)-Si(3)-C(101)	111.00(9)	C(93)-Si(3)-C(56)	103.46(10)
C(101)-Si(3)-C(56)	105.97(10)	C(101)-Si(3)-C(93)	108.40(10)
Si(1)-O(1)-Mo(1)	173.36(11)	Si(2)-O(2)-Mo(1)	173.21(10)
Si(3)-O(3)-Mo(1)	172.12(10)	C(1)-N(1)-Mo(2)	112.14(13)
C(1)-N(1)-C(9)	111.71(17)	C(9)-N(1)-Mo(2)	136.15(14)
C(13)-N(2)-Mo(2)	114.74(14)	C(13)-N(2)-C(21)	111.46(17)
C(21)-N(2)-Mo(2)	133.73(14)	C(25)-N(3)-Mo(2)	112.68(13)
C(25)-N(3)-C(33)	111.48(17)	C(33)-N(3)-Mo(2)	135.83(14)
C(2)-C(1)-N(1)	119.9(2)	C(6)-C(1)-N(1)	120.6(2)
C(6)-C(1)-C(2)	119.4(2)	C(1)-C(2)-H(2)	119.6
C(3)-C(2)-C(1)	120.8(2)	C(3)-C(2)-H(2)	119.6
C(2)-C(3)-C(7)	120.9(3)	C(4)-C(3)-C(2)	118.5(2)
C(4)-C(3)-C(7)	120.6(2)	C(3)-C(4)-H(4)	118.9
C(3)-C(4)-C(5)	122.1(2)	C(5)-C(4)-H(4)	118.9
C(4)-C(5)-C(6)	118.8(2)	C(4)-C(5)-C(8)	121.7(2)

C(6)-C(5)-C(8)	119.5(3)	C(1)-C(6)-C(5)	120.4(2)
C(1)-C(6)-H(6)	119.8	C(5)-C(6)-H(6)	119.8
C(3)-C(7)-H(7A)	109.5	C(3)-C(7)-H(7B)	109.5
C(3)-C(7)-H(7C)	109.5	H(7A)-C(7)-H(7B)	109.5
H(7A)-C(7)-H(7C)	109.5	H(7B)-C(7)-H(7C)	109.5
C(5)-C(8)-H(8A)	109.5	C(5)-C(8)-H(8B)	109.5
C(5)-C(8)-H(8C)	109.5	H(8A)-C(8)-H(8B)	109.5
H(8A)-C(8)-H(8C)	109.5	H(8B)-C(8)-H(8C)	109.5
N(1)-C(9)-C(10)	111.48(19)	N(1)-C(9)-C(11)	110.89(19)
N(1)-C(9)-C(12)	107.76(18)	C(11)-C(9)-C(10)	109.7(2)
C(12)-C(9)-C(10)	107.9(2)	C(12)-C(9)-C(11)	109.0(2)
C(9)-C(10)-H(10A)	109.5	C(9)-C(10)-H(10B)	109.5
C(9)-C(10)-H(10C)	109.5	H(10A)-C(10)-H(10B)	109.5
H(10A)-C(10)-H(10C)	109.5	H(10B)-C(10)-H(10C)	109.5
C(9)-C(11)-H(11A)	109.5	C(9)-C(11)-H(11B)	109.5
C(9)-C(11)-H(11C)	109.5	H(11A)-C(11)-H(11B)	109.5
H(11A)-C(11)-H(11C)	109.5	H(11B)-C(11)-H(11C)	109.5
C(9)-C(12)-H(12A)	109.5	C(9)-C(12)-H(12B)	109.5
C(9)-C(12)-H(12C)	109.5	H(12A)-C(12)-H(12B)	109.5
H(12A)-C(12)-H(12C)	109.5	H(12B)-C(12)-H(12C)	109.5
C(14)-C(13)-N(2)	120.7(2)	C(14)-C(13)-C(18)	119.1(2)
C(18)-C(13)-N(2)	120.0(2)	C(13)-C(14)-H(14)	119.6
C(13)-C(14)-C(15)	120.7(2)	C(15)-C(14)-H(14)	119.6
C(14)-C(15)-C(19)	120.3(2)	C(16)-C(15)-C(14)	118.9(2)
C(16)-C(15)-C(19)	120.8(2)	C(15)-C(16)-H(16)	119.1
C(15)-C(16)-C(17)	121.7(2)	C(17)-C(16)-H(16)	119.1
C(16)-C(17)-C(18)	118.3(2)	C(16)-C(17)-C(20)	120.5(2)
C(18)-C(17)-C(20)	121.2(2)	C(13)-C(18)-C(17)	121.2(2)
C(13)-C(18)-H(18)	119.4	C(17)-C(18)-H(18)	119.4
C(15)-C(19)-H(19A)	109.5	C(15)-C(19)-H(19B)	109.5
C(15)-C(19)-H(19C)	109.5	H(19A)-C(19)-H(19B)	109.5
H(19A)-C(19)-H(19C)	109.5	H(19B)-C(19)-H(19C)	109.5
C(17)-C(20)-H(20A)	109.5	C(17)-C(20)-H(20B)	109.5
C(17)-C(20)-H(20C)	109.5	H(20A)-C(20)-H(20B)	109.5
H(20A)-C(20)-H(20C)	109.5	H(20B)-C(20)-H(20C)	109.5
N(2)-C(21)-C(22)	111.45(19)	N(2)-C(21)-C(23)	110.19(19)

N(2)-C(21)-C(24)	108.55(17)	C(23)-C(21)-C(22)	110.54(19)
C(24)-C(21)-C(22)	107.4(2)	C(24)-C(21)-C(23)	108.6(2)
C(21)-C(22)-H(22A)	109.5	C(21)-C(22)-H(22B)	109.5
C(21)-C(22)-H(22C)	109.5	H(22A)-C(22)-H(22B)	109.5
H(22A)-C(22)-H(22C)	109.5	H(22B)-C(22)-H(22C)	109.5
C(21)-C(23)-H(23A)	109.5	C(21)-C(23)-H(23B)	109.5
C(21)-C(23)-H(23C)	109.5	H(23A)-C(23)-H(23B)	109.5
H(23A)-C(23)-H(23C)	109.5	H(23B)-C(23)-H(23C)	109.5
C(21)-C(24)-H(24A)	109.5	C(21)-C(24)-H(24B)	109.5
C(21)-C(24)-H(24C)	109.5	H(24A)-C(24)-H(24B)	109.5
H(24A)-C(24)-H(24C)	109.5	H(24B)-C(24)-H(24C)	109.5
C(26)-C(25)-N(3)	121.2(2)	C(26)-C(25)-C(30)	118.8(2)
C(30)-C(25)-N(3)	120.0(2)	C(25)-C(26)-H(26)	119.5
C(27)-C(26)-C(25)	121.0(2)	C(27)-C(26)-H(26)	119.5
C(26)-C(27)-C(31)	120.2(2)	C(28)-C(27)-C(26)	118.8(2)
C(28)-C(27)-C(31)	121.0(2)	C(27)-C(28)-H(28)	119.3
C(27)-C(28)-C(29)	121.4(2)	C(29)-C(28)-H(28)	119.3
C(28)-C(29)-C(32)	120.9(2)	C(30)-C(29)-C(28)	119.0(2)
C(30)-C(29)-C(32)	120.2(2)	C(25)-C(30)-H(30)	119.5
C(29)-C(30)-C(25)	121.0(2)	C(29)-C(30)-H(30)	119.5
C(27)-C(31)-H(31A)	109.5	C(27)-C(31)-H(31B)	109.5
C(27)-C(31)-H(31C)	109.5	H(31A)-C(31)-H(31B)	109.5
H(31A)-C(31)-H(31C)	109.5	H(31B)-C(31)-H(31C)	109.5
C(29)-C(32)-H(32A)	109.5	C(29)-C(32)-H(32B)	109.5
C(29)-C(32)-H(32C)	109.5	H(32A)-C(32)-H(32B)	109.5
H(32A)-C(32)-H(32C)	109.5	H(32B)-C(32)-H(32C)	109.5
N(3)-C(33)-C(34A)	111.6(2)	N(3)-C(33)-C(34B)	111.8(9)
N(3)-C(33)-C(35A)	106.48(19)	N(3)-C(33)-C(36A)	111.5(2)
N(3)-C(33)-C(36B)	108.7(6)	C(34B)-C(33)-C(36B)	105.5(10)
C(35A)-C(33)-C(34A)	107.9(3)	C(35B)-C(33)-N(3)	112.2(7)
C(35B)-C(33)-C(34B)	113.5(11)	C(35B)-C(33)-C(36B)	104.6(9)
C(36A)-C(33)-C(34A)	110.9(3)	C(36A)-C(33)-C(35A)	108.3(3)
C(33)-C(34A)-H(34A)	109.5	C(33)-C(34A)-H(34B)	109.5
C(33)-C(34A)-H(34C)	109.5	H(34A)-C(34A)-H(34B)	109.5
H(34A)-C(34A)-H(34C)	109.5	H(34B)-C(34A)-H(34C)	109.5
C(33)-C(34B)-H(34D)	109.5	C(33)-C(34B)-H(34E)	109.5

C(33)-C(34B)-H(34F)	109.5	H(34D)-C(34B)-H(34E)	109.5
H(34D)-C(34B)-H(34F)	109.5	H(34E)-C(34B)-H(34F)	109.5
C(33)-C(35A)-H(35A)	109.5	C(33)-C(35A)-H(35B)	109.5
C(33)-C(35A)-H(35C)	109.5	H(35A)-C(35A)-H(35B)	109.5
H(35A)-C(35A)-H(35C)	109.5	H(35B)-C(35A)-H(35C)	109.5
C(33)-C(35B)-H(35D)	109.5	C(33)-C(35B)-H(35E)	109.5
C(33)-C(35B)-H(35F)	109.5	H(35D)-C(35B)-H(35E)	109.5
H(35D)-C(35B)-H(35F)	109.5	H(35E)-C(35B)-H(35F)	109.5
C(33)-C(36A)-H(36A)	109.5	C(33)-C(36A)-H(36B)	109.5
C(33)-C(36A)-H(36C)	109.5	H(36A)-C(36A)-H(36B)	109.5
H(36A)-C(36A)-H(36C)	109.5	H(36B)-C(36A)-H(36C)	109.5
C(33)-C(36B)-H(36D)	109.5	C(33)-C(36B)-H(36E)	109.5
C(33)-C(36B)-H(36F)	109.5	H(36D)-C(36B)-H(36E)	109.5
H(36D)-C(36B)-H(36F)	109.5	H(36E)-C(36B)-H(36F)	109.5
C(38)-C(37)-Si(1)	117.1(2)	C(42)-C(37)-Si(1)	126.25(17)
C(42)-C(37)-C(38)	116.6(2)	C(37)-C(38)-H(38)	118.9
C(39)-C(38)-C(37)	122.2(3)	C(39)-C(38)-H(38)	118.9
C(38)-C(39)-H(39)	119.8	C(38)-C(39)-C(40)	120.4(2)
C(40)-C(39)-H(39)	119.8	C(39)-C(40)-H(40)	120.3
C(39)-C(40)-C(41)	119.4(3)	C(41)-C(40)-H(40)	120.3
C(40)-C(41)-H(41)	119.8	C(40)-C(41)-C(42)	120.4(3)
C(42)-C(41)-H(41)	119.8	C(37)-C(42)-C(43)	121.4(2)
C(41)-C(42)-C(37)	120.9(2)	C(41)-C(42)-C(43)	117.5(2)
C(44)-C(43)-C(42)	118.7(2)	C(44)-C(43)-C(48)	119.1(2)
C(48)-C(43)-C(42)	122.1(2)	C(43)-C(44)-H(44)	119.5
C(45)-C(44)-C(43)	120.9(2)	C(45)-C(44)-H(44)	119.5
C(44)-C(45)-C(46)	119.5(2)	C(44)-C(45)-C(49)	121.21(19)
C(46)-C(45)-C(49)	119.2(2)	C(45)-C(46)-H(46)	119.8
C(47)-C(46)-C(45)	120.3(2)	C(47)-C(46)-H(46)	119.8
C(46)-C(47)-C(48)	119.49(19)	C(46)-C(47)-C(55)	121.0(2)
C(48)-C(47)-C(55)	119.45(19)	C(43)-C(48)-C(47)	120.6(2)
C(43)-C(48)-H(48)	119.7	C(47)-C(48)-H(48)	119.7
C(50)-C(49)-C(45)	117.7(2)	C(50)-C(49)-C(54)	121.0(2)
C(54)-C(49)-C(45)	121.20(19)	C(49)-C(50)-H(50)	119.7
C(51)-C(50)-C(49)	120.6(2)	C(51)-C(50)-H(50)	119.7
C(50)-C(51)-H(51)	120.1	C(52)-C(51)-C(50)	119.8(2)

C(52)-C(51)-H(51)	120.1	C(51)-C(52)-H(52)	120.2
C(51)-C(52)-C(53)	119.5(2)	C(53)-C(52)-H(52)	120.2
C(52)-C(53)-H(53)	118.8	C(52)-C(53)-C(54)	122.4(2)
C(54)-C(53)-H(53)	118.8	C(49)-C(54)-Si(2)	126.41(16)
C(49)-C(54)-C(53)	116.3(2)	C(53)-C(54)-Si(2)	117.28(17)
C(56)-C(55)-C(47)	120.9(2)	C(60)-C(55)-C(47)	118.2(2)
C(60)-C(55)-C(56)	120.8(2)	C(55)-C(56)-Si(3)	125.99(16)
C(57)-C(56)-Si(3)	117.24(17)	C(57)-C(56)-C(55)	116.7(2)
C(56)-C(57)-H(57)	118.9	C(58)-C(57)-C(56)	122.2(2)
C(58)-C(57)-H(57)	118.9	C(57)-C(58)-H(58)	120.1
C(59)-C(58)-C(57)	119.8(2)	C(59)-C(58)-H(58)	120.1
C(58)-C(59)-H(59)	120.4	C(58)-C(59)-C(60)	119.2(2)
C(60)-C(59)-H(59)	120.4	C(55)-C(60)-H(60)	119.5
C(59)-C(60)-C(55)	120.9(2)	C(59)-C(60)-H(60)	119.5
C(62)-C(61)-Si(1)	123.5(2)	C(66)-C(61)-Si(1)	119.3(2)
C(66)-C(61)-C(62)	117.2(3)	C(61)-C(62)-H(62)	119.2
C(61)-C(62)-C(63)	121.5(3)	C(63)-C(62)-H(62)	119.2
C(62)-C(63)-C(67)	119.3(4)	C(64)-C(63)-C(62)	118.9(3)
C(64)-C(63)-C(67)	121.8(3)	C(63)-C(64)-H(64)	119.0
C(63)-C(64)-C(65)	122.1(3)	C(65)-C(64)-H(64)	119.0
C(64)-C(65)-C(66)	117.9(3)	C(64)-C(65)-C(68)	122.0(3)
C(66)-C(65)-C(68)	120.1(3)	C(61)-C(66)-C(65)	122.3(3)
C(61)-C(66)-H(66)	118.9	C(65)-C(66)-H(66)	118.9
C(63)-C(67)-H(67A)	109.5	C(63)-C(67)-H(67B)	109.5
C(63)-C(67)-H(67C)	109.5	H(67A)-C(67)-H(67B)	109.5
H(67A)-C(67)-H(67C)	109.5	H(67B)-C(67)-H(67C)	109.5
C(65)-C(68)-H(68A)	109.5	C(65)-C(68)-H(68B)	109.5
C(65)-C(68)-H(68C)	109.5	H(68A)-C(68)-H(68B)	109.5
H(68A)-C(68)-H(68C)	109.5	H(68B)-C(68)-H(68C)	109.5
C(70)-C(69)-Si(1)	125.58(18)	C(70)-C(69)-C(74)	117.2(2)
C(74)-C(69)-Si(1)	117.2(2)	C(69)-C(70)-H(70)	118.9
C(69)-C(70)-C(71)	122.2(2)	C(71)-C(70)-H(70)	118.9
C(70)-C(71)-C(75)	121.7(2)	C(72)-C(71)-C(70)	117.9(3)
C(72)-C(71)-C(75)	120.4(2)	C(71)-C(72)-H(72)	119.1
C(73)-C(72)-C(71)	121.8(2)	C(73)-C(72)-H(72)	119.1
C(72)-C(73)-C(74)	119.1(2)	C(72)-C(73)-C(76)	120.3(3)

C(74)-C(73)-C(76)	120.7(3)	C(69)-C(74)-H(74)	119.1
C(73)-C(74)-C(69)	121.8(3)	C(73)-C(74)-H(74)	119.1
C(71)-C(75)-H(75A)	109.5	C(71)-C(75)-H(75B)	109.5
C(71)-C(75)-H(75C)	109.5	H(75A)-C(75)-H(75B)	109.5
H(75A)-C(75)-H(75C)	109.5	H(75B)-C(75)-H(75C)	109.5
C(73)-C(76)-H(76A)	109.5	C(73)-C(76)-H(76B)	109.5
C(73)-C(76)-H(76C)	109.5	H(76A)-C(76)-H(76B)	109.5
H(76A)-C(76)-H(76C)	109.5	H(76B)-C(76)-H(76C)	109.5
C(78)-C(77)-Si(2)	123.21(19)	C(78)-C(77)-C(82)	117.0(2)
C(82)-C(77)-Si(2)	119.73(19)	C(77)-C(78)-H(78)	119.0
C(77)-C(78)-C(79)	122.1(3)	C(79)-C(78)-H(78)	119.0
C(78)-C(79)-C(84)	120.1(3)	C(80)-C(79)-C(78)	118.4(3)
C(80)-C(79)-C(84)	121.6(3)	C(79)-C(80)-H(80)	119.0
C(79)-C(80)-C(81)	121.9(2)	C(81)-C(80)-H(80)	119.0
C(80)-C(81)-C(82)	118.1(3)	C(80)-C(81)-C(83)	121.6(3)
C(82)-C(81)-C(83)	120.3(3)	C(77)-C(82)-H(82)	118.8
C(81)-C(82)-C(77)	122.4(3)	C(81)-C(82)-H(82)	118.8
C(81)-C(83)-H(83A)	109.5	C(81)-C(83)-H(83B)	109.5
C(81)-C(83)-H(83C)	109.5	H(83A)-C(83)-H(83B)	109.5
H(83A)-C(83)-H(83C)	109.5	H(83B)-C(83)-H(83C)	109.5
C(79)-C(84)-H(84A)	109.5	C(79)-C(84)-H(84B)	109.5
C(79)-C(84)-H(84C)	109.5	H(84A)-C(84)-H(84B)	109.5
H(84A)-C(84)-H(84C)	109.5	H(84B)-C(84)-H(84C)	109.5
C(86)-C(85)-Si(2)	117.42(17)	C(90)-C(85)-Si(2)	124.81(16)
C(90)-C(85)-C(86)	117.8(2)	C(85)-C(86)-H(86)	118.9
C(87)-C(86)-C(85)	122.2(2)	C(87)-C(86)-H(86)	118.9
C(86)-C(87)-C(91)	120.7(2)	C(88)-C(87)-C(86)	118.1(2)
C(88)-C(87)-C(91)	121.3(2)	C(87)-C(88)-H(88)	119.0
C(87)-C(88)-C(89)	122.0(2)	C(89)-C(88)-H(88)	119.0
C(88)-C(89)-C(90)	118.5(2)	C(88)-C(89)-C(92)	120.6(2)
C(90)-C(89)-C(92)	121.0(2)	C(85)-C(90)-C(89)	121.5(2)
C(85)-C(90)-H(90)	119.3	C(89)-C(90)-H(90)	119.3
C(87)-C(91)-H(91A)	109.5	C(87)-C(91)-H(91B)	109.5
C(87)-C(91)-H(91C)	109.5	H(91A)-C(91)-H(91B)	109.5
H(91A)-C(91)-H(91C)	109.5	H(91B)-C(91)-H(91C)	109.5
C(89)-C(92)-H(92A)	109.5	C(89)-C(92)-H(92B)	109.5

C(89)-C(92)-H(92C)	109.5	H(92A)-C(92)-H(92B)	109.5
H(92A)-C(92)-H(92C)	109.5	H(92B)-C(92)-H(92C)	109.5
C(94)-C(93)-Si(3)	119.93(17)	C(98)-C(93)-Si(3)	122.89(17)
C(98)-C(93)-C(94)	117.1(2)	C(93)-C(94)-H(94)	118.8
C(95)-C(94)-C(93)	122.3(2)	C(95)-C(94)-H(94)	118.8
C(94)-C(95)-C(99)	121.3(2)	C(96)-C(95)-C(94)	118.1(2)
C(96)-C(95)-C(99)	120.6(2)	C(95)-C(96)-H(96)	118.9
C(97)-C(96)-C(95)	122.2(2)	C(97)-C(96)-H(96)	118.9
C(96)-C(97)-C(98)	118.4(2)	C(96)-C(97)-C(100)	120.5(2)
C(98)-C(97)-C(100)	121.1(2)	C(93)-C(98)-C(97)	121.9(2)
C(93)-C(98)-H(98)	119.1	C(97)-C(98)-H(98)	119.1
C(95)-C(99)-H(99A)	109.5	C(95)-C(99)-H(99B)	109.5
C(95)-C(99)-H(99C)	109.5	H(99A)-C(99)-H(99B)	109.5
H(99A)-C(99)-H(99C)	109.5	H(99B)-C(99)-H(99C)	109.5
C(97)-C(100)-H(10D)	109.5	C(97)-C(100)-H(10E)	109.5
C(97)-C(100)-H(10F)	109.5	H(10D)-C(100)-H(10E)	109.5
H(10D)-C(100)-H(10F)	109.5	H(10E)-C(100)-H(10F)	109.5
C(102)-C(101)-Si(3)	117.60(17)	C(106)-C(101)-Si(3)	124.47(17)
C(106)-C(101)-C(102)	117.7(2)	C(101)-C(102)-H(102)	119.1
C(103)-C(102)-C(101)	121.8(2)	C(103)-C(102)-H(102)	119.1
C(102)-C(103)-C(107)	120.7(2)	C(104)-C(103)-C(102)	118.2(2)
C(104)-C(103)-C(107)	121.2(2)	C(103)-C(104)-H(104)	118.9
C(105)-C(104)-C(103)	122.2(2)	C(105)-C(104)-H(104)	118.9
C(104)-C(105)-C(106)	118.3(2)	C(104)-C(105)-C(108)	121.0(2)
C(106)-C(105)-C(108)	120.8(2)	C(101)-C(106)-H(106)	119.1
C(105)-C(106)-C(101)	121.8(2)	C(105)-C(106)-H(106)	119.1
C(103)-C(107)-H(10G)	109.5	C(103)-C(107)-H(10H)	109.5
C(103)-C(107)-H(10I)	109.5	H(10G)-C(107)-H(10H)	109.5
H(10G)-C(107)-H(10I)	109.5	H(10H)-C(107)-H(10I)	109.5
C(105)-C(108)-H(10J)	109.5	C(105)-C(108)-H(10K)	109.5
C(105)-C(108)-H(10L)	109.5	H(10J)-C(108)-H(10K)	109.5
H(10J)-C(108)-H(10L)	109.5	H(10K)-C(108)-H(10L)	109.5
C(23A)-O(4A)-C(22A)	111.4(6)	H(21A)-C(21A)-H(21B)	109.5
H(21A)-C(21A)-H(21C)	109.5	H(21B)-C(21A)-H(21C)	109.5
C(22A)-C(21A)-H(21A)	109.5	C(22A)-C(21A)-H(21B)	109.5
C(22A)-C(21A)-H(21C)	109.5	O(4A)-C(22A)-C(21A)	105.7(5)

O(4A)-C(22A)-H(22D)	110.6	O(4A)-C(22A)-H(22E)	110.6
C(21A)-C(22A)-H(22D)	110.6	C(21A)-C(22A)-H(22E)	110.6
H(22D)-C(22A)-H(22E)	108.7	O(4A)-C(23A)-H(23D)	109.9
O(4A)-C(23A)-H(23E)	109.9	O(4A)-C(23A)-C(24A)	109.0(8)
H(23D)-C(23A)-H(23E)	108.3	C(24A)-C(23A)-H(23D)	109.9
C(24A)-C(23A)-H(23E)	109.9	C(23A)-C(24A)-H(24D)	109.5
C(23A)-C(24A)-H(24E)	109.5	C(23A)-C(24A)-H(24F)	109.5
H(24D)-C(24A)-H(24E)	109.5	H(24D)-C(24A)-H(24F)	109.5
H(24E)-C(24A)-H(24F)	109.5	C(23B)-O(4B)-C(22B)	122.4(7)
H(21D)-C(21B)-H(21E)	109.5	H(21D)-C(21B)-H(21F)	109.5
H(21E)-C(21B)-H(21F)	109.5	C(22B)-C(21B)-H(21D)	109.5
C(22B)-C(21B)-H(21E)	109.5	C(22B)-C(21B)-H(21F)	109.5
O(4B)-C(22B)-H(22F)	108.0	O(4B)-C(22B)-H(22G)	108.0
C(21B)-C(22B)-O(4B)	117.0(7)	C(21B)-C(22B)-H(22F)	108.0
C(21B)-C(22B)-H(22G)	108.0	H(22F)-C(22B)-H(22G)	107.3
O(4B)-C(23B)-H(23F)	108.0	O(4B)-C(23B)-H(23G)	108.0
H(23F)-C(23B)-H(23G)	107.3	C(24B)-C(23B)-O(4B)	117.1(8)
C(24B)-C(23B)-H(23F)	108.0	C(24B)-C(23B)-H(23G)	108.0
C(23B)-C(24B)-H(24G)	109.5	C(23B)-C(24B)-H(24H)	109.5
C(23B)-C(24B)-H(24I)	109.5	H(24G)-C(24B)-H(24H)	109.5
H(24G)-C(24B)-H(24I)	109.5	H(24H)-C(24B)-H(24I)	109.5

Single Crystal Structure Analysis of Complex 16

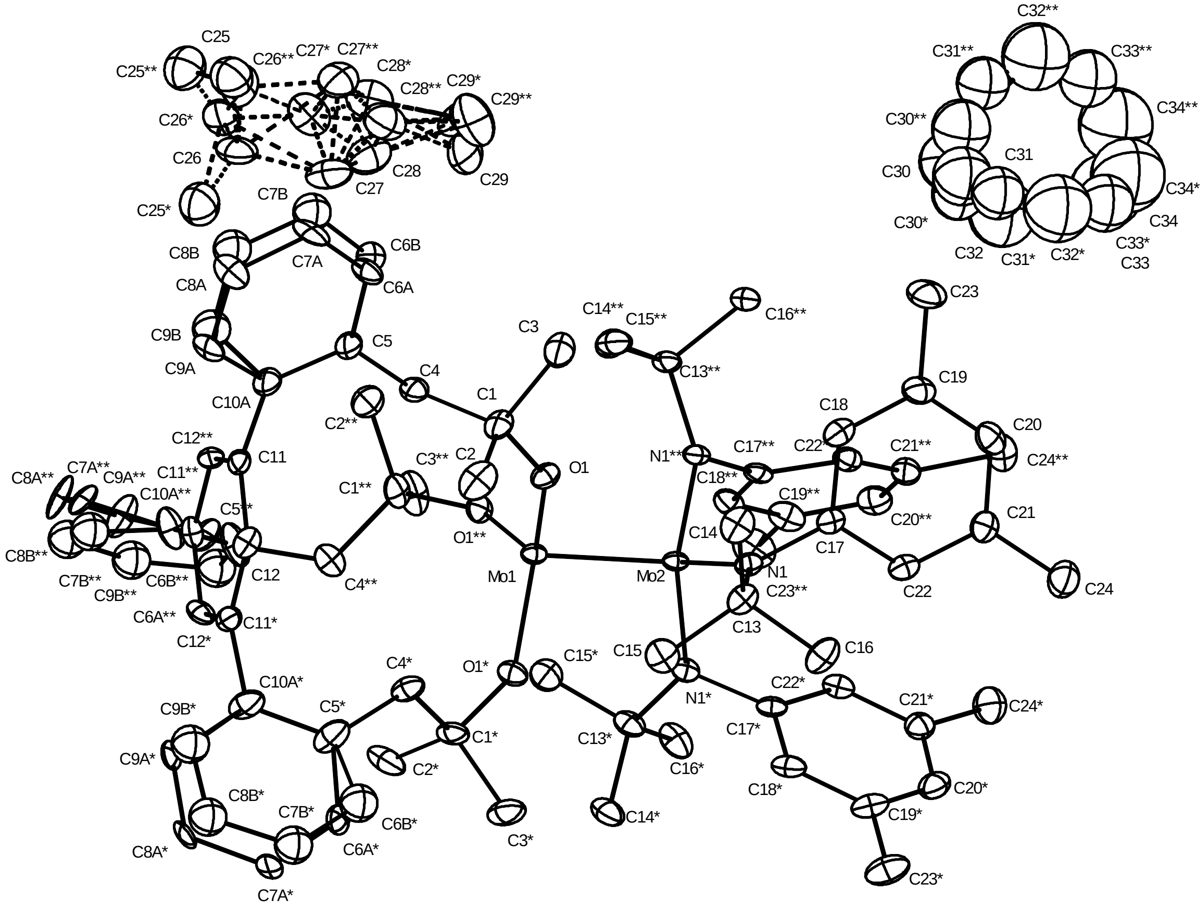

Figure S24. The molecular structure of complex **16**; H atoms removed for clarity.

X-ray Crystal Structure Analysis of 16: C_{83} H_{111} Mo_2 N_3 O_3, M_r = 1390.62 g mol^{-1}, orange plate, crystal size 0.048 x 0.044 x 0.017 mm^3, Trigonal, space group *P*-3 [147], *a* = 11.3823(6) Å, *c* = 33.098(3) Å, *V* = 3713.6(5) Å^3, *T* = 100(2) K, *Z* = 2, D_{calc} = 1.244 g·cm^3, λ = 0.71073 Å, μ(*Mo-K*α) = 0.387 mm^{-1}, Gaussian absorption correction ($T_{\min}$ = 0.9618, $T_{\max}$ = 0.9964), Bruker-AXS Kappa Mach3 with APEX-II detector and IμS microfocus Mo-anode X-ray source, 1.230 < θ < 26.022°, 79459 measured reflections, 4900 independent reflections, 3506 reflections with $I > 2\sigma(I)$, R_{int} = 0.1063. The structure was solved by *SHELXT* and refined by full-matrix least-squares (*SHELXL*) against F^2 to R_1 = 0.0421 [$I > 2\sigma(I)$], wR_2 = 0.0995 [all data], 298 parameters and 73 restraints. **CCDC-2303031**.

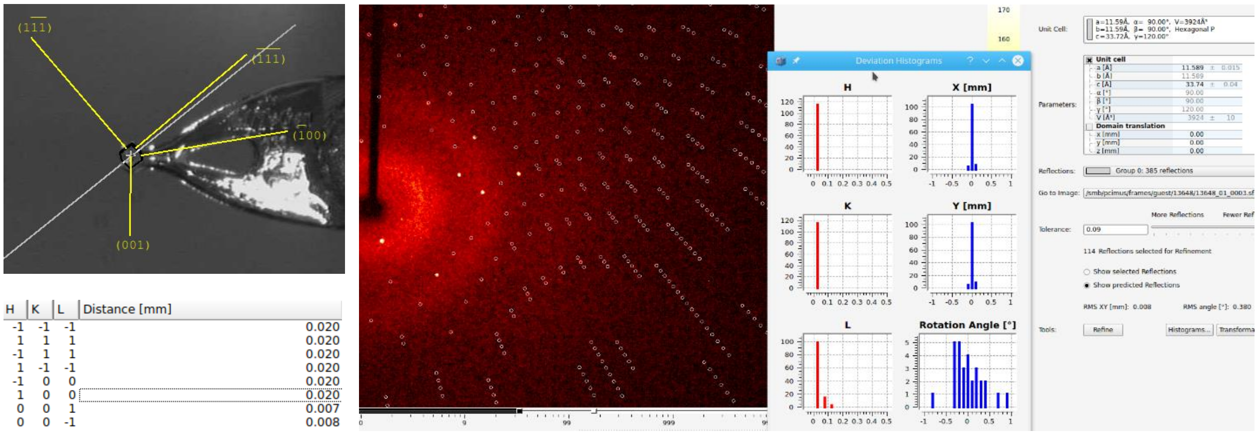

Figure S15. Crystal faces and unit cell determination/refinement of complex **16**.

INTENSITY STATISTICS FOR DATASET

Resolution	#Data	#Theory	%Complete	Redundancy	Mean I	Mean I/s	Rmerge	Rsigma
Inf - 3.30	78	79	98.7	21.61	121.78	80.17	0.0249	0.0088
3.30 - 2.18	184	184	100.0	23.76	59.74	55.38	0.0387	0.0116
2.18 - 1.73	258	258	100.0	23.50	42.86	43.04	0.0541	0.0152
1.73 - 1.50	266	266	100.0	23.78	37.19	38.67	0.0638	0.0173
1.50 - 1.36	270	270	100.0	23.56	31.49	32.85	0.0757	0.0203
1.36 - 1.26	248	248	100.0	23.48	20.74	24.52	0.1115	0.0292
1.26 - 1.19	260	260	100.0	23.23	21.34	23.11	0.1143	0.0303
1.19 - 1.13	247	247	100.0	22.96	20.85	22.41	0.1244	0.0332
1.13 - 1.08	266	266	100.0	20.13	19.12	18.84	0.1353	0.0396
1.08 - 1.03	298	298	100.0	16.33	19.09	16.49	0.1453	0.0466
1.03 - 1.00	245	245	100.0	14.19	15.39	12.81	0.1694	0.0614
1.00 - 0.97	236	236	100.0	13.12	14.75	11.30	0.1763	0.0682
0.97 - 0.94	284	284	100.0	11.98	14.29	10.05	0.1898	0.0761
0.94 - 0.92	220	220	100.0	10.95	11.37	8.01	0.2168	0.0972
0.92 - 0.89	333	333	100.0	10.05	9.88	6.74	0.2425	0.1178
0.89 - 0.87	257	257	100.0	9.67	10.17	6.45	0.2379	0.1201
0.87 - 0.85	316	316	100.0	9.34	10.50	6.63	0.2372	0.1218
0.85 - 0.84	143	143	100.0	9.13	6.10	3.89	0.3596	0.2032
0.84 - 0.82	316	316	100.0	8.96	9.05	5.71	0.2851	0.1482
0.82 - 0.81	175	175	100.0	9.02	8.99	5.35	0.2724	0.1507
0.81 - 0.80	262	271	96.7	7.60	8.24	4.61	0.3041	0.1905
0.90 - 0.80	1567	1576	99.4	9.00	9.26	5.74	0.2658	0.1438
Inf - 0.80	5162	5172	99.8	15.76	20.74	18.44	0.0999	0.0472

A resolution cut off (SHEL 999 0.81) was applied to exclude poorly determined reflections. Several disordered parts are found. A phenyl ring of the asymmetric unit is disordered over two positions with fixed occupancies of 70:30%. It was treated with FLAT instruction to flatten it. Both solute molecules (benzene and pentane) show positional disorder and are located on a crystallographic special position. The occupancies of the solute molecule were described by using fixed occupancies of 33.333%. Isotropic atomic displacement parameters were used for the minor components. Several restraints were applied to model the disordered solute molecules.

Table S10. Crystal data and structure refinement of complex **16**.

Identification code	13648	
Empirical formula	$C_{83}H_{111}Mo_2N_3O_3$	
Color	orange	
Formula weight	1390.62 g·mol^{-1}	
Temperature	100(2) K	
Wavelength	0.71073 Å	
Crystal system	Trigonal	
Space group	*P*-3, (no. 147)	
Unit cell dimensions	a = 11.3823(6) Å	α= 90°.
	b = 11.3823(6) Å	β= 90°.
	c = 33.098(3) Å	γ = 120°.
Volume	3713.6(5) Å^3	
Z	2	
Density (calculated)	1.244 Mg·m^{-3}	
Absorption coefficient	0.387 mm^{-1}	
F(000)	1476 e	
Crystal size	0.048 x 0.044 x 0.017 mm^3	
θ range for data collection	1.230 to 26.022°.	
Index ranges	$-14 \le h \le 14$, $-14 \le k \le 14$, $-40 \le l \le 40$	
Reflections collected	79459	
Independent reflections	4900 [R_{int} = 0.1063]	
Reflections with I>2σ(I)	3506	
Completeness to θ = 25.242°	100.0 %	
Absorption correction	Gaussian	
Max. and min. transmission	0.9964 and 0.9618	
Refinement method	Full-matrix least-squares on F^2	
Data / restraints / parameters	4900 / 73 / 298	
Goodness-of-fit on F^2	1.037	
Final R indices [I>2σ(I)]	R_1 = 0.0421	wR^2 = 0.0866
R indices (all data)	R_1 = 0.0729	wR^2 = 0.0995
Extinction coefficient	n/a	
Largest diff. peak and hole	0.912 and -0.723 e·Å^{-3}	

Table S11. Bond lengths [Å] and angles [°] of complex **16**.

Mo(1)-Mo(2)	2.2955(7)	Mo(1)-O(1)	1.915(2)
Mo(1)-O(1)#1	1.915(2)	Mo(1)-O(1)#2	1.915(2)
Mo(2)-N(1)#1	1.998(3)	Mo(2)-N(1)	1.998(3)
Mo(2)-N(1)#2	1.998(3)	O(1)-C(1)	1.440(4)
N(1)-C(13)	1.508(4)	N(1)-C(17)	1.445(4)
C(1)-C(2)	1.515(5)	C(1)-C(3)	1.538(5)
C(1)-C(4)	1.546(5)	C(2)-H(2A)	0.9800
C(2)-H(2B)	0.9800	C(2)-H(2C)	0.9800
C(3)-H(3A)	0.9800	C(3)-H(3B)	0.9800
C(3)-H(3C)	0.9800	C(4)-H(4A)	0.9900
C(4)-H(4B)	0.9900	C(4)-C(5)	1.518(5)
C(5)-C(6A)	1.401(7)	C(5)-C(6B)	1.458(17)
C(5)-C(10A)	1.396(5)	C(6A)-H(6A)	0.9500
C(6A)-C(7A)	1.381(8)	C(6B)-H(6B)	0.9500
C(6B)-C(7B)	1.35(2)	C(7A)-H(7A)	0.9500
C(7A)-C(8A)	1.396(8)	C(7B)-H(7B)	0.9500
C(7B)-C(8B)	1.380(19)	C(8A)-H(8A)	0.9500
C(8A)-C(9A)	1.387(8)	C(8B)-H(8B)	0.9500
C(8B)-C(9B)	1.36(2)	C(9A)-H(9A)	0.9500
C(9A)-C(10A)	1.447(7)	C(9B)-H(9B)	0.9500
C(9B)-C(10A)	1.326(16)	C(10A)-C(11)	1.487(5)
C(11)-C(12)	1.392(5)	C(11)-C(12)#2	1.384(4)
C(12)-H(12)	0.9500	C(13)-C(14)	1.531(5)
C(13)-C(15)	1.514(5)	C(13)-C(16)	1.542(5)
C(14)-H(14A)	0.9800	C(14)-H(14B)	0.9800
C(14)-H(14C)	0.9800	C(15)-H(15A)	0.9800
C(15)-H(15B)	0.9800	C(15)-H(15C)	0.9800
C(16)-H(16A)	0.9800	C(16)-H(16B)	0.9800
C(16)-H(16C)	0.9800	C(17)-C(18)	1.402(5)
C(17)-C(22)	1.394(4)	C(18)-H(18)	0.9500
C(18)-C(19)	1.389(5)	C(19)-C(20)	1.385(5)
C(19)-C(23)	1.510(5)	C(20)-H(20)	0.9500
C(20)-C(21)	1.394(5)	C(21)-C(22)	1.390(5)
C(21)-C(24)	1.512(5)	C(22)-H(22)	0.9500
C(23)-H(23A)	0.9800	C(23)-H(23B)	0.9800
C(23)-H(23C)	0.9800	C(24)-H(24A)	0.9800
C(24)-H(24B)	0.9800	C(24)-H(24C)	0.9800
C(25)-H(25A)	0.9800	C(25)-H(25B)	0.9800
C(25)-H(25C)	0.9800	C(25)-C(26)	1.5202(10)
C(26)-H(26A)	0.9900	C(26)-H(26B)	0.9900
C(26)-C(27)	1.5201(10)	C(27)-H(27A)	0.9900
C(27)-H(27B)	0.9900	C(27)-C(28)	1.5204(11)
C(28)-H(28A)	0.9900	C(28)-H(28B)	0.9900
C(28)-C(29)	1.5195(11)	C(29)-H(29A)	0.9800

C(29)-H(29B)	0.9800	C(29)-H(29C)	0.9800
C(33)-H(33)	0.9500	C(33)-C(34)	1.3900
C(33)-C(32)	1.3900	C(34)-H(34)	0.9500
C(34)-C(35)	1.3900	C(35)-H(35)	0.9500
C(35)-C(30)	1.3900	C(30)-H(30)	0.9500
C(30)-C(31)	1.3900	C(31)-H(31)	0.9500
C(31)-C(32)	1.3900	C(32)-H(32)	0.9500

O(1)#1-Mo(1)-Mo(2)	100.44(7)	O(1)#2-Mo(1)-Mo(2)	100.44(7)
O(1)-Mo(1)-Mo(2)	100.44(7)	O(1)-Mo(1)-O(1)#1	116.79(4)
O(1)#1-Mo(1)-O(1)#2	116.79(4)	O(1)-Mo(1)-O(1)#2	116.79(4)
N(1)#2-Mo(2)-Mo(1)	106.29(8)	N(1)-Mo(2)-Mo(1)	106.29(8)
N(1)#1-Mo(2)-Mo(1)	106.29(8)	N(1)#1-Mo(2)-N(1)#2	112.46(7)
N(1)#2-Mo(2)-N(1)	112.45(7)	N(1)#1-Mo(2)-N(1)	112.46(7)
C(1)-O(1)-Mo(1)	144.7(2)	C(13)-N(1)-Mo(2)	138.2(2)
C(17)-N(1)-Mo(2)	109.92(19)	C(17)-N(1)-C(13)	111.9(2)
O(1)-C(1)-C(2)	108.3(3)	O(1)-C(1)-C(3)	106.8(3)
O(1)-C(1)-C(4)	109.5(3)	C(2)-C(1)-C(3)	110.2(3)
C(2)-C(1)-C(4)	110.7(3)	C(3)-C(1)-C(4)	111.1(3)
C(1)-C(2)-H(2A)	109.5	C(1)-C(2)-H(2B)	109.5
C(1)-C(2)-H(2C)	109.5	H(2A)-C(2)-H(2B)	109.5
H(2A)-C(2)-H(2C)	109.5	H(2B)-C(2)-H(2C)	109.5
C(1)-C(3)-H(3A)	109.5	C(1)-C(3)-H(3B)	109.5
C(1)-C(3)-H(3C)	109.5	H(3A)-C(3)-H(3B)	109.5
H(3A)-C(3)-H(3C)	109.5	H(3B)-C(3)-H(3C)	109.5
C(1)-C(4)-H(4A)	107.9	C(1)-C(4)-H(4B)	107.9
H(4A)-C(4)-H(4B)	107.2	C(5)-C(4)-C(1)	117.4(3)
C(5)-C(4)-H(4A)	107.9	C(5)-C(4)-H(4B)	107.9
C(6A)-C(5)-C(4)	123.5(4)	C(6B)-C(5)-C(4)	111.7(7)
C(10A)-C(5)-C(4)	121.9(3)	C(10A)-C(5)-C(6A)	114.4(4)
C(10A)-C(5)-C(6B)	124.7(7)	C(5)-C(6A)-H(6A)	117.5
C(7A)-C(6A)-C(5)	125.1(5)	C(7A)-C(6A)-H(6A)	117.5
C(5)-C(6B)-H(6B)	122.7	C(7B)-C(6B)-C(5)	114.6(13)
C(7B)-C(6B)-H(6B)	122.7	C(6A)-C(7A)-H(7A)	120.2
C(6A)-C(7A)-C(8A)	119.5(5)	C(8A)-C(7A)-H(7A)	120.2
C(6B)-C(7B)-H(7B)	119.2	C(6B)-C(7B)-C(8B)	121.5(14)
C(8B)-C(7B)-H(7B)	119.2	C(7A)-C(8A)-H(8A)	120.5
C(9A)-C(8A)-C(7A)	119.0(5)	C(9A)-C(8A)-H(8A)	120.5
C(7B)-C(8B)-H(8B)	120.6	C(9B)-C(8B)-C(7B)	118.8(13)
C(9B)-C(8B)-H(8B)	120.6	C(8A)-C(9A)-H(9A)	120.3
C(8A)-C(9A)-C(10A)	119.4(5)	C(10A)-C(9A)-H(9A)	120.3
C(8B)-C(9B)-H(9B)	117.2	C(10A)-C(9B)-C(8B)	125.7(13)
C(10A)-C(9B)-H(9B)	117.2	C(5)-C(10A)-C(9A)	122.3(4)
C(5)-C(10A)-C(11)	120.3(3)	C(9A)-C(10A)-C(11)	117.3(4)
C(9B)-C(10A)-C(5)	113.2(7)	C(9B)-C(10A)-C(11)	124.0(7)
C(12)-C(11)-C(10A)	119.7(3)	C(12)#2-C(11)-C(10A)	121.5(3)
C(12)#2-C(11)-C(12)	118.6(3)	C(11)#1-C(12)-C(11)	121.4(3)

C(11)#1-C(12)-H(12)	119.3	C(11)-C(12)-H(12)	119.3
N(1)-C(13)-C(14)	111.1(3)	N(1)-C(13)-C(15)	109.2(3)
N(1)-C(13)-C(16)	111.0(3)	C(14)-C(13)-C(16)	108.3(3)
C(15)-C(13)-C(14)	109.4(3)	C(15)-C(13)-C(16)	107.8(3)
C(13)-C(14)-H(14A)	109.5	C(13)-C(14)-H(14B)	109.5
C(13)-C(14)-H(14C)	109.5	H(14A)-C(14)-H(14B)	109.5
H(14A)-C(14)-H(14C)	109.5	H(14B)-C(14)-H(14C)	109.5
C(13)-C(15)-H(15A)	109.5	C(13)-C(15)-H(15B)	109.5
C(13)-C(15)-H(15C)	109.5	H(15A)-C(15)-H(15B)	109.5
H(15A)-C(15)-H(15C)	109.5	H(15B)-C(15)-H(15C)	109.5
C(13)-C(16)-H(16A)	109.5	C(13)-C(16)-H(16B)	109.5
C(13)-C(16)-H(16C)	109.5	H(16A)-C(16)-H(16B)	109.5
H(16A)-C(16)-H(16C)	109.5	H(16B)-C(16)-H(16C)	109.5
C(18)-C(17)-N(1)	120.1(3)	C(22)-C(17)-N(1)	121.3(3)
C(22)-C(17)-C(18)	118.6(3)	C(17)-C(18)-H(18)	119.4
C(19)-C(18)-C(17)	121.1(3)	C(19)-C(18)-H(18)	119.4
C(18)-C(19)-C(23)	120.5(3)	C(20)-C(19)-C(18)	118.9(3)
C(20)-C(19)-C(23)	120.6(3)	C(19)-C(20)-H(20)	119.3
C(19)-C(20)-C(21)	121.5(3)	C(21)-C(20)-H(20)	119.3
C(20)-C(21)-C(24)	121.2(3)	C(22)-C(21)-C(20)	118.8(3)
C(22)-C(21)-C(24)	120.0(3)	C(17)-C(22)-H(22)	119.4
C(21)-C(22)-C(17)	121.2(3)	C(21)-C(22)-H(22)	119.4
C(19)-C(23)-H(23A)	109.5	C(19)-C(23)-H(23B)	109.5
C(19)-C(23)-H(23C)	109.5	H(23A)-C(23)-H(23B)	109.5
H(23A)-C(23)-H(23C)	109.5	H(23B)-C(23)-H(23C)	109.5
C(21)-C(24)-H(24A)	109.5	C(21)-C(24)-H(24B)	109.5
C(21)-C(24)-H(24C)	109.5	H(24A)-C(24)-H(24B)	109.5
H(24A)-C(24)-H(24C)	109.5	H(24B)-C(24)-H(24C)	109.5
H(25A)-C(25)-H(25B)	109.5	H(25A)-C(25)-H(25C)	109.5
H(25B)-C(25)-H(25C)	109.5	C(26)-C(25)-H(25A)	109.5
C(26)-C(25)-H(25B)	109.5	C(26)-C(25)-H(25C)	109.5
C(25)-C(26)-H(26A)	110.4	C(25)-C(26)-H(26B)	110.4
H(26A)-C(26)-H(26B)	108.6	C(27)-C(26)-C(25)	106.7(9)
C(27)-C(26)-H(26A)	110.4	C(27)-C(26)-H(26B)	110.4
C(26)-C(27)-H(27A)	111.0	C(26)-C(27)-H(27B)	111.0
C(26)-C(27)-C(28)	103.6(9)	H(27A)-C(27)-H(27B)	109.0
C(28)-C(27)-H(27A)	111.0	C(28)-C(27)-H(27B)	111.0
C(27)-C(28)-H(28A)	113.2	C(27)-C(28)-H(28B)	113.2
H(28A)-C(28)-H(28B)	110.5	C(29)-C(28)-C(27)	92.6(8)
C(29)-C(28)-H(28A)	113.2	C(29)-C(28)-H(28B)	113.2
C(28)-C(29)-H(29A)	109.5	C(28)-C(29)-H(29B)	109.5
C(28)-C(29)-H(29C)	109.5	H(29A)-C(29)-H(29B)	109.5
H(29A)-C(29)-H(29C)	109.5	H(29B)-C(29)-H(29C)	109.5
C(34)-C(33)-H(33)	120.0	C(34)-C(33)-C(32)	120.0
C(32)-C(33)-H(33)	120.0	C(33)-C(34)-H(34)	120.0
C(33)-C(34)-C(35)	120.0	C(35)-C(34)-H(34)	120.0
C(34)-C(35)-H(35)	120.0	C(30)-C(35)-C(34)	120.0

C(30)-C(35)-H(35)	120.0	C(35)-C(30)-H(30)	120.0
C(35)-C(30)-C(31)	120.0	C(31)-C(30)-H(30)	120.0
C(30)-C(31)-H(31)	120.0	C(32)-C(31)-C(30)	120.0
C(32)-C(31)-H(31)	120.0	C(33)-C(32)-H(32)	120.0
C(31)-C(32)-C(33)	120.0	C(31)-C(32)-H(32)	120.0

Symmetry transformations used to generate equivalent atoms:
#1 -x+y+1,-x+2,z   #2 -y+2,x-y+1,z

Single Crystal Structure Analysis of Complex 17

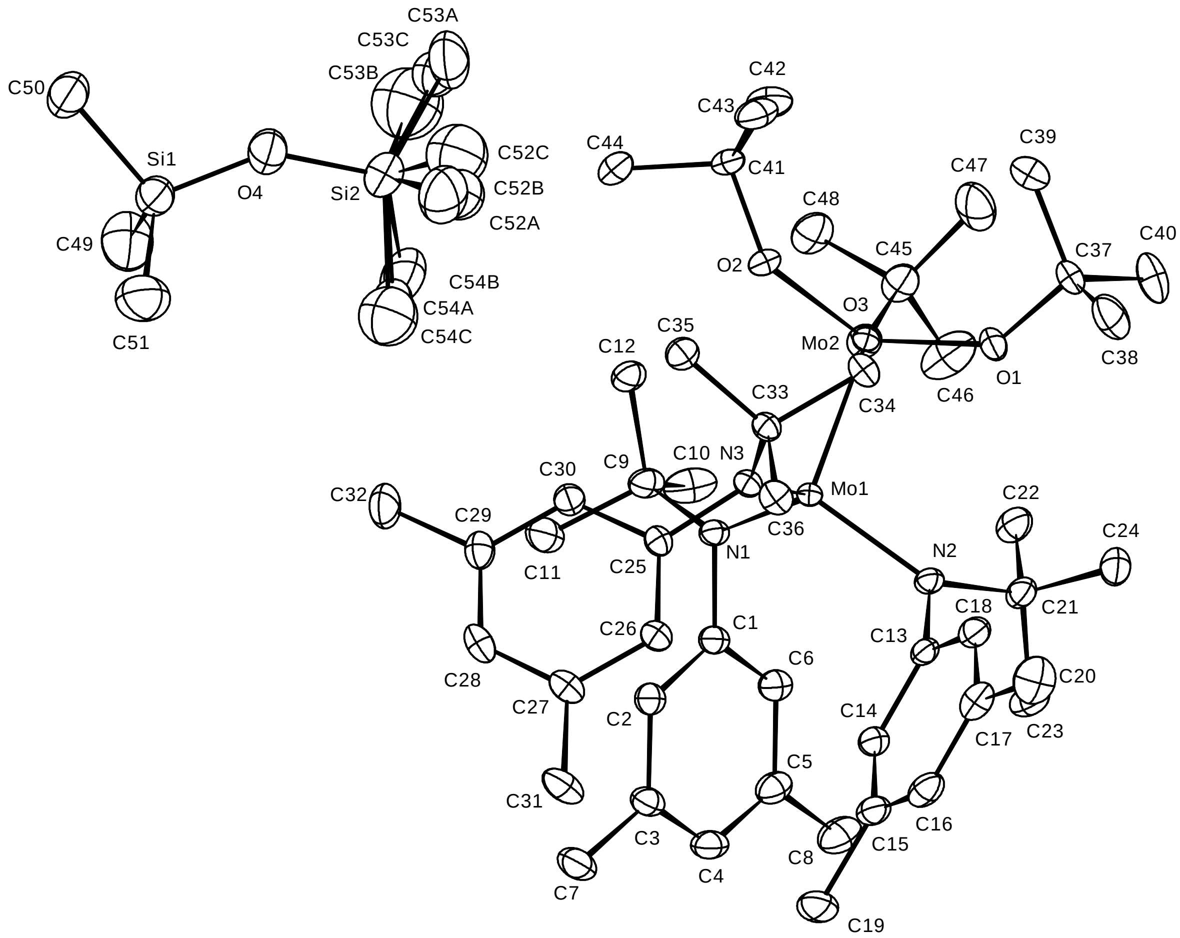

Figure S26. The molecular structure of complex **17**; H atoms removed for clarity.

X-ray Crystal Structure Analysis of 17: C_{54} H_{99} Mo_2 N_3 O_4 Si_2, M_r = 1102.42 g mol^{-1}, orange needle, crystal size 0.225 x 0.051 x 0.021 mm^3, Triclinic, space group *P*-1 [2], *a* = 13.6675(5) Å, , b = 15.2361(5) Å, c = 15.6598(5) Å, α= 65.205(2)°, β = 85.004(2)°, γ = 88.036(2), *V* = 2949.11(18) Å^3, *T* = 100(2) K, *Z* = 2, D_{calc} = 1.241 g·cm^3, λ = 0.71073 Å, $\mu(Mo\text{-}K_\alpha)$ = 0.508 mm^{-1}, Gaussian absorption correction ($T_{\min}$ = 0.8586, $T_{\max}$ = 0.9761), Bruker AXS D8-Venture diffractometer with IµS Diamond Mo-anode X-ray source and PHOTON III detector, 2.244 < θ < 31.069°, 580534 measured reflections, 18822 independent reflections, 15563 reflections with $I > 2\sigma(I)$, R_{int} = 0.1070. The structure was solved by *SHELXT* and refined by full-matrix least-squares (*SHELXL*) against F^2 to R_1 = 0.0337 [$I > 2\sigma(I)$], wR_2 = 0.0741 [all data], 646 parameters and 0 restraints. **CCDC-2303033**.

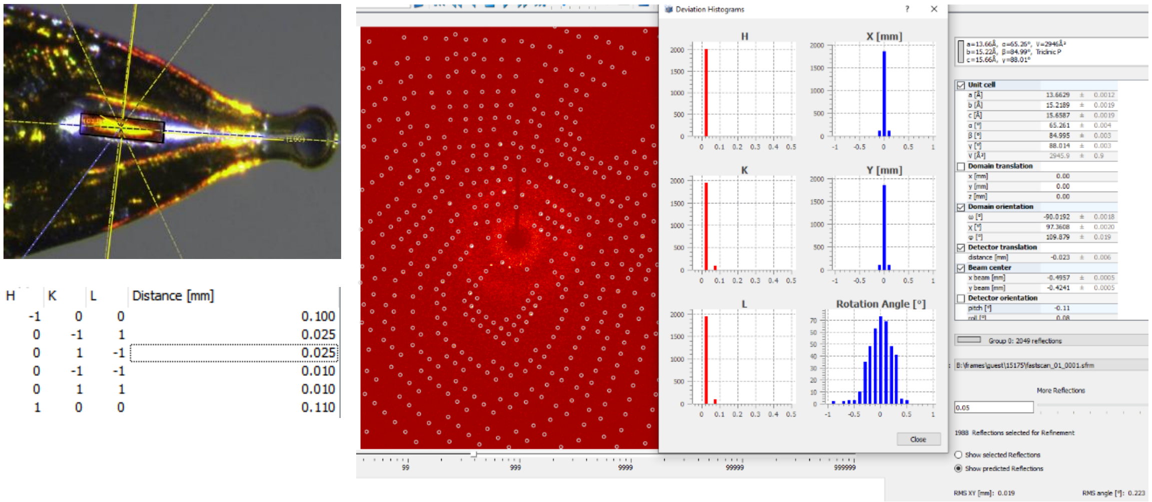

Figure S27. Crystal faces and unit cell determination/refinement of complex **17**.

INTENSITY STATISTICS FOR DATASET

Resolution	#Data	#Theory	%Complete	Redundancy	Mean I	Mean I/s	Rmerge	Rsigma
Inf - 2.74	283	296	95.6	49.37	120.97	103.35	0.0375	0.0133
2.74 - 1.86	664	664	100.0	56.84	77.14	86.40	0.0411	0.0077
1.86 - 1.48	945	945	100.0	53.19	61.82	72.32	0.0536	0.0095
1.48 - 1.29	970	970	100.0	41.25	40.58	49.56	0.0716	0.0139
1.29 - 1.17	972	972	100.0	36.66	29.45	36.29	0.0906	0.0186
1.17 - 1.09	927	927	100.0	34.37	27.49	33.26	0.0990	0.0211
1.09 - 1.03	884	884	100.0	32.74	26.25	30.76	0.1076	0.0233
1.03 - 0.97	1122	1122	100.0	30.60	23.15	26.98	0.1214	0.0271
0.97 - 0.93	906	906	100.0	30.24	17.75	21.75	0.1485	0.0334
0.93 - 0.89	1084	1084	100.0	28.42	16.79	19.67	0.1619	0.0370
0.89 - 0.86	931	931	100.0	27.93	14.02	16.69	0.1823	0.0430
0.86 - 0.84	706	706	100.0	27.79	13.86	16.25	0.1933	0.0455
0.84 - 0.81	1226	1226	100.0	25.98	12.06	13.71	0.2100	0.0541
0.81 - 0.79	895	895	100.0	26.06	11.41	13.10	0.2265	0.0572
0.79 - 0.77	1002	1002	100.0	25.78	10.83	12.29	0.2394	0.0612
0.77 - 0.75	1094	1094	100.0	24.97	10.21	11.19	0.2568	0.0674
0.75 - 0.74	621	621	100.0	24.50	9.63	10.54	0.2716	0.0727
0.74 - 0.72	1266	1266	100.0	24.25	8.80	9.56	0.2978	0.0806
0.72 - 0.71	750	750	100.0	21.58	8.24	8.68	0.3331	0.0934
0.71 - 0.70	763	763	100.0	21.84	7.67	7.97	0.3553	0.1004
0.70 - 0.69	811	884	91.7	18.38	7.55	7.47	0.3594	0.1170
0.79 - 0.69	6307	6380	98.9	23.22	9.08	9.81	0.2897	0.0808
Inf - 0.69	18822	18908	99.5	30.70	22.63	25.77	0.1019	0.0293

One trimethylsilyl group of the HMDSO solute shows a rotational disorder over three positions. It has been described with fixed occupancies of 60:25:15% and isotropic displacement parameters have been applied to the minor part.

Table S12. Crystal data and structure refinement of complex **17**.

Identification code	15175	
Empirical formula	$C_{54}H_{99}Mo_2N_3O_4Si_2$	
Color	orange	
Formula weight	1102.42 g·mol^{-1}	
Temperature	100(2) K	
Wavelength	0.71073 Å	
Crystal system	Triclinic	
Space group	*P*-1, (no. 2)	
Unit cell dimensions	a = 13.6675(5) Å	α= 65.205(2)°.
	b = 15.2361(5) Å	β= 85.004(2)°.
	c = 15.6598(5) Å	γ = 88.036(2)°.
Volume	2949.11(18) Å^3	
Z	2	
Density (calculated)	1.241 Mg·m^{-3}	
Absorption coefficient	0.508 mm^{-1}	
F(000)	1176 e	
Crystal size	0.225 x 0.051 x 0.021 mm^3	
θ range for data collection	2.244 to 31.069°.	
Index ranges	-19 ≤ h ≤ 19, -22≤ k ≤ 22, -22 ≤ l ≤ 22	
Reflections collected	580534	
Independent reflections	18822 [R_{int} = 0.1070]	
Reflections with I>2σ(I)	15563	
Completeness to θ = 25.242°	99.9 %	
Absorption correction	Numerical	
Max. and min. transmission	0.9761 and 0.8586	
Refinement method	Full-matrix least-squares on F^2	
Data / restraints / parameters	18822 / 0 / 646	
Goodness-of-fit on F^2	1.074	
Final R indices [I>2σ(I)]	R_1 = 0.0337	wR^2 = 0.0681
R indices (all data)	R_1 = 0.0472	wR^2 = 0.0741
Extinction coefficient	n/a	
Largest diff. peak and hole	0.691 and -0.698 e·Å^{-3}	

Table S13. Bond lengths [Å] and angles [°] of complex **17**.

Mo(1)-Mo(2)	2.2944(2)	Mo(1)-N(1)	1.9893(14)
Mo(1)-N(2)	1.9910(14)	Mo(1)-N(3)	2.0010(14)
Mo(2)-O(1)	1.9147(12)	Mo(2)-O(2)	1.9122(12)
Mo(2)-O(3)	1.9175(13)	O(1)-C(37)	1.443(2)
O(2)-C(41)	1.441(2)	O(3)-C(45)	1.444(2)
N(1)-C(1)	1.454(2)	N(1)-C(9)	1.503(2)
N(2)-C(13)	1.444(2)	N(2)-C(21)	1.501(2)
N(3)-C(25)	1.444(2)	N(3)-C(33)	1.503(2)
C(1)-C(2)	1.396(2)	C(1)-C(6)	1.396(2)
C(2)-H(2)	0.9500	C(2)-C(3)	1.394(2)
C(3)-C(4)	1.390(3)	C(3)-C(7)	1.511(3)
C(4)-H(4)	0.9500	C(4)-C(5)	1.392(3)
C(5)-C(6)	1.395(2)	C(5)-C(8)	1.508(3)
C(6)-H(6)	0.9500	C(7)-H(7A)	0.9800
C(7)-H(7B)	0.9800	C(7)-H(7C)	0.9800
C(8)-H(8A)	0.9800	C(8)-H(8B)	0.9800
C(8)-H(8C)	0.9800	C(9)-C(10)	1.531(3)
C(9)-C(11)	1.538(3)	C(9)-C(12)	1.525(2)
C(10)-H(10A)	0.9800	C(10)-H(10B)	0.9800
C(10)-H(10C)	0.9800	C(11)-H(11A)	0.9800
C(11)-H(11B)	0.9800	C(11)-H(11C)	0.9800
C(12)-H(12A)	0.9800	C(12)-H(12B)	0.9800
C(12)-H(12C)	0.9800	C(13)-C(14)	1.399(2)
C(13)-C(18)	1.394(2)	C(14)-H(14)	0.9500
C(14)-C(15)	1.398(2)	C(15)-C(16)	1.387(3)
C(15)-C(19)	1.504(3)	C(16)-H(16)	0.9500
C(16)-C(17)	1.390(3)	C(17)-C(18)	1.390(2)
C(17)-C(20)	1.506(3)	C(18)-H(18)	0.9500
C(19)-H(19A)	0.9800	C(19)-H(19B)	0.9800
C(19)-H(19C)	0.9800	C(20)-H(20A)	0.9800
C(20)-H(20B)	0.9800	C(20)-H(20C)	0.9800
C(21)-C(22)	1.524(2)	C(21)-C(23)	1.535(3)
C(21)-C(24)	1.532(3)	C(22)-H(22A)	0.9800
C(22)-H(22B)	0.9800	C(22)-H(22C)	0.9800
C(23)-H(23A)	0.9800	C(23)-H(23B)	0.9800
C(23)-H(23C)	0.9800	C(24)-H(24A)	0.9800
C(24)-H(24B)	0.9800	C(24)-H(24C)	0.9800
C(25)-C(26)	1.399(2)	C(25)-C(30)	1.400(2)
C(26)-H(26)	0.9500	C(26)-C(27)	1.398(2)
C(27)-C(28)	1.390(3)	C(27)-C(31)	1.509(3)
C(28)-H(28)	0.9500	C(28)-C(29)	1.390(3)
C(29)-C(30)	1.393(2)	C(29)-C(32)	1.507(3)
C(30)-H(30)	0.9500	C(31)-H(31A)	0.9800
C(31)-H(31B)	0.9800	C(31)-H(31C)	0.9800

C(32)-H(32A)	0.9800	C(32)-H(32B)	0.9800
C(32)-H(32C)	0.9800	C(33)-C(34)	1.522(2)
C(33)-C(35)	1.536(2)	C(33)-C(36)	1.534(2)
C(34)-H(34A)	0.9800	C(34)-H(34B)	0.9800
C(34)-H(34C)	0.9800	C(35)-H(35A)	0.9800
C(35)-H(35B)	0.9800	C(35)-H(35C)	0.9800
C(36)-H(36A)	0.9800	C(36)-H(36B)	0.9800
C(36)-H(36C)	0.9800	C(37)-C(38)	1.526(3)
C(37)-C(39)	1.517(3)	C(37)-C(40)	1.517(3)
C(38)-H(38A)	0.9800	C(38)-H(38B)	0.9800
C(38)-H(38C)	0.9800	C(39)-H(39A)	0.9800
C(39)-H(39B)	0.9800	C(39)-H(39C)	0.9800
C(40)-H(40A)	0.9800	C(40)-H(40B)	0.9800
C(40)-H(40C)	0.9800	C(41)-C(42)	1.519(3)
C(41)-C(43)	1.522(3)	C(41)-C(44)	1.528(3)
C(42)-H(42A)	0.9800	C(42)-H(42B)	0.9800
C(42)-H(42C)	0.9800	C(43)-H(43A)	0.9800
C(43)-H(43B)	0.9800	C(43)-H(43C)	0.9800
C(44)-H(44A)	0.9800	C(44)-H(44B)	0.9800
C(44)-H(44C)	0.9800	C(45)-C(46)	1.526(3)
C(45)-C(47)	1.520(3)	C(45)-C(48)	1.527(3)
C(46)-H(46A)	0.9800	C(46)-H(46B)	0.9800
C(46)-H(46C)	0.9800	C(47)-H(47A)	0.9800
C(47)-H(47B)	0.9800	C(47)-H(47C)	0.9800
C(48)-H(48A)	0.9800	C(48)-H(48B)	0.9800
C(48)-H(48C)	0.9800	Si(1)-O(4)	1.6310(18)
Si(1)-C(49)	1.855(3)	Si(1)-C(50)	1.850(2)
Si(1)-C(51)	1.853(3)	Si(2)-O(4)	1.6295(18)
Si(2)-C(52C)	1.67(3)	Si(2)-C(52A)	1.943(9)
Si(2)-C(52B)	1.74(3)	Si(2)-C(54C)	1.987(19)
Si(2)-C(53A)	1.884(6)	Si(2)-C(53B)	1.869(17)
Si(2)-C(53C)	1.83(3)	Si(2)-C(54A)	1.840(7)
Si(2)-C(54B)	1.898(18)	C(49)-H(49A)	0.9800
C(49)-H(49B)	0.9800	C(49)-H(49C)	0.9800
C(50)-H(50A)	0.9800	C(50)-H(50B)	0.9800
C(50)-H(50C)	0.9800	C(51)-H(51A)	0.9800
C(51)-H(51B)	0.9800	C(51)-H(51C)	0.9800
C(52C)-H(52A)	0.9800	C(52C)-H(52B)	0.9800
C(52C)-H(52C)	0.9800	C(52A)-H(52D)	0.9800
C(52A)-H(52E)	0.9800	C(52A)-H(52F)	0.9800
C(52B)-H(52G)	0.9800	C(52B)-H(52H)	0.9800
C(52B)-H(52I)	0.9800	C(54C)-H(54A)	0.9800
C(54C)-H(54B)	0.9800	C(54C)-H(54C)	0.9800
C(53A)-H(53A)	0.9800	C(53A)-H(53B)	0.9800
C(53A)-H(53C)	0.9800	C(53B)-H(53D)	0.9800
C(53B)-H(53E)	0.9800	C(53B)-H(53F)	0.9800
C(53C)-H(53G)	0.9800	C(53C)-H(53H)	0.9800

C(53C)-H(53I)	0.9800	C(54A)-H(54D)	0.9800
C(54A)-H(54E)	0.9800	C(54A)-H(54F)	0.9800
C(54B)-H(54G)	0.9800	C(54B)-H(54H)	0.9800
C(54B)-H(54I)	0.9800		

N(1)-Mo(1)-Mo(2)	106.72(4)	N(1)-Mo(1)-N(2)	112.27(6)
N(1)-Mo(1)-N(3)	113.55(6)	N(2)-Mo(1)-Mo(2)	105.58(4)
N(2)-Mo(1)-N(3)	111.97(6)	N(3)-Mo(1)-Mo(2)	106.07(4)
O(1)-Mo(2)-Mo(1)	99.49(4)	O(1)-Mo(2)-O(3)	116.90(5)
O(2)-Mo(2)-Mo(1)	99.14(4)	O(2)-Mo(2)-O(1)	117.76(5)
O(2)-Mo(2)-O(3)	115.89(5)	O(3)-Mo(2)-Mo(1)	102.41(4)
C(37)-O(1)-Mo(2)	139.23(11)	C(41)-O(2)-Mo(2)	139.38(11)
C(45)-O(3)-Mo(2)	139.44(12)	C(1)-N(1)-Mo(1)	109.60(10)
C(1)-N(1)-C(9)	111.86(13)	C(9)-N(1)-Mo(1)	138.48(11)
C(13)-N(2)-Mo(1)	110.96(10)	C(13)-N(2)-C(21)	112.34(13)
C(21)-N(2)-Mo(1)	136.69(11)	C(25)-N(3)-Mo(1)	111.21(10)
C(25)-N(3)-C(33)	111.45(12)	C(33)-N(3)-Mo(1)	137.20(10)
C(2)-C(1)-N(1)	120.62(15)	C(6)-C(1)-N(1)	120.60(15)
C(6)-C(1)-C(2)	118.73(16)	C(1)-C(2)-H(2)	119.6
C(3)-C(2)-C(1)	120.87(17)	C(3)-C(2)-H(2)	119.6
C(2)-C(3)-C(7)	119.99(17)	C(4)-C(3)-C(2)	119.14(17)
C(4)-C(3)-C(7)	120.87(16)	C(3)-C(4)-H(4)	119.3
C(3)-C(4)-C(5)	121.31(16)	C(5)-C(4)-H(4)	119.3
C(4)-C(5)-C(6)	118.59(17)	C(4)-C(5)-C(8)	120.94(17)
C(6)-C(5)-C(8)	120.46(18)	C(1)-C(6)-H(6)	119.3
C(5)-C(6)-C(1)	121.35(17)	C(5)-C(6)-H(6)	119.3
C(3)-C(7)-H(7A)	109.5	C(3)-C(7)-H(7B)	109.5
C(3)-C(7)-H(7C)	109.5	H(7A)-C(7)-H(7B)	109.5
H(7A)-C(7)-H(7C)	109.5	H(7B)-C(7)-H(7C)	109.5
C(5)-C(8)-H(8A)	109.5	C(5)-C(8)-H(8B)	109.5
C(5)-C(8)-H(8C)	109.5	H(8A)-C(8)-H(8B)	109.5
H(8A)-C(8)-H(8C)	109.5	H(8B)-C(8)-H(8C)	109.5
N(1)-C(9)-C(10)	110.93(15)	N(1)-C(9)-C(11)	110.44(14)
N(1)-C(9)-C(12)	109.64(14)	C(10)-C(9)-C(11)	108.87(17)
C(12)-C(9)-C(10)	109.76(15)	C(12)-C(9)-C(11)	107.13(16)
C(9)-C(10)-H(10A)	109.5	C(9)-C(10)-H(10B)	109.5
C(9)-C(10)-H(10C)	109.5	H(10A)-C(10)-H(10B)	109.5
H(10A)-C(10)-H(10C)	109.5	H(10B)-C(10)-H(10C)	109.5

C(9)-C(11)-H(11A)	109.5	C(9)-C(11)-H(11B)	109.5
C(9)-C(11)-H(11C)	109.5	H(11A)-C(11)-H(11B)	109.5
H(11A)-C(11)-H(11C)	109.5	H(11B)-C(11)-H(11C)	109.5
C(9)-C(12)-H(12A)	109.5	C(9)-C(12)-H(12B)	109.5
C(9)-C(12)-H(12C)	109.5	H(12A)-C(12)-H(12B)	109.5
H(12A)-C(12)-H(12C)	109.5	H(12B)-C(12)-H(12C)	109.5
C(14)-C(13)-N(2)	121.37(15)	C(18)-C(13)-N(2)	119.99(15)
C(18)-C(13)-C(14)	118.62(15)	C(13)-C(14)-H(14)	119.7
C(15)-C(14)-C(13)	120.70(17)	C(15)-C(14)-H(14)	119.7
C(14)-C(15)-C(19)	120.88(18)	C(16)-C(15)-C(14)	118.92(17)
C(16)-C(15)-C(19)	120.20(17)	C(15)-C(16)-H(16)	119.2
C(15)-C(16)-C(17)	121.66(16)	C(17)-C(16)-H(16)	119.2
C(16)-C(17)-C(20)	120.82(17)	C(18)-C(17)-C(16)	118.46(17)
C(18)-C(17)-C(20)	120.71(18)	C(13)-C(18)-H(18)	119.2
C(17)-C(18)-C(13)	121.61(17)	C(17)-C(18)-H(18)	119.2
C(15)-C(19)-H(19A)	109.5	C(15)-C(19)-H(19B)	109.5
C(15)-C(19)-H(19C)	109.5	H(19A)-C(19)-H(19B)	109.5
H(19A)-C(19)-H(19C)	109.5	H(19B)-C(19)-H(19C)	109.5
C(17)-C(20)-H(20A)	109.5	C(17)-C(20)-H(20B)	109.5
C(17)-C(20)-H(20C)	109.5	H(20A)-C(20)-H(20B)	109.5
H(20A)-C(20)-H(20C)	109.5	H(20B)-C(20)-H(20C)	109.5
N(2)-C(21)-C(22)	109.07(14)	N(2)-C(21)-C(23)	109.52(14)
N(2)-C(21)-C(24)	112.21(15)	C(22)-C(21)-C(23)	108.63(15)
C(22)-C(21)-C(24)	108.74(15)	C(24)-C(21)-C(23)	108.61(15)
C(21)-C(22)-H(22A)	109.5	C(21)-C(22)-H(22B)	109.5
C(21)-C(22)-H(22C)	109.5	H(22A)-C(22)-H(22B)	109.5
H(22A)-C(22)-H(22C)	109.5	H(22B)-C(22)-H(22C)	109.5
C(21)-C(23)-H(23A)	109.5	C(21)-C(23)-H(23B)	109.5
C(21)-C(23)-H(23C)	109.5	H(23A)-C(23)-H(23B)	109.5
H(23A)-C(23)-H(23C)	109.5	H(23B)-C(23)-H(23C)	109.5
C(21)-C(24)-H(24A)	109.5	C(21)-C(24)-H(24B)	109.5
C(21)-C(24)-H(24C)	109.5	H(24A)-C(24)-H(24B)	109.5
H(24A)-C(24)-H(24C)	109.5	H(24B)-C(24)-H(24C)	109.5
C(26)-C(25)-N(3)	122.12(15)	C(26)-C(25)-C(30)	118.55(15)
C(30)-C(25)-N(3)	119.30(14)	C(25)-C(26)-H(26)	119.7
C(27)-C(26)-C(25)	120.60(16)	C(27)-C(26)-H(26)	119.7

C(26)-C(27)-C(31)	121.03(17)	C(28)-C(27)-C(26)	119.25(16)
C(28)-C(27)-C(31)	119.70(16)	C(27)-C(28)-H(28)	119.3
C(29)-C(28)-C(27)	121.50(16)	C(29)-C(28)-H(28)	119.3
C(28)-C(29)-C(30)	118.44(16)	C(28)-C(29)-C(32)	120.60(17)
C(30)-C(29)-C(32)	120.95(17)	C(25)-C(30)-H(30)	119.2
C(29)-C(30)-C(25)	121.63(16)	C(29)-C(30)-H(30)	119.2
C(27)-C(31)-H(31A)	109.5	C(27)-C(31)-H(31B)	109.5
C(27)-C(31)-H(31C)	109.5	H(31A)-C(31)-H(31B)	109.5
H(31A)-C(31)-H(31C)	109.5	H(31B)-C(31)-H(31C)	109.5
C(29)-C(32)-H(32A)	109.5	C(29)-C(32)-H(32B)	109.5
C(29)-C(32)-H(32C)	109.5	H(32A)-C(32)-H(32B)	109.5
H(32A)-C(32)-H(32C)	109.5	H(32B)-C(32)-H(32C)	109.5
N(3)-C(33)-C(34)	109.50(13)	N(3)-C(33)-C(35)	110.95(13)
N(3)-C(33)-C(36)	110.42(13)	C(34)-C(33)-C(35)	109.49(14)
C(34)-C(33)-C(36)	107.66(14)	C(36)-C(33)-C(35)	108.75(14)
C(33)-C(34)-H(34A)	109.5	C(33)-C(34)-H(34B)	109.5
C(33)-C(34)-H(34C)	109.5	H(34A)-C(34)-H(34B)	109.5
H(34A)-C(34)-H(34C)	109.5	H(34B)-C(34)-H(34C)	109.5
C(33)-C(35)-H(35A)	109.5	C(33)-C(35)-H(35B)	109.5
C(33)-C(35)-H(35C)	109.5	H(35A)-C(35)-H(35B)	109.5
H(35A)-C(35)-H(35C)	109.5	H(35B)-C(35)-H(35C)	109.5
C(33)-C(36)-H(36A)	109.5	C(33)-C(36)-H(36B)	109.5
C(33)-C(36)-H(36C)	109.5	H(36A)-C(36)-H(36B)	109.5
H(36A)-C(36)-H(36C)	109.5	H(36B)-C(36)-H(36C)	109.5
O(1)-C(37)-C(38)	107.29(14)	O(1)-C(37)-C(39)	110.93(15)
O(1)-C(37)-C(40)	108.63(16)	C(39)-C(37)-C(38)	109.77(18)
C(40)-C(37)-C(38)	109.16(17)	C(40)-C(37)-C(39)	110.97(17)
C(37)-C(38)-H(38A)	109.5	C(37)-C(38)-H(38B)	109.5
C(37)-C(38)-H(38C)	109.5	H(38A)-C(38)-H(38B)	109.5
H(38A)-C(38)-H(38C)	109.5	H(38B)-C(38)-H(38C)	109.5
C(37)-C(39)-H(39A)	109.5	C(37)-C(39)-H(39B)	109.5
C(37)-C(39)-H(39C)	109.5	H(39A)-C(39)-H(39B)	109.5
H(39A)-C(39)-H(39C)	109.5	H(39B)-C(39)-H(39C)	109.5
C(37)-C(40)-H(40A)	109.5	C(37)-C(40)-H(40B)	109.5
C(37)-C(40)-H(40C)	109.5	H(40A)-C(40)-H(40B)	109.5
H(40A)-C(40)-H(40C)	109.5	H(40B)-C(40)-H(40C)	109.5

O(2)-C(41)-C(42)	111.43(15)	O(2)-C(41)-C(43)	108.96(14)
O(2)-C(41)-C(44)	106.63(14)	C(42)-C(41)-C(43)	110.08(16)
C(42)-C(41)-C(44)	109.63(16)	C(43)-C(41)-C(44)	110.05(16)
C(41)-C(42)-H(42A)	109.5	C(41)-C(42)-H(42B)	109.5
C(41)-C(42)-H(42C)	109.5	H(42A)-C(42)-H(42B)	109.5
H(42A)-C(42)-H(42C)	109.5	H(42B)-C(42)-H(42C)	109.5
C(41)-C(43)-H(43A)	109.5	C(41)-C(43)-H(43B)	109.5
C(41)-C(43)-H(43C)	109.5	H(43A)-C(43)-H(43B)	109.5
H(43A)-C(43)-H(43C)	109.5	H(43B)-C(43)-H(43C)	109.5
C(41)-C(44)-H(44A)	109.5	C(41)-C(44)-H(44B)	109.5
C(41)-C(44)-H(44C)	109.5	H(44A)-C(44)-H(44B)	109.5
H(44A)-C(44)-H(44C)	109.5	H(44B)-C(44)-H(44C)	109.5
O(3)-C(45)-C(46)	106.93(16)	O(3)-C(45)-C(47)	111.05(17)
O(3)-C(45)-C(48)	108.85(15)	C(46)-C(45)-C(48)	108.99(18)
C(47)-C(45)-C(46)	110.02(19)	C(47)-C(45)-C(48)	110.89(17)
C(45)-C(46)-H(46A)	109.5	C(45)-C(46)-H(46B)	109.5
C(45)-C(46)-H(46C)	109.5	H(46A)-C(46)-H(46B)	109.5
H(46A)-C(46)-H(46C)	109.5	H(46B)-C(46)-H(46C)	109.5
C(45)-C(47)-H(47A)	109.5	C(45)-C(47)-H(47B)	109.5
C(45)-C(47)-H(47C)	109.5	H(47A)-C(47)-H(47B)	109.5
H(47A)-C(47)-H(47C)	109.5	H(47B)-C(47)-H(47C)	109.5
C(45)-C(48)-H(48A)	109.5	C(45)-C(48)-H(48B)	109.5
C(45)-C(48)-H(48C)	109.5	H(48A)-C(48)-H(48B)	109.5
H(48A)-C(48)-H(48C)	109.5	H(48B)-C(48)-H(48C)	109.5
O(4)-Si(1)-C(49)	110.03(13)	O(4)-Si(1)-C(50)	108.07(10)
O(4)-Si(1)-C(51)	109.44(12)	C(50)-Si(1)-C(49)	109.26(13)
C(50)-Si(1)-C(51)	109.79(14)	C(51)-Si(1)-C(49)	110.23(14)
O(4)-Si(2)-C(52C)	112.1(9)	O(4)-Si(2)-C(52A)	105.9(3)
O(4)-Si(2)-C(52B)	116.3(8)	O(4)-Si(2)-C(54C)	102.6(6)
O(4)-Si(2)-C(53A)	110.7(2)	O(4)-Si(2)-C(53B)	106.4(5)
O(4)-Si(2)-C(53C)	100.3(9)	O(4)-Si(2)-C(54A)	112.5(2)
O(4)-Si(2)-C(54B)	107.6(6)	C(52C)-Si(2)-C(54C)	107.7(11)
C(52C)-Si(2)-C(53C)	121.2(13)	C(52B)-Si(2)-C(53B)	113.7(10)
C(52B)-Si(2)-C(54B)	103.0(12)	C(53A)-Si(2)-C(52A)	104.4(4)
C(53B)-Si(2)-C(54B)	109.4(7)	C(53C)-Si(2)-C(54C)	111.3(12)
C(54A)-Si(2)-C(52A)	113.7(4)	C(54A)-Si(2)-C(53A)	109.3(4)

Si(2)-O(4)-Si(1)	145.04(12)	Si(1)-C(49)-H(49A)	109.5
Si(1)-C(49)-H(49B)	109.5	Si(1)-C(49)-H(49C)	109.5
H(49A)-C(49)-H(49B)	109.5	H(49A)-C(49)-H(49C)	109.5
H(49B)-C(49)-H(49C)	109.5	Si(1)-C(50)-H(50A)	109.5
Si(1)-C(50)-H(50B)	109.5	Si(1)-C(50)-H(50C)	109.5
H(50A)-C(50)-H(50B)	109.5	H(50A)-C(50)-H(50C)	109.5
H(50B)-C(50)-H(50C)	109.5	Si(1)-C(51)-H(51A)	109.5
Si(1)-C(51)-H(51B)	109.5	Si(1)-C(51)-H(51C)	109.5
H(51A)-C(51)-H(51B)	109.5	H(51A)-C(51)-H(51C)	109.5
H(51B)-C(51)-H(51C)	109.5	Si(2)-C(52C)-H(52A)	109.5
Si(2)-C(52C)-H(52B)	109.5	Si(2)-C(52C)-H(52C)	109.5
H(52A)-C(52C)-H(52B)	109.5	H(52A)-C(52C)-H(52C)	109.5
H(52B)-C(52C)-H(52C)	109.5	Si(2)-C(52A)-H(52D)	109.5
Si(2)-C(52A)-H(52E)	109.5	Si(2)-C(52A)-H(52F)	109.5
H(52D)-C(52A)-H(52E)	109.5	H(52D)-C(52A)-H(52F)	109.5
H(52E)-C(52A)-H(52F)	109.5	Si(2)-C(52B)-H(52G)	109.5
Si(2)-C(52B)-H(52H)	109.5	Si(2)-C(52B)-H(52I)	109.5
H(52G)-C(52B)-H(52H)	109.5	H(52G)-C(52B)-H(52I)	109.5
H(52H)-C(52B)-H(52I)	109.5	Si(2)-C(54C)-H(54A)	109.5
Si(2)-C(54C)-H(54B)	109.5	Si(2)-C(54C)-H(54C)	109.5
H(54A)-C(54C)-H(54B)	109.5	H(54A)-C(54C)-H(54C)	109.5
H(54B)-C(54C)-H(54C)	109.5	Si(2)-C(53A)-H(53A)	109.5
Si(2)-C(53A)-H(53B)	109.5	Si(2)-C(53A)-H(53C)	109.5
H(53A)-C(53A)-H(53B)	109.5	H(53A)-C(53A)-H(53C)	109.5
H(53B)-C(53A)-H(53C)	109.5	Si(2)-C(53B)-H(53D)	109.5
Si(2)-C(53B)-H(53E)	109.5	Si(2)-C(53B)-H(53F)	109.5
H(53D)-C(53B)-H(53E)	109.5	H(53D)-C(53B)-H(53F)	109.5
H(53E)-C(53B)-H(53F)	109.5	Si(2)-C(53C)-H(53G)	109.5
Si(2)-C(53C)-H(53H)	109.5	Si(2)-C(53C)-H(53I)	109.5
H(53G)-C(53C)-H(53H)	109.5	H(53G)-C(53C)-H(53I)	109.5
H(53H)-C(53C)-H(53I)	109.5	Si(2)-C(54A)-H(54D)	109.5
Si(2)-C(54A)-H(54E)	109.5	Si(2)-C(54A)-H(54F)	109.5
H(54D)-C(54A)-H(54E)	109.5	H(54D)-C(54A)-H(54F)	109.5
H(54E)-C(54A)-H(54F)	109.5	Si(2)-C(54B)-H(54G)	109.5
Si(2)-C(54B)-H(54H)	109.5	Si(2)-C(54B)-H(54I)	109.5
H(54G)-C(54B)-H(54H)	109.5	H(54G)-C(54B)-H(54I)	109.5

H(54H)-C(54B)-H(54I)	109.5

Plot of the Mo≡Mo Bond Distances Determined by X-Ray Diffraction Analysis

Complexes with a non-bridged Mo≡Mo core and a coordination number CN = 4 on both metal atoms were searched for. The Mo–Mo bond distance was selected as the 3D parameter for comparison. In addition, the options ‘any bond’ and ‘acyclic’ were selected. A database search (CSD version 5.45, March 2024) for related structures was performed on 20 January 2025. The following search motif was used in the ConQuest program (Version 2023.2.0)

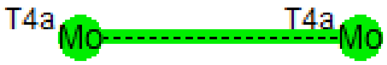

To extend the search to current daily structures, WebCSD (Chemical Structure Search: https://www.ccdc.cam.ac.uk/structures/WebCSD/StructureSearch) was performed on 20 January 2025. This search did not yield any additional structures.

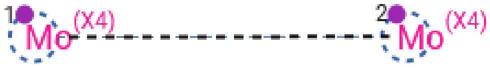

The initial search yielded 58 structures containing the predefined Mo subunit. In some cases, the structures had more than one molecule per asymmetric unit; these data are also listed, thus leading to a total of 67 data points.

Table S14. Complexes comprising an unbridged Mo≡Mo core and a coordination number CN = 4 on either metal atom found in the Cambridge Crystallograpic Data Centre (search on 20 January 2025) ordered according to increasing Mo–Mo distances; the structures are outlined below.

**Number**	**CCDC Refcode**	**Mo ··· Mo distance / Å**
1	TMSIMO01	2.157
2	TMSIMO01	2.164
3	GUGWEN	2.165
4	TMSIMO01	2.170
5	WOFLOS	2.170
6	GEPKIV	2.175
7	TMSIMO01	2.175
8	WOFLIM	2.176
9	WOFLIM	2.177
10	QEHYEH	2.19
11	QEHYEH	2.191
12	BUBTUN	2.196
13	BOFBON	2.200
14	BOFBON10	2.200
15	DMACMO	2.200
16	BOZXAP	2.201
17	BOZXAP10	2.201
18	DMACMO	2.202
19	MANMOA	2.203
20	PYMTMP	2.204
21	TIQMIP	2.206
22	BELYUM	2.209
23	BERZOO	2.210
24	BESBAD	2.211
25	HXMAMO10	2.211
26	LEFLEN	2.211
27	FEFCIC	2.214
28	CUKTOR	2.216
29 +	FUBKUI	2.216
30	SOMXEX	2.216
31	BOYCIB	2.217
32	DAKKAB	2.217
33	HXMAMO10	2.217
34	VERLEJ	2.217
35	VERLEJ01	2.217
36	CALVOA	2.218
37	VERKOS	2.218

38	VERKOS01	2.218
39	ZORMIC	2.219
40	VERLEJ02	2.220
41	NEPXMO10	2.221
42	OTUTUT	2.222
43	OTUVEF	2.222
44	OTUVEF	2.222
45	OTUTUT	2.223
46	BUBVAV	2.226
47	WEJCIA	2.226
48	JOGSUT	2.227
49	CANDEA	2.228
50	CANDEA01	2.228
51	CIRDEM	2.228
52	OWIRAQ	2.228
53	SONYOM	2.229
54	CIGNAH	2.203
55	KULVOC	2.230
56	LEHBAB	2.238
57	OTUVAB	2.239
58	DESJEQ	2.240
59	CAWHOX	2.241
60	OTUVAB	2.242
61	IRALIZ	2.243
62	OTUVIJ	2.246
63	BERZUU	2.247
64	DEVFIW	2.247
65	CAWHOX	2.249
66	EXEMID	2.258
67	IDOQAT	2.271
68	complex **13**	2.2873
69	complex **16**	2.2955
70	complex **17**	2.2944
71	complex **14**	2.3440

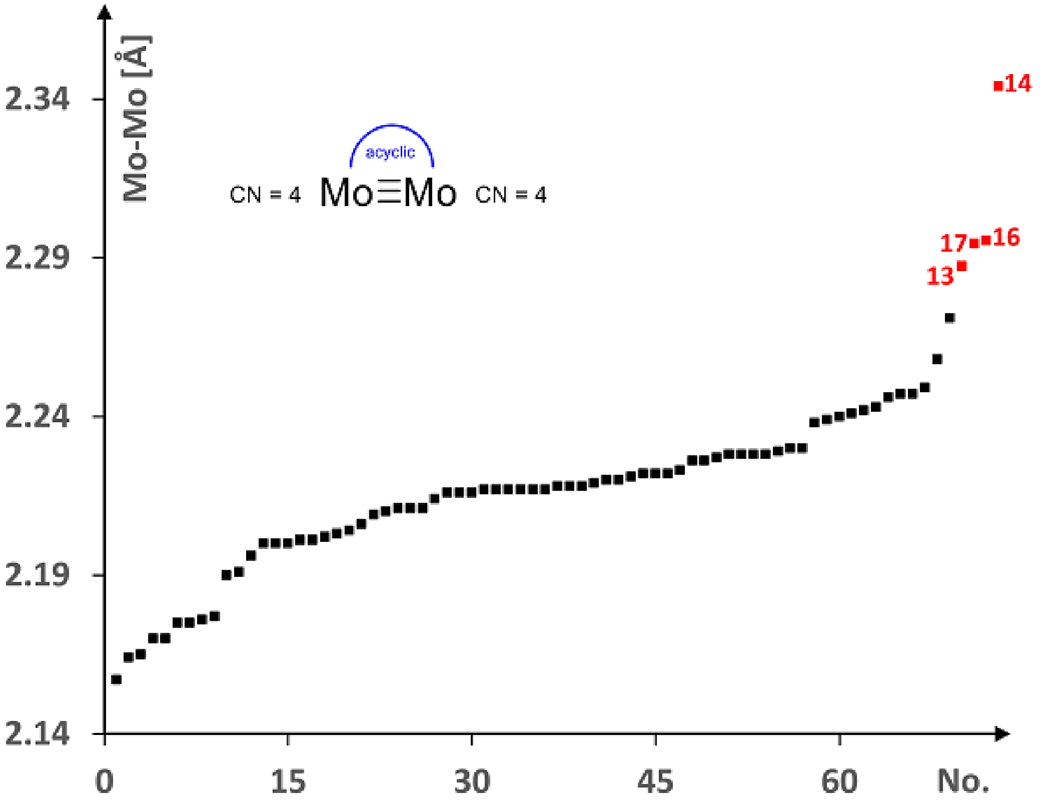

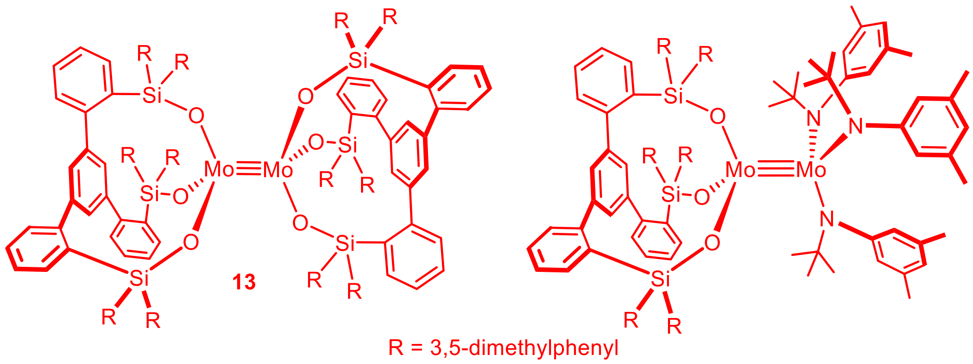

13 (d(Mo≡Mo) 2.2873(3) Å) **14** (d(Mo≡Mo) 2.3440(3) Å)

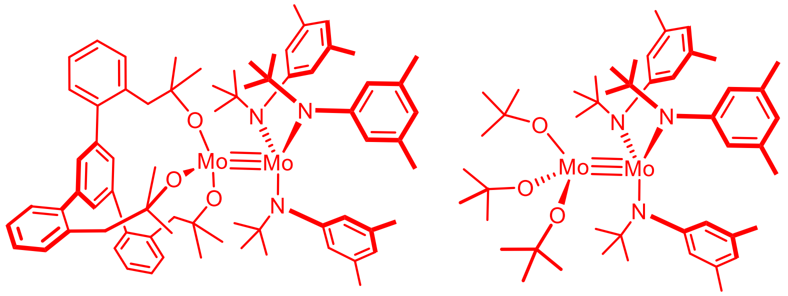

16 (d(Mo≡Mo) 2.2954(6) Å) **17** (d(Mo≡Mo) 2.2944(2) Å)

Search: search8 (Mon Jan 27 10:55:10 2025): Hits 1-4

BELYUM

Reference: M.H.Chisholm, K.Folting, J.C.Huffman, I.P.Rothwell (1982) *Organometallics* ,**1**,251

Formula: C_{24} H_{62} Mo_2 O_2 Si_4

Compound Name: bis(t-Butoxy)-tetrakis(trimethylsilylmethyl)-di-molybdenum

Space Group: P21/n	**Cell:**	***a*** 10.025(3)	***b*** 18.473(9)	***c*** 9.975(5)
Space Group No.: 14	**(Å,°)**	α 90.00	β 102.03(3)	γ 90.00

R-Factor (%): 8.30 ***Temperature(K):*** 110 ***Density(g/cm³):*** 1.263

Parameters

Fragment 1
DIST1 (D) 2.209

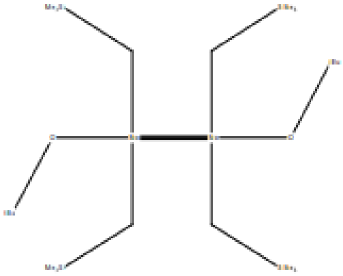

BERZOO

Reference: K.L.Fujdala, T.D.Tilley (2004) *Chem.Mater.* ,**16**,1035

Formula: C_{32} H_{78} Mo_2 N_4 O_8 Si_2

Compound Name: tetrakis(Dimethylamido)-bis(tris(t-butoxy)siloxy)-di-molybdenum(iii)

Space Group: C2/c	**Cell:**	***a*** 9.565(0)	***b*** 16.175(0)	***c*** 29.761(0)
Space Group No.: 15	**(Å,°)**	α 90.00	β 90.42(0)	γ 90.00

R-Factor (%): 3.52 ***Temperature(K):*** 151 ***Density(g/cm³):*** 1.291

Parameters

Fragment 1
DIST1 (D) 2.210

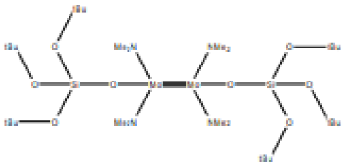

BERZUU

Reference: K.L.Fujdala, T.D.Tilley (2004) *Chem.Mater.* ,**16**,1035

Formula: C_{40} H_{90} Mo_2 O_{12} Si_2

Compound Name: tetrakis(t-Butoxy)-bis(tris(t-butoxy)siloxy)-di-molybdenum(iii)

Space Group: P21/n	**Cell:**	***a*** 12.773(0)	***b*** 12.417(0)	***c*** 17.386(0)
Space Group No.: 14	**(Å,°)**	α 90.00	β 102.30	γ 90.00

R-Factor (%): 3.33 ***Temperature(K):*** 154 ***Density(g/cm³):*** 1.246

Parameters

Fragment 1
DIST1 (D) 2.247

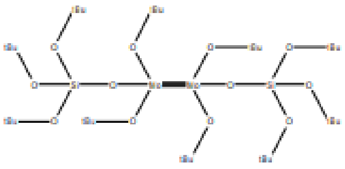

BESBAD

Reference: K.L.Fujdala, T.D.Tilley (2004) *Chem.Mater.* ,**16**,1035

Formula: C_{56} H_{132} B_2 Mo_2 N_4 O_{18} Si_4

Compound Name: tetrakis(Dimethylamido)-bis(bis(tris(t-butoxy)siloxy)boryloxido)-di-molybdenum(iii)

Space Group: P21/c	**Cell:**	***a*** 15.609(0)	***b*** 13.306(0)	***c*** 19.450(0)
Space Group No.: 14	**(Å,°)**	α 90.00	β 93.72(0)	γ 90.00

R-Factor (%): 3.81 ***Temperature(K):*** 145 ***Density(g/cm³):*** 1.216

Parameters

Fragment 1
DIST1 (D) 2.211

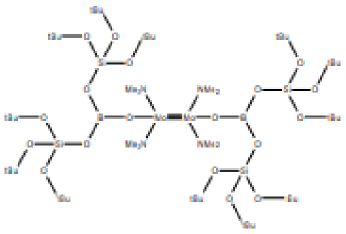

Page 2

Search: search8 (Mon Jan 27 10:55:10 2025): Hits 5-8

BOFBON

Reference: M.J.Chetcuti, M.H.Chisholm, K.Folting, J.C.Huffman, J.Janos (1982) *J.Am.Chem.Soc.* ,**104**,4684

Formula: C_{22} H_{38} Mo_2 N_4

Compound Name: Dibenzyl-tetrakis(dimethylamido)-di-molybdenum

Space Group:	P21/a	***Cell:***	***a*** 17.595(7)	***b*** 16.038(6)	***c*** 10.542(4)
Space Group No.:	14	***(Å,°)***	α 90.00	β 122.11(2)	γ 90.00
R-Factor (%):	3.60	***Temperature(K):*** 108	***Density(g/cm³):*** 1.451		

Parameters

Fragment 1

DIST1 (D) 2.200

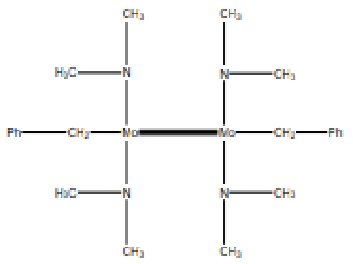

BOFBON10

Reference: M.J.Chetcuti, M.H.Chisholm, K.Folting, D.A.Haitko, J.C.Huffman, J.Janos (1983) *J.Am.Chem.Soc.* ,**105**,1163

Formula: C_{22} H_{38} Mo_2 N_4

Compound Name: 1,2-Dibenzyl-tetrakis(dimethylamido)-di-molybdenum

Space Group:	P21/a	***Cell:***	***a*** 17.595(7)	***b*** 16.038(6)	***c*** 10.542(4)
Space Group No.:	14	***(Å,°)***	α 90.00	β 122.11(2)	γ 90.00
R-Factor (%):	3.60	***Temperature(K):*** 108	***Density(g/cm³):*** 1.451		

Parameters

Fragment 1

DIST1 (D) 2.200

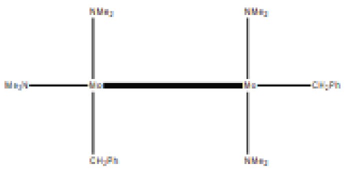

BOYCIB

Reference: M.H.Chisholm, J.F.Corning, J.C.Huffman (1983) *Inorg.Chem.* ,**22**,38

Formula: C_{16} H_{42} Mo_2 N_4 S_2

Compound Name: bis(t-Butylthio)-tetrakis(dimethylamino)-di-molybdenum

Space Group:	P21/n	***Cell:***	***a*** 14.322(4)	***b*** 17.514(5)	***c*** 9.865(2)
Space Group No.:	14	***(Å,°)***	α 90.00	β 101.39(1)	γ 90.00
R-Factor (%):	3.30	***Temperature(K):*** 113	***Density(g/cm³):*** 1.497		

Parameters

Fragment 1

DIST1 (D) 2.217

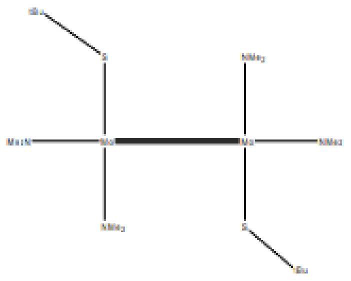

BOZXAP

Reference: M.J.Chetcuti, M.H.Chisholm, H.T.Chiu, J.C.Huffman (1983) *J.Am.Chem.Soc.* ,**105**,1060

Formula: C_{26} H_{78} Mo_2 N_4 Sn_8,2(C_7 H_8)

Compound Name: bis(tris(Trimethylstannyl)-tin)-tetrakis(dimethylamino)-di-molybdenum toluene solvate

Space Group:	Pbn21	***Cell:***	***a*** 14.600(4)	***b*** 31.737(13)	***c*** 13.545(4)
Space Group No.:	33	***(Å,°)***	α 90.00	β 90.00	γ 90.00
R-Factor (%):	4.60	***Temperature(K):*** 111	***Density(g/cm³):*** 1.876		

Parameters

Fragment 1

DIST1 (D) 2.201

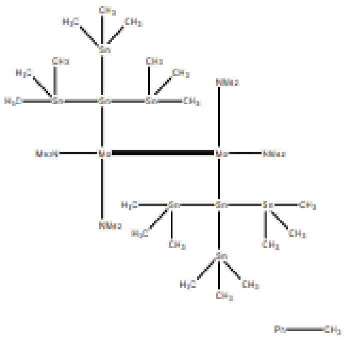

Search: search8 (Mon Jan 27 10:55:10 2025): Hits 9-12

BOZXAP10

Reference: M.H.Chisholm, H.-T.Chiu, K.Folting, J.C.Huffman (1984) *Inorg.Chem.* ,**23**,4097

Formula: C_{26} H_{78} Mo_2 N_4 Sn_8,2(C_7 H_8)

Compound Name: tetrakis(Dimethylamino)-bis(tris(trimethyl-tin)-tin)-di-molybdenum toluene solvate

Space Group: Pbn21 ***Cell:*** ***a*** 14.600(4) ***b*** 31.737(13) ***c*** 13.545(4)
Space Group No.: 33 **(Å,°)** α 90.00 β 90.00 γ 90.00

R-Factor (%): 4.60 ***Temperature(K):*** 111 ***Density(g/cm^3):*** 1.876

Parameters
Fragment 1
DIST1 (D) 2.201

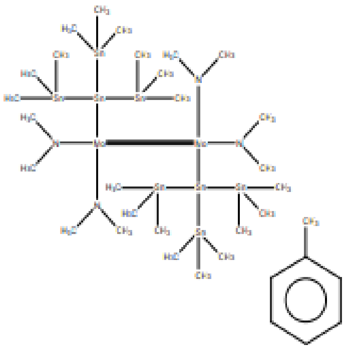

BUBTUN

Reference: M.J.Chetcuti, M.H.Chisholm, K.Folting, D.A.Haitko, J.C.Huffman, J.Janos (1983) *J.Am.Chem.Soc.* ,**105**,1163

Formula: C_{22} H_{38} Mo_2 N_4

Compound Name: 1,2-bis(p-Tolyl)-tetrakis(dimethylamido)-di-molybdenum

Space Group: Pcan ***Cell:*** ***a*** 8.048(2) ***b*** 17.319(7) ***c*** 18.179(8)
Space Group No.: 60 **(Å,°)** α 90.00 β 90.00 γ 90.00

R-Factor (%): 2.60 ***Temperature(K):*** 111 ***Density(g/cm^3):*** 1.443

Parameters
Fragment 1
DIST1 (D) 2.196

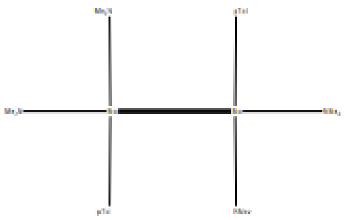

BUBVAV

Reference: M.J.Chetcuti, M.H.Chisholm, K.Folting, D.A.Haitko, J.C.Huffman, J.Janos (1983) *J.Am.Chem.Soc.* ,**105**,1163

Formula: C_{22} H_{38} Mo_2 N_4

Compound Name: 1,2-bis(o-Tolyl)-tetrakis(dimethylamido)-di-molybdenum

Space Group: A2/a ***Cell:*** ***a*** 16.845(4) ***b*** 17.651(5) ***c*** 8.451(2)
Space Group No.: 15 **(Å,°)** α 90.00 β 102.74(1) γ 90.00

R-Factor (%): 2.30 ***Temperature(K):*** 112 ***Density(g/cm^3):*** 1.492

Parameters
Fragment 1
DIST1 (D) 2.226

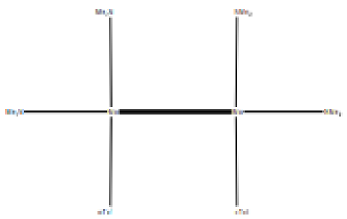

CALVOA

Reference: T.W.Coffindaffer, I.P.Rothwell, J.C.Huffman (1983) *Chem.Commun.* ,1249

Formula: C_{32} H_{62} Mo_2 O_2 Si_4

Compound Name: bis(2,6-Dimethylphenoxy)-tetrakis(trimethylsilylmethyl)-di-molybdenum

Space Group: P-1 ***Cell:*** ***a*** 12.091(6) ***b*** 10.839(5) ***c*** 10.384(5)
Space Group No.: 2 **(Å,°)** α 120.06(2) β 120.65(2) γ 74.96(3)

R-Factor (%): 4.10 ***Temperature(K):*** 104 ***Density(g/cm^3):*** 1.283

Parameters
Fragment 1
DIST1 (D) 2.218

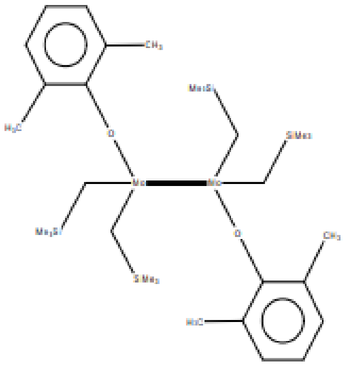

Page 4

Search: search8 (Mon Jan 27 10:55:10 2025): Hits 13-16

CANDEA

Reference: M.H.Chisholm, J.F.Corning, J.C.Huffman (1983)
J.Am.Chem.Soc. ,**105**,5924

Formula: $C_{54}\ H_{66}\ Mo_2\ S_6,C_6\ H_{14}$

Compound Name: hexakis(2,4,6-Trimethylbenzenethiolato)-di-molybdenum n-hexane solvate

Space Group:	R-3	***Cell:***	**a** 15.361(10)	**b** 15.361(10)	**c** 20.929		
Space Group No.:	148	***(Å,°)***	α 90.00	β 90.00	γ 120.00		
R-Factor (%):	2.90	***Temperature(K):***	111	***Density(g/cm³):***	1.381		

Parameters

Fragment 1
DIST1 (D) 2.228

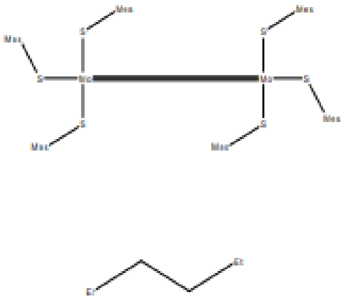

CANDEA01

Reference: M.H.Chisholm, J.F.Corning, K.Folting, J.C.Huffman (1985)
Polyhedron ,**4**,383

Formula: $C_{54}\ H_{66}\ Mo_2\ S_6,C_6\ H_{14}$

Compound Name: hexakis(Mesitylthiolato-S)-di-molybdenum hexane solvate

Space Group:	R-3	***Cell:***	**a** 15.361(10)	**b** 15.361(10)	**c** 20.929(13)		
Space Group No.:	148	***(Å,°)***	α 90.00	β 90.00	γ 120.00		
R-Factor (%):	2.95	***Temperature(K):***	111	***Density(g/cm³):***	1.381		

Parameters

Fragment 1
DIST1 (D) 2.228

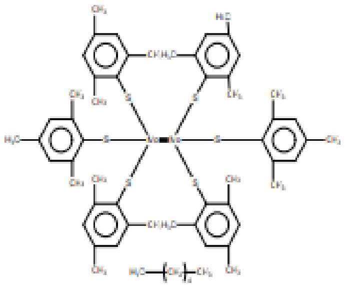

CAWHOX

Reference: T.W.Coffindaffer, I.P.Rothwell, J.C.Huffman (1983)
Inorg.Chem. ,**22**,2906

Formula: $C_{38}\ H_{50}\ Mo_2\ O_6$

Compound Name: 1,2-bis(Isopropoxy)-tetrakis(2,6-dimethylphenoxy)-di-molybdenum

Space Group:	P-1	***Cell:***	**a** 18.169(6)	**b** 11.327(3)	**c** 10.678(3)		
Space Group No.:	2	***(Å,°)***	α 99.58(2)	β 57.82(2)	γ 97.63(2)		
R-Factor (%):	4.06	***Temperature(K):***	110	***Density(g/cm³):***	1.441		

Parameters

Fragment 1
DIST1 (D) 2.241

Fragment 2
DIST1 (D) 2.249

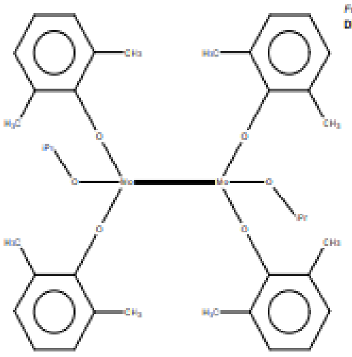

CIGNAH

Reference: M.H.Chisholm, J.F.Corning, J.C.Huffman (1984)
Inorg.Chem. ,**23**,754

Formula: $C_{42}\ H_{58}\ Mo_2\ O_2\ S_4$

Compound Name: bis(Isopropoxy-bis(mesitylenethiolato)-molybdenum)

Space Group:	C2/c	***Cell:***	**a** 21.778(2)	**b** 8.600(2)	**c** 24.587(2)		
Space Group No.:	15	***(Å,°)***	α 90.00	β 108.90(1)	γ 90.00		
R-Factor (%):	2.60	***Temperature(K):***	106	***Density(g/cm³):***	1.395		

Parameters

Fragment 1
DIST1 (D) 2.230

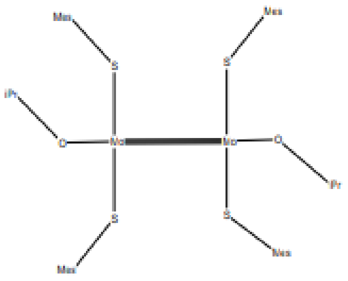

Search: search8 (Mon Jan 27 10:55:10 2025): Hits 17-20

CIRDEM

Reference: T.W.Coffindaffer, I.P.Rothwell, J.C.Huffman (1984) *Inorg.Chem.* **23**,1433

Formula: C_{36} H_{48} Mo_2 N_2 O_4

Compound Name: bis(Dimethylamino)-tetrakis(2,5-dimethylphenoxy)-di-molybdenum

**Space Group:**	P21/n	**Cell:**	**a** 16.618(3)	**b** 10.208(1)	**c** 10.369(2)
**Space Group No.:**	14	**(Å,°)**	α 90.00	β 95.25(1)	γ 90.00

R-Factor (%): 3.03 **Temperature(K):** 105 **Density(g/cm³):** 1.450

Parameters

Fragment 1

DIST1 (D) 2.228

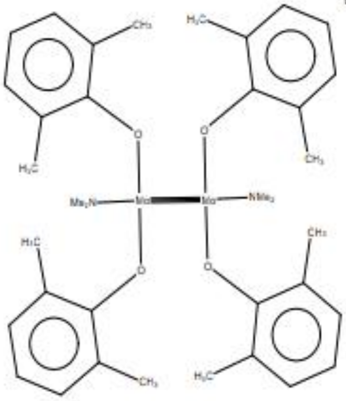

CUKTOR

Reference: M.H.Chisholm, H.-T.Chiu, K.Folting, J.C.Huffman (1984) *Inorg.Chem.* **23**,4097

Formula: C_{26} H_{78} Mo_2 N_4 Si_8

Compound Name: tetrakis(Dimethylamino)-bis(tris(trimethylsilyl)-allyl)-di-molybdenum

**Space Group:**	P-1	**Cell:**	**a** 9.400(3)	**b** 9.949(4)	**c** 13.304(6)
**Space Group No.:**	2	**(Å,°)**	α 83.24(3)	β 97.81(2)	γ 69.80(2)

R-Factor (%): 2.60 **Temperature(K):** 108 **Density(g/cm³):** 1.259

Parameters

Fragment 1

DIST1 (D) 2.216

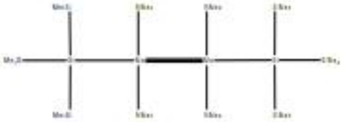

DAKKAB

Reference: T.W.Coffindaffer, I.P.Rothwell, J.C.Huffman (1985) *Inorg.Chem.* **24**,1643

Formula: C_{32} H_{54} Mo_2 N_4 O_2

Compound Name: 1,2-bis(2-t-Butyl-6-methylphenoxy)-tetrakis(dimethylamido)-di-molybdenum

**Space Group:**	P212121	**Cell:**	**a** 13.532(4)	**b** 16.634(6)	**c** 14.969(10)
**Space Group No.:**	19	**(Å,°)**	α 90.00	β 90.00	γ 90.00

R-Factor (%): 4.50 **Temperature(K):** 113 **Density(g/cm³):** 1.369

Parameters

Fragment 1

DIST1 (D) 2.217

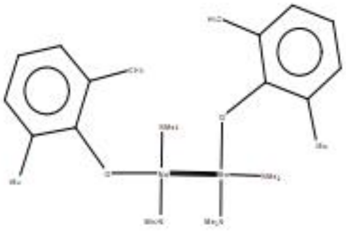

DESJEQ

Reference: P.J.Blower, J.R.Dilworth, J.Zubieta (1985) Inorg.Chem. , **24**,2866

Formula: C_{90} H_{138} Mo_2 S_6

Compound Name: hexakis(2,4,6-Tri-isopropylphenylthiolato-S)-di-molybdenum

**Space Group:**	Pbcn	**Cell:**	**a** 17.679(3)	**b** 21.019(4)	**c** 24.215(5)
**Space Group No.:**	60	**(Å,°)**	α 90.00	β 90.00	γ 90.00

R-Factor (%): 7.00 **Temperature(K):** 295 **Density(g/cm³):** 1.184

Parameters

Fragment 1

DIST1 (D) 2.240

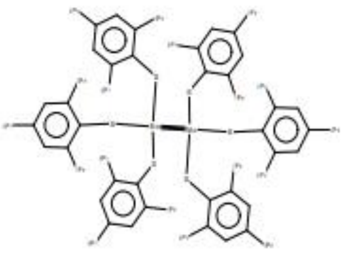

Search: search8 (Mon Jan 27 10:55:10 2025): Hits 21-24

DEVFIW

Reference: R.D.Rogers, T.Gilbert (2018)
CSD Communication(Private Communication) ,

Formula: C_{32} H_{64} Mo_2 N_2 O_4

Compound Name: tetrakis(1-methylcyclohexan-1-olato)-bis(dimethylamino)-di-molybdenum

**Space Group:**	P21/c	**Cell:**	**a**	10.678(0)	**b**	19.167(0)	**c**	9.163(0)
**Space Group No.:**	14	**(Å,°)**	α	90.00	β	107.88(0)	γ	90.00
**R-Factor (%):**	4.04	**Temperature(K):**	173		**Density(g/cm³):**	1.382		

Parameters

Fragment 1
DIST1 (D) 2.247

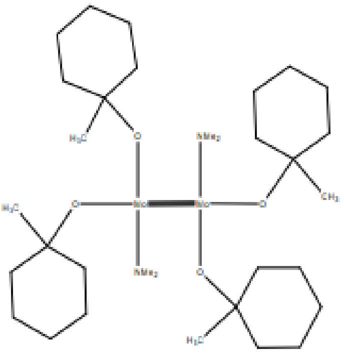

DMACMO

Reference: M.Akiyama, M.H.Chisholm, F.A.Cotton, M.W.Extine,
C.A.Murillo (1977) *Inorg.Chem.* ,**16**,2407

Formula: C_8 H_{24} Cl_2 Mo_2 N_4

Compound Name: Dichloro-tetrakis(dimethylamido) dimolybdenum

**Space Group:**	P21/c	**Cell:**	**a**	15.031(2)	**b**	13.248(1)	**c**	8.411(1)
**Space Group No.:**	14	**(Å,°)**	α	90.00	β	96.38(1)	γ	90.00
**R-Factor (%):**	4.60	**Temperature(K):**	295		**Density(g/cm³):**	1.752		

Parameters

Fragment 1
DIST1 (D) 2.200

Fragment 2
DIST1 (D) 2.202

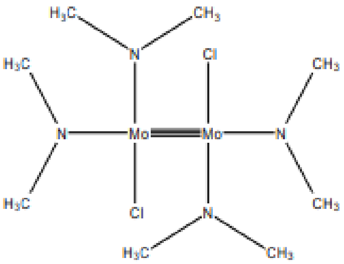

EXEMID

Reference: T.M.Gilbert, J.C.Littrell, C.E.Talley, M.A.Vance,
R.F.Dallinger, R.D.Rogers (2004) *Inorg.Chem.* ,**43**,1762

Formula: C_{78} H_{138} Mo_2 O_6

Compound Name: hexakis(4-Pentylbicyclo(2.2.2)octyloxy)-di-molybdenum

**Space Group:**	P21/c	**Cell:**	**a**	13.016(0)	**b**	10.887(0)	**c**	27.649(0)
**Space Group No.:**	14	**(Å,°)**	α	90.00	β	100.70(0)	γ	90.00
**R-Factor (%):**	4.70	**Temperature(K):**	296		**Density(g/cm³):**	1.176		

Parameters

Fragment 1
DIST1 (D) 2.258

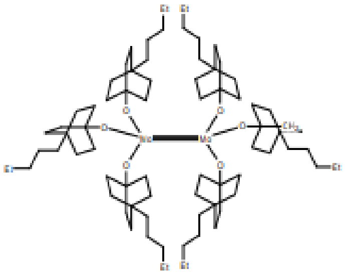

FEFCIC

Reference: W.E.Buhro, M.H.Chisholm, K.Folting, J.C.Huffman (1987)
J.Am.Chem.Soc. ,**109**,905

Formula: C_{24} H_{60} Mo_2 N_4 P_2

Compound Name: 1,2-bis(Di-t-butylphosphido)-tetrakis(dimethylimido)-di-molybdenum

**Space Group:**	P21/n	**Cell:**	**a**	9.763(1)	**b**	18.210(3)	**c**	10.031(1)
**Space Group No.:**	14	**(Å,°)**	α	90.00	β	117.64(1)	γ	90.00
**R-Factor (%):**	2.40	**Temperature(K):**	117		**Density(g/cm³):**	1.384		

Parameters

Fragment 1
DIST1 (D) 2.214

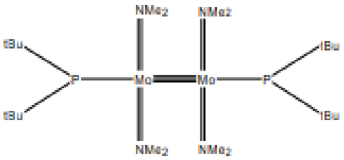

Search: search8 (Mon Jan 27 10:55:10 2025): Hits 25-28

FUBKUI

Reference:	M.H.Chisholm, J.C.Huffman, J.W.Pasterczyk (1987) *Inorg.Chem.* ,**26**,3781
Formula:	C_{24} H_{60} As_2 Mo_2 N_4
Compound Name:	bis(Di-t-butylarsenido)-tetrakis(dimethylamido)-di-molybdenum

Space Group:	P21/n	***Cell:***	***a*** 14.841(3)	***b*** 10.478(2)	***c*** 10.387(1)
Space Group No.:	14	***(Å,°)***	α 90.00	β 90.84(1)	γ 90.00
R-Factor (%):	3.95	***Temperature(K):*** 118	***Density(g/cm³):*** 1.538		

Parameters

Fragment 1
DIST1 (D) 2.216

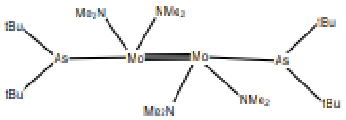

GEPKIV

Reference:	S.M.Beshouri, I.P.Rothwell, K.Folting, J.C.Huffman, W.E.Streib (1986) *Polyhedron* ,**5**,1191
Formula:	C_{42} H_{42} Mo_2
Compound Name:	hexabenzyl-di-molybdenum

Space Group:	R-3	***Cell:***	***a*** 14.682(6)	***b*** 14.682(6)	***c*** 13.182(5)
Space Group No.:	148	***(Å,°)***	α 90.00	β 90.00	γ 120.00
R-Factor (%):	1.88	***Temperature(K):*** 119	***Density(g/cm³):*** 1.495		

Parameters

Fragment 1
DIST1 (D) 2.175

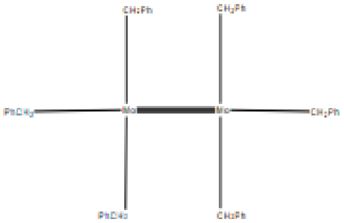

GUGWEN

Reference:	A.Chapovetsky, R.R.Langeslay, Gokhan Celik, F.A.Perras, M.Pruski, M.S.Ferrandon, E.C.Wegener, Hacksung Kim, Fulya Dogan, Jianguo Wen, N.Khetrapal, P.Sharma, J.White, A.Jeremy Kropf, A.P.Sattelberger, D.M.Kaphan, M.Delferro (2020) Organometallics ,**39**,1035
Formula:	C_{30} H_{66} Mo_2
Compound Name:	hexakis(2,2-dimethylpropyl)-di-molybdenum

Space Group:	R-3	***Cell:***	***a*** 10.018(0)	***b*** 10.018(0)	***c*** 29.388(0)
Space Group No.:	148	***(Å,°)***	α 90.00	β 90.00	γ 120.00
R-Factor (%):	3.87	***Temperature(K):*** 100	***Density(g/cm³):*** 1.207		

Parameters

Fragment 1
DIST1 (D) 2.165

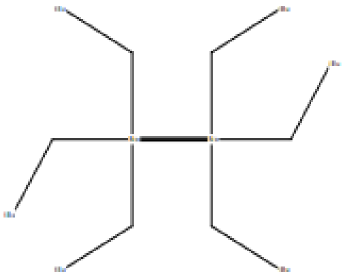

HXMAMO10

Reference:	M.H.Chisholm, F.A.Cotton, B.A.Frenz, W.W.Reichert, L.W.Shive, B.R.Stults (1976) *J.Am.Chem.Soc.* ,**98**,4469
Formula:	C_{12} H_{36} Mo_2 N_6
Compound Name:	hexakis(Dimethylamido) dimolybdenum

Space Group:	P21/c	***Cell:***	***a*** 11.461(3)	***b*** 12.052(4)	***c*** 15.409(3)
Space Group No.:	14	***(Å,°)***	α 90.00	β 101.83(2)	γ 90.00
R-Factor (%):	5.70	***Temperature(K):*** 295	***Density(g/cm³):*** 1.455		

Parameters

Fragment 1
DIST1 (D) 2.211

Fragment 2
DIST1 (D) 2.217

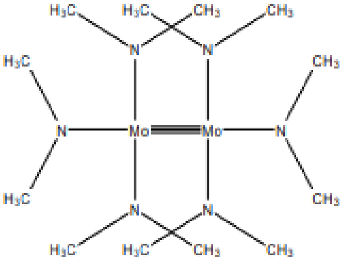

Search: search8 (Mon Jan 27 10:55:10 2025): Hits 29-32

IDOQAT

Reference: A.Furstner, C.Mathes, C.W.Lehmann (2001) *Chem.-Eur.J.* ,7,5299

Formula: $C_{48} H_{72} Cl_2 Mo_2 N_4$

Compound Name: bis(Chloro-bis(t-butyl(3,5-dimethylphenyl)amido)-molybdenum)

**Space Group:**	P21/n	**Cell:**	***a***	9.338(1)	***b***	19.863(4)	***c***	12.807(3)
**Space Group No.:**	14	**(Å,°)**	α	90.00	β	92.54(3)	γ	90.00
***R*-Factor (%):**	5.25	***Temperature(K):***	100		***Density(g/cm³):***	1.390		

Parameters

Fragment 1
DIST1 (D) 2.271

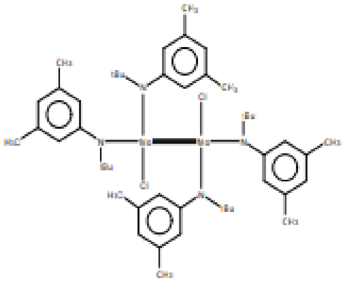

JOGSUT

Reference: M.H.Chisholm, I.P.Parkin, J.C.Huffman, E.M.Lobkovsky, K.Folting (1991) *Polyhedron* ,**10**,2839

Formula: $C_{46} H_{54} Mo_2 N_4 O_2$

Compound Name: anti-bis(bis(Dimethylamino)-(triphenylmethanol-O)-molybdenum)

**Space Group:**	P21/n	**Cell:**	***a***	9.528(2)	***b***	11.133(3)	***c***	20.602(5)
**Space Group No.:**	14	**(Å,°)**	α	90.00	β	103.24(1)	γ	90.00
***R*-Factor (%):**	4.82	***Temperature(K):***	102		***Density(g/cm³):***	1.385		

Parameters

Fragment 1
DIST1 (D) 2.227

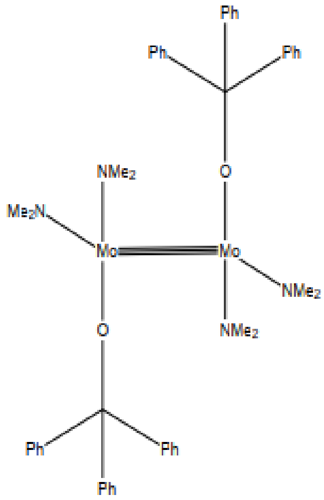

KULVOC

Reference: T.M.Gilbert, A.M.Landes, R.D.Rogers (1992) *Inorg.Chem.* , **31**,3438

Formula: $C_{24} H_{18} F_{36} Mo_2 O_6$

Compound Name: hexakis(1,1-bis(Trifluoromethyl)ethoxy)-di-molybdenum

**Space Group:**	R-3	**Cell:**	***a***	10.894(8)	***b***	10.894(8)	***c***	29.255(9)
**Space Group No.:**	148	**(Å,°)**	α	90.00	β	90.00	γ	120.00
***R*-Factor (%):**	7.10	***Temperature(K):***	253		***Density(g/cm³):***	2.118		

Parameters

Fragment 1
DIST1 (D) 2.230

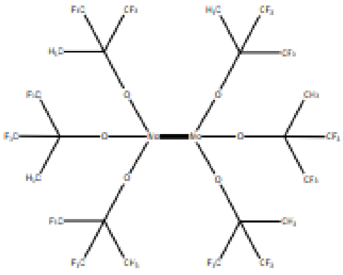

LEFLEN

Reference: H.Schulz, K.Folting, J.C.Huffman, W.E.Streib, M.H.Chisholm (1993) *Inorg.Chem.* ,**32**,6056

Formula: $0.9(C_{10} H_{30} I_1 Mo_2 N_5).0.1(C_8 H_{24} I_2 Mo_2 N_4)$

Compound Name: pentakis(Dimethylamido)-iodo-di-molybdenum tetrakis(dimethylamido)-di-iodo-di-molybdenum

**Space Group:**	Pmnb	**Cell:**	***a***	14.251(2)	***b***	18.031(2)	***c***	7.477(1)
**Space Group No.:**	62	**(Å,°)**	α	90.00	β	90.00	γ	90.00
***R*-Factor (%):**	3.46	***Temperature(K):***	95		***Density(g/cm³):***	1.893		

Parameters

Fragment 1
DIST1 (D) 2.211

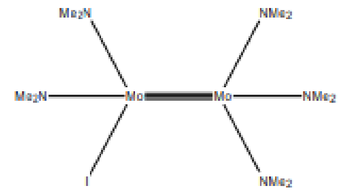

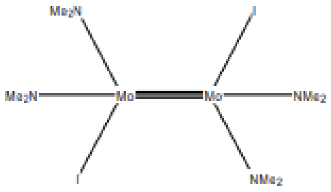

Page 9

Search: search8 (Mon Jan 27 10:55:10 2025): Hits 33-36

LEHBAB

Reference: T.M.Gilbert, C.B.Bauer, A.H.Bond, R.D.Rogers (1999) *Polyhedron* ,**18**,1293

Formula: $C_{54} H_{66} Mo_2 O_6$

Compound Name: hexakis(α,α-Dimethylbenzyloxy)-di-molybdenum

Space Group:	P21/n	***Cell:***	***a***	9.909(0)	***b***	24.173(0)	***c***	10.358(0)
Space Group No.:	14	***(Å,°)***	α	90.00	β	96.05(0)	γ	90.00

R-Factor (%): 3.28 ***Temperature(K):*** 173 ***Density(g/cm³):*** 1.350

Parameters

Fragment 1

DIST1 (D) 2.238

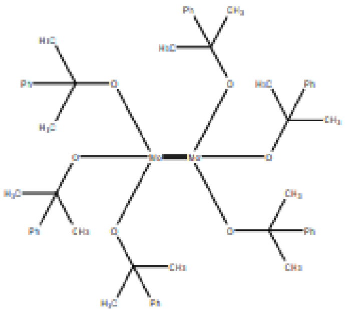

MANMOA

Reference: M.H.Chisholm, D.A.Haitko, K.Folting, J.C.Huffman (1981) *J.Am.Chem.Soc.* ,**103**,4046

Formula: $C_{12} H_{34} Mo_2 N_4$

Compound Name: Diethyl-tetrakis(N,N-dimethylamino)-di-molybdenum

Space Group:	P-1	***Cell:***	***a***	11.730(2)	***b***	8.402(1)	***c***	9.840(1)
Space Group No.:	2	***(Å,°)***	α	98.09(1)	β	98.43(1)	γ	82.74(1)

R-Factor (%): 3.20 ***Temperature(K):*** 101 ***Density(g/cm³):*** 1.499

Parameters

Fragment 1

DIST1 (D) 2.203

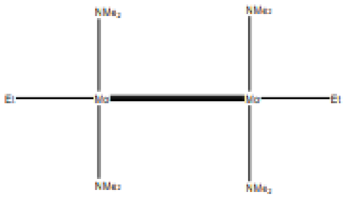

NEPXMO10

Reference: M.H.Chisholm, F.A.Cotton, C.A.Murillo, W.W.Reichert (1977) *Inorg.Chem.* ,**16**,1801

Formula: $C_{30} H_{66} Mo_2 O_6$

Compound Name: hexakis(2,2-Dimethylpropoxy)-di-molybdenum

Space Group:	P21/n	***Cell:***	***a***	18.160(10)	***b***	11.051(7)	***c***	9.956(6)
Space Group No.:	14	***(Å,°)***	α	90.00	β	104.30(4)	γ	90.00

R-Factor (%): 7.10 ***Temperature(K):*** 295 ***Density(g/cm³):*** 1.226

Parameters

Fragment 1

DIST1 (D) 2.221

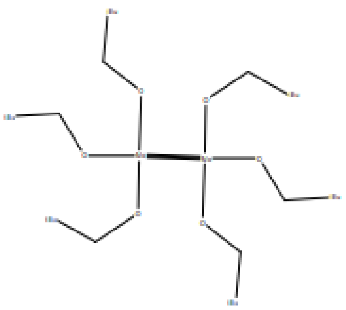

OTUTUT

Reference: S.Krackl, Jian-Gong Ma, Y.Aksu, M.Driess (2011) *Eur.J.Inorg.Chem.* ,1725

Formula: $C_{30} H_{62} Mo_2 O_6$

Compound Name: tetrakis(2,2-dimethylpropan-1-olato)-bis(2-methylbut-3-en-2-olato)-di-molybdenum

Space Group:	P-1	***Cell:***	***a***	11.874(0)	***b***	12.184(0)	***c***	15.658(0)
Space Group No.:	2	***(Å,°)***	α	75.57(0)	β	69.98(0)	γ	61.08(0)

R-Factor (%): 5.21 ***Temperature(K):*** 150 ***Density(g/cm³):*** 1.273

Parameters

Fragment 1

DIST1 (D) 2.222

Fragment 2

DIST1 (D) 2.223

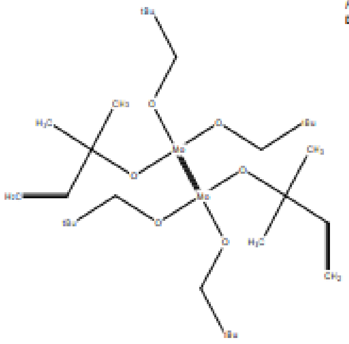

Page 10

Search: search8 (Mon Jan 27 10:55:10 2025): Hits 37-40

OTUVAB

Reference: S.Krackl, Jian-Gong Ma, Y.Aksu, M.Driess (2011)
Eur.J.Inorg.Chem. ,1725

Formula: C_{30} H_{66} Mo_2 O_8

Compound Name: tetrakis(2,2-dimethylpropan-1-olato)-bis(1-methoxy-2-methylpropan-2-olato)-di-molybdenum

Space Group:	P-1	***Cell:***	**a** 10.049(0)	**b** 11.077(0)	**c** 20.079(1)
Space Group No.:	2	***(Å,°)***	α 97.10(0)	β 99.64(0)	γ 114.84(0)
R-Factor (%):	7.18	***Temperature(K):*** 150	***Density(g/cm³):*** 1.270		

Parameters
Fragment 1
DIST1 (D) 2.239
Fragment 2
DIST1 (D) 2.242

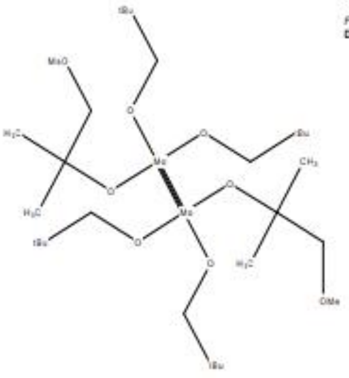

OTUVEF

Reference: S.Krackl, Jian-Gong Ma, Y.Aksu, M.Driess (2011)
Eur.J.Inorg.Chem. ,1725

Formula: C_{28} H_{62} Mo_2 O_6

Compound Name: tetrakis(2,2-dimethylpropan-1-olato)-bis(2-methylpropan-2-olato)-di-molybdenum

Space Group:	P-1	***Cell:***	**a** 11.930(0)	**b** 12.026(0)	**c** 15.027(0)
Space Group No.:	2	***(Å,°)***	α 78.30(0)	β 71.87(0)	γ 62.22(0)
R-Factor (%):	4.50	***Temperature(K):*** 150	***Density(g/cm³):*** 1.261		

Parameters
Fragment 1
DIST1 (D) 2.222
Fragment 2
DIST1 (D) 2.222

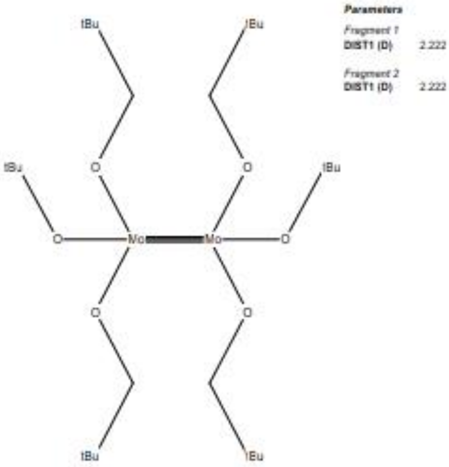

OTUVIJ

Reference: S.Krackl, Jian-Gong Ma, Y.Aksu, M.Driess (2011)
Eur.J.Inorg.Chem. ,1725

Formula: C_{50} H_{90} Mo_2 O_6

Compound Name: bis(2,2-dimethylpropan-1-olato)-tetrakis(2-(4-methylcyclohex-3-en-1-yl) propan-2-olato)-di-molybdenum

Space Group:	P21/c	***Cell:***	**a** 10.061(0)	**b** 20.087(0)	**c** 13.546(0)
Space Group No.:	14	***(Å,°)***	α 90.00	β 111.20(0)	γ 90.00
R-Factor (%):	5.53	***Temperature(K):*** 150	***Density(g/cm³):*** 1.274		

Parameters
Fragment 1
DIST1 (D) 2.246

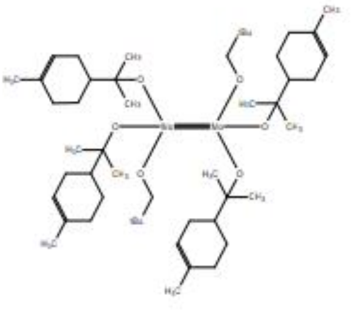

PAQSIJ

Reference: M.H.Chisholm, K.Folting, W.E.Streib, De-Dong Wu (1998)
Inorg.Chem. ,**37**,50

Formula: C_{54} H_{100} Mo_2 N_2 O_4,1.5(C_4 H_{10} O_1)

Compound Name: bis(2,2'-Ethylidenebis(4,6-di-t-butylphenolato))-bis(dimethylamino-molybdenum) diethyl ether solvate

Space Group:	P-1	***Cell:***	**a** 16.483(3)	**b** 16.817(3)	**c** 14.305(2)
Space Group No.:	2	***(Å,°)***	α 106.13(1)	β 108.80(1)	γ 71.70(1)
R-Factor (%):	5.03	***Temperature(K):*** 105	***Density(g/cm³):*** 1.202		

Parameters
Fragment 1
DIST1 (D) 2.220

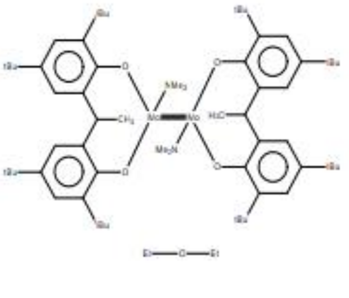

Search: search8 (Mon Jan 27 10:55:10 2025): Hits 41-44

PYMTMP

Reference: M.H.Chisholm, K.Folting, J.C.Huffman, I.P.Rothwell (1981) *Inorg.Chem.* ,**20**,1496

Formula: C_{22} H_{40} Mo_2 N_6

Compound Name: bis(6-Methyl-2-pyridyl)-methyl-tetrakis(dimethylamido)-di-molybdenum

Space Group: P21/a **Cell:** **a** 22.013(5) **b** 7.974(2) **c** 14.933(3)
Space Group No.: 14 **(Å,°)** α 90.00 β 96.43(1) γ 90.00

R-Factor (%): 5.60 **Temperature(K):** 102 **Density(g/cm³):** 1.480

Parameters

Fragment 1
DIST1 (D) 2.204

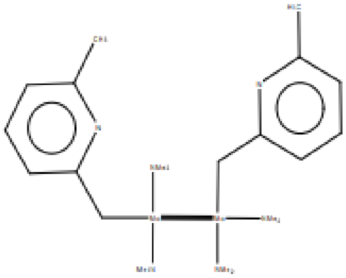

QEHYEH

Reference: M.H.Chisholm, D.R.Click, J.C.Huffman (2000) Organometallics ,**19**,3916

Formula: C_{30} H_{60} Mo_2 N_1 P_1 Si_4

Compound Name: (Dimethylamino)-(diphenylphosphino)-tetrakis(trimethylsilylmethyl)-dimolybdenum

Space Group: P-1 **Cell:** **a** 11.179(1) **b** 17.655(1) **c** 21.060(2)
Space Group No.: 2 **(Å,°)** α 81.23(0) β 75.85(0) γ 84.58(0)

R-Factor (%): 7.69 **Temperature(K):** 108 **Density(g/cm³):** 1.286

Parameters

Fragment 1
DIST1 (D) 2.190

Fragment 2
DIST1 (D) 2.191

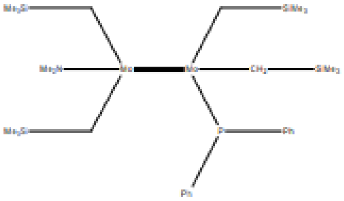

ROCJIC

Reference: M.H.Chisholm, Jui-Hsien Huang, J.C.Huffman, I.P.Parkin (1997) *Inorg.Chem.* ,**36**,1642

Formula: C_{50} H_{72} Mo_2 N_2 O_4,3(C_6 H_6)

Compound Name: bis((2,2'-Methylene-bis(6-t-butyl-4-methylphenolato))-dimethylamino-molybdenum) benzene solvate

Space Group: P21/c **Cell:** **a** 15.229(2) **b** 16.947(2) **c** 24.950(3)
Space Group No.: 14 **(Å,°)** α 90.00 β 106.96(1) γ 90.00

R-Factor (%): 4.57 **Temperature(K):** 103 **Density(g/cm³):** 1.285

Parameters

Fragment 1
DIST1 (D) 2.217

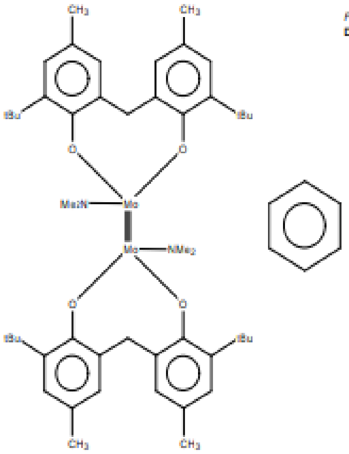

SOMXEX

Reference: R.G.Abbott, F.A.Cotton, L.R.Falvello (1990) *Polyhedron* ,**9**, 1821

Formula: C_{20} H_{12} F_{36} Mo_2 N_2 O_4

Compound Name: bis(bis(Perfluoro-t-butoxy)-dimethylamino-molybdenum)

Space Group: P21/n **Cell:** **a** 10.571(3) **b** 14.927(3) **c** 12.099(3)
Space Group No.: 14 **(Å,°)** α 90.00 β 103.21(3) γ 90.00

R-Factor (%): 8.80 **Temperature(K):** 295 **Density(g/cm³):** 2.180

Parameters

Fragment 1
DIST1 (D) 2.216

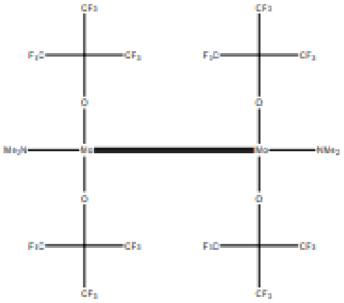

Search: search8 (Mon Jan 27 10:55:10 2025): Hits 45-48

SONYOM

Reference: R.Rogers (2019) *CSD Communication(Private Communication)* ,

Formula: C_{66} H_{102} Mo_2 O_6

Compound Name: hexakis((adamantan-1-yl)methanolato)-di-molybdenum

**Space Group:**	P-1	**Cell:**	***a*** 10.383(0)	***b*** 11.903(0)	***c*** 12.761(0)
**Space Group No.:**	2	**(Å,°)**	α 90.96(0)	β 95.42(0)	γ 113.09(0)
R-Factor (%):	3.94	***Temperature(K):*** 173	***Density(g/cm^3):*** 1.363		

Parameters

Fragment 1
DIST1 (D) 2.229

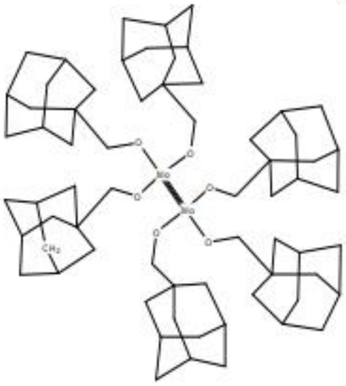

TIQMIP

Reference: M.J.A.Johnson, P.M.Lee, A.L.Odom, W.M.Davis, C.C.Cummins (1997) *Angew.Chem.,Int.Ed.* ,**36**,87

Formula: C_{12} H_{36} Mo_2 N_7,C_{12} H_{36} Mo_2 N_6

Compound Name: hexakis(Dimethylamido)-di-molybdenum (μ_2-nitrido)-bis(tris(dimethylamido)-molybdenum)

**Space Group:**	P-1	**Cell:**	***a*** 8.435(0)	***b*** 8.488(0)	***c*** 14.980(1)
**Space Group No.:**	2	**(Å,°)**	α 102.79(0)	β 92.06(0)	γ 97.33(0)
R-Factor (%):	6.71	***Temperature(K):*** 149	***Density(g/cm^3):*** 1.487		

Parameters

Fragment 1
DIST1 (D) 2.206

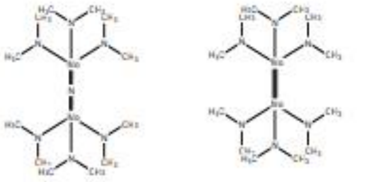

TMSIMO01

Reference: Fazlul Huq (2007) *Asian J.Chem.* ,**19**,49

Formula: C_{24} H_{66} Mo_2 Si_6

Compound Name: hexakis(trimethylsilylmethyl)-di-molybdenum

**Space Group:**	Pc	**Cell:**	***a*** 31.200	***b*** 12.457	***c*** 23.415
**Space Group No.:**	7	**(Å,°)**	α 90.00	β 112.17	γ 90.00
R-Factor (%):	4.20	***Temperature(K):*** 295	***Density(g/cm^3):*** 1.127		

Parameters

Fragment 1
DIST1 (D) 2.157

Fragment 2
DIST1 (D) 2.164

Fragment 3
DIST1 (D) 2.170

Fragment 4
DIST1 (D) 2.175

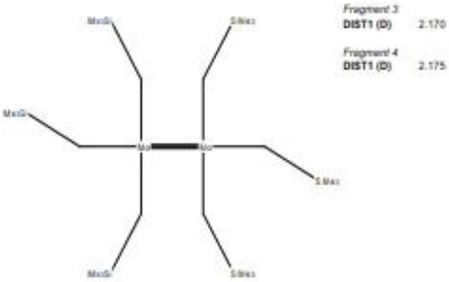

VERKOS

Reference: M.H.Chisholm, I.P.Parkin, J.C.Huffman, W.E.Streib (1990) *Chem.Commun.* ,920

Formula: C_{54} H_{66} Mo_2 Se_6

Compound Name: hexakis(2,4,6-Trimethylphenylseleno)-di-molybdenum

**Space Group:**	P-1	**Cell:**	***a*** 11.747(2)	***b*** 11.725(2)	***c*** 11.139(2)
**Space Group No.:**	2	**(Å,°)**	α 95.49(1)	β 96.11(1)	γ 118.20(1)
R-Factor (%):	4.15	***Temperature(K):*** 295	***Density(g/cm^3):*** 1.730		

Parameters

Fragment 1
DIST1 (D) 2.218

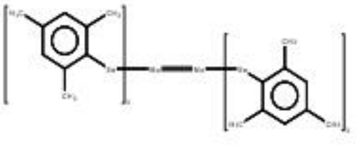

References

[1] D. Kratzert, I. Krossing *J. Appl. Cryst.* **2018**, 51, 928-934.

[2] D. Kratzert, J. Holstein, I. Krossing *J. Appl. Cryst.* **2015**, 48, 933-938.

[3] O. V. Dolomanov, L. J. Bourhis, R. J. Gildea, J. A. K. Howard, H. Puschmann *J. Appl. Cryst.* **2009**, 42, 339-341.

[4] P. van der Sluis, A. L. Spek *Acta Cryst*. **1990**, A46, 194-201.
