# Supplementary material for: Tripodal Silanolate Ligands Expand [MoX3] Chemistry Beyond Its Traditional Borders
Source: J Am Chem Soc. 2025 Apr 11;147(16):13871–84. doi: 10.1021/jacs.5c02178 (PMC12022994; doi:10.1021/jacs.5c02178)
Supplement: Supplementary file 3 — ja5c02178_si_003.pdf [file ja5c02178_si_003.pdf]

SUPPORTING INFORMATION

COMPUTATIONAL STUDIES

Tripodal Silanolate Ligands Expand [MoX_3] Chemistry Beyond Its Traditional Borders

Daniel Rütter, Nils Nöthling, Markus Leutzsch, Alexander A. Auer, Alois Fürstner*

Max-Planck-Institut für Kohlenforschung, 45470 Mülheim/Ruhr, Germany

Email: fuerstner@kofo.mpg.de

Table of Content

Computational Studies and Details of the Quantum Chemical Calculations S2

xyz Coordinates and Mo Shielding Tensors Obtained from the B3LYP-D3/def2-TZVP Optimizations S2

Cartesian Coordinates (Å) and Shielding Tensors S3

Compound **20** S3

Compound **19** S5

Compound **18** S7

Compound **14** S10

Compound **13** S15

Compound **12·thf (S=1/2)** S19

Compound **12·thf (S=3/2)** S22

Compound **12 (S=1/2)** S25

Compound **12 (S=3/2)** S28

Molecular Orbital Analysis of Selected Compounds S32

References S37

Computational Studies and Details of the Quantum Chemical Calculations

In order to simulate the chemical shifts of selected compounds, the geometries of all complexes were optimized at the B3LYP-D3(BJ)/def2-TZVP CPCM(toluene) level of theory [1] using the RI approximation and tight convergence and grid criteria. Minima were confirmed via normal mode analysis. NMR chemical shifts were then computed at the GIAO TPSSh/def2-TZVPP level of theory (for Mo, no ECPs and the uncontracted SARC-ZORA-TZVPP basis set was used) in conjunction with the Tau=Dobson flag [2]. Preliminary calculations with different functionals, with the inclusion of ZORA and with a variety of basis set options showed that this level of theory yields results in good agreement with the experimental values in agreement with our previous studies on the chemical shifts of Rh paddlewheel compounds [3]. All calculations were carried out within the ORCA 6.0 program package [4].

xyz Coordinates and Mo Shielding Tensors Obtained from the B3LYP-D3/def2-TZVP Optimizations

Sample input file for NMR calculations using ORCA 6.0 :

```
!TPSSH def2-TZVPP def2/J def2/JK NMR CPCM(Toluene)

%basis
decontract true
DelECP Mo
NewGTO Mo "SARC-ZORA-TZVPP" end
NewAuxJKGTO Mo "AutoAux" end
end

%eprnmr
Tau =  Dobson
end

*xyzfile 0 1 molecule.xyz
```

computed shielding values for selected compound with a comparison to the experimental relative shifts in ppm :

	σcalc			δcalc	δexp
	σ Mo1	σ Mo2	σ avg	δ calc	δexp
$Mo_2(OtBu)_6$	-2579.20	-2575.30	-2577.25	2588.7	2640
$Mo_2(NMe_2)_6$	-2596.60	-2596.90	-2596.75	2610.0	2441
$Mo_2(CH_2tBu)_6$	-3858.11	-3873.78	-3865.94	3995.1	3695
$tBuO_3$_Mo_MoNtBuAr$_3$	-3075.29	-	-3075.29	3132.2	3260
$tBuO_3$Mo_Mo_NtBuAr$_3$	-	-3169.49	-3169.49	3235.0	3127
Complex 11	-2582.88	-2581.943	-2582.41	2594.3	2631.5
(TripodalSiO)$_3$_Mo_Mo(NtBuAr)$_3$	-3596.91	-	-3596.91	3701.5	3314
(TripodalSiO)$_3$Mo_Mo_(NtBuAr)$_3$	-	-3234.90	-3234.90	3306.4	-

For labeling see coordinates and shielding tensors below.

Cartesian Coordinates (Å) and Shielding Tensors

Compound 20

Mo	-0.001541	5.774449	5.923738
C	2.068199	5.246929	6.174214
H	1.913372	4.159984	6.280567
H	2.646144	5.372365	5.258545
C	2.921477	5.750211	7.354407
C	3.193029	7.253201	7.212811
H	3.632752	7.480434	6.238841
H	3.885177	7.596774	7.985811
H	2.274504	7.833711	7.308685
C	4.267844	5.005450	7.328977
H	4.116263	3.927590	7.425297
H	4.913984	5.332152	8.149059
H	4.793892	5.187556	6.388876
C	2.236741	5.478967	8.697383
H	1.297022	6.025861	8.780485
H	2.873103	5.791258	9.529496
H	2.021791	4.415019	8.818972
C	-0.569022	7.833642	6.169688
H	0.455298	8.226875	6.283620
H	-0.952377	8.278640	5.251779
C	-1.434625	8.332648	7.341821
C	-2.876332	7.833991	7.183642
H	-3.281688	8.122630	6.211130
H	-3.520821	8.256733	7.958657
H	-2.932227	6.747114	7.260350
C	-1.443168	9.871553	7.315489
H	-0.430006	10.265759	7.424879
H	-2.054646	10.275306	8.127749
H	-1.847388	10.240462	6.369976
C	-0.875995	7.869579	8.690020
H	-0.892157	6.782775	8.773451
H	-1.468169	8.271523	9.516313
H	0.155122	8.205189	8.821436
C	-1.493384	4.246563	6.169303
H	-2.357993	4.925939	6.258511
H	-1.663645	3.672720	5.258179
C	-1.490806	3.269791	7.360372
C	-0.323445	2.282798	7.233476
H	-0.352164	1.768531	6.270044
H	-0.367041	1.527811	8.022564
H	0.638246	2.791518	7.312647
C	-2.808044	2.473902	7.339147
H	-3.666443	3.143884	7.429546
H	-2.846831	1.758060	8.165371
H	-2.910939	1.919063	6.403713
C	-1.390753	4.010820	8.696856
H	-0.453315	4.561752	8.777528

H	-1.430994	3.309251	9.534227
H	-2.213269	4.719935	8.814053
Mo	-0.000410	5.777173	3.769598
C	-2.057061	6.325905	3.470622
H	-1.833728	7.345664	3.107445
H	-2.611363	6.457838	4.400879
C	-2.973954	5.624590	2.449525
C	-3.382071	4.238535	2.963807
H	-3.846263	4.307537	3.949637
H	-4.097349	3.771403	2.282207
H	-2.521127	3.574501	3.048792
C	-4.246584	6.472634	2.275112
H	-3.999736	7.468539	1.899707
H	-4.935957	6.003630	1.567099
H	-4.767519	6.590691	3.228051
C	-2.293485	5.481515	1.083864
H	-1.377069	4.893341	1.151428
H	-2.955033	4.982227	0.371311
H	-2.035084	6.458644	0.670716
C	0.555045	3.725550	3.454956
H	-0.435266	3.420009	3.070963
H	0.702061	3.166206	4.379972
C	1.629395	3.289082	2.440129
C	3.031458	3.599327	2.978208
H	3.187816	3.134987	3.953966
H	3.796390	3.221776	2.294936
H	3.189324	4.672478	3.093415
C	1.513485	1.767789	2.235079
H	0.530041	1.504615	1.838455
H	2.270626	1.407687	1.532555
H	1.648802	1.238111	3.180841
C	1.437782	3.979175	1.085253
H	1.514426	5.063705	1.174020
H	2.197679	3.650959	0.371508
H	0.458870	3.745013	0.662204
C	1.499566	7.286941	3.467101
H	2.265516	6.583060	3.093453
H	1.899513	7.695020	4.396211
C	1.348275	8.435833	2.450806
C	0.391781	9.504101	2.994057
H	0.728837	9.873035	3.964812
H	0.335380	10.353259	2.308217
H	-0.617771	9.110830	3.120445
C	2.729458	9.081414	2.238293
H	3.440612	8.351819	1.843709
H	2.668400	9.913705	1.531129
H	3.127228	9.465007	3.180623
C	0.832093	7.932844	1.098539
H	-0.153580	7.475338	1.192548
H	0.747490	8.757192	0.385971
H	1.508328	7.190179	0.670728

Mo shielding tensors:

Nucleus 0Mo:
Diamagnetic contribution to the shielding tensor (ppm) :

4090.413	-0.742	3.487
-0.151	4089.474	-0.285
3.589	-10.295	4086.923

Paramagnetic contribution to the shielding tensor (ppm):

-8137.766	95.447	-9.662
-98.170	-8144.449	-1.041
-3.754	1.333	-7558.923

Total shielding tensor (ppm):

-4047.353	94.705	-6.175
-98.320	-4054.975	-1.326
-0.166	-8.962	-3472.000

Diagonalized matrix:

sDSO	4086.968	4090.600	4089.242	iso=	4088.937
sPSO	-7558.906	-8137.553	-8144.679	iso=	-7947.046
Total	-3471.938	-4046.953	-4055.437	iso=	-3858.109

Nucleus 49Mo:
Diamagnetic contribution to the shielding tensor (ppm) :

4064.299	-2.478	14.591
2.130	4064.556	-0.266
0.960	11.528	4085.379

Paramagnetic contribution to the shielding tensor (ppm):

-8199.844	80.329	-10.617
-78.186	-8202.636	-2.154
-4.584	-7.458	-7433.087

Total shielding tensor (ppm):

-4135.546	77.851	3.974
-76.056	-4138.080	-2.420
-3.624	4.070	-3347.707

Diagonalized matrix:

sDSO	4085.397	4064.208	4064.630	iso=	4071.412
sPSO	-7433.103	-8199.468	-8202.996	iso=	-7945.189
Total	-3347.707	-4135.260	-4138.366	iso=	-3873.778

Compound 19

Mo	2.668255	2.772685	6.521410
N	1.251837	3.698107	7.550674
N	3.687433	3.935309	5.283191
N	2.128406	1.024245	5.762819
C	0.118751	4.205838	6.792476
H	0.224100	3.996012	5.726219
H	0.017513	5.294474	6.904649
H	-0.824115	3.750430	7.126525
C	1.100529	3.994119	8.960510
H	1.948292	3.615026	9.524927
H	0.184340	3.539691	9.364022
H	1.026218	5.077616	9.131628

C	2.972798	4.447075	4.123931
H	1.936962	4.102617	4.106740
H	3.443499	4.119091	3.186419
H	2.954310	5.545945	4.117403
C	5.060464	4.397014	5.284099
H	5.588659	4.024964	6.157811
H	5.108230	5.495402	5.292373
H	5.593511	4.056711	4.384835
C	1.113122	1.045204	4.721089
H	0.788253	2.063388	4.498568
H	0.222490	0.474231	5.019062
H	1.488739	0.607035	3.785815
C	2.540973	-0.334222	6.050104
H	3.304366	-0.347123	6.823242
H	2.947110	-0.822692	5.152784
H	1.691379	-0.942171	6.392770
Mo	4.146395	2.232603	8.054368
N	5.562753	1.307197	7.024997
C	6.697265	0.801999	7.782733
H	6.592902	1.013524	8.848789
H	6.799563	-0.286725	7.672277
H	7.639258	1.257879	7.446826
C	5.712752	1.009056	5.615480
H	4.863876	1.386213	5.051461
H	6.627925	1.463909	5.210147
H	5.788148	-0.074637	5.446053
N	3.127137	1.069927	9.292490
C	3.841243	0.559357	10.452594
H	4.876830	0.904630	10.470400
H	3.369546	0.887515	11.389562
H	3.860568	-0.539507	10.459742
C	1.754446	0.607219	9.290750
H	1.226688	0.978385	8.416405
H	1.707494	-0.491215	9.283064
H	1.220442	0.947659	10.189401
N	4.686242	3.981072	8.812936
C	5.702934	3.960247	9.853289
H	6.028740	2.942113	10.074867
H	6.592821	4.531930	9.554398
H	5.328283	4.397720	10.789293
C	4.272431	5.339401	8.526814
H	3.507993	5.352150	7.754713
H	3.867194	5.827215	9.424904
H	5.121185	5.948028	8.183266

Mo shielding tensors:

Nucleus 0Mo:
Diamagnetic contribution to the shielding tensor (ppm) :

4089.918	2.124	0.034
0.494	4091.002	-0.453
-1.564	2.638	4092.414

Paramagnetic contribution to the shielding tensor (ppm):

-6350.066	-472.417	1332.801
-470.241	-7460.590	-489.419
1337.014	-488.184	-6252.461

Total shielding tensor (ppm):

-2260.148	-470.293	1332.835
-469.747	-3369.589	-489.873
1335.449	-485.547	-2160.047

Diagonalized matrix:

sDSO	4089.673	4091.792	4091.869 iso=	4091.111
sPSO	-4792.645	-7633.416	-7637.057 iso=	-6687.706
Total	-702.972	-3541.624	-3545.188 iso=	-2596.595

Nucleus 28Mo:

Diamagnetic contribution to the shielding tensor (ppm) :

4097.656	0.222	-6.487
1.622	4102.140	3.251
-5.575	-0.292	4094.216

Paramagnetic contribution to the shielding tensor (ppm):

-6358.772	-470.789	1340.047
-471.268	-7471.396	-492.080
1341.195	-484.508	-6254.552

Total shielding tensor (ppm):

-2261.116	-470.567	1333.561
-469.645	-3369.256	-488.829
1335.621	-484.799	-2160.337

Diagonalized matrix:

sDSO	4089.776	4102.197	4102.038 iso=	4098.004
sPSO	-4793.164	-7643.091	-7648.465 iso=	-6694.907
Total	-703.389	-3540.893	-3546.427 iso=	-2596.903

Compound 18

Mo	28.282923	8.363074	10.536403
O	27.060400	9.731196	10.992420
O	27.542944	6.700021	9.913819
O	29.541685	8.184679	11.916406
C	26.856069	11.149124	11.052938
C	28.045364	11.763919	11.788940
H	28.138865	11.320767	12.781151
H	27.911784	12.841896	11.901009
H	28.962330	11.579633	11.229353
C	26.728814	11.732680	9.650797
H	27.611242	11.499524	9.059218
H	26.628678	12.818554	9.704436
H	25.849505	11.334240	9.146352
C	25.562254	11.354119	11.839557

H	25.655933	10.923686	12.837763
H	24.729425	10.868210	11.328370
H	25.337053	12.417894	11.937908
C	26.716457	5.825631	10.713552
C	25.510805	6.595680	11.254137
H	25.823390	7.395360	11.928648
H	24.848474	5.926962	11.806964
H	24.947681	7.043083	10.434239
C	27.546712	5.244565	11.857802
H	27.911868	6.035881	12.514816
H	28.407922	4.703392	11.463886
H	26.946616	4.553991	12.453390
C	26.259280	4.716373	9.767863
H	27.123435	4.188407	9.362535
H	25.692011	5.138970	8.937186
H	25.625893	3.998175	10.292394
C	30.846336	8.360598	12.481139
C	31.238971	7.020745	13.102612
H	30.498658	6.720441	13.845759
H	32.211615	7.097942	13.592122
H	31.295049	6.246417	12.338615
C	31.848914	8.799541	11.420977
H	31.894657	8.081533	10.604499
H	32.843582	8.885591	11.862791
H	31.561811	9.763673	11.007077
C	30.712148	9.435329	13.559767
H	29.960704	9.141997	14.294424
H	30.409879	10.381522	13.110977
H	31.664423	9.581429	14.073340
Mo	29.363610	8.985921	8.676123
O	30.586124	7.617798	8.220095
O	30.103597	10.648969	9.298713
O	28.104842	9.164330	7.296127
C	30.790501	6.199875	8.159622
C	29.601209	5.585016	7.423671
H	29.507669	6.028129	6.431448
H	29.734821	4.507039	7.311636
H	28.684250	5.769291	7.983275
C	30.917808	5.616375	9.561781
H	30.035384	5.849519	10.153370
H	31.017981	4.530502	9.508178
H	31.797114	6.014862	10.066193
C	32.084304	5.994894	7.372980
H	31.990587	6.425287	6.374760
H	32.917128	6.480852	7.884129
H	32.309539	4.931124	7.274665
C	30.930062	11.523377	8.498977
C	32.135730	10.753349	7.958397
H	31.823156	9.953649	7.283904
H	32.798041	11.422072	7.405553
H	32.698871	10.305974	8.778299
C	30.099798	12.104422	7.354724

H	29.734662	11.313099	6.697706
H	29.238573	12.645575	7.748635
H	30.699881	12.795010	6.759140
C	31.387211	12.632649	9.444662
H	30.523042	13.160595	9.849986
H	31.954487	12.210068	10.275342
H	32.020583	13.350859	8.920131
C	26.800193	8.988396	6.731393
C	26.407578	10.328221	6.109849
H	27.147902	10.628481	5.366694
H	25.434939	10.251007	5.620330
H	26.351498	11.102590	6.873805
C	25.797598	8.549518	7.791566
H	25.751852	9.267570	8.608004
H	24.802934	8.463450	7.349746
H	26.084689	7.585406	8.205520
C	26.934377	7.913609	5.652819
H	27.685835	8.206894	4.918157
H	27.236625	6.967434	6.101658
H	25.982106	7.767499	5.139241

Nucleus 0Mo:

Diamagnetic contribution to the shielding tensor (ppm) :

4113.315	-10.767	28.865
-0.863	4106.821	-8.379
-42.253	11.676	4041.334

Paramagnetic contribution to the shielding tensor (ppm):

-6991.090	603.509	-1332.142
483.863	-7660.751	-696.805
-1529.418	-1060.804	-5347.230

Total shielding tensor (ppm):

-2877.775	592.742	-1303.278
483.000	-3553.929	-705.184
-1571.671	-1049.128	-1305.896

Diagonalized matrix:

sDSO	4064.067	4089.509	4107.895	iso=	4087.157
sPSO	-4219.051	-7812.993	-7967.027	iso=	-6666.357
Total	-154.984	-3723.484	-3859.132	iso=	-2579.200

Nucleus 43Mo:

Diamagnetic contribution to the shielding tensor (ppm) :

4087.879	4.243	-18.376
-13.416	4086.144	20.158
44.896	-14.906	4157.401

Paramagnetic contribution to the shielding tensor (ppm):

-6965.433	585.861	-1286.290
493.635	-7631.768	-723.732
-1614.704	-1033.519	-5460.111

Total shielding tensor (ppm):

-2877.554	590.104	-1304.666

480.219	-3545.624	-703.575
-1569.808	-1048.425	-1302.709

Diagonalized matrix:

sDSO	4125.512	4115.656	4090.256	iso=	4110.475
sPSO	-4279.058	-7838.292	-7939.961	iso=	-6685.770
Total	-153.547	-3722.636	-3849.705	iso=	-2575.296

Compound 14

Mo	7.874496	19.298547	16.692237
Mo	7.217774	17.641441	18.134746
Si	8.122442	22.387847	18.466334
Si	11.052713	18.426552	15.307206
Si	5.447475	19.617578	14.095788
O	8.003068	20.897819	17.777203
O	9.577215	18.702209	15.982353
O	6.461950	19.390623	15.371681
N	8.160500	17.871967	19.880483
N	7.671837	15.864686	17.344570
N	5.246618	17.757803	18.427856
C	7.352058	17.487917	21.004187
C	6.525434	18.431449	21.622969
H	6.477016	19.429827	21.212433
C	5.773899	18.107760	22.745648
C	5.872052	16.813350	23.265032
H	5.295119	16.548962	24.145041
C	6.695446	15.857946	22.677856
C	7.418984	16.202827	21.534935
H	8.052297	15.469818	21.060596
C	4.866197	19.124920	23.384052
H	5.079127	19.231286	24.450466
H	3.817995	18.827235	23.292296
H	4.979940	20.101193	22.914964
C	6.839699	14.484035	23.275965
H	6.844103	13.715735	22.503720
H	6.033154	14.268043	23.977533
H	7.784978	14.396788	23.819631
C	9.510462	18.366435	20.296239
C	10.225390	17.359553	21.213377
H	9.718257	17.246360	22.169720
H	11.235039	17.726276	21.408239
H	10.300254	16.381977	20.743075
C	9.394844	19.703968	21.019471
H	8.856859	20.402158	20.391163
H	10.385717	20.115498	21.222361
H	8.861173	19.612409	21.964681
C	10.360340	18.546818	19.061106
H	10.427371	17.626763	18.490136
H	11.364337	18.849755	19.349435
H	9.961701	19.324358	18.420675

C	8.066490	14.904296	18.336596
C	7.146795	14.012985	18.882100
H	6.110202	14.081528	18.591694
C	7.548574	13.027844	19.785368
C	8.885848	12.970562	20.164299
H	9.203650	12.219536	20.879914
C	9.828887	13.862477	19.644916
C	9.406916	14.816040	18.726744
H	10.119505	15.510556	18.304551
C	6.549486	12.030889	20.309391
H	6.929855	11.512347	21.190118
H	6.323150	11.276098	19.550670
H	5.608164	12.513731	20.569885
C	11.269759	13.789070	20.074302
H	11.359117	13.853664	21.161515
H	11.851664	14.597859	19.635591
H	11.725076	12.843741	19.767254
C	7.726123	15.301934	15.959254
C	6.923607	13.995290	15.844564
H	7.361972	13.191474	16.433220
H	6.920023	13.683646	14.798221
H	5.893745	14.136900	16.163565
C	9.170014	15.039617	15.545367
H	9.736659	15.955225	15.653327
H	9.219853	14.724311	14.501345
H	9.635818	14.271006	16.160558
C	7.116070	16.301347	15.005837
H	6.103260	16.557493	15.297239
H	7.094289	15.879045	14.003550
H	7.704740	17.209000	14.960255
C	4.636942	16.465638	18.577532
C	4.434373	15.907729	19.836500
H	4.790786	16.433199	20.708411
C	3.762328	14.693771	19.986148
C	3.322794	14.025445	18.847965
H	2.808855	13.075944	18.954130
C	3.527518	14.553189	17.569419
C	4.177744	15.775392	17.450879
H	4.338543	16.208075	16.473359
C	3.494119	14.144888	21.362159
H	3.178049	13.102018	21.319818
H	2.701727	14.713183	21.857697
H	4.378831	14.214142	21.994420
C	3.066526	13.803553	16.348218
H	2.024371	13.490975	16.445571
H	3.662659	12.899273	16.196091
H	3.156317	14.418398	15.453885
C	4.246598	18.868487	18.513242
C	3.373366	18.749613	19.774129
H	2.714292	17.883866	19.737943
H	2.750899	19.643447	19.848329
H	3.985978	18.682876	20.670416

C	4.985383	20.183208	18.595475
H	5.634603	20.219020	19.463776
H	4.269658	20.999282	18.663133
H	5.589582	20.351963	17.712277
C	3.347388	18.878206	17.281746
H	3.958963	18.966949	16.392922
H	2.663216	19.728817	17.314130
H	2.756032	17.967208	17.201089
C	7.489543	23.807946	17.374601
C	6.975504	24.938380	18.029645
H	6.959643	24.965266	19.110121
C	6.454824	26.023605	17.337675
H	6.067998	26.876284	17.881653
C	6.404922	25.992530	15.949468
H	5.973752	26.816938	15.395473
C	6.935091	24.903686	15.274313
H	6.940898	24.889427	14.191629
C	7.510644	23.834515	15.967822
C	8.209229	22.792692	15.185591
C	9.546250	22.510547	15.461136
H	10.050414	23.038938	16.257021
C	10.242411	21.565771	14.716330
C	9.592069	20.910281	13.672603
H	10.131545	20.190348	13.074873
C	8.258901	21.176547	13.383762
C	7.579868	22.132555	14.136634
H	6.552708	22.367395	13.901280
C	11.672995	21.287764	14.961267
C	12.570660	22.359078	14.925888
H	12.183284	23.363771	14.811574
C	13.939672	22.146449	14.984331
H	14.621452	22.986583	14.941418
C	14.424628	20.847455	15.074697
H	15.490995	20.660585	15.098027
C	13.533250	19.787032	15.167401
H	13.934041	18.790152	15.285796
C	12.142070	19.973496	15.139732
C	7.570269	20.510640	12.257749
C	6.328831	19.870736	12.431926
C	5.670166	19.424322	11.275166
H	4.706759	18.942808	11.364790
C	6.223664	19.556773	10.008656
H	5.680847	19.200494	9.142018
C	7.485937	20.118848	9.866442
H	7.943953	20.203684	8.888930
C	8.149499	20.592956	10.988044
H	9.116102	21.069560	10.883404
C	7.108221	22.549002	20.057755
C	7.680763	22.425819	21.327958
H	8.746181	22.273616	21.424618
C	6.920427	22.496096	22.492564
C	5.550322	22.730513	22.379830

H	4.945532	22.792343	23.278706
C	4.943274	22.894948	21.137409
C	5.737525	22.807577	19.996447
H	5.265832	22.947834	19.035049
C	7.556173	22.272740	23.838112
H	7.454518	21.225336	24.139313
H	7.084986	22.881702	24.611489
H	8.621649	22.504444	23.815198
C	3.460138	23.121247	21.018509
H	3.234219	23.897599	20.285444
H	3.023680	23.412643	21.974412
H	2.955616	22.208443	20.686992
C	9.904494	22.760639	18.924592
C	10.989990	21.972044	18.549577
H	10.831529	21.099514	17.932555
C	12.291040	22.280801	18.949617
C	12.499413	23.427763	19.711058
H	13.507935	23.679218	20.023366
C	11.443571	24.262413	20.079475
C	10.156276	23.908850	19.686277
H	9.332997	24.545468	19.990455
C	13.435854	21.373527	18.596280
H	13.508663	20.547279	19.309675
H	14.387168	21.906603	18.612089
H	13.304152	20.941774	17.606724
C	11.694893	25.522079	20.864972
H	12.504519	25.387382	21.584440
H	10.801128	25.834222	21.406698
H	11.981647	26.344019	20.202253
C	12.124816	17.231060	16.314010
C	12.256914	15.878631	15.982464
H	11.757554	15.489657	15.106921
C	13.021998	15.000387	16.745600
C	13.698620	15.500881	17.857281
H	14.300544	14.828760	18.460051
C	13.621692	16.846613	18.207169
C	12.841069	17.690915	17.421122
H	12.799664	18.737831	17.684412
C	14.330591	17.378844	19.423448
H	13.615364	17.599897	20.221410
H	14.860151	18.307216	19.200782
H	15.050434	16.658046	19.812544
C	13.074203	13.535495	16.404977
H	14.037523	13.096198	16.669047
H	12.896697	13.370202	15.341579
H	12.302675	12.987391	16.954797
C	10.862123	17.664765	13.601026
C	12.014226	17.190263	12.961780
H	12.978929	17.285904	13.447048
C	11.962731	16.579655	11.711827
C	10.717029	16.424122	11.102884
H	10.656252	15.929800	10.138546

C	9.549416	16.889944	11.702246
C	9.643394	17.528005	12.939295
H	8.742403	17.917799	13.389418
C	13.218982	16.110966	11.026807
H	13.573471	16.856819	10.309161
H	13.048824	15.184768	10.474866
H	14.020771	15.940704	11.746436
C	8.210281	16.669316	11.055385
H	7.763389	15.733746	11.405986
H	8.295524	16.606527	9.969883
H	7.518958	17.473224	11.296993
C	4.288930	18.153050	13.787592
C	4.739886	17.011812	13.120987
H	5.757437	16.973432	12.760381
C	3.918475	15.911776	12.888257
C	2.593789	15.974082	13.315149
H	1.938235	15.127622	13.137784
C	2.090795	17.103559	13.959642
C	2.946219	18.181147	14.176914
H	2.546825	19.062431	14.659667
C	4.468666	14.675135	12.230187
H	4.992277	14.051751	12.961701
H	5.187303	14.928021	11.448871
H	3.675763	14.070255	11.788613
C	0.667887	17.140795	14.449130
H	0.582657	16.645897	15.421409
H	-0.006013	16.626347	13.761800
H	0.319041	18.166737	14.572409
C	4.339391	21.106154	14.377665
C	3.413070	21.420602	13.372794
H	3.372037	20.820575	12.470677
C	2.534071	22.490186	13.494531
C	2.581681	23.255547	14.662799
H	1.897881	24.090487	14.778147
C	3.487877	22.977356	15.680065
C	4.370474	21.906423	15.514771
H	5.096243	21.705383	16.288763
C	1.546360	22.816240	12.405879
H	0.518180	22.713029	12.762763
H	1.673520	22.154325	11.549012
H	1.665293	23.846104	12.060274
C	3.506510	23.792288	16.942532
H	2.929738	24.710900	16.831197
H	4.523908	24.061684	17.220392
H	3.076875	23.227336	17.774235

Nucleus 0Mo:
Diamagnetic contribution to the shielding tensor (ppm) :

4274.526	217.226	-177.727
-41.046	3938.821	-34.365
-22.391	94.157	4223.490

Paramagnetic contribution to the shielding tensor (ppm):

-9249.777 1071.619 -635.319
1017.142 -6520.262 -2504.928
-1138.221 -2518.506 -7457.517
Total shielding tensor (ppm):
-4975.252 1288.844 -813.046
976.096 -2581.441 -2539.293
-1160.612 -2424.349 -3234.027

Diagonalized matrix:

sDSO	4126.131	4148.073	4162.633	iso= 4145.612
sPSO	-4081.266	-9557.535	-9588.756	iso= -7742.519
Total	44.865	-5409.462	-5426.122	iso= -3596.907

Nucleus 1Mo:
Diamagnetic contribution to the shielding tensor (ppm) :
4259.460 222.045 -194.840
-27.662 4023.937 64.125
-11.075 32.183 4169.069
Paramagnetic contribution to the shielding tensor (ppm):
-8310.047 330.216 -507.403
805.832 -6647.230 -1481.496
-431.970 -1535.396 -7199.901
Total shielding tensor (ppm):
-4050.587 552.261 -702.243
778.170 -2623.293 -1417.371
-443.045 -1503.212 -3030.831

Diagonalized matrix:

sDSO	4133.196	4158.960	4160.310	iso= 4150.822
sPSO	-5225.407	-8458.685	-8473.086	iso= -7385.726
Total	-1092.211	-4299.724	-4312.776	iso= -3234.904

Compound 13

Mo	12.917238	8.664912	7.005544
Si	12.810007	10.911811	9.695674
Si	10.094991	6.824166	6.353530
Si	14.051872	10.227080	4.067034
O	12.739785	9.743270	8.552532
O	11.599475	7.307906	6.814667
O	13.454207	9.495897	5.404540
C	11.285986	11.988113	6.892397
C	10.347819	11.127192	7.456499
H	10.130654	11.196739	8.511304
C	9.694796	10.158786	6.692686
C	9.973701	10.082877	5.328192
H	9.461615	9.355547	4.718097
C	10.911982	10.929491	4.738835
C	11.540958	11.889885	5.525445
H	12.265589	12.549333	5.070539

C	12.026317	12.978363	7.712823
C	12.682821	12.632245	8.913886
C	13.349737	13.655550	9.604564
H	13.871409	13.423418	10.525120
C	13.384823	14.965802	9.144986
H	13.910756	15.725894	9.709138
C	12.749434	15.286813	7.952182
H	12.768754	16.301535	7.574639
C	12.080910	14.297178	7.247005
H	11.572068	14.548506	6.324711
C	8.706007	9.256713	7.336177
C	8.755496	7.848406	7.223613
C	7.747164	7.116773	7.869681
H	7.755530	6.036446	7.813133
C	6.734136	7.723577	8.602251
H	5.975656	7.118799	9.083439
C	6.711736	9.106253	8.719422
H	5.934095	9.598919	9.289616
C	7.693475	9.858714	8.091017
H	7.669979	10.938695	8.165820
C	11.269714	10.789762	3.305593
C	12.604578	10.575814	2.898432
C	12.858642	10.497465	1.522082
H	13.870659	10.323130	1.176208
C	11.848164	10.612476	0.574935
H	12.082831	10.549086	-0.480359
C	10.535379	10.790464	0.993463
H	9.734727	10.869327	0.268681
C	10.253593	10.874931	2.350282
H	9.233671	11.030041	2.680478
Mo	14.693683	7.448331	7.486838
Si	14.798368	5.200633	4.798347
Si	17.512973	9.292745	8.142510
Si	13.559070	5.884091	10.424336
O	14.874139	6.371067	5.939169
O	16.009732	8.806527	7.680097
O	14.156532	6.616248	9.087233
C	16.327915	4.127592	7.600687
C	17.264660	4.990181	7.036702
H	17.481539	4.922167	5.981712
C	17.916725	5.958870	7.801120
C	17.637783	6.033827	9.165709
H	18.148951	6.761581	9.776084
C	16.700413	5.185952	9.754728
C	16.072980	4.224955	8.967719
H	15.349360	3.564135	9.422259
C	15.588458	3.136062	6.780780
C	14.928827	3.481013	5.581138
C	14.262426	2.456496	4.891708
H	13.738452	2.687669	3.972189
C	14.230823	1.146109	5.351135
H	13.705179	0.385083	4.787958

C	14.869461	0.826141	6.542483
H	14.852978	-0.188680	6.919906
C	15.537486	1.817005	7.246423
H	16.048696	1.566620	8.167664
C	18.904854	6.862385	7.158554
C	18.854307	8.270546	7.272783
C	19.862610	9.003710	6.628420
H	19.853400	10.083966	6.686478
C	20.876539	8.398521	5.895736
H	21.634888	9.004527	5.415858
C	20.899829	7.015979	5.776648
H	21.678130	6.524534	5.206284
C	19.918183	6.262006	6.403447
H	19.942512	5.182111	6.327289
C	16.341629	5.324955	11.187786
C	15.006095	5.536870	11.593886
C	14.750783	5.614373	12.970041
H	13.738227	5.787058	13.315148
C	15.760634	5.500562	13.917979
H	15.525000	5.563317	14.973090
C	17.074073	5.324733	13.500518
H	17.874210	5.246970	14.226069
C	17.357105	5.241075	12.143922
H	18.377517	5.087563	11.814474
C	13.147140	5.340912	3.933887
H	13.080745	4.715178	3.041903
H	12.334589	5.067139	4.606593
H	12.993448	6.378384	3.629386
C	16.165487	5.433127	3.538872
H	17.149059	5.543298	3.996773
H	16.197986	4.564589	2.875500
H	15.979207	6.317914	2.929174
C	17.673209	11.078415	7.599079
H	18.571361	11.551539	8.002755
H	17.688198	11.183707	6.513382
H	16.808308	11.629085	7.977482
C	17.662400	9.248092	10.015783
H	16.991623	8.512178	10.459916
H	18.683658	9.007977	10.320543
H	17.409274	10.223562	10.433449
C	12.379752	7.058857	11.274093
H	11.671889	7.442854	10.536241
H	11.809817	6.573225	12.069062
H	12.914121	7.906218	11.705152
C	12.618192	4.354827	9.903680
H	13.187243	3.729749	9.215634
H	12.343776	3.750938	10.772201
H	11.698922	4.656873	9.397089
C	14.457947	10.771486	10.566354
H	14.519283	11.393593	11.461281
H	14.612559	9.733083	10.867119
H	15.272633	11.049857	9.898056

C	11.438526	10.674813	10.949838
H	10.457948	10.555009	10.488058
H	11.628905	9.793822	11.563706
H	11.396255	11.545471	11.609793
C	9.932241	5.038999	6.897790
H	9.032387	4.567430	6.496081
H	10.795308	4.486513	6.517838
H	9.919194	4.934281	7.983583
C	9.944501	6.868668	4.480200
H	10.617533	7.602215	4.035526
H	10.194395	5.892254	4.062721
H	8.923895	7.111959	4.175596
C	15.229060	9.050622	3.216945
H	15.938619	8.667964	3.953921
H	14.693262	8.202534	2.789043
H	15.797109	9.534515	2.419526
C	14.994767	11.755421	4.586733
H	14.427683	12.379943	5.276890
H	15.915197	11.452452	5.090647
H	15.267083	12.360182	3.718109

Mo shielding tensors:

Nucleus 0Mo:

Diamagnetic contribution to the shielding tensor (ppm) :

4141.170	19.269	0.775
20.376	4105.821	1.014
-58.490	-1.137	4113.819

Paramagnetic contribution to the shielding tensor (ppm):

-5460.656	-1700.344	726.246
-1769.881	-6753.397	-473.592
790.446	-449.003	-7892.586

Total shielding tensor (ppm):

-1319.486	-1681.074	727.021
-1749.506	-2647.577	-472.577
731.956	-450.140	-3778.767

Diagonalized matrix:

sDSO	4101.067	4127.301	4132.442	iso=	4120.270
sPSO	-4051.286	-7943.702	-8111.652	iso=	-6702.213
Total	49.781	-3816.401	-3979.210	iso=	-2581.943

Nucleus 46Mo:

Diamagnetic contribution to the shielding tensor (ppm) :

4087.279	4.400	-16.636
5.743	4124.930	1.879
60.158	6.026	4145.840

Paramagnetic contribution to the shielding tensor (ppm):

-5405.857	-1685.334	744.674
-1753.206	-6773.218	-473.486
669.306	-458.194	-7927.614

Total shielding tensor (ppm):

-1318.579	-1680.935	728.038
-1747.463	-2648.289	-471.606
729.464	-452.168	-3781.774

Diagonalized matrix:

sDSO	4103.888	4124.931	4129.230	iso= 4119.349
sPSO	-4054.952	-7941.068	-8110.670	iso= -6702.230
Total	48.936	-3816.137	-3981.440	iso= -2582.880

Compound 12·thf (S=1/2)

Mo	5.95298538640105	8.53292347164540	4.50789888329139
Si	4.98672288865273	11.20503361350406	2.97372002598272
Si	8.69254666381755	8.08064405117859	6.61230191621170
Si	4.63931830041193	5.59613166864212	3.22044433752585
O	4.97743204860170	10.14123291200253	4.23624384726623
O	7.22682570676794	8.22826062163500	5.90068333475106
O	5.15350315093753	6.83924572687686	4.13547614864648
O	4.52132174725957	8.38570924497249	6.27930609170615
C	7.67182322916787	9.23158533845454	3.14264201011046
C	8.79405366942289	8.49861637248870	3.65887291063208
H	9.51632056637345	9.03156893112899	4.25944716145713
C	8.91364015107870	7.14298549675516	3.53868286229593
C	8.08746226721452	6.44262162218847	2.59055890711281
H	8.27921805491600	5.39888480861272	2.38064655979688
C	7.04506647665714	7.08440959104963	1.99992217547797
C	6.70030867390115	8.42650003975673	2.41315505995313
H	6.00979598563513	8.93825949524620	1.75862047949770
C	7.84663899832471	10.68971143698602	2.83916109295635
C	6.78361208520912	11.60393209167263	2.64071182620156
C	7.08376032288331	12.92057574174668	2.27225025378025
H	6.27137445616775	13.62481323146751	2.13617174201024
C	8.38895592931009	13.36550536509299	2.11183725139283
H	8.58799496842727	14.39270067326797	1.83372112992100
C	9.42969890682949	12.47469263369010	2.33056676102516
H	10.45849258600327	12.79476540978786	2.22083269603980
C	9.15671466116858	11.16002303602938	2.67851657426446
H	9.98412933909004	10.47646078132035	2.80562665645869
C	4.08798938302635	12.74204599374075	3.52943487016808
C	4.44744870975774	13.35081663939724	4.73980051726740
H	5.23887304273986	12.91751405801160	5.34144980873465
C	3.81655820174489	14.50343818316308	5.18849247108458
C	2.80212750232138	15.05441656866855	4.39824803803309
H	2.30141485246133	15.95609023364223	4.73667010693568
C	2.41705674103858	14.47950447300828	3.19304585044582
C	3.07178758243230	13.31756819250689	2.77151560074778
H	2.77613459882946	12.85516634629630	1.83583175892284
C	4.20715757153936	15.15262113329490	6.48939177920136
H	5.02410886193672	14.61305122132512	6.96895495963650

H	3.36588731862020	15.17988602798942	7.18679557343062
H	4.53150199038806	16.18472388699842	6.33477321496848
C	1.32407859056431	15.08542211736190	2.35343448054014
H	0.88833694056860	15.95960917280532	2.83796370396542
H	0.52303555777327	14.36509178083527	2.17100842164960
H	1.70470631936657	15.39632500696940	1.37721358528345
C	4.13944610246337	10.39574782498234	1.51721624896230
C	4.65932353278193	10.49618652556947	0.22313179939103
H	5.52341938673075	11.12609930023194	0.04082111767042
C	4.12371303052342	9.76423241650031	-0.83318536782379
C	3.03488339961774	8.93156988859891	-0.57760358574742
H	2.61734309672397	8.34592590493412	-1.38956043697507
C	2.47623369316605	8.81810695393118	0.69271856592870
C	3.04255852999468	9.55725743706501	1.73177452146723
H	2.63261115252210	9.45343381005014	2.73042879404699
C	4.72821815763065	9.82730624596128	-2.20907313554578
C	1.31887228297721	7.89077168284741	0.94274878558166
C	9.81941314648814	6.42945186451451	4.45975996471110
C	9.83704666879983	6.79140669784760	5.82870715343633
C	10.73662087261670	6.12867676870107	6.67311685782805
H	10.75723273743149	6.37840842064428	7.72740906354458
C	11.57666173762294	5.12511814564012	6.20657106553817
H	12.25582080641803	4.62528049627135	6.88577253491765
C	11.51537347404541	4.74724862312871	4.87022761775063
H	12.15145030028875	3.95356130295422	4.49842379403674
C	10.63948435495464	5.39155341245427	4.00908423366459
H	10.60498033207448	5.10661614710772	2.96493553700171
C	8.29997442547301	7.51336244825445	8.35832557069799
C	7.86681408214872	6.20547478899840	8.58579776293591
H	7.86268253606326	5.49314336280043	7.76932730720096
C	7.42329341696886	5.78737983152058	9.83937792397802
C	7.42319706638000	6.71137837363629	10.88234507903429
H	7.07242763541400	6.40226108204523	11.86183657046752
C	7.85600476897557	8.02492614100710	10.69858209777401
C	8.28764918765797	8.41019539951798	9.43026598946334
H	8.61537484811056	9.43200757400514	9.27376071618090
C	6.95993646498251	4.37102259672595	10.05400148676299
H	6.31054417225086	4.04026766643766	9.24103163193985
H	6.41125777494245	4.26898657735033	10.99087601043395
H	7.80849505910056	3.68216531433083	10.08943694973406
C	7.86996291810016	8.99493556569197	11.84999293372061
H	7.91390645743283	10.02586074124449	11.49737153435300
H	8.74059425149829	8.82893361927867	12.49103627355122
H	6.98191738157383	8.88416380457947	12.47539224403164
C	9.47220655192664	9.78371572452748	6.64891516485778
C	10.83967082130569	9.97063563905009	6.88114037123276
H	11.47460251751298	9.11225480929508	7.07390068154200
C	11.41639729142804	11.23618550269014	6.84477415113227
C	10.59815502375152	12.33279555618877	6.55930599678811
H	11.04007929083174	13.32281723978077	6.51007701292102
C	9.23576933469071	12.18738960196182	6.32264705497821
C	8.68850104448742	10.90479699495297	6.37961115107470

H	7.63166967850110	10.78291868947158	6.17293798218120
C	12.88618415653368	11.43003082725867	7.10670587179632
H	13.05267082236511	11.91572810955326	8.07236957332340
H	13.34555339157466	12.06268401079346	6.34398202247930
H	13.41408480088225	10.47616300930986	7.11925118536607
C	8.36629227550858	13.36590356746230	5.97979429610509
H	7.53249735457755	13.45718111168449	6.68053932739103
H	7.94265967824356	13.25583826161775	4.98001975034175
H	8.93024331027145	14.29860488688167	6.00502609475000
C	6.14891926504431	6.46983619332706	0.98236829886621
C	4.94980760404142	5.85732607977688	1.38707624601307
C	4.08143787717683	5.37786933715799	0.40303940749217
H	3.14547202762770	4.91679632961743	0.69460278716217
C	4.38523367319294	5.49386150069868	-0.94810169933544
H	3.69414999138116	5.12192270013978	-1.69449948813565
C	5.57645744585595	6.09395171912146	-1.33595018712573
H	5.82300800877038	6.18702583869911	-2.38648767454288
C	6.45049495024171	6.58528595925871	-0.37340769498279
H	7.37101460304471	7.07256462392966	-0.67099507194830
C	2.79574610607456	5.47368946353511	3.53852780483946
C	2.15562293098932	6.56050361400496	4.14024098825563
H	2.74134356177809	7.41876958729192	4.44520746234966
C	0.78017337064232	6.56905442289524	4.36234320271094
C	0.03764271988814	5.45724383197751	3.97279862948592
H	-1.03429071477215	5.44811619129357	4.14430467897498
C	0.63938157876027	4.34914965297599	3.37609635853230
C	2.01761506002418	4.37196082481293	3.17123009953349
H	2.48756465958306	3.50000491834449	2.72962705758197
C	0.11344202638138	7.77032599262029	4.97754258659994
H	0.82038525001063	8.35053149138009	5.57199348879301
H	-0.72193088569777	7.48237093018387	5.61819242481931
H	-0.28377790342902	8.43523429554624	4.20441122385819
C	-0.18793709597012	3.16413180449705	2.95360057233087
H	-0.91914180896315	2.89573697615694	3.71873585933954
H	0.43900501697167	2.29264050021451	2.76247909683619
H	-0.74341476752282	3.38036816765256	2.03644122702263
C	5.58211262128312	4.06373009691112	3.73438017889314
C	5.42492866954739	2.85607148957895	3.04558083357298
H	4.72935200698871	2.79379470113559	2.21505032028776
C	6.17255612295782	1.72830000383544	3.36929632780218
C	7.10919253512681	1.82879049204110	4.39931252976022
H	7.71468338325032	0.96317887185909	4.64881399170173
C	7.30238269599595	3.01468202252213	5.10210249508942
C	6.52636296321869	4.12362402252106	4.75979606995306
H	6.68794524733914	5.06161022250708	5.27793175673584
C	5.97968397904508	0.42941388992802	2.63290811121209
H	5.46081599498651	0.58523181268867	1.68634549545741
H	5.38507375476359	-0.27274358321797	3.22470225219551
H	6.93545455202058	-0.05419140619933	2.42136329192432
C	8.32470111798180	3.10295253853171	6.20228479790887
H	7.84942641958788	3.04589850045421	7.18581504414042
H	8.87525911101256	4.04181411129522	6.15620511184022

H	9.04641078052306	2.28799282433733	6.13902279101786
C	4.48747521231082	7.15189438067045	7.05907970423369
H	5.38537310016583	6.58225253110813	6.83747611192619
H	3.61381542393983	6.59338195701649	6.72533728515669
C	4.40422359225578	7.59026854980881	8.51766907320553
H	5.40088296100107	7.62848178349427	8.95591783852738
H	3.79671081743179	6.90861208488986	9.11214563076130
C	3.81366700547812	9.00109651234560	8.42563479328550
H	4.04000919540093	9.61511362262945	9.29700082437482
H	2.72919239297346	8.96590637709295	8.29917283311754
C	4.47392598594873	9.52950250865529	7.16607465525080
H	3.93135390045494	10.31462962426450	6.64642443892943
H	5.49689643927972	9.86178640420303	7.36497644296312
H	5.44300959624551	10.64648203560355	-2.29092859077737
H	5.25616093872047	8.89748455475770	-2.43731848863520
H	0.74887987642959	7.71598849963039	0.02931591069585
H	1.66797569616446	6.92385258805145	1.31015817881407
H	0.64347357878589	8.29291523204071	1.69850355469345
H	3.96277020717403	9.96349445283387	-2.97611361803611

Compound 12·thf (S=3/2)

Mo	5.58002780877807	8.59480690808268	4.57528750958410
Si	5.23455413941377	11.47505687863492	2.93986784334260
Si	8.30685075041302	7.79096598627942	6.54755918493698
Si	4.60799481225320	5.42961447221976	3.44785386495296
O	5.08394098097226	10.44801961572542	4.19484024563064
O	6.86056489455768	7.72342240553617	5.84726948724494
O	4.79680939049757	6.79246789651278	4.27631643593411
O	4.18189992933454	8.73697948995729	6.23883133764402
C	7.85888519010177	9.31794580152300	2.78659503136816
C	8.75599918212471	8.49451275043495	3.47970054384903
H	9.53559218112794	8.94527171633943	4.07112448137399
C	8.64135831492698	7.10675420382769	3.46250664669523
C	7.67424897025766	6.50835164298222	2.65190734583542
H	7.60918274052217	5.43182691574980	2.60670255655477
C	6.75371470630601	7.28922247820133	1.95886868112423
C	6.82639465312468	8.68875650168201	2.06558605738066
H	6.15171301653480	9.27935266262862	1.46718471995529
C	8.09617125396720	10.78572622041913	2.68887107796602
C	7.07470218573613	11.76554138142605	2.61199761876389
C	7.46330681914524	13.09894141193266	2.43062007993214
H	6.69974487586165	13.86513147250355	2.38938796415557
C	8.79454969880174	13.48555558249766	2.34419012514498
H	9.05000695665321	14.52905390832182	2.20925506949987
C	9.78592111518835	12.52285770192966	2.45017899987397
H	10.83151546703107	12.79902086862913	2.39888251963133
C	9.43347124088303	11.19253285437529	2.61366570114250
H	10.21429062989962	10.44646700920946	2.66490901679239
C	4.45025484753132	13.10313685127685	3.42591186058767
C	4.44298726885386	13.49015875477844	4.76514358086739

H	4.86702408600227	12.82771670279381	5.51046321862329
C	3.90359036628954	14.71172548177252	5.17131747901690
C	3.36307443712937	15.55027105790125	4.20179420835047
H	2.94235382963942	16.50438704527089	4.50307215672609
C	3.34067232717290	15.19358062559090	2.85126552121882
C	3.88456014209189	13.96725774329675	2.48186560464665
H	3.85817858505456	13.67609008694372	1.43730939202558
C	3.90539204055625	15.09946158105236	6.62621751888578
H	3.24330586130889	14.45115287192691	7.20681487810920
H	3.56968213972686	16.12747138573178	6.76555665050614
H	4.90516465641558	15.00878225675217	7.05718697329915
C	2.73781632722774	16.11956414347959	1.82863718726824
H	1.69243954259191	16.33819831527039	2.06026003135485
H	2.77644479767020	15.68288794326809	0.83057117516073
H	3.26825831189661	17.07491748002110	1.79904567089096
C	4.33605547319553	10.75878816469321	1.45982130505620
C	4.68835466998454	11.06130501455422	0.13972281201246
H	5.49726607649862	11.75845760563078	-0.05460738331935
C	4.04991149094311	10.45488360040098	-0.93811243165499
C	3.03686014332319	9.53003143829105	-0.67751100033332
H	2.54584965143443	9.03670251303945	-1.50951518320616
C	2.65777038783546	9.20304054632460	0.61961752162787
C	3.31651326073161	9.83202599445450	1.67736264428709
H	3.03964613888483	9.57176872918650	2.69337878277577
C	4.45409250185232	10.75759061224224	-2.35594985754896
C	1.58754149652363	8.18141680952021	0.88971612085899
C	9.48698035858526	6.28613221343570	4.36160485151813
C	9.49279574924792	6.54686294499964	5.74915505740513
C	10.32163976312947	5.76055933960387	6.55458920616389
H	10.32964611880987	5.92685494916922	7.62522249541589
C	11.10542967489877	4.74090408848115	6.02615616587225
H	11.73324895556680	4.14623219707870	6.67818676119776
C	11.05661693346171	4.47141959997314	4.66528526300751
H	11.64639821678927	3.66642343091630	4.24502813061492
C	10.24815521308221	5.24158759773907	3.83977261089651
H	10.21348724412154	5.04085758761040	2.77593595877043
C	8.09558937700340	7.32261797460044	8.35568250214756
C	7.11403155449121	6.39367991347480	8.71496383322030
H	6.49063230420587	5.95871137058656	7.94382883458562
C	6.90713951847790	6.01593638930257	10.03760761162928
C	7.71795431697040	6.58304163831259	11.02221970830988
H	7.57088062953603	6.29724352625426	12.05905847170099
C	8.70836262436925	7.50990749220748	10.70739944951683
C	8.87887064582398	7.87400532642881	9.36997255218068
H	9.63272532033093	8.61335229192641	9.12109684766953
C	5.81702697508606	5.04284808831970	10.39943624423913
H	4.93734340830487	5.56480816079100	10.78823425527394
H	6.14457965289541	4.34340669448232	11.17092827914574
H	5.49992894966044	4.46655940539413	9.52955651823091
C	9.59165635705484	8.09189928738559	11.77916715186237
H	9.84491183814032	9.13071384067942	11.56117558060495
H	10.53186967213461	7.53760165188596	11.85435040559858

H	9.11037097589133	8.05478682985865	12.75746604475492
C	9.06008506926573	9.51311783453314	6.47619963637579
C	10.44670043073310	9.70585512211802	6.45707279102718
H	11.10623586126285	8.84515297034122	6.50058468933423
C	11.00753181580349	10.97517538069777	6.35164187208574
C	10.15142417426675	12.07491628440712	6.26734395143780
H	10.57401449132267	13.06978655928969	6.17179151833834
C	8.76918340801005	11.92710432228866	6.28788617444101
C	8.24230015195297	10.63839562137906	6.39392787613602
H	7.16428544215716	10.52026666063318	6.38537702159038
C	12.50035391171673	11.16759423662537	6.32209870672295
H	12.84491211748375	11.72696087572069	7.19599806498995
H	12.80930950088602	11.73033400639880	5.43765553358590
H	13.02108449335451	10.20978992312890	6.31147070683391
C	7.85886717836364	13.11809185474622	6.17123619098797
H	7.06683722284161	13.08354766230738	6.92236713524063
H	7.37940615063798	13.14722160120616	5.19214430980148
H	8.40740434096331	14.05170276803704	6.29961815257595
C	5.71512023857303	6.68010901659682	1.08699722215714
C	4.76300795452791	5.77383661533403	1.59396471827885
C	3.83760100142135	5.23215141361613	0.69620273888915
H	3.07940471764443	4.55292297443912	1.06687964329868
C	3.83894778646222	5.56884490136117	-0.65267257544610
H	3.10738316324108	5.13225216227034	-1.32148326855886
C	4.76649911860888	6.48385031570808	-1.13084389987289
H	4.76638007892497	6.77050758728651	-2.17495899167960
C	5.69522452427512	7.03841650075812	-0.26160631268504
H	6.41973455942635	7.75379814867490	-0.63057056941740
C	2.85481474006814	4.83201002728154	3.74998990893364
C	1.84965862266317	5.78634220611545	3.95060204990402
H	2.11849069284866	6.83652742804628	3.97715529783336
C	0.51859719008046	5.42186942875841	4.11667815639537
C	0.19528178355722	4.06265742411271	4.08985236652182
H	-0.84007116128079	3.76217331102852	4.21753517457279
C	1.16673418446732	3.08374690751566	3.90745298991079
C	2.49421241248313	3.48658496048629	3.73624493000365
H	3.25879183554189	2.72916418007060	3.59712899804707
C	-0.55443248653081	6.46103835769667	4.30669616719757
H	-0.12358008738749	7.45979376763319	4.38317721659474
H	-1.13755099349738	6.27235158379530	5.21149301639431
H	-1.25460994474435	6.46346905449875	3.46695813681826
C	0.80591714965290	1.62184728087140	3.91676720306342
H	-0.26963395535646	1.47664972558625	3.80928502146306
H	1.11350486447318	1.14906132261043	4.85382508292601
H	1.30491445879224	1.08541008373624	3.10719400756227
C	5.83301748627464	4.10527036211477	3.94956009725341
C	6.18390873427198	3.07017772768780	3.07553441105012
H	5.75062292913128	3.03786197302578	2.08088360121702
C	7.10726253718296	2.09314746226810	3.43663183741253
C	7.67318149991386	2.15607883131517	4.71053095373952
H	8.39851411761590	1.40404381900397	5.00473674529546
C	7.34295509059312	3.16396742537446	5.61206728805819

C	6.43096665345637	4.14128260226867	5.20934414431455
H	6.20715028989309	4.96669777467516	5.87428753779594
C	7.49716612201859	0.99603051061792	2.48169108382303
H	6.99388849386445	1.11143986381285	1.52134095688879
H	7.23749423365596	0.01211237713609	2.88112248441918
H	8.57481425149920	0.99496603683073	2.29910003253577
C	7.93525792537926	3.17843522707972	6.99482754053753
H	7.25902413593904	2.69845763442949	7.70915521291383
H	8.11238423962284	4.19340856668827	7.34417416225989
H	8.88382054645473	2.64173690688607	7.02458811047183
C	3.89054721359159	7.54079603155580	7.03538379587597
H	4.69893259492896	6.83810267703883	6.87161280846119
H	2.96169187392304	7.12392043417879	6.64766844668075
C	3.77794096547728	8.04378551895602	8.46548468943580
H	4.75377335674499	8.00777796966342	8.95248276414397
H	3.07962528378911	7.44649507733591	9.05072334246937
C	3.32388932912728	9.49674591898373	8.28586136365717
H	3.53634484585892	10.12210572647695	9.15243846925038
H	2.25265058408406	9.54636374381040	8.07917505450505
C	4.11817795404501	9.92921056551250	7.06710469777725
H	3.66660517967202	10.71315000329128	6.46557831259984
H	5.13673607345365	10.21843070909601	7.33511582054129
H	5.24130297837383	11.51099243152752	-2.39106954490538
H	4.82534097497321	9.85997543939152	-2.85792514976228
H	1.13232514708505	7.82833322325559	-0.03561453873300
H	1.99955110781024	7.31535524778825	1.40964428111872
H	0.79817568529958	8.59588467285222	1.52153105972673
H	3.60739624417996	11.12589721438933	-2.94044800938222

Compound 12 (S=1/2)

Si	8.41664018991434	8.24031038386302	6.41377205700937
Si	4.56128898803584	5.51439691433088	3.12178210141250
O	7.02949073843101	8.21072892013672	5.56403768901778
O	4.81319185555210	7.03714735531330	3.70830311920801
C	8.59608365958566	7.00273206005458	3.47880259198438
C	7.79339894546515	6.29827879905229	2.52873219580248
H	7.70942768611958	5.22477394658075	2.59076965214474
C	7.00317240571223	7.00200645511642	1.62487124426169
C	4.30426052777187	11.36768830012368	-2.33691311080980
C	1.88639430820424	7.83771413751256	0.31695644546249
C	9.35939419636807	6.27565483410077	4.52002860704986
C	9.45326351257876	6.77284447979426	5.83875302483926
C	10.20603619014893	6.04127035783531	6.76240644780687
H	10.26454362806568	6.38628784583382	7.78763740415734
C	10.84711561373567	4.85844603147518	6.41561217452975
H	11.42293593893779	4.31537140403558	7.15471064369494
C	10.72756785679393	4.36905924695234	5.12153507659794
H	11.21051585762976	3.44186484801852	4.83955668471102
C	9.98841689111027	5.07472496622626	4.18392117261167
H	9.90757208894743	4.69387984319345	3.17362208695332

C	7.99231400268944	8.03092747921050	8.21828139815198
C	6.76549357717952	7.47684845927693	8.57879699055802
H	6.04881332144991	7.22746947613831	7.80436041000204
C	6.43409248253364	7.24728065311030	9.91582330757437
C	7.36026743356107	7.59127231018008	10.89470652843391
H	7.11550583432112	7.41724456203209	11.93778387222344
C	8.59383698150206	8.16420279003282	10.57314723460452
C	8.89264788276936	8.37930201417347	9.23199791534183
H	9.84086855432950	8.83982265678129	8.97376796811212
C	5.10649248018452	6.63556528091454	10.27501875704610
H	4.27946711344785	7.24297148269250	9.89972523629244
H	4.99153073789022	6.53762824957022	11.35486918835753
H	5.00079312801616	5.64156594520094	9.83270414099161
C	9.56237834207624	8.54673572116443	11.66048781884460
H	9.14183039551310	9.31950718663823	12.30927835893247
H	10.49277960947664	8.93164956691249	11.24237848773758
H	9.80514174280077	7.69036620543649	12.29456869055994
C	9.29413435944918	9.86500243824557	6.11917704756115
C	10.68589526765648	9.97059857514483	6.04134675208120
H	11.30005793253993	9.08312971053332	6.15846521043672
C	11.30730654496031	11.19050506473263	5.78148085928709
C	10.50573622167463	12.31903368767931	5.61206402720553
H	10.97686804529824	13.27380688620937	5.40042877254248
C	9.11648180746066	12.25588026452743	5.69647761688050
C	8.52461495978230	11.01640462418890	5.93722227696996
H	7.44379187923044	10.94070589605244	5.95819535805567
C	12.80642755148767	11.29698970500836	5.68796354062945
H	13.21678728516261	11.85026328781966	6.53726291566262
H	13.11068734790588	11.82598325722265	4.78180937839469
H	13.27275452342228	10.31130626202045	5.67730551928770
C	8.27954546265290	13.49833956559661	5.56217982914732
H	8.14931702352875	13.97903599243503	6.53673156014797
H	7.28811285102168	13.27182103781671	5.17381711439367
H	8.74963830963310	14.22327947008710	4.89719734735435
C	6.01996552702492	6.31061312565139	0.74839549316232
C	4.94897974012161	5.55823105883513	1.27272108874238
C	4.08297950905253	4.94113995002788	0.36471773117799
H	3.23439843715375	4.38292283814287	0.74022474869605
C	4.25594946640193	5.06028579718265	-1.00893144792180
H	3.56453367626182	4.57408545711212	-1.68574242351083
C	5.29864879417554	5.82902788914375	-1.50787530402007
H	5.43093071864698	5.94697910632773	-2.57575911049794
C	6.17087342845826	6.45762844707858	-0.62964935584236
H	6.98030325786486	7.06640685374995	-1.01313408914733
C	2.76750530102458	5.09287363596604	3.38467282866802
C	1.84654854018860	6.10739941231822	3.63876806876907
H	2.19471283594157	7.12933028468752	3.73366494029449
C	0.48435324660433	5.83351113782030	3.78188716274409
C	0.06032085231306	4.51380667128315	3.67232225764758
H	-0.99586910015242	4.28816411475339	3.78006618347357
C	0.95683957906493	3.46755524393581	3.43620942035575
C	2.30587587514885	3.77352534514873	3.29698989047928

H	3.01091140033429	2.96593898706088	3.12804130245677
C	-0.48976609145468	6.95033550574588	4.04384662409515
H	-0.26022968627678	7.45884591463875	4.98335376307506
H	-1.51399401989660	6.58097275412016	4.10081132776230
H	-0.44351474840105	7.70296953941764	3.25303601931681
C	0.46381503152818	2.04801483358191	3.34667511895054
H	0.01163404747038	1.72765398302289	4.28883477020554
H	1.27831266754758	1.36097826953769	3.11615584710508
H	-0.29827758026964	1.94158277363783	2.57068180698017
C	5.68876839697372	4.30935461716109	4.00202094599736
C	6.21486791985405	3.18752192054740	3.35159838176777
H	5.95038874768913	2.99309297256827	2.31706663634751
C	7.10841216406073	2.33115988281976	3.98928162180727
C	7.46776252879069	2.61049628361670	5.30824255438333
H	8.17882601632329	1.96250332244529	5.81001723553831
C	6.95532548128846	3.70728052951791	5.99451747771378
C	6.06860653464842	4.55162586968605	5.32396553832385
H	5.68946586877046	5.42633263958753	5.84030379696079
C	7.68694180022424	1.13684508838077	3.27854536052523
H	7.39971183655504	1.12459054203468	2.22679568464000
H	7.34167845777262	0.20320992370710	3.73051415097883
H	8.77833292267991	1.13570705747468	3.33201850477225
C	7.33429684827956	3.96660729054972	7.42608705675674
H	6.58302337065330	3.55109772181686	8.10452702176204
H	7.40709384778519	5.03276048823511	7.63355960953634
H	8.29274266315744	3.50878804535816	7.66979058212490
Mo	6.51058995446016	7.92473915132307	3.71385895429439
Si	5.43669623079105	10.90634954851393	2.90920550072039
O	5.55091920523767	9.67338523443965	3.94646419484829
C	7.02782452060748	8.44280805346390	1.64873185751903
H	6.40528112706253	8.99083065724381	0.96217651071987
C	8.11221562432148	9.13327834061618	2.33904767585804
C	8.88454019643821	8.40248179121893	3.20614587827447
H	9.62848455686611	8.90494347191209	3.80493827248067
C	9.54729322591805	11.09804989831227	1.94847012802179
H	10.37480623770905	10.40755846680331	1.84248818407424
C	9.76291670888187	12.46221705058015	1.81627498194334
H	10.75898855976804	12.83524744853584	1.61465126783106
C	8.69429616830850	13.33741282557880	1.94484988746996
H	8.84775000880928	14.40490287472600	1.84499092684693
C	7.42626538932610	12.84196912485035	2.22677594421826
H	6.61561392295645	13.54448491382879	2.37429648078963
C	7.17371769154206	11.47381597943077	2.36634376639960
C	8.27035828459103	10.59815395295715	2.20446331139572
C	4.62268624859581	12.34122067966504	3.79055854752046
C	3.98949874598474	13.38484083270840	3.10554842139667
H	3.90079793699579	13.34172398198376	2.02490422521713
C	3.45389778433209	14.47756617215581	3.77970139258279
C	3.56036443311669	14.51609186413206	5.17249179615321
H	3.15048135024248	15.36552336602020	5.70999753619716
C	4.16846844349143	13.49037794773096	5.88874169953800
C	4.68986496357544	12.40509357751859	5.18151983624205

H	5.15166680479315	11.58798553825426	5.72409394929984
C	4.47276284737514	10.44432179637307	1.36294705874327
C	3.55141474256768	9.39300051143621	1.40621190000812
H	3.38656764749169	8.87417484503487	2.34328797725974
C	2.88333361031542	8.96335603303673	0.26102342671903
C	3.15126546371896	9.60965471015547	-0.94584524113307
H	2.65126003470534	9.27046604307939	-1.84768516313901
C	4.05223191854474	10.66875628488829	-1.02724540985946
C	4.70831171279611	11.07015998337710	0.13646367663266
H	5.44272884754384	11.86665886263973	0.07186717606891
C	4.28341795976212	13.54499189407958	7.38858928115039
H	5.32951266591802	13.62245015187828	7.69716531711441
H	3.88095963458456	12.63926864926715	7.84766912508538
H	3.74795983214724	14.40162713398789	7.79929179719629
C	2.76081902807210	15.58944284360483	3.03755917724157
H	3.17674589691013	16.56441057775121	3.30310737169486
H	1.69417724444775	15.61795172716334	3.27641595794943
H	2.85684380427411	15.46311202649719	1.95882507465808
H	5.30093009842537	11.81071676911556	-2.36393060641963
H	4.21207416560170	10.68054015231097	-3.17983167954112
H	1.92785772444001	7.22759955876579	-0.58643370352007
H	2.06501539215637	7.18778758239172	1.17124282492327
H	0.86703418305590	8.22589503344740	0.40570064426916
H	3.58319603731744	12.17530206543540	-2.49495267236085

Compound 12 (S=3/2)

Si	8.31045049750563	8.10657682415061	6.48518621688853
Si	4.60913816996531	5.65527927673228	3.26780387992723
O	6.88107134885754	8.05463180511988	5.71738505615810
O	4.88604687966681	7.12955892453181	3.88871972758224
C	8.68487313198334	7.11720296298587	3.40358825558692
C	7.75364467905544	6.45968232795707	2.57903472684599
H	7.59186166223954	5.40162006061033	2.70624304529460
C	6.97537012743477	7.17866935721797	1.62596079668017
C	4.50590855275767	10.93070923042844	-2.48003672569207
C	1.84280538293112	7.87210788979349	0.50093081794845
C	9.43256446974904	6.35481550673560	4.43419713423676
C	9.42605131132679	6.72964021808426	5.79542402846861
C	10.17374360698139	5.94447697562575	6.67814324702672
H	10.16506383948780	6.18564748125671	7.73350043215078
C	10.89411511374532	4.83435639229497	6.25279931367016
H	11.45897526917126	4.24961689335742	6.96824086655095
C	10.86489886015901	4.46519572818420	4.91589862811598
H	11.40560396516808	3.59201024303812	4.57387152492865
C	10.13218726465670	5.22309098404823	4.01399748487951
H	10.10825424382309	4.94158221184183	2.96857884315155
C	8.01665062870927	7.79252032404252	8.30175637555302
C	6.81013774920402	7.22796035592593	8.71115268543340
H	6.04547974059527	7.02232549062348	7.97076282083489
C	6.56081929138148	6.93496327793224	10.05381529642717

C	7.54800849744757	7.22634297166336	10.98879120526075
H	7.36549153270117	7.00589162640860	12.03583103649711
C	8.76423026447412	7.80722265125387	10.61879702449794
C	8.98048657774511	8.08644830275172	9.27390432717339
H	9.91435992617197	8.55572941354984	8.98156166371182
C	5.25381484081685	6.31193155831560	10.46504550815667
H	4.40751323791693	6.91219183082621	10.12347499877669
H	5.18337265123374	6.21087059599081	11.54847425230044
H	5.13883980085049	5.31740021992436	10.02585093618294
C	9.80665019956728	8.11975887597255	11.65921042594669
H	9.39643315279721	8.74524376853751	12.45567428089569
H	10.65543968175790	8.64591319900495	11.22161808687978
H	10.18337496708259	7.20667473527400	12.12821941227800
C	9.14882230043439	9.76389934948638	6.22683623735455
C	10.54097454671676	9.89303680904819	6.18533493803257
H	11.16674059358710	9.01644337844112	6.32100634391360
C	11.15119959801531	11.11975598796400	5.93355609133627
C	10.33761605660948	12.23421710059876	5.73095344101474
H	10.79897002708828	13.19378439199723	5.52014451473573
C	8.94838917459287	12.14973680175582	5.78129327209406
C	8.36776924265642	10.90334635557742	6.01895860256872
H	7.28762482990601	10.81305515284321	6.01190067582375
C	12.65045193855510	11.24860553638018	5.88221958870171
H	13.02829053024196	11.80174954869436	6.74659508379262
H	12.97240968046481	11.78851784898308	4.98876069529880
H	13.13195079233619	10.27013451950371	5.87887799750579
C	8.09943350057803	13.37944270907787	5.61229093298947
H	7.91571167680813	13.85409100457395	6.58109207025012
H	7.13077278017847	13.14121418022465	5.17765227110526
H	8.58821237332223	14.11388120425420	4.97177551182607
C	5.94733452371828	6.49013838984650	0.80740484175312
C	4.92063837666904	5.71684738448587	1.39239659147952
C	4.01610573092599	5.09580776261749	0.52617000925648
H	3.19878151092317	4.52058887168686	0.94261404263222
C	4.10386677046927	5.22849815127374	-0.85486485359519
H	3.38215855500448	4.73312316415803	-1.49252554605497
C	5.10091665533582	6.01789820645930	-1.40921479817000
H	5.16913359717928	6.14599782420167	-2.48190119095791
C	6.01514676318991	6.64785868405617	-0.57709514190670
H	6.79862973814594	7.26130625923124	-1.00440178354360
C	2.81960626235951	5.21353260350803	3.56035845485075
C	1.90184401827524	6.22294107229081	3.84400845919501
H	2.25326113595506	7.24370835464901	3.94250366287239
C	0.54314051672537	5.94654444224954	4.01287315594245
C	0.11806033071902	4.62709635752477	3.90291422216260
H	-0.93501997502939	4.39856099825752	4.03341164845736
C	1.01149764826715	3.58504893896384	3.63932484545075
C	2.35674734776985	3.89515561184047	3.47219666776180
H	3.05963297373357	3.09059052462564	3.28149704853222
C	-0.42857680281655	7.06127504811399	4.29340387452922
H	-0.13070292942630	7.63040286213379	5.17695396321997
H	-1.43674382261860	6.68013975600897	4.45850882972305

H	-0.46641854391838	7.76456425869511	3.45731409255129
C	0.52006081013988	2.16469696791478	3.55029594590332
H	0.07593586542248	1.84067421131735	4.49506485332633
H	1.33425126526370	1.48009176854280	3.31148460022105
H	-0.24799121280318	2.05830384973102	2.77997801956782
C	5.74219459853953	4.37695249248479	4.04203121867128
C	6.20926181549762	3.26978542317561	3.32565300616459
H	5.89970488363320	3.12751006188957	2.29500140666529
C	7.09922669947915	2.35944420422872	3.89067538284811
C	7.51450475900008	2.56913057932624	5.20569471999418
H	8.21868637573126	1.87564424159199	5.65421631049743
C	7.05897164486280	3.64907900408520	5.95742301009297
C	6.18011495855537	4.55190234416443	5.35671270552919
H	5.85391563814516	5.42270460705948	5.91356583085850
C	7.60305563468778	1.17493118784909	3.10966023861679
H	7.34250818719307	1.25740780309021	2.05395599985397
H	7.17270974321279	0.24275563949992	3.48621027058195
H	8.68884842255799	1.08182442542463	3.18580422432444
C	7.47997662926865	3.81902475775121	7.39099081027430
H	6.75003520056564	3.35547726423668	8.06169987362288
H	7.55146835379371	4.86945861207089	7.66636115958105
H	8.44649486152624	3.35121845243870	7.57924859037764
Mo	6.55921116600264	8.17612583522070	3.77106517851247
Si	5.46546745497349	11.06149020200283	2.81916249043019
O	5.59938677610619	9.89629052773253	3.94199913386056
C	7.18948741493182	8.56215647977539	1.48029568534524
H	6.59648884965648	9.11443320787372	0.76924971318664
C	8.12587260422743	9.25395539276626	2.30305452393268
C	8.88096203079397	8.51914160694589	3.23661979708264
H	9.58324934429290	9.03524723485152	3.87136894329728
C	9.59288935713783	11.20624819924716	1.99322920216210
H	10.41698811339818	10.50662486415428	1.92907216240193
C	9.83093288133347	12.56681861583199	1.86175244160938
H	10.83961266738620	12.92572177513533	1.70199997046902
C	8.76946727958573	13.45677647532863	1.93871310554433
H	8.94184513147269	14.52143613922882	1.84082122434562
C	7.48428157928250	12.97969837123712	2.16919048269207
H	6.67650869642193	13.69180305299630	2.28132078748133
C	7.21057931557118	11.61567357264508	2.30642110560640
C	8.29922357109007	10.72361736351934	2.19359822455006
C	4.57507021217763	12.51647985214721	3.57754114813536
C	4.00458675660063	13.52861116822868	2.79649813929186
H	4.02691504275096	13.45050754113551	1.71431291835131
C	3.39116357724427	14.63411316998365	3.37577297657184
C	3.35173801681463	14.71625931664890	4.77038512942424
H	2.87877919378862	15.57578838921214	5.23488971232867
C	3.89329605387671	13.72257106106747	5.57873450522114
C	4.49904420170948	12.62402873441215	4.96490670615795
H	4.91357954570966	11.83218084516861	5.57844552665256
C	4.54832050335441	10.43933903294238	1.30626204498782
C	3.58538205789266	9.43610075994729	1.44469222563570
H	3.37836598601741	9.03090781075657	2.42850206893425

C	2.91262753629516	8.91683941612446	0.33828438086010
C	3.23739458902111	9.40741250477564	-0.92475435962619
H	2.73945162093886	8.99100197234560	-1.79457409499345
C	4.19101200090649	10.40850521759652	-1.10340784692007
C	4.83450991993475	10.91654107607906	0.02339965301998
H	5.59728206540081	11.67757635758156	-0.10849927162021
C	3.84430440623485	13.82233676846595	7.07975496984246
H	4.85106869671510	13.88825977374589	7.50071928577616
H	3.37173656023395	12.93993330928617	7.51710533189935
H	3.28653215008529	14.70174077783895	7.40311194288654
C	2.76636003392385	15.71222238264831	2.53071366121782
H	3.12877415517786	16.70229741921150	2.81740745572861
H	1.67895156822912	15.72109570652434	2.64457164157618
H	2.98860857918412	15.56327494780987	1.47372750470298
H	5.46788201240306	11.44452399997015	-2.49840781325635
H	4.53597519211599	10.12309675121118	-3.21416507809615
H	1.78556334328080	7.22422488809355	-0.37400718935878
H	2.02105436628799	7.24815567436249	1.37432561359017
H	0.86347182997198	8.34353573023063	0.62870885296915
H	3.74551071701326	11.64294093180101	-2.81339709366854

Molecular Orbital Analysis of Selected Compounds

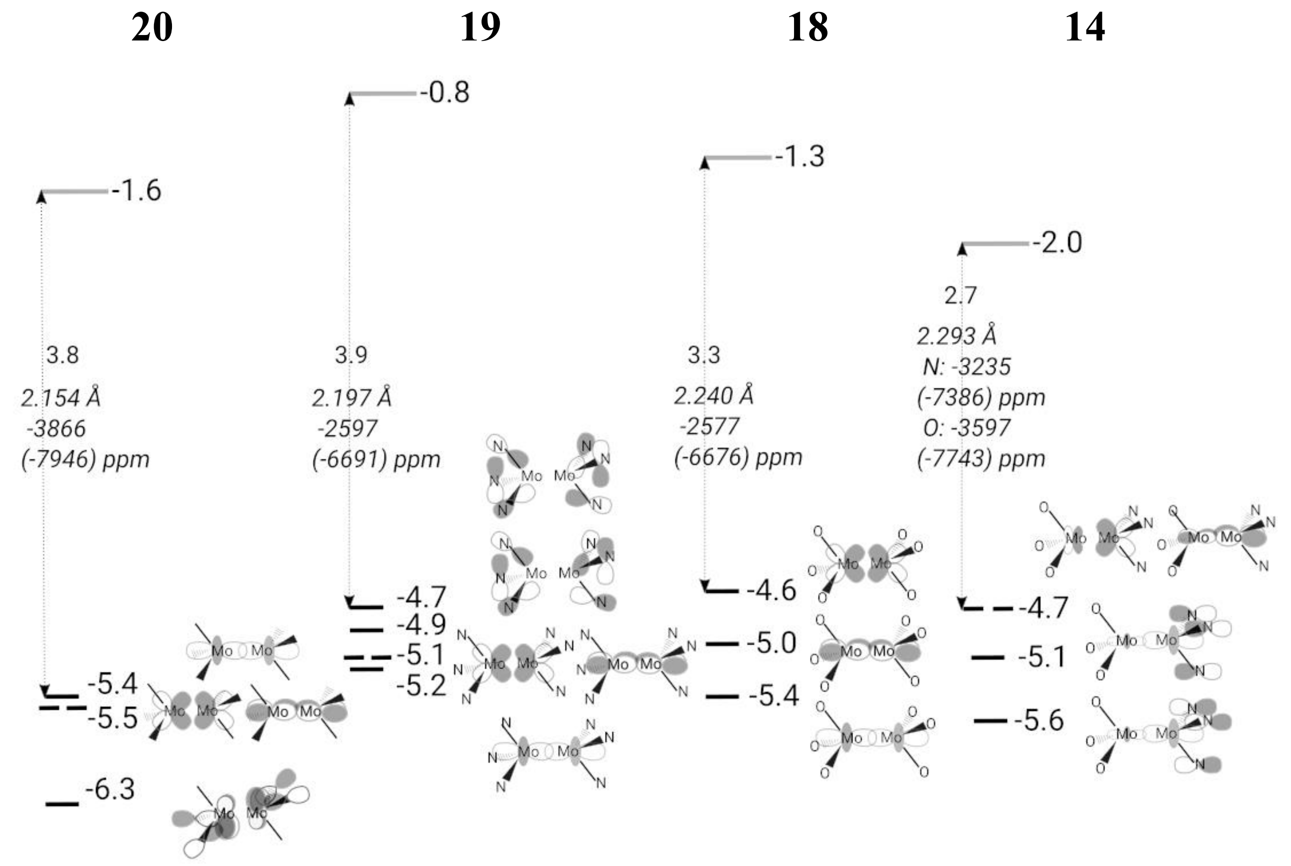

Figure S1. MO energies [eV] (TPSSh/TZVPP + CPCM(Toluene)), computed Mo-Mo bond lengths [Å], computed isotropic absolute isotropic chemical shieldings [pmm] (paramagnetic contribution given in parenthesis) together with schematic representations of the frontier occupied molecular orbitals.

In order to assess the complex relation between bond lengths, electronic structure and chemical shifts, we carried out density functional theory (DFT) calculations. A detailed analysis shows that while the Mo-Mo bond length and the Mo NMR chemical shifts show pronounced differences between the different compounds, there is only a very poor correlation between the structural parameters and the NMR chemical shifts. A closer inspection of the frontier orbitals exhibits what has already been discussed by Bursten et al. [JACS 1980, 102, 4579] for Mo_2L_6 compounds with N,O and C containing ligands: The frontier orbitals that play a crucial role for the molecluar structure and the NMR chemical shift do change drastically in their eneriges and most importantly in their character.

On the one hand, the MOs of the Mo-Mo bond that arise from Mo d-orbitals do change in the order of σ and π orbitals. On the other hand, high-lying lone pair orbitals of N atoms can lead to MOs that lie even higher in energy. And while the shielding of the homo-dimers roughly scales with the energy of the corresponding HOMO orbitals, the shielding of the hetero-dimers is dominated by a polarization of the frontier orbitals due to the different electronegativity of the ligand atoms and a further effect of the steric bulk cannot be excluded as well.

Detailed MO plots of the frontier orbitals

Compound 20:

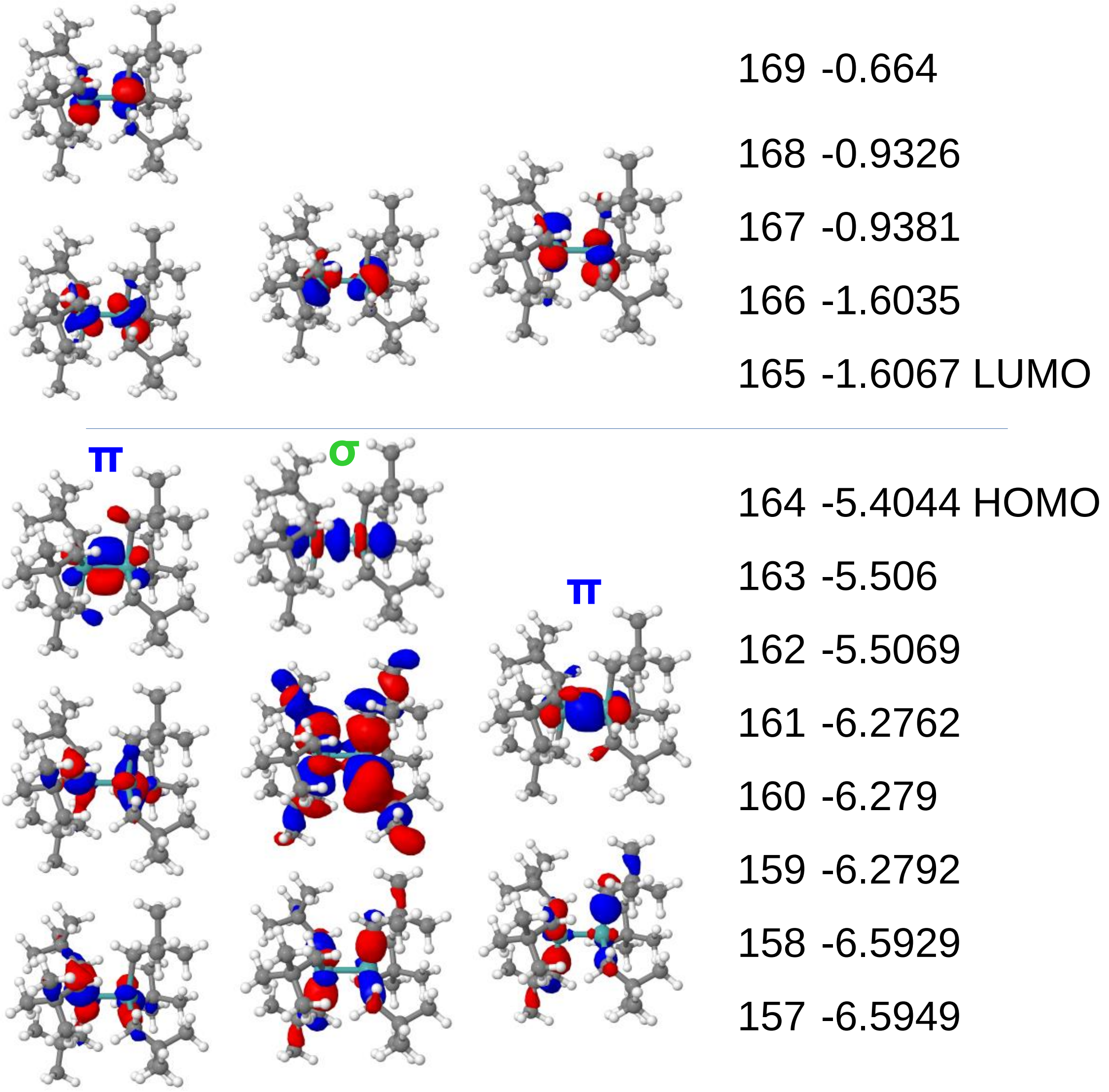

Compound 19:

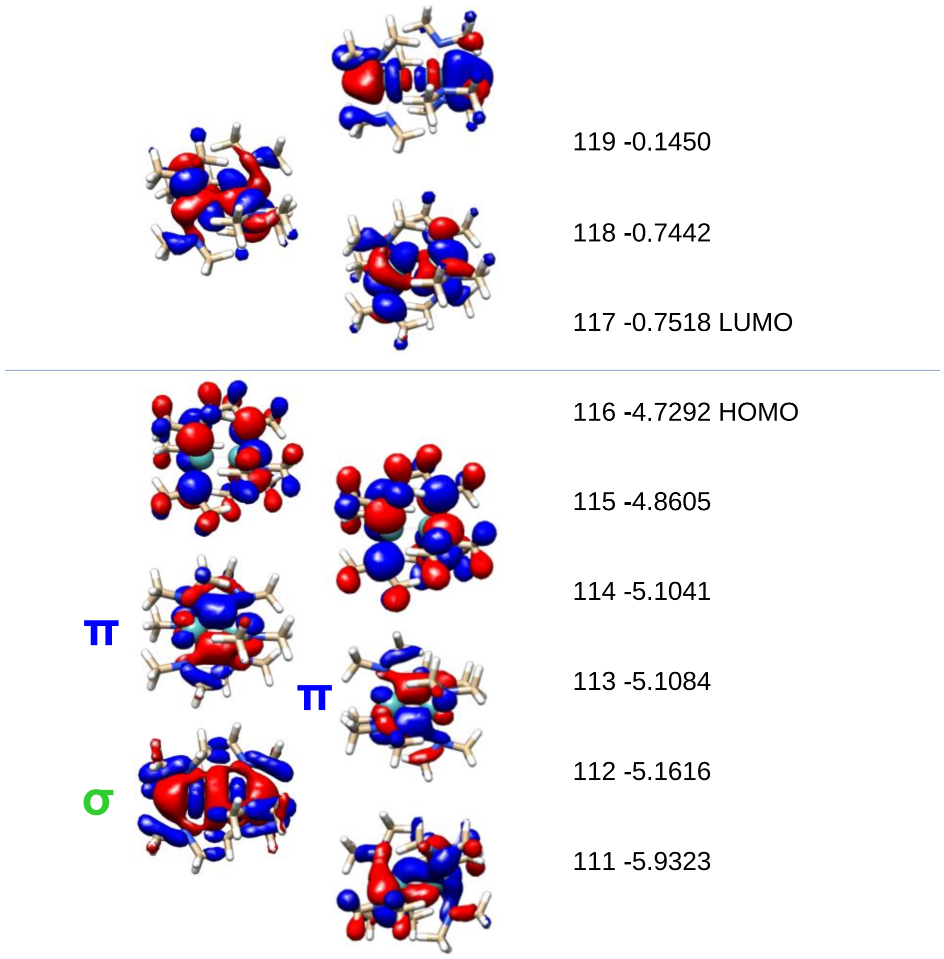

119 -0.1450
118 -0.7442
117 -0.7518 LUMO
116 -4.7292 HOMO
115 -4.8605
114 -5.1041
113 -5.1084
112 -5.1616
111 -5.9323
π
π
σ

Compound 18:

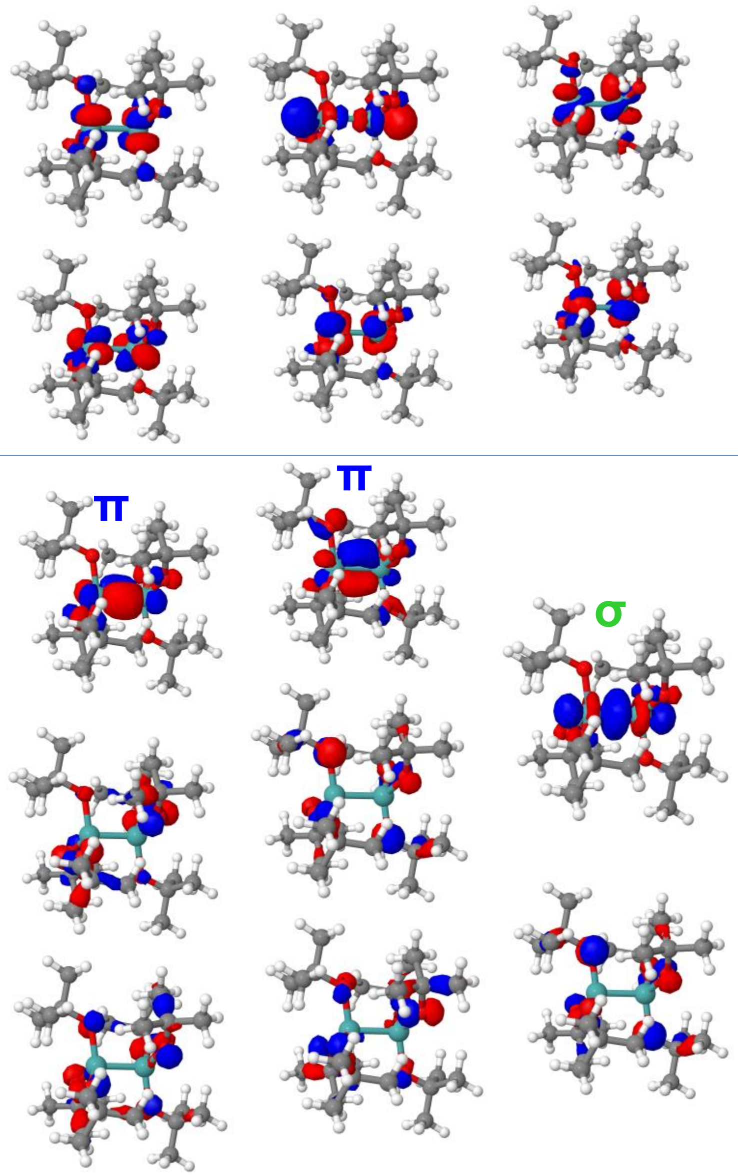

170 0.0266
169 -0.0022
168 -0.2802
167 -0.436
166 -1.1697
165 -1.3055 LUMO

164 -4.6498 HOMO
163 -4.9579
162 -5.4492
161 -6.6136
160 -6.7594
159 -6.7973
158 -7.134
157 -7.2332

Compound 14:

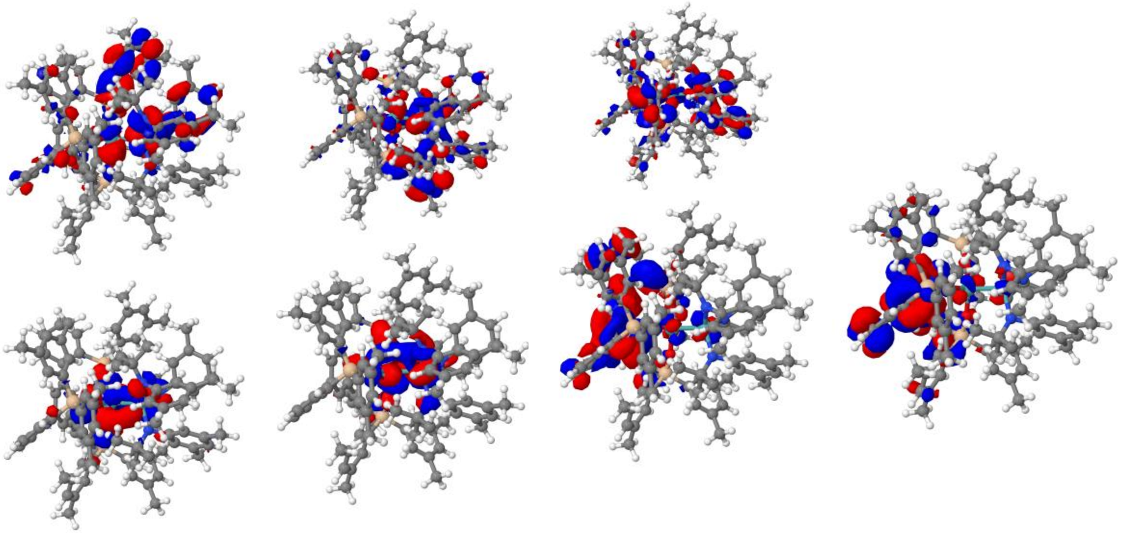

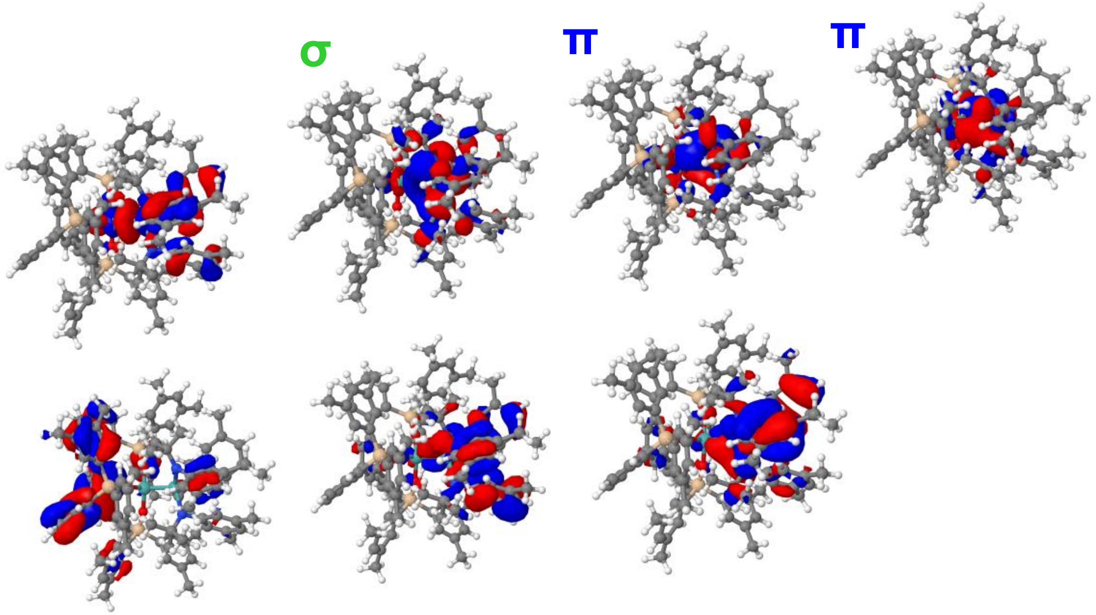

477	-1.0604
476	-1.0633
475	-1.0729
474	-1.3436
473	-1.3488
472	-1.9404
471	-1.9505 LUMO
470	-4.6783 HOMO
469	-4.6819
468	-5.0571
467	-5.6065
466	-5.766
465	-5.7677
464	-5.8964

References

[1] (a) Becke, A. D., *J. Chem. Phys*. **1993**, *98*, 1372. (b) Lee, C.; Yang, W.; Parr, R. G., *Phys. Rev. B* **1988**, *37*, 785. (c) Andrae, D.; Haeussermann, U.; Dolg, M.; Stoll, H.; Preuss, H., *Theor. Chim. Acta* **1990**, *77*, 123. (d) Weigend, F.; Ahlrichs, R., *Phys. Chem. Chem. Phys*. **2005**, *7*, 3297. (e) Weigend, F., *Phys. Chem. Chem. Phys*. **2006**, *8*, 1057. (f) Weigend, F., *J. Comput. Chem*. **2008**, *29*, 167. (g) Grimme, S.; Antony, J.; Ehrlich, S.; Krieg, H., *J. Chem. Phys*. **2010**, *132*, 154104. (h) Grimme, S.; Ehrlich, S.; Goerigk, L., *J. Comput. Chem*. **2011**, *32*, 1456. (i) Garcia-Rates, M.; Neese, F., *J. Comput. Chem*. **2019**, *40*, 1816. (j) Garcia-Rates, M.; Neese, F., *J. Comput. Chem.* **2020**, *41*, 922.

[2] (a) Perdew, J. P.; Kurth, S.; Zupan, A.; Blaha, P., *Phys. Rev. Lett*., **1999**, *82*, 2544. (b) Tao J.; Perdew, J. P.; Staroverov, V. N.; Scuseria, G. E., *Phys. Rev. Lett*. **2003**, *91*, 146401. (c) Staroverov, V. N.; Scuseria, G. E.; Tao, J.; Perdew, J. P., *J. Chem. Phys*. **2003**, *119*, 12129. (d) Perdew, J. P.; Tao, J.; Staroverov, V. N.; Scuseria, G. E., *J. Chem. Phys*. **2004**, *120*, 6898. (e) Rolfes, J. D.; Neese F.; Pantazis, D. A., *J. Comput. Chem*. **2020**, *41*, 1842. (f) Stoychev, G. L.; Auer, A. A.; Neese, F., *J. Chem. Theory Comput*. **2017**, *13*, 554. (g) Wolinski, K.; Hinton, J. F.; Pulay, P., *J. Am. Chem. Soc*. **1990**, *112*, 8251. (h) van Lenthe, E.; Baerends, E. J.; Snijders, J. G., *J. Chem. Phys*. **1993**, *99*, 4597. (i) van Lenthe, E.; Baerends, E. J.; Snijders, J. G., *J. Chem. Phys*. **1994**, *101*, 9783. (j) Sadlej, A. J.; Snijders, J. G.; van Lenthe, E.; Baerends, E. J., *J. Chem. Phys*. **1995**, *102*, 1758. (k) Staroverov, V. N.; Scuseria, G. E.; Tao, J.; Perdew, J. P., *J. Chem. Phys*. **2003**, *119*, 12129. (l) Rolfes, J. D.; Neese, F.; Pantazis, D. A., *J. Comput. Chem*. **2020**, *41*, 1842. (m) Stoychev, G. L.; Auer, A. A.; Izsak, R.; Neese, F., *J. Chem. Theory Comput*. **2018**, *14*, 619. (n) Dobson, J. F., *J. Phys. Condens. Matter* **1992**, *4*, 7877.

[3] (a) Caló, F. P.; Bistoni, G.; Auer, A. A.; Leutzsch, M.; Fürstner, A. *J. Am. Chem. Soc.* **2021,** *143*, 12473. (b) Gui, X.; Sorbelli, D.; Caló, F. P.; Leutzsch, M.; Patzer, M.; Fürstner, A.; Bistoni, G.; Auer, A. A., *Chem. Eur. J.* **2024**, *30*, e202301846.

[4] (a) Neese, F., *WIRES Comput. Molec. Sci*. **2022**, *12*, e1606. (b) Neese, F., *WIRES Comput. Molec. Sci.* **2012**, *2*, 73-78. (c) Neese, F., *WIRES Comput. Molec. Sci*. **2018**, *8*, 1-6. (d) Neese, F., Wennmohs, F.; Becker, U.; Riplinger, C., *J. Chem. Phys*. **2020**, *152*, L224108. (e) Neese, F., J. *Comp. Chem*. **2003**, *24*, 1740-1747. (f) Neese, F.; Wennmohs, F.; Hansen, A.; Becker, U., *Chem. Phys.* **2009**, *356*, 98-109. (g) Helmich-Paris, B.; de Souza, B.; Neese, F.; Izsák, R., *J. Chem. Phys*. **2021**, *155*, 104109. (h) Neese, F., *J. Comp. Chem*. **2023**, *44*, 381.
